# A practical and scalable system for heteroaryl amino acid synthesis†
†Electronic supplementary information (ESI) available. See DOI: 10.1039/c7sc03612d


**DOI:** 10.1039/c7sc03612d

**Published:** 2017-10-02

**Authors:** R. A. Aycock, D. B. Vogt, N. T. Jui

**Affiliations:** a Department of Chemistry and Winship Cancer Institute , Emory University , Atlanta , GA 30322 , USA . Email: njui@emory.edu

## Abstract

A robust system for the preparation of β-heteroaryl α-amino acid derivatives has been developed using photoredox catalysis. This system operates *via* regiospecific activation of halogenated pyridines (or other heterocycles) and conjugate addition to dehydroalanine derivatives to deliver a wide range of unnatural amino acids. This process was conducted with good efficiency on large scale, the application of these conditions to amino ketone synthesis is shown, and a simple protocol is given for the preparation of enantioenriched amino acid synthesis, from a number of radical precursors.

## Abstract 2

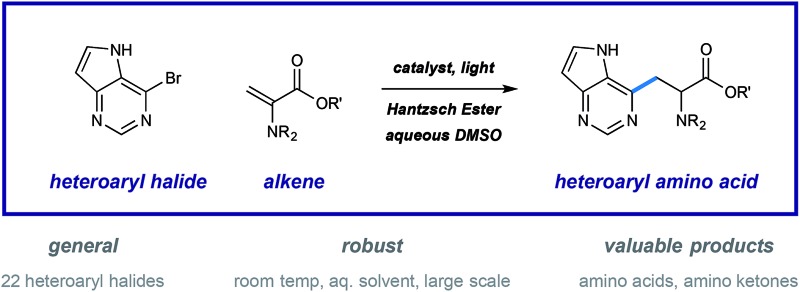
Here, we describe a highly-effective catalytic method for the synthesis of heteroarene-containing unnatural amino acids.

## Introduction

Amino acids play a central role in the chemical and biological sciences. As primary members of the chiral pool, they are precursors to drugs,^1^ chiral auxiliaries,^2^ and catalysts.^3^ In addition, they are fundamental building blocks for the construction of biomolecules. The use of peptides as therapeutic agents is attractive because they can display extremely diverse, potent, and selective biological activities.^4^ However, there are significant challenges in peptide drug design, including low metabolic stability or poor physical properties. One proven strategy for overcoming these challenges involves substitution of the native residues with unnatural amino acids (synthetic mutagenesis).^5^ Nitrogen-containing heteroaromatics are common in pharmaceuticals because they directly alter the solubility, metabolic stability, and binding affinity of the molecules that they comprise.^6^ As such, heteroarene-containing unnatural amino acids are promising tools in the design of peptide therapeutics.

**Fig. 1:**
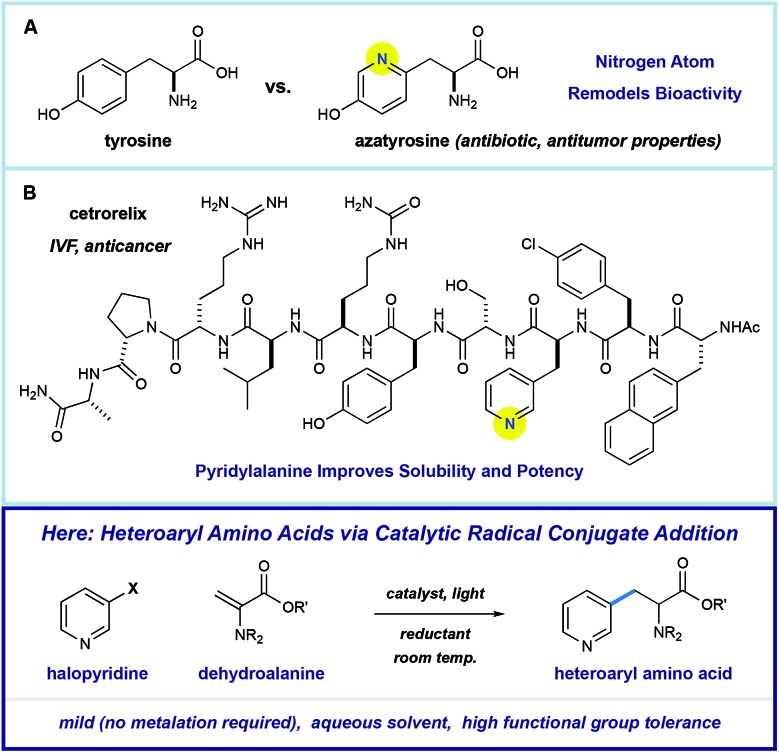
Impact of pyridine incorporation into amino acids and peptide drugs.

Pyridine incorporation has a dramatic impact on the properties of amino acids and peptides. For example, azatyrosine—a natural product that differs from the essential amino acid tyrosine by substitution of a single atom—displays potent antibiotic and antitumor properties (Fig. 1A).^7^ Installation of the 3-pyridylalanine (3-pyr-Ala) residue in the gonadotropin-releasing hormone antagonist cetrorelix (Fig. 1B) was found to improve both aqueous solubility and receptor affinity,^8^ and similar effects were observed in the development of other peptide hormones (not shown).5b–d As part of a program centered on the catalytic functionalization of heteroaromatics, we target the development of impactful synthetic methods for the construction of novel β-heteroaryl α-amino acids through a radical conjugate addition mechanism.

We have found that pyridyl halide activation *via* single electron reduction using photoredox catalysts^9^ can be accomplished, and that the intermolecular reactivity of the resulting radical species can be dictated by the reaction conditions.^10^,^11^ More specifically, we found that pyridyl radicals display nucleophilic reactivity in aqueous DMSO, and they readily couple with electron-poor alkenes. We questioned whether this approach could be translated to heteroaryl amino acid synthesis through radical conjugate addition to dehydroalanine derivatives. There are a number of powerful methods for the synthesis of unnatural β-heteroaryl α-amino acids, including malonate (or enolate) alkylation,^12^ cross-coupling of serine-derived organometallic reagents,^13^ and reduction of dehydroamino acid derivatives.^14^ However, strategies based on radical addition to DHA derivatives are unique due to the highly-chemoselective nature of radical species, and the broad functional group tolerance that results.^15^ Alkyl radical addition to DHA has been effectively accomplished even in the complex setting of intact proteins.^16^ While this is a highly attractive attribute, a radical approach to heteroaryl amino acids is currently unknown. Here, we describe the successful translation of our reductive heteroarene activation system to amino acid synthesis.

## Results and discussion

**Fig. 2:**
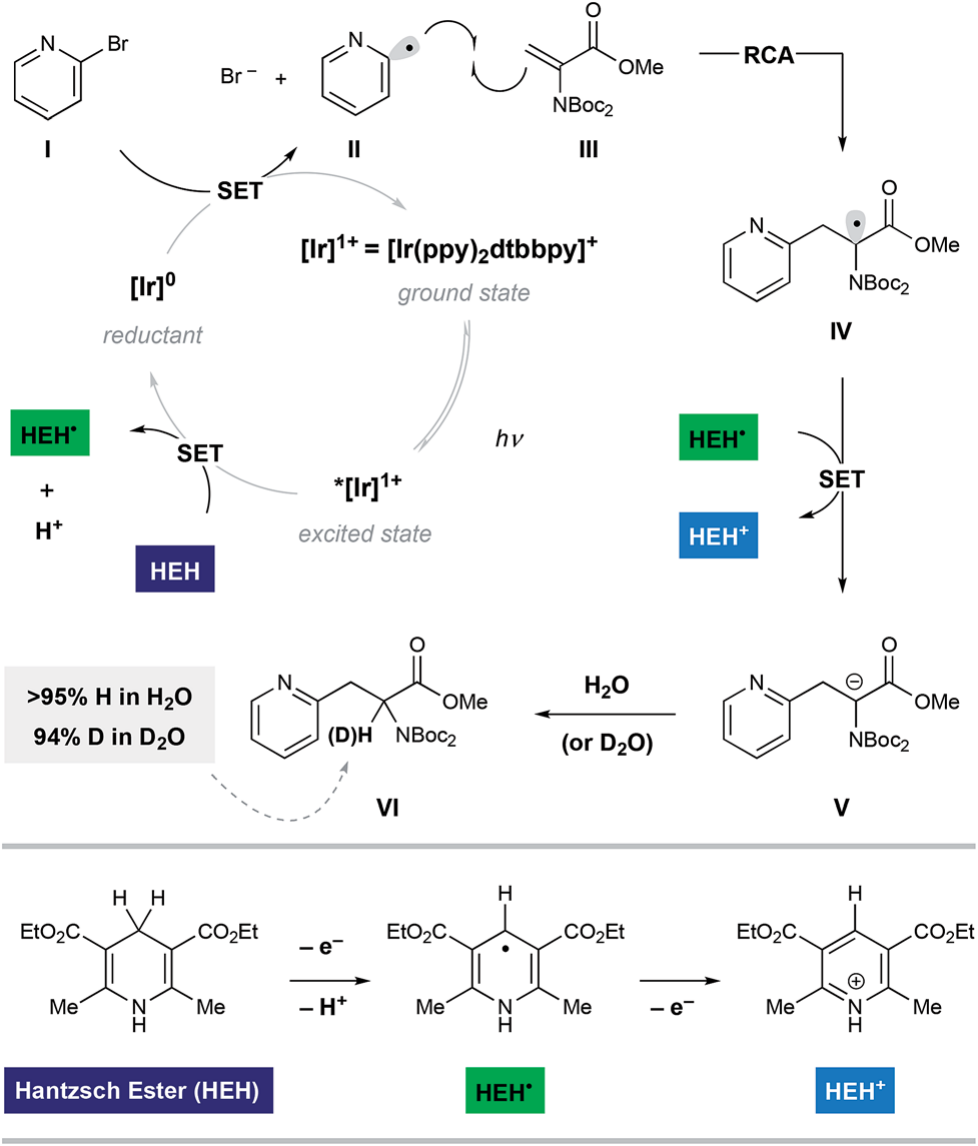
A proposed mechanism of heteroaryl radical conjugate addition to dehydroalanine.

Shown in Fig. 2 is a mechanistic picture that is consistent with our observations. Excitation of the photocatalyst [Ir(ppy)_2_(dtbbpy)]PF_6_ ([Ir]^1+^), followed by reductive quenching of the excited state by Hantzsch ester (HEH) gives rise to the [Ir]^0^ (*E*_1/2_ = –1.51 V).^17^ Stern–Volmer quenching studies indicated that Hantzsch ester is the most significant excited state quencher (see ESI for details†). Single electron reduction of halo pyridine **I**, followed by rapid mesolytic cleavage in polar solvents (X = Br, I)^18^ affords heteroaryl radical intermediate **II**, which exhibits nucleophilic radical behavior in aqueous DMSO.10*a* It is possible that halopyridine reduction is assisted by protonation, as each catalytic turnover produces an nominal equivalent of Hantzsch pyridinium bromide (HEH^+^ Br^–^). Hydrodehalogenation (HDH) of the arene is observed as a common byproduct, but this undesired pathway can be suppressed by limiting the solubility of the stoichiometric reductant, Hantzsch ester (HEH), in accord with our previous findings. Radical conjugate addition (RCA) to dehydroalanine **III** and subsequent single electron reduction of the nascent radical **IV** would deliver the corresponding enolate **V**. The intermediacy of **V** is supported by the fact that the α-H amino acid product **VI** is produced in the presence of H_2_O as a cosolvent (regardless of H/D labeling of HEH). Conversely, when D_2_O is used as a cosolvent, complete deuterium incorporation is obtained at the α-position.

As illustrated in Table 1, we identified conditions that efficiently unite 2-bromo-5-hydroxypyridine with the indicated dehydroalanine derivative (readily accessed on 35 g scale from Boc-Ser-OMe) to give the protected azatyrosine **1** in 98% NMR yield (entry 1). These conditions employ 1 mol% of the photosensitizer [Ir(ppy)_2_(dtbbpy)]PF_6_ (excited by irradiation with a commercial blue LED) and Hantzsch ester (1.5 equiv.) as a stoichiometric reductant in aqueous DMSO. Control experiments indicated that all of these components are necessary for the reaction (entries 2–4, 0% yield), and that use of the prototypical Ru(bpy)_3_^2+^ chromophore results in product formation, although with diminished efficiency (entry 5, 58% yield). Omission of water as a cosolvent was not well tolerated here (entry 6, 14% yield), a finding that is in consistent with our previous observations.10*a* We found that other aqueous solvent mixtures can be used (entries 7 and 8, 35% and 71% yield, respectively), and that this photoredox system is remarkably robust; an experiment using bourbon as solvent (open to air) afforded the desired product in 93% yield (entry 9). Importantly, protection of the phenol O–H function was not required under these mild radical conditions.

**Table 1:** Optimal conditions for pyridyl radical addition to a dehydroalanine substrate^*a*^

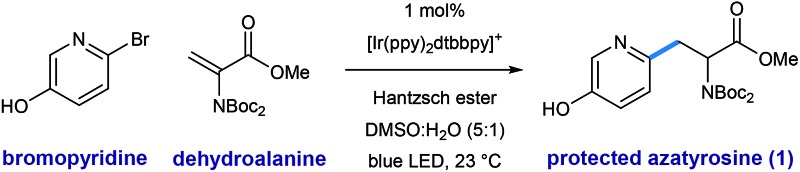		
Entry	Deviation from optimal conditions	Yield of 1^*b*^
1	None	98%
2	Without Hantzsch ester	0%
3	Without light	0%
4	Without catalyst	0%
5	Ru(bpy)_3_Cl_2_ as catalyst	58%
6	Without H_2_O	14%
7	DMF : H_2_O (5 : 1) as solvent	35%
8	EtOH : H_2_O (5 : 1) as solvent	71%
9	Bourbon as solvent (open to air)	93%

^*a*^Conditions: 2-bromo-5-hydroxypyridine (0.2 mmol), dehydroalanine (0.4 mmol), Ir(ppy)_2_dtbbpy·PF_6_ (1 mol%), Hantzsch ester (0.3 mmol), H_2_O (0.33 mL), DMSO (1.66 mL), blue LED, 23 °C, 16 h.

^*b*^Yield determined by NMR.

Using the optimized protocol outlined above, we found that the heteroaryl halide scope of this transformation is broad (as shown in Table 2). Some reactions are complete in as little as 2 hours, but each experiment was conducted overnight (16 h) for consistency and convenience without negatively impacting the yields. Regiospecific activation of each pyridyl position is possible *via* single electron reduction, and these conditions effectively delivered amino ester products from 2- and 3-iodopyridine (**2** and **10**), in 97% and 73% yield, respectively. Although less efficient, 4-iodopyridine also affords 4-pyridylalanine in useful yield (**16**, 34% yield), where reductive pyridine production is a significant alternative pathway. Methyl substitution is well-tolerated at all positions of 2-bromo pyridines, cleanly furnishing the corresponding pyridylalanines **3–6** in very high yield (93–97% yield). Reaction of 2-bromo-5-trifluoromethylpyridine (**7**) efficiently afforded product in 94% yield. Electron-donating groups are well-tolerated including amino (**9**, 71% yield), phenol (**11**, 67% yield), amide (**12**, 73% yield), and methoxy (**17**, 66% yield) groups. Dihalogenated pyridines can be programmed for regiospecific radical formation and subsequent conjugate addition at any position, preserving 2-chloro-substituents in the presence of more reactive iodo-substituents. Coupling reactions of 2-chloro-3-iodo- (**14**), 2-chloro-4-iodo-(**18**), 2-chloro-5-iodo-(**13**), and 2-chloro-3-methyl-4-iodopyridine (**19**) each gave single pyridylalanine products in good yield (73–83% yield). 2,5-Diiodopyridine is selectively activated at the more electrophilic 2-position to afford the corresponding amino ester (**8**) as a single regioisomer in 74% yield. We found that halopyrimidines are also viable substrates in this process: 4-iodo-2-(methylthio)pyrimidine (**15**) and 4-bromodeazapurine (**21**) gave product in 80% and 95% yield respectively. This photoredox process is amenable to gram-scale preparation of heteroaryl amino acid synthesis, without the need for special equipment. We reacted 25 mmol of 2-bromopyridine with a slight excess (1.2 equivalents, 30 mmol) of the dehydroalanine substrate. In the presence of 1.0 equivalent of Hantzsch ester, in the presence of 1.0 equivalent of Hantzsch ester, and only 0.1 mol% (23 mg) of the iridium photoredox catalyst, the desired pyridylalanine derivative **2** was produced in 84% yield (8.0 g) after purification. As anticipated, selective unveiling of the amine and acid groups (in compound **2**) using standard conditions went without issue. Hydrolysis of the methyl ester (2.0 equiv. of LiOH in THF/H_2_O) occurred with preservation of both Boc groups. Exposure of **2** to trifluoroacetic acid in CH_2_Cl_2_ revealed the free amine as the TFA salt while leaving the methyl ester intact. Finally, sequential treatment of **2** with KOH in EtOH/H_2_O followed by direct acidification of the reaction mixture with HCl afforded the fully deprotected 2-pyridylalanine as the double HCl salt. Each of these processes occurred in high yield at room temperature (see ESI for details†).

**Table 2:** Catalytic amino acid synthesis: scope of the halogenated heteroarene^*a*^

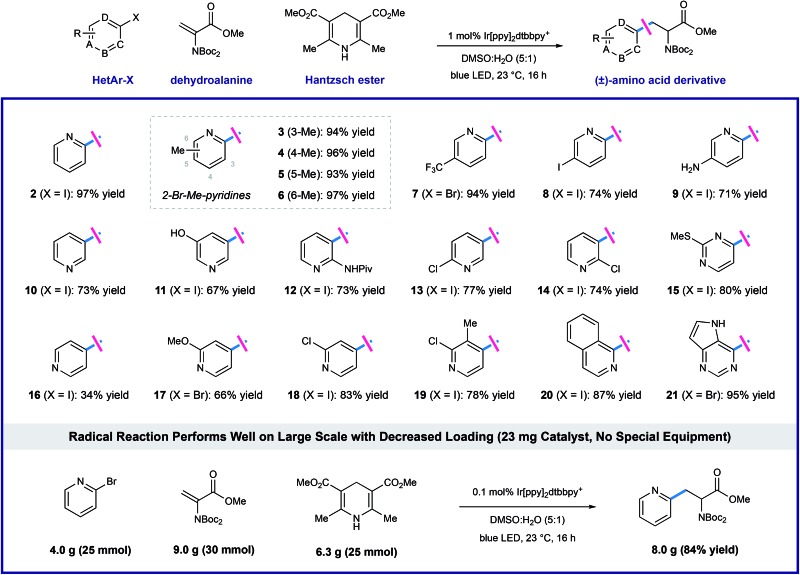

^*a*^Conditions: halogenated heteroarene (1.0 mmol), dehydroalanine (2.0 mmol), Ir(ppy)_2_dtbbpy·PF_6_ (1.0 mol%), Hantzsch ester (HEH, 1.3–1.5 mmol), H_2_O (1.7 mL), DMSO (8.3 mL), blue LED, 23 °C, 16 h, isolated yields shown.

We conducted a brief evaluation of the scope of amino-substituted alkenes with the expectation that this reaction template could be flexibly utilized to deliver other amino acid or amino-carbonyl substructures. We found that dehydroamino acid substrates with methyl- and phenyl-substituents in the β-position could be successfully employed, giving rise to products **22** and **23** in acceptable yield (66% and 54% yield, respectively) with modest diastereocontrol. Replacement of the α-imide group in the alkene starting material (a structural artifact of dehydroalanine synthesis *via* Boc_2_O-induced β-elimination) with an N–H aniline group or electronically diverse arylmethylamine groups was tolerated, although diastereoselectivity was low (**25–28**, 66–75% yield, ≤3 : 1 dr). These radical conjugate addition conditions directly translated to the synthesis of β-heteroaryl α-amino ketone derivatives **29–31**, giving the desired products in 64–77% yield. These results are notable because they show the ability of this mild radical system to accomplish the formation of other of α-aminocarbonyl classes.

We have demonstrated that this process is robust, scalable, and generally applicable for the synthesis of many heteroaryl amino acid and ketone derivatives. However, we recognize that the formation of products as racemic mixtures represents a main limitation of this method. To address this, we prepared the chiral *tert*-butyl oxazolidinone **32** that was described by Beckwith,^19^ building on early work by Karady,^20^ and Seebach.^21^ In accord with early studies, we found that heteroaryl radical addition followed by diastereoselective protonation from the less hindered *Re*-face could be achieved with a variety of halo-heteroarenes, furnished *syn*-products **33–36** with complete diasterocontrol (57–80% yield, >20 : 1 dr). Concurrent carbamate cleavage and hemiaminal hydrolysis of **36** under acidic conditions cleanly afforded the amino acid **37** with retention of stereochemical purity (98% yield, 97% ee) (Table 3).

**Table 3:** Radical conjugate addition: scope of the amino-substituted alkene coupling partner^*a*^

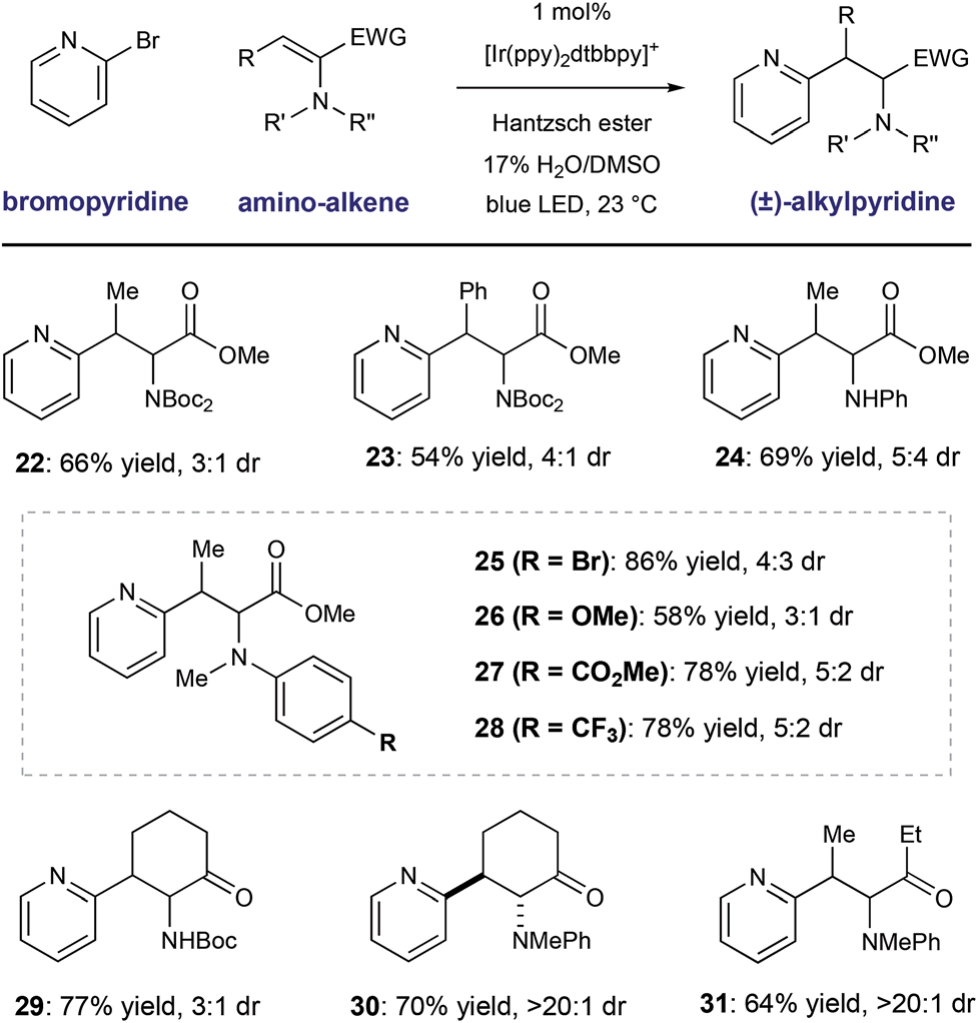

^*a*^Conditions as in Table 2, diastereomeric ratio (dr) determined by ^1^H NMR.

Other reducible radical precursors can be employed without modification of the reaction conditions to afford oxazolidinone adducts as single diastereomers. For example, the reaction of allyl bromide gives oxazolidinone **39** (42% yield). A redox-active *N*-hydroxyphthalimide ester^22^ reacted to give **39** in high yield (86% yield). Finally, reducible fluorinated alky halides operate within this manifold, affording oxazolidinone adducts **40–42** with good efficiency (60–93% yield). Deprotection of two of these products would directly yield fluorinated amino acids which have been enabling tools in a number of biomedical applications.^23^ For example, the difluorinated phosphonate l-pSer minic (deprotected **41**) is an important tool in the study of kinase-dependent signal transduction.23*a* Because chiral alkene **32** is easily accessible from cysteine (detailed in the ESI†), and both enantiomers of this starting material are commercial, this strategy would enable access to either enantiomer of the unnatural heteroaryl amino acids (Table 4).

**Table 4:** Diastereoselective RCA to Karady–Beckwith Alkene^*a*^

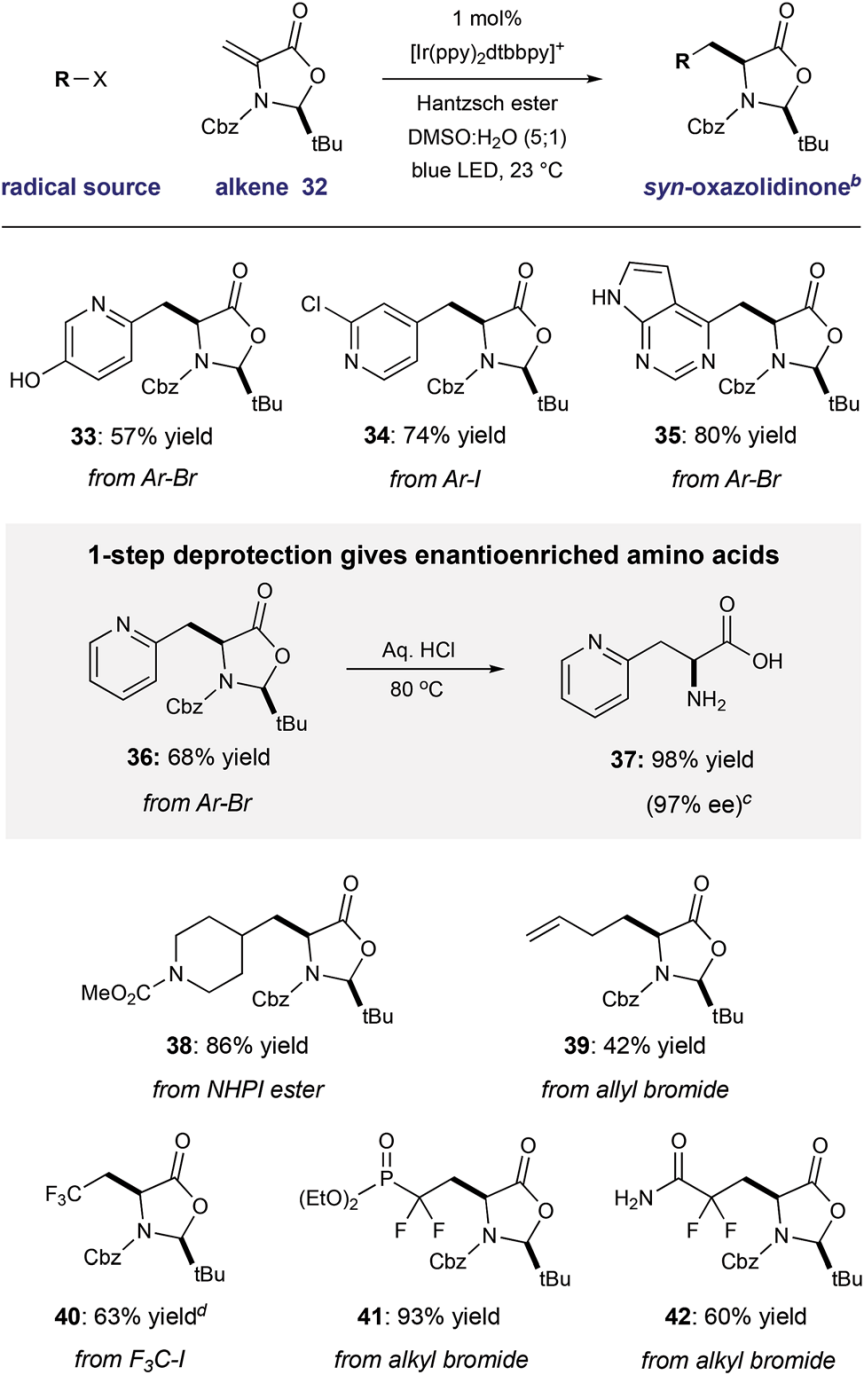

^*a*^Conditions: halogenated heteroarene (1.0 equiv.), dehydroalanine (2.0 equiv.), Ir(ppy)_2_dtbbpy·PF_6_ (1.0 mol%), Hantzsch ester (HEH, 1.3 equiv.), DMSO/H_2_O (5 : 1, 0.1 M), blue LED, 23 °C, 16 h, isolated yields shown.

^*b*^The *syn* isomer was observed with >20 : 1 selectivity in all cases.

^*c*^Enantiomeric excess (% ee) determined by chiral HPLC.

^*d*^Alkene 32 used as limiting reagent.

## Conclusions

In summary, we have described an efficient catalytic system for the preparation of unnatural α-amino acids. This protocol is effective for regiospecific generation of a broad range of heteroaryl radicals, and intermolecular coupling with dehydroamino acid derivatives and α-aminoenones. We demonstrate that this photoredox system can be conducted on large scale using near-stoichiometric conditions with good efficiency. We also show that diastereoselective radical conjugate addition to a chiral alkene is a viable strategy to access enantioenriched products, and that this process allows utilization of a range of radical precursors. The application of these findings to the synthesis of other valuable, highly complex products is a current aim of our program.

## Conflicts of interest

There are no conflicts to declare.

## Supplementary Material

Supplementary information
