# Supplementary material for: A practical and scalable system for heteroaryl amino acid synthesis
Source: Chem Sci. 2017 Oct 2;8(12):7998–8003. doi: 10.1039/c7sc03612d (PMC5863445; doi:10.1039/c7sc03612d)
Supplement: Supplementary file 1 [file SC-008-C7SC03612D-s001.pdf]

Electronic Supplementary Material (ESI) for Chemical Science.
This journal is © The Royal Society of Chemistry 2017

Mild, Practical, Scalable, Radical Synthesis of Heteroaryl Amino Acids

R. Adam Aycock, David B. Vogt, Nathan T. Jui*

Department of Chemistry and Winship Cancer Institute, Emory University, Atlanta, GA 30322

Table of Contents

I. General Information……………………………………………………S-2
I-A. General Reagent Information……………………………………S-2
I-B. General Analytical Information…………………………………S-2

II. General Procedure…………………………………………………S-3

III. Optimization Details………………………………………………S-3
III-A. Procedure for In-Text Deviation from Optimal Conditions…………S-3
III-B. Optimization Procedure …………………………………………S-3
III-C. Gas Chromatography Method Conditions…………………………S-4
III-D. Optimization Table………………………………………………S-5

IV. Preparation of Starting Materials………………………………… S-6

V. Procedure and Characterization Data………………………………S-16

VI. Deprotection Procedure and Characterization Data…………………S-34

VII. ^{1}H and ^{13}C NMR Spectra………………………………………S-37

VIII. Chiral HPLC Data…………………………………………………S-107

IX. Stern-Volmer Fluorescence Quenching Experiments……………… S-109

X. Procedure for α-Deuteration With D_2O as Solvent…………………S-111

I. General Information

I-A. General Reagent Information:

All reactions were set up on the bench top and conducted under nitrogen atmosphere while subject to irradiation from blue LEDs (LEDwholesalers PAR38 Indoor Outdoor 16-Watt LED Flood Light Bulb, Blue; or PARsource PowerPAR LED Bulb-Blue 15 Watt/440 nm, available at www.eaglelight.com). Flash chromatography was carried out using Siliaflash® P60 silica gel obtained from Silicycle. Photoredox catalyst, $[Ir(ppy)_2(dtbbpy)]PF_6$, was prepared according to a literature procedure[1]. Halogenated heteroarenes were purchased from Aldrich Chemical Co., Alfa Aesar, Acros Organics, Combi-Blocks, or Oakwood Products and were used as received. Dehydroalanines were prepared according to the designated procedures in section IV, Preparation of Dehydroalanine Substrates. Molecular sieves were activated in a commercial microwave oven then cooled under high vacuum. DMSO was purified on a Pure Process Technologies solvent purification system. Reaction solvent was prepared by combining DMSO and tap water (5:1, V:V) which was degassed in a sidearm flask under weak vacuum while subject to sonication. Alcoholic beverages used as solvents for optimization screenings were purchased from a local package store and used as received.

I-B. General Analytical Information:

All yields refer to isolated yields. New compounds were characterized by NMR, IR spectroscopy, HRMS, and melting point. NMR data were recorded on one of six spectrometers: Bruker 600 MHz, INOVA 600 MHz, INOVA 500 MHz, VNMR 400 MHz, INOVA 400 MHz, or Mercury 300 MHz. Chemical shifts (δ) are internally referenced to residual protio solvent ($CDCl_3$: δ 7.26 ppm for ^{1}H NMR and 77.23 ppm for ^{13}C NMR; C_6D_6: 7.15 ppm for ^{1}H NMR and 128.4 ppm for ^{13}C NMR; CD_3OD: δ 3.31 ppm for ^{1}H NMR and 49.1 ppm for ^{13}C NMR, or D_2O). IR spectra were obtained with a Thermo Scientific Nicolet iS10 Fourier transform infrared spectrophotometer. Mass spectrometry data were obtained from the Emory Mass Spectrometry Center using a Thermo LTQ-FTMS high resolution mass spectrometer. Melting point data was obtained with a Thomas Hoover Unimelt capillary melting point apparatus. Adduct yields for optimization data were obtained via H^1 NMR with an Inova 400 MHz NMR using 1,3,5-trimethoxybenzene as internal standard, with relaxation delay set to 5 seconds. Hydrodehalogenated yields for optimization data were obtained via gas chromatography with an Agilent Technologies 7890B Gas Chromatography system (flame-ionization detection) equipped with an Agilent Technologies 19091J-413 HP-5 column (30 m x 0.320 mm x 0.25 µm, 5% phenyl methyl siloxane) and an Agilent Technologies G4513A autoinjector. Enantioenriched samples were analyzed on a 1100 Series Agilent HPLC on Daicel Chiralcel columns (250 x 4.6 mm ID). Optical rotations were measured at 20 °C using a Perkin Elmer Model 341 Polarimeter at λ = 589 nm.

---

[1] Lowry, M. S.; Hudson, W. R.; Pascal, R. A.; Bernhard, S. *J. Am. Chem. Soc.* **2004**, *126*, 14129.

II. General Procedure:

A 20-mL screw-top test tube equipped with a stir bar was charged with Hantzsch ester (1.3 – 1.5 equiv), [Ir(ppy)$_2$(dtbbpy)]PF$_6$ (1 mol%), dehydroalanine (2 equiv), and halogenated heteroarene (1 equiv). The tube was sealed with PTFE/silicon septum and connected to a Schlenk line. The atmosphere was exchanged by applying vacuum and backfilling with N_2 (this process was conducted a total of three times). Under N_2 atmosphere, the tube was charged with degassed solvent (5:1 DMSO:H_2O, 10 mL/mmol heteroarene) by syringe. The resulting suspension was stirred under irradiation with blue LEDs for 16 hours while being cooled with a fan. The reaction was quenched with saturated sodium bicarbonate solution (60 mL) and extracted with ethyl acetate (3 x 40 mL). The extracts were combined, dried over magnesium sulfate, filtered, and concentrated by rotary evaporation. The residue was purified by flash column chromatography using the indicated solvent mixture to afford the title compound.

III. Optimization Details

III-A. Procedure for In-Text Deviation from Optimal Conditions:

A 15-mL screw-top test tube equipped with a stir bar was charged with Hantzsch ester (76 mg, 0.30 mmol, 1.5 equiv), [Ir(ppy)$_2$(dtbbpy)]PF$_6$ (1.8 mg, 0.002 mmol, 1 mol%), methyl-2-(di(*tert*-butoxycarbonyl)amino)but-2-enoate (120 mg, 0.4 mmol, 2 equiv), and 2-bromo-5-hydroxypyridine (34.8 mg, 0.20 mmol, 1 equiv). The tube was sealed with PTFE/silicon septum and connected to a Schlenk line. The atmosphere was exchanged by applying vacuum and backfilling with N_2 (this process was conducted a total of three times). Under N_2 atmosphere, the tube was charged degassed solvent (2.0 mL) by syringe. The resulting suspension was stirred under irradiation with blue LEDs for 16 hours while being cooled with a fan. The reaction was quenched with saturated sodium bicarbonate solution (10 mL) and extracted with ethyl acetate (5 x 5 mL). The extracts were combined and passed through a plug of silica which was flushed with additional ethyl acetate, and the solution was transferred to a 20-mL scintillation vial. The contents of the vial were concentrated via rotary evaporation and then subject to high vacuum for 2 hours. 1,3,5-trimethoxybenzene (33.6 mg, 1 equiv) was added, and the contents were thoroughly dissolved in $CDCl_3$. An aliquot was analyzed by H^1NMR, and the integral values were used to calculate azatyrosine ester yield.

III-B. Optimization Procedure:

A 15-mL screw-top test tube equipped with a stir bar was charged with Hantzsch ester (76 mg, 0.30 mmol, 1.5 equiv), photoredox catalyst (1 mol%), methyl-2-(di(*tert*-butoxycarbonyl)amino)but-2-enoate (120 mg, 0.4 mmol, 2 equiv), and 2-bromopyridine (31.6 mg, 0.20 mmol, 1 equiv). The tube was sealed with PTFE/silicon septum and connected to a Schlenk line. The atmosphere was exchanged by applying vacuum and backfilling with N_2 (this process was conducted a total of three times). Under N_2 atmosphere, the tube was charged degassed solvent (2.0 mL) by syringe. The resulting

suspension was stirred under irradiation with blue LEDs for 16 hours. The reaction was quenched with saturated sodium bicarbonate solution (10 mL) and extracted with ethyl acetate (5 x 5 mL). The extracts were combined and passed through a plug of silica which was flushed with additional ethyl acetate, and the solution was transferred to a 20-mL scintillation vial. An internal standard of dodecane (10 μL, 0.044 mmol) was delivered to the vial, and the contents were thoroughly mixed. A sample was analyzed by gas chromatography, and the integral values were used to calculate hydrodehalogenation product (pyridine) yield. The contents of the vial were concentrated via rotary evaporation and then subject to high vacuum for 2 hours. 1,3,5-trimethoxybenzene (33.6 mg, 1 equiv) was added, and the contents were thoroughly dissolved in $CDCl_3$. An aliquot was analyzed by H^1NMR, and the integral values were used to calculate pyridylalanine ester yield.

III-C. Gas Chromatography Method Conditions:

The gas chromatography system hardware is reported in section I-B, General Analytical Information. The injection volume for each trial is 0.5 μL. The initial oven temperature was set to 50 °C, and the ramp rate was programmed to 20 °C/min until reaching 150 °C. With no hold time, the temperature ramp rate is adjusted to 25 °C/min until reaching the maximum temperature of 325 °C. Maximum temperature is held for one minute before concluding the run.

III-D. Optimization Table:

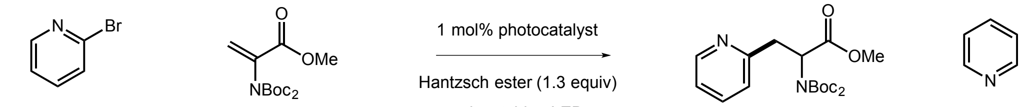

2-bromopyridine **DHA (2.0 equiv)** **(±)-A** **B**

entry	photocatalyst	solvent	deviation	% yield **A**	% yield **B**	selectivity (**A**:**B**)
1	$Ru(bpy)_3Cl_2$	MeCN (0.1 M)	-	0	0	-
2	$Ir(ppy)_3$	MeCN (0.1 M)	-	48	2	24:1
3	$Ir(dF(CF_3)ppy)_2dtbbpy{\bullet}PF_6$	MeCN (0.1 M)	-	50	2	25:1
4	$Ir(ppy)_2dtbbpy{\bullet}PF_6$	MeCN (0.1 M)	-	52	2	26:1
5	$Ru(bpy)_3Cl_2$	DMSO (0.1 M)	-	54	2	27:1
6	$Ir(ppy)_3$	DMSO (0.1 M)	-	65	2	33:1
7	$Ir(dF(CF_3)ppy)_2dtbbpy{\bullet}PF_6$	DMSO (0.1 M)	-	81	11	7:1
8	$Ir(ppy)_2dtbbpy{\bullet}PF_6$	DMSO (0.1 M)	-	81	11-	7:1
9	$Ir(ppy)_2dtbbpy{\bullet}PF_6$	DMF/H_2O (3:1, 0.1M)	-	34	2	17:1
10	$Ir(ppy)_2dtbbpy{\bullet}PF_6$	MeCN/H_2O (3:1, 0.1M)	-	27	3	9:1
11	$Ir(ppy)_2dtbbpy{\bullet}PF_6$	MeOH/H_2O (3:1, 0.1 M)	-	30	1	30:1
12	$Ir(ppy)_2dtbbpy{\bullet}PF_6$	DMSO/H_2O (3:1, 0.1 M)	-	85	3	28:1
13	$Ir(ppy)_2dtbbpy{\bullet}PF_6$	DMSO/H_2O (3:1, 0.03 M)	-	98	2	49:1
14	$Ir(ppy)_2dtbbpy{\bullet}PF_6$	DMSO/H_2O (5:1, 0.1 M)	-	98	2	49:1
15	$Ir(ppy)_2dtbbpy{\bullet}PF_6$	DMSO/H_2O (5:1, 0.1 M)	1.1 equiv DHA	93	7	13:1
16	$Ir(ppy)_2dtbbpy{\bullet}PF_6$	DMSO/H_2O (5:1, 0.1 M)	air-exposed	90	5	18:1
17	$Ir(ppy)_2dtbbpy{\bullet}PF_6$	DMSO/H_2O (5:1, 0.1 M)	1.1 equiv DHA, air-exposed	85	7	12:1
18	$Ir(ppy)_2dtbbpy{\bullet}PF_6$	DMSO/H_2O (5:1, 0.1 M)	no Hantzsch ester	0	0	-
19	$Ir(ppy)_2dtbbpy{\bullet}PF_6$	DMSO/H_2O (5:1, 0.1 M)	no light	0	0	-
20	none	DMSO/H_2O (5:1, 0.1 M)	-	0	0	-
21	$Ir(ppy)_2dtbbpy{\bullet}PF_6$	Grey Goose vodka (0.3 M)	-	85	12	7:1
22	$Ir(ppy)_2dtbbpy{\bullet}PF_6$	Bacardi white rum (0.3 M)	-	86	12	7:1
23	$Ir(ppy)_2dtbbpy{\bullet}PF_6$	Johnnie Walker Scotch (0.3 M)	-	84	12	7:1
24	$Ir(ppy)_2dtbbpy{\bullet}PF_6$	Woodford Reserve Bourbon (0.3 M)	-	93	4	23:1
25	$Ir(ppy)_2dtbbpy{\bullet}PF_6$	Seagram's gin (0.3 M)	-	94	4	24:1
26	$Ir(ppy)_2dtbbpy{\bullet}PF_6$	Redbull/Grey Goose (1:2, 0.3 M)	-	42	1	42:1

IV. Preparation of Starting Materials:

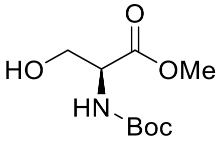

methyl (*tert*-butoxycarbonyl)-L-serinate:

To a stirring solution of L-serine, methyl ester hydrochloride (20.0g, 128 mmol, 1 equiv) in dichloromethane (130 mL) at 0 °C was added triethylamine (40 mL, 282 mmol, 2.2 equiv) and di-*tert*-butyl dicarbonate (37 mL, 135 mmol, 1.1 equiv). After stirring for 30 minutes, the solution was warmed to room temperature, and stirred for an additional 18 hours. The reaction mixture was concentrated by rotary evaporation, diluted with ethyl acetate, and washed with 1M HCl, saturated aqueous $NaHCO_3$, and brine. The organic phase was dried over sodium sulfate, filtered, and concentrated by rotary evaporation. The reside was passed through a silica plug (50% ethyl acetate in hexanes) to afford the product as a clear, colorless oil (37.2 g 94% yield). The physical properties and spectral data are consistent with the reported values.[2]

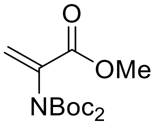

methyl-2-(di(*tert*-butoxycarbonyl)amino)but-2-enoate:

To a stirring solution of methyl (*tert*-butoxycarbonyl)-L-serinate (37.2, 120 mmol, 1.0 equiv) in acetonitrile (200 mL) at 0 °C was added di-*tert*-butyl dicarbonate (58.3 mL, 281 mmol, 2.2 equiv) and 4-dimethylaminopyridine (3. 12 g, 25.6 mmol, 0.20 equiv). The resulting solution was warmed to room temperature and stirred for 8 hours. DBU (2.00 g, 12.8 mmol, 0.10 equiv) was added, and the resulting mixture was stirred for an additional 8 hours. The reaction was concentrated by rotary evaporation then diluted in ethyl acetate. The mixture was washed with 1M HCl and saturated aqueous $NaHCO_3$, dried over sodium sulfate, filtered, and concentrated by rotary evaporation. The residue was purified by passing through a short plug of silica (5% – 15% ethyl acetate/hexanes) to afford the product (31.5 g, 89% yield) as a white solid. The physical properties and spectral data are consistent with the reported values.[3]

---

[2] Hiroaki, T. and Hisashi, Y. *J. Am. Chem. Soc.* **2016**, *138*, 14218.

[3] Adams, L. A.; Aggarwal, V. K.; Bonnert, R. V.; Bressel, B.; Cox, R. J.; Shepherd, J.; de Vicente, J.; Walter, M.; Whittingham, W. G.; and Winn, C. L. *J. Org. Chem.* **2003**, *68*, 9433.

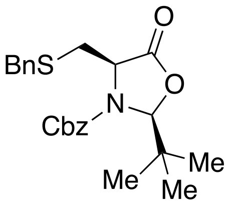

benzyl (2*S*,4*R*)-4-((benzylthio)methyl)-2-(*tert*-butyl)-5-oxooxazolidine-3-carboxylate: To a round bottom flask equipped with a stir bar was added *S*-benzyl-L-cysteine (10 g, 47 mmol, 1 equiv.), NaOH (1.8 g, 45 mmol, 0.95 equiv), and anhydrous MeOH (500 mL). The reaction was stirred at room temperature for 30 minutes or until nearly homogenous. Pivaldehyde (4.9 g, 57 mmol, 1.2 equiv) and activated 3 Å molecular sieves (50 g) were added to the reaction flask, each in one portion. The reaction was placed under nitrogen atmosphere and stirred at room temperature until the starting material had been consumed (determined by ^{1}H NMR of a filtered and concentrated aliquot of the reaction solution dissolved in D_3COD). The reaction was quickly filtered through celite and concentrated by rotary evaporation. The residue was dried under high vacuum for 4 hours to afford the imine as a white solid. The imine was dissolved in anhydrous DCM (500 mL) and cooled to 0 °C in an oversized, well-insulated ice bath. Benzyl chloroformate (10.1 mL, 71 mmol, 1.5 equiv) was added to the cooled reaction dropwise via syringe. The reaction was stirred at 0 °C for a full 18 hours then warmed to room temperature and stirred for an additional 6 hours. The mixture was washed with 1 M aqueous NaOH (1 x 250 mL). The organic layer was dried over sodium sulfate, filtered, and concentrated by rotary evaporation. The residue was purified by flash chromatography (5% – 15% ethyl acetate/hexanes) to afford the product (8.2 g, 42% yield) as a colorless oil.

^{1}H NMR (300 MHz, $CDCl_3$) δ 7.43 – 7.34 (m, 5H), 7.33 – 7.20 (m, 5H), 5.55 (s, 2H), 5.21 (dd, *J* = 16.6, 12.1 Hz, 2H), 4.55 (dd, *J* = 7.8, 6.2 Hz, 1H), 3.78 (q, *J* = 13.4 Hz, 1H), 2.94 (dd, *J* = 13.9, 8.0 Hz, 1H), 2.79 (dd, *J* = 13.9, 6.1 Hz, 1H) 0.93 (s, 9H).
^{13}C NMR (75 MHz, $CDCl_3$) δ 171.1, 155.6, 137.6, 135.0, 128.9, 128.5, 128.4, 128.3, 127.0, 96.1, 68.3, 57.4, 36.7, 36.3, 33.1, 24.7.
FTIR (neat) ν_{max}: 33063, 3031, 2970, 1791, 1717, 1481, 1454, 1390, 1344, 1324, 1221, 1196, 1170, 1118, 1036, 1016, 968, 908, 728, and 697 cm^{-1}.
HRMS (NSI) *m/z*: $[M+H]^+$ calcd. for $C_{23}H_{28}O_4NS$, 414.1733; found, 414.1731.

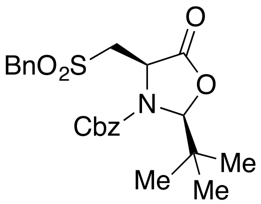

benzyl (2*S*,4*R*)-4-((benzylsulfonyl)methyl)-2-(*tert*-butyl)-5-oxooxazolidine-3-carboxy-late:

To a round bottom flask equipped with a stir bar was added benzyl (2*S*,4*R*)-4-((benzylthio)methyl)-2-(*tert*-butyl)-5-oxooxazolidine-3-carboxylate (2.4 g, 6 mmol, 1 equiv), *meta*-chloroperoxybenzoic acid (2.5 g, 15 mmol, 2.5 equiv), and DCM (200 mL). The reaction was stirred at room temperature for 18 hours. The reaction mixture was washed with 1 M aqueous sodium hydroxide (3 x 100 mL). The organic layer was dried

over sodium sulfate, filtered, and concentrated by rotary evaporation. The residue was purified by flash chromatography (5% – 30% ethyl acetate/hexanes) to afford the product (2.5 g, 95% yield) as a white foam.

^{1}H NMR (400 MHz, $CDCl_3$) δ 7.47 – 7.22 (m, 10H), 5.60 (s, 1H), 5.30 – 5.14 (m, 2H), 5.07 (dd, *J* = 7.9, 4.0 Hz, 1H), 4.63 (d, *J* = 14.0 Hz, 1H), 4.40 (d, *J* = 14.0 Hz, 1H), 3.42 (dd, *J* = 15.3, 7.9 Hz, 1H), 3.15 (dd, *J* = 15.3, 4.0 Hz, 1H), 0.87 (s, 9H).
^{13}C NMR (100 MHz, $CDCl_3$) δ 170.7, 155.3, 134.9, 129.0, 128.9, 128.7, 128.7, 128.7, 127.9, 96.8, 68.8, 60.2, 53.5, 52.6, 37.0, 24.5.
FTIR (neat) ν_{max}: 3066, 3034, 2972, 2874, 2256, 1791, 1719, 1456, 1392, 1312, 1285, 1119, 1039, 966, 908, 725, and 696 cm^{-1}.
HRMS (NSI) *m/z*: $[M+H]^+$ calcd. for $C_{24}H_{24}O_2N_5S$, 446.1645; found, 446.1640.

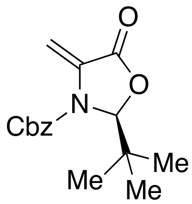

benzyl (*S*)-2-(*tert*-butyl)-4-methylene-5-oxooxazolidine-3-carboxylate (22):
To a round bottom flask equipped with a stir bar was added (benzyl (2*S*,4*R*)-4-((benzylsulfonyl)methyl)-2-(*tert*-butyl)-5-oxooxazolidine-3-carboxylate) (3.6g, 8 mmol, 1 equiv), and DCM (100 mL). The flask was chilled to 0 °C in an ice bath, and DBU (1.3 mL, 9 mmol, 1.1 equiv) was added dropwise via syringe. The reaction was stirred at 0 °C until the starting material had been consumed (determined by TLC, about 10 minutes). While still at 0 °C, the reaction mixture was quenched with saturated aqueous ammonium chloride (50 mL), the layers were separated, and the organic phase was washed with saturated aqueous ammonium chloride (3 x 100 mL). The organic layer was dried over sodium sulfate, filtered, and concentrated by rotary evaporation. The residue was purified by flash chromatography (5% – 15% ethyl acetate/ hexanes) to afford the product (2.0 g, 87% yield) as a colorless oil. The physical properties and spectral data are consistent with the reported values[4]. Chiral HPLC analysis of the alkene (OJ-H, 5% IPA/hexanes, 1.0 mL/min, 254 nm) indicated 97% ee for the major enantiomer (t_R (minor) = 11.800 min, t_R (major) = 13.225 min).

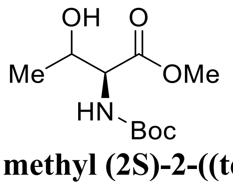

methyl (2S)-2-((tert-butoxycarbonyl)amino)-3-hydroxybutanoate:
To a stirring solution of L-threonine, methyl ester hydrochloride (8.2 g, 49 mmol, 1.0 equiv) in dichloromethane (80 mL) at 0 °C was added triethylamine (21 mL, 150 mmol, 3.0 equiv) and di-*tert*-butyl dicarbonate (12 g, 53 mmol, 1.1 equiv). After stirring 30 minutes, the solution was warmed to room temperature, and stirring was continued for an

[4] Hargrave, J. D.; Bish, D.; Köhn, G. K.; and Frost, C. G *Org. Biomol. Chem.*, **2010**, *8*, 5120.

additional 18 hours. The reaction mixture was concentrated by rotary evaporation, diluted with ethyl acetate, and washed with 1M HCl, saturated aqueous $NaHCO_3$, and brine. The organic phase was dried over sodium sulfate, filtered, and concentrated by rotary evaporation. The residue was passed through a silica plug to afford the product (10.7 g, 95% yield) as a clear, colorless oil. The physical properties and spectral data are consistent with the reported values.[2]

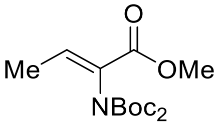

methyl-2-(di(tert-butoxycarbonyl)amino)but-2-enoate:
To a stirring solution of methyl (2S)-2-((tert-butoxycarbonyl)amino)-3-hydroxybutanoate (10.0 g, 42.9 mmol, 1.0 equiv) in acetonitrile (120 mL) at 0 °C was added di-*tert*-butyl dicarbonate (19.6 g, 90.1 mmol, 2.1 equiv) and DMAP (510 mg, 4.2 mmol, 0.10 equiv). The resulting solution was warmed to room temperature, and after stirring for 8 hours DBU (1.31 g, 8.59 mmol, 0.20 equiv) was added, and the resulting mixture was stirred for 8 hours. The reaction was concentrated by rotary evaporation then diluted in ethyl acetate. The mixture was washed with 1M HCl and saturated aqueous $NaHCO_3$, dried over sodium sulfate, filtered, and concentrated by rotary evaporation. The residue was purified by passing through a short pad of silica (hexane/ethyl acetate = 30%) to afford the product (9.59 g , 71% yield) as a clear, colorless oil.

^{1}H NMR (300 MHz, $CDCl_3$) δ 6.93 – 6.68 (m, 1H), 3.72 (s, 3H), 1.72 (d, J = 7.1 Hz, 2H), 1.41 (d, J = 1.0 Hz, 18H).
^{13}C NMR (75 MHz, $CDCl_3$) δ 164.3, 150.4, 136.5, 130.1, 82.7, 52.0, 27.8, 13.3.
FTIR (neat) ν_{max}: 2980, 2953, 2935, 1792, 1757, 1727, 1368, 1270, 1250, 1152, 1093, 1044, and 730 cm^{-1}.
HRMS (NSI) *m/z*: $[M+H]^+$ calcd. for $C_{15}H_{26}NO_6$, 316.1755; found, 316.1756.

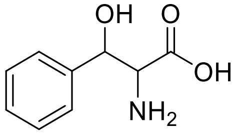

2-amino-3-hydroxy-3-phenylpropanoic acid:
To a stirring solution of NaOH (10 g, 250 mmol, 4.5 equiv) in water (50 mL) was added glycine (5.7 mL, 56 mmol, 1.0 equiv). The solution was stirred 10 minutes, then benzaldehyde (10 g, 151 mmol, 2.9 equiv) was added. The solution was stirred for an additional 30 minutes as an off-white emulsion formed. The precipitant was broken apart in the flask, and concentrated HCl (aq) (130 mL) was added slowly while stirring until consumption of the solid was observed to give a clear yellow solution. After stirring an additional 10 minutes, a beige precipitate formed. The reaction mixture was cooled to 0 °C, and the precipitate was collected by vacuum filtration and washed with ether. The solid

was dried under high vacuum to give the product as an off-white solid (11.6 g, 72% yield). The physical properties and spectral data are consistent with the reported values.[5]

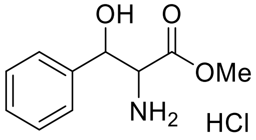

methyl (2S)-2-amino-3-hydroxy-3-phenylpropanoate:
To a stirring solution of 3-hydroxyphenylalanine (3.62 g, 20 mmol, 1.0 equiv) in methanol (80 mL) at 0 °C was added thionyl chloride (3.5 g, 30 mmol, 1.5 equiv) dropwise via syringe, and the reaction mixture was stirred for 30 minutes while gradually warming to room temperature. Upon reaching room temperature, a reflux condenser was attached, and the reaction mixture was heated to 65 °C and stirred under reflux for an additional 5 hours. After cooling to room temperature, the reaction mixture was concentrated by rotary evaporation, diluted with chloroform, concentrated by rotary evaporation, washed with ether, and dried under high vacuum for 2 hours to afford the product as a white solid (4.6 g, 99% yield). The physical properties and spectral data are consistent with the reported values.[6]

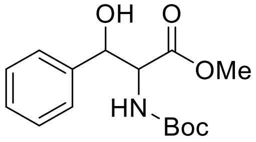

phenylalanine, *N*-[(1,1-dimethylethoxy)carbonyl]-β-hydroxy-, methyl ester:
To a stirring solution of methyl (2S)-2-amino-3-hydroxy-3-phenylpropanoate hydrochloride (5.8 g, 25 mmol, 1.0 equiv) in dichloromethane (70 mL) at 0 °C was added triethylamine (7.6 mL, 57 mmol, 2.5 equiv) and di-*tert*-butyl dicarbonate (5.5 mL, 25 mmol, 1.0 equiv). After stirring 30 minutes, the solution was warmed to room temperature, and stirred for an additional 18 hours. The reaction mixture was concentrated by rotary evaporation, diluted with ethyl acetate, and washed with 1M HCl, saturated aqueous $NaHCO_3$, and brine. The organic phase was dried over sodium sulfate, filtered, and concentrated by rotary evaporation. The reside was passed through a silica plug (50% ethyl acetate/hexanes) to afford the product as a clear, colorless oil (5.10 g 91% yield). The physical properties and spectral data are consistent with the reported values.[7]

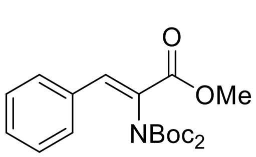

methyl 2-(di(tert-butoxycarbonyl)amino)-3-phenylacrylate:
To a stirring solution of phenylalanine, *N*-[(1,1-dimethylethoxy)carbonyl]-β-hydroxy-, methyl ester (5.01 g, 16.9 mmol, 1.0 equiv) in acetonitrile (22 mL) at 0 °C was added di-

---

[5] Shiraiwa, T.; Saijoh, R.; Suzuki, M.; Yoshida, K.; Nishimura, S.; Nagasawa, H. *Chem. Pharm. Bull.* **2003**, *51*, 1363.
[6] Miyata, O.; Asai, H.; Naito, T. *Chem. Pharm. Bull.* **2005**, *53*, 355.
[7] Bengtsson, C.; Nelander, H.; Almqvist, F. *Chem. Eur. J.*, **2013**, *19*, 9916.

tert-butyl dicarbonate (8.10 g, 37.2 mmol, 2.2 equiv) and DMAP (206 mg, 1.69 mmol, 0.10 equiv). The resulting solution was warmed to room temperature, and after stirring for 8 hours DBU (516 mg, 3.4 mmol, 0.2 equiv) was added, and the resulting mixture was allowed to continue stirring for an additional 8 hours. The reaction was concentrated by rotary evaporation then diluted with ethyl acetate. The organic layer was washed with 1M HCl and saturated aqueous $NaHCO_3$, dried over sodium sulfate, filtered, and concentrated by rotary evaporation. The residue was purified by flash column chromatography (5% – 15% ethyl acetate/hexanes) to afford the product, (5.17 g, 81% yield) as a clear, colorless oil.

^{1}H NMR (300 MHz, $CDCl_3$) δ 7.52 (s, 1H), 7.50 – 7.45 (m, 2H), 7.40 – 7.35 (m, 3H), 3.83 (s, 3H), 1.30 (s, 18H).
^{13}C NMR (125 MHz, $CDCl_3$) δ 165.4, 150.0, 135.7, 133.0, 129.9, 129.6, 128.9, 127.1, 82.9, 52.4, 27.6.
FTIR (neat) ν_{max}: 2979, 2952, 2934, 1794, 1752, 1722, 1393, 1317, 1248, 1149, 1113, 1093, 1027, 850, and 780 cm^{-1}.
HRMS (NSI) *m/z*: $[M+H]^+$ calcd. for $C_{21}H_{24}N_2O_2$, 378.1925; found, 378.1919.

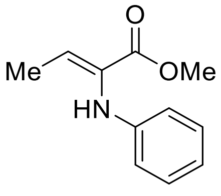

methyl 2-(phenylamino)but-2-enoate:
To a round-bottom flask equipped with stir bar was added methyl 2-oxobutanoate (6.1 g, 52 mmol, 1.0 equiv), aniline (4.8 g, 52 mmol, 1.0 equiv), *p*-toluenesulfonic acid monohydrate (494 mg, 2.6 mmol, 0.05 equiv), and benzene (150 mL). A Dean-Stark apparatus and reflux condenser were attached, and the mixture was heated to 95 °C while stirring for 24 hours. The reaction mixture was concentrated by rotary evaporation, and the residue was purified by flash column chromatography (5% – 50% ethyl acetate/hexanes) to afford the product (6.0 g, 59% yield) as an orange oil.

^{1}H NMR (300 MHz, $CDCl_3$) δ 7.30 – 7.19 (m, 2H), 6.86 (t, J = 7.5 Hz, 1H), 6.78 – 6.43 (m, 3H), 5.64 (s, 1H), 3.79 (s, 3H), 1.73 (d, J = 7.3 Hz, 2H).
^{13}C NMR (75 MHz, $CDCl_3$) δ 166.3, 144.2, 130.3, 129.0, 119.4, 115.3, 52.2, 14.5.
FTIR (neat) ν_{max}: 3375, 3053, 3026, 2971, 2951, 1708, 1647, 1599, 1497, 1434, 1266, 1244, 1175, 747, and 693 cm^{-1}.
HRMS (NSI) m/z: [M+H]+ calcd. for $C_{11}H_{14}O_2N$, 192.1019; found, 192.1019.

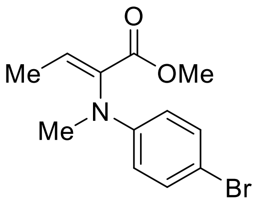

methyl 2-((4-bromophenyl)(methyl)amino)but-2-enoate:
To a round-bottom flask equipped with stir bar was added methyl 2-oxobutanoate (2.3 g, 20 mmol, 2.0 equiv), 4-bromo-*N*-methylaniline (1.1 g, 10 mmol, 1.0 equiv), *p*-toluenesulfonic acid monohydrate (95 mg, 0.50 mmol, 0.05 equiv), and benzene (50 mL). A Dean-Stark apparatus and reflux condenser were attached, and the mixture was heated to 95 °C while stirring for 24 hours. The reaction mixture was concentrated by rotary evaporation, and the residue was purified by flash column chromatography (5% – 30% ethyl acetate/hexanes) to afford the product (1.6 g, 83% yield) as a clear, colorless oil.

^{1}H NMR (500 MHz, $CDCl_3$) δ 7.41 – 7.18 (m, 2H), 6.99 (q, J = 7.0 Hz, 1H), 6.49 (d, J = 9.0 Hz, 2H), 3.68 (d, J = 0.9 Hz, 3H), 3.04 (s, 3H), 1.81 – 1.70 (m, 3H).
^{13}C NMR (125 MHz, $CDCl_3$) δ 165.6, 146.9, 138.2, 136.7, 131.7, 113.9, 109.5, 51.9, 38.0, 13.5.
FTIR (neat) ν_{max}: 2972, 2950, 2819, 1732, 1589, 1498, 1434, 1371, 1303, 1239, 1206, 808, and 747cm^{-1}.
HRMS (NSI) m/z: [M+H]+ calcd. for $C_{12}H_{15}O_2NBr$, 284.0281; found, 284.0285.

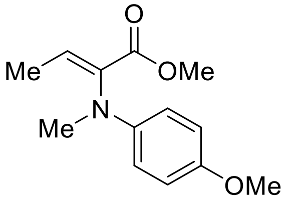

methyl 2-((4-methoxyphenyl)(methyl)amino)but-2-enoate:
To a round-bottom flask equipped with stir bar was added methyl 2-oxobutanoate (2.3 g, 20 mmol, 2.0 equiv), 4-methoxy-*N*-methylaniline (1.4 g, 10 mmol, 1.0 equiv), *p*-toluenesulfonic acid (95 mg, 0.50 mmol, 0.05equiv), and benzene (50 mL). A Dean-Stark apparatus and reflux condenser were attached, and the mixture was heated to 95 ^{o}C while stirring for 24 hours. The reaction mixture was concentrated by rotary evaporation, and the residue was purified by flash column chromatography (5% – 40% ethyl acetate/hexanes) to afford the product (1.6 g, 68% yield) as a clear, colorless oil.

^{1}H NMR (500 MHz, $CDCl_3$) δ 6.91 (q, J = 7.1 Hz, 1H), 6.81 (d, J = 9.0 Hz, 2H), 6.59 (d, J = 9.1 Hz, 2H), 3.75 (s, 3H), 3.67 (s, 3H), 3.05 (s, 3H), 1.79 (d, J = 7.1 Hz, 3H).
^{13}C NMR (125 MHz, $CDCl_3$) δ 166.2, 151.9, 142.4, 137.6, 136.9, 114.7, 113.3, 55.6, 51.7, 38.3, 13.4.
FTIR (neat) ν_{max}: 2992, 2948, 2906, 2832, 1718, 1647, 1507, 1238, 1201, 1123, 1114, 1036, and 817 cm^{-1}.
HRMS (NSI) m/z: [M+H]+ calcd. for $C_{13}H_{18}O_3N$, 236.1281; found, 236.1278.

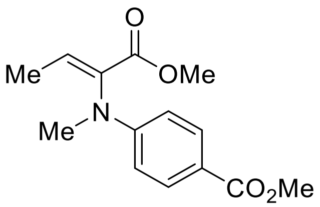

methyl 4-((1-methoxy-1-oxobut-2-en-2-yl)(methyl)amino)benzoate:

To a round-bottom flask equipped with stir bar was added methyl 2-oxobutanoate (2.3 g, 20 mmol, 1.0 equiv), methyl 4-(methylamino)benzoate (3.3 g, 20 mmol, 1.0 equiv), *p*-toluenesulfonic acid (190 mg, 1.0 mmol, 0.05 equiv), and benzene (50 mL). A Dean-Stark apparatus and reflux condenser were attached, and the mixture was heated to 95 °C while stirring for 24 hours. The reaction mixture was concentrated by rotary evaporation, and the residue was purified by flash column chromatography (5% – 50% ethyl acetate/hexanes) to afford the product (4.1 g, 77% yield) as a clear, colorless oil.

^{1}H NMR (500 MHz, $CDCl_3$) δ 7.87 (d, J = 8.5 Hz, 2H), 7.02 (q, J = 6.8 Hz, 1H), 6.57 (d, J = 8.5 Hz, 2H), 3.81 (s, 4H), 3.66 (s, 3H), 3.08 (s, 3H), 1.72 (d, J = 7.1 Hz, 3H).
^{13}C NMR (125 MHz, $CDCl_3$) δ 167.1, 165.2, 151.4, 138.7, 136.2, 131.2, 118.6, 111.2, 52.0, 51.4, 38.0, 13.5.
FTIR (neat) ν_{max}: 2990, 2949, 2907, 1705, 1601, 1516, 1433, 1275, 1255, 1177, 1108, 1042, and 768 cm^{-1}.
HRMS (NSI) m/z: [M+H]+ calcd. for $C_{14}H_{18}O_4N$, 264.1230; found, 264.1227.

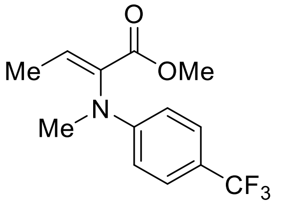

methyl 2-(methyl(4-(trifluoromethyl)phenyl)amino)but-2-enoate:

To a round-bottom flask equipped with stir bar was added methyl 2-oxobutanoate (2.3 g, 20 mmol, 2.0 equiv), 4-trifluoromethyl-*N*-methylaniline (1.8 g, 10 mmol, 1.0 equiv), *p*-toluenesulfonic acid monohydrate (95 mg, 0.5 mmol, 0.05 equiv), and benzene (50 mL). A Dean-Stark apparatus and reflux condenser were attached, and the mixture was heated to 95 °C while stirring for 24 hours. The reaction mixture was concentrated by rotary evaporation, and the residue was purified by flash column chromatography (5% – 40% ethyl acetate/hexanes) to afford the product (2.3 g, 84% yield) as a clear, colorless oil.

^{1}H NMR (500 MHz, $CDCl_3$) δ 7.44 (d, J = 9.1 Hz, 1H), 7.07 (q, J = 7.0 Hz, 0H), 6.65 (d, J = 8.9 Hz, 1H), 3.71 (s, 1H), 3.10 (s, 1H), 1.76 (d, J = 7.1 Hz, 1H).
^{13}C NMR (75 MHz, $CDCl_3$) δ 165.3, 150.2, 139.0, 136.2, 126.5 (q, J = 4.2 Hz), 125.0 (q, J = 270.0 Hz), 118.7 (q, J = 33.1 Hz), 111.5, 52.0, 38.0, 13.4.
^{19}F NMR (282 MHz, $CDCl_3$) δ -61.01.
FTIR (neat) ν_{max}: 2994, 2953, 2912, 2825, 1720, 1650, 1613, 1524, 1321, 1257, 1205, 1193, 1102, 1067, 1043, 849, and 576 cm^{-1}.
HRMS (NSI) m/z: [M+H]+ calcd. for $C_{13}H_{15}O_2NF_3$, 274.1049; found, 274.1050.

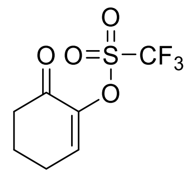

6-oxocyclohex-1-en-1-yl trifluoromethanesulfonate:
To a stirring solution of 1,2-cyclohexanedione (5.0 g, 45 mmol, 1.0 equiv) in dichlormethane (100 mL) at -78 °C was added triethylamine (5.5 g, 54 mmol, 1.2 equiv) and trifluoromethanesulfonic anhydride (12.7 g, 45 mmol, 1.0 equiv). The resulting solution was warmed to room temperature and stirred for an additional 3 hours. The reaction was concentrated by rotary evaporation then diluted with ethyl acetate. The organic layer was washed with 1M HCl and saturated aqueous sodium bicarbonate, dried over sodium sulfate, filtered, and concentrated by rotary evaporation. The residue was purified by flash column chromatography (10% – 30% ethyl acetate/hexanes) to afford the product (6.2 g, 57% yield) as a white crystalline, low-melting solid.

^{1}H NMR (600 MHz, $CDCl_3$) δ 6.93 (t, J = 4.4 Hz, 1H), 2.84 – 2.34 (m, 4H), 2.07 (p, J = 7.4, 6.9, 5.6, 5.6 Hz, 2H).
^{13}C NMR (150 MHz, $CDCl_3$) δ 189.8, 144.5, 139.5, 118.5 (q, J = 320.1 Hz), 37.6, 24.9, 21.8.
^{19}F NMR (282 MHz, $CDCl_3$) δ -73.99.
FTIR (neat) ν_{max}: 2953, 1702, 1419, 1349, 1202, 1137, 1070, 914, and 809 cm^{-1}.
HRMS (NSI) *m/z*: $[M+H]^+$ calcd. for $C_7H_8O_4S$, 245.0090; found, 245.0087.

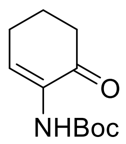

tert-butyl (6-oxocyclohex-1-en-1-yl)carbamate:
A three-neck round-bottom flask equipped with a stir bar was charged with 6-oxocyclohex-1-en-1-yl trifluoromethanesulfonate (4.0 g, 16.3 mmol, 1.0 equiv), tert-butyl carbamate (2.2 g, 19.6 mmol, 1.2 equiv, $Pd_2(dba)_3$ (372 mg, 0.41 mmol, 0.025 equiv), 2-di-tert-butylphosphino-2′,4′,6′-triisopropylbiphenyl (691 mg, 1.6 mmol, 0.10 equiv), and K_2CO_3 (5.5 g, 40.8 mmol, 2.5 equiv). A reflux condenser was connected, and each inlet was sealed with a rubber septum. The atmosphere was exchanged by applying vacuum and backfilling with N_2 (this process was conducted a total of three times). Under N_2 atmosphere, the tube was charged with degassed toluene (40 mL). The reaction mixture was heated to 80 °C and stirred under N_2 for 12 hours. After cooling to room temperature, the reaction was quenched with saturated ammonium chloride solution and extracted with dichloromethane (3 x 100 mL). The combined organic layers were dried over sodium sulfate, filtered, and concentrated by rotary evaporation. The residue was purified by flash column chromatography (10% – 70% ethyl acetate/hexanes) to afford the product (2.8 g, 80% yield) as a pale yellow oil.

^{1}H NMR (400 MHz, $CDCl_3$) δ 7.29 (t, J = 4.7 Hz, 1H), 7.05 (s, 1H), 2.56 – 2.28 (m, 4H), 2.00 – 1.76 (m, 2H), 1.39 (s, 9H).
^{13}C NMR (75 MHz, $CDCl_3$) 193.7, 152.8, 132.4, 127.2, 80.1, 37.0, 28.1, 24.5, 22.4.

FTIR (neat) ν_{max}: 3402, 2977, 2933, 2871, 2832, 1784, 1721, 1672, 1638, 1507, 1355, 1227, 1151, 1042, 1020, 877, and 867 cm^{-1}.
HRMS (NSI) m/z: [M+H]+ calcd. for $C_{11}H_{18}O_3N$, 212.1281; found, 212.1279.

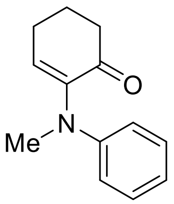

2-(methyl(phenyl)amino)cyclohex-2-en-1-one:
To a round-bottom flask equipped with stir bar was added cyclohexane-1,2-dione (2.2 g, 20 mmol, 2.0 equiv), *N*-methylaniline (1.1 g, 10 mmol, 1.0 equiv), *p*-toluenesulfonic acid (95 mg, 0.5 mmol, 0.05 equiv), and benzene (50 mL). A Dean-Stark apparatus and reflux condenser were attached, and the mixture was heated to 95 °C while stirring for 24 hours. The reaction mixture was diluted with ethyl acetate, washed with water (2 x 50 mL), dried over sodium sulfate, filtered, and concentrated by rotary evaporation. The residue was purified by flash column chromatography (5% – 30% ethyl acetate/hexanes) to afford the product (1.5 g, 80% yield) as a yellow oil.

^{1}H NMR (300 MHz, $CDCl_3$) δ 7.19 (dd, J = 8.7, 7.2 Hz, 2H), 6.83 – 6.76 (m, 2H), 6.75 – 6.68 (m, 2H), 3.07 (s, 3H), 2.54 (qd, J = 6.3, 3.8 Hz, 4H), 2.09 (p, J = 6.2 Hz, 2H).
^{13}C NMR (75 MHz, $CDCl_3$) δ 196.4, 149.0, 144.6, 143.1, 128.8, 118.4, 114.7, 39.5, 39.3, 26.0, 22.9.
FTIR (neat) ν_{max}: 3058, 3024, 2942, 2874, 2813, 1680, 1596, 1497, 1323, 1128, 747, and 691 cm^{-1}.
HRMS (NSI) m/z: [M+H]+ calcd. for $C_{13}H_{16}ON$, 202.1226; found, 202.1225.

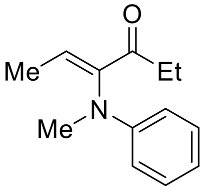

4-(methyl(phenyl)amino)hex-4-en-3-one:
To a round-bottom flask equipped with stir bar was added hexane-3,4-dione (5.5 g, 50 mmol, 5.0 equiv), *N*-methylaniline (1.1 g, 10 mmol, 1.0 equiv), *p*-toluenesulfonic acid (95 mg, 0.5 mmol, 0.05 equiv), and benzene (50 mL). A Dean-Stark apparatus and reflux condenser were attached, and the mixture was heated to 95 °C while stirring for 18 hours. The reaction mixture was diluted with ethyl acetate, washed with water (2 x 50 mL), dried over sodium sulfate, filtered, and concentrated by rotary evaporation. The residue was purified by flash column chromatography (5% – 30% ethyl acetate/hexanes) to afford the product (1.8 g, 91% yield) as a yellow oil.

^{1}H NMR (300 MHz, $CDCl_3$) δ 7.28 – 7.16 (m, 2H), 6.88 – 6.80 (m, 1H), 6.75 (tt, J = 7.3, 1.2 Hz, 1H), 6.60 (dd, J = 7.7, 1.1 Hz, 2H), 3.13 (s, 3H), 2.44 (q, J = 7.1 Hz, 2H), 1.75 (d, J = 7.1 Hz, 3H), 1.01 (t, J = 7.2 Hz, 3H).

^{13}C NMR (75 MHz, $CDCl_3$) δ 201.5, 147.5, 145.0, 135.0, 129.3, 117.3, 111.9, 38.1, 31.8, 13.6, 7.9.
FTIR (neat) ν_{max}: 3060, 2972, 2935, 2917, 1713, 1593, 1503, 1360, 746, and 691 cm^{-1}.
HRMS (NSI) m/z: [M+H]+ calcd. for $C_{13}H_{18}ON$, 204.1383; found, 204.1381.

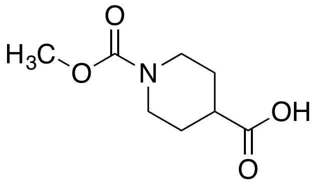

1-(methoxycarbonyl)piperidine-4-carboxylic acid:
To a round bottom flask equipped with a stir bar was added piperidine-4-carboxylic acid (5.0 g, 39 mmol, 1 equiv), THF (100 mL), and saturated aqueous sodium bicarbonate (100 mL). Methyl chloroformate (6.0 mL, 77.0 mmol, 2 equiv) was then added dropwise via syringe. The reaction was allowed to stir at room temperature overnight. The reaction mixture was filtered over celite then concentrated to remove THF. The remaining solution was acidified to pH = 2 using 1 M HCl then extracted with ethyl acetate (3 x 100 mL). The combined extracts were dried over sodium sulfate, filtered, then concentrated by rotary evaporation. The residue was purified by flash chromatography (5% – 40% ethyl acetate/hexanes) to afford the product (5.65 g, 78% yield) as a white solid.

^{1}H NMR (500 MHz, $CDCl_3$) δ 11.46 (s, 1H), 4.01 (m, 2H), 3.65 (s, 3H), 2.89 (t, *J* = 11.5 Hz, 2H), 2.46 (tt, *J* = 10.8, 4.0 Hz, 1H), 1.88 (d, *J* = 11.5 Hz, 2H), 1.61 (qd, *J* = 11.2, 4.0 Hz, 2H).
^{13}C NMR (125 MHz, $CDCl_3$) δ 179.6, 156.0, 52.8, 43.1, 40.6, 27.6.
FTIR (neat) ν_{max}: 3003, 2956, 2863, 1674, 1479, 1449, 1411, 1275, 1209, 1182, 1126, 1080, 1033, 930, 758, and 730 cm^{-1}.
HRMS (NSI) *m/z*: $[M+H]^+$ calcd. for $C_8H_{14}O_4N$, 188.0917; found, 188.0916.

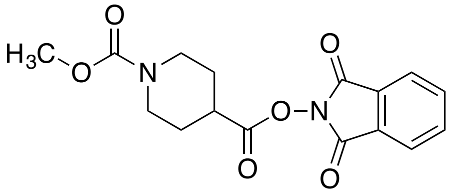

4-(1,3-dioxoisoindolin-2-yl) 1-methyl piperidine-1,4-dicarboxylate:
To a round bottom flask equipped with a stir bar was added 1-(methoxycarbonyl)piperidine-4-carboxylic acid (5.7 g, 30 mmol, 1 equiv), *N*-hydroxyphthalamide (4.9 g, 30 mmol, 1 equiv), DMAP (369 mg, 3 mmol, 0.1 equiv), and DCM (300 mL). DIC (4.7 mL, 30 mmol, 1 equiv) was then added dropwise via syringe. The reaction was allowed to stir at this temperature until the starting material had been consumed (determined by TLC). The reaction mixture was filtered over celite and rinsed with an additional 50 mL of DCM. The filtrate was concentrated by rotary evaporation and the residue was purified by flash chromatography (5% – 40% ethyl acetate/hexanes) to afford the product (7.52 g, 75% yield) as a white solid.

^{1}H NMR ^{1}H NMR (400 MHz, $CDCl_3$) δ 7.85 (dd, *J* = 5.6, 3.0 Hz, 1H), 7.77 (dd, *J* = 5.6, 3.0 Hz, 1H), 4.13 – 3.90 (m, 1H), 3.68 (d, *J* = 1.4 Hz, 2H), 3.05 (t, *J* = 11.0 Hz, 2H), 2.91 (tt, *J* = 10.2, 4.0 Hz, 1H), 2.10 – 2.00 (m, 3H), 1.84 (ddt, *J* = 13.3, 10.2, 5.3 Hz, 3H).
^{13}C NMR (101 MHz, $CDCl_3$) δ 170.5, 161.9, 155.8, 134.9, 128.8, 124.0, 52.7, 42.7, 38.3, 27.7.
FTIR (neat) ν_{max}: 2956, 2863, 1813, 1785, 1754, 1694, 1468, 1448, 1411, 1373, 1316, 1276, 1233, 1186, 1128, 1076, 1000, 968, 913, 877, 786, 767, 729, 695 cm^{-1}.
HRMS (NSI) *m/z*: $[M+H]^+$ calcd. for $C_{16}H_{17}O_6N_2$, 333.1081; found, 333.1081.

V. Procedure and Characterization Data:

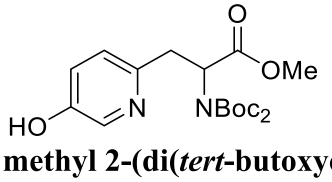

methyl 2-(di(*tert*-butoxycarbonyl)amino)-3-(5-hydroxypyridin-2-yl)propanoate (1):
Following the general procedure, the reaction of 6-bromopyridin-3-ol (174 mg, 1.00 mmol, 1 equiv), methyl 2-(di(*tert*-butoxycarbonyl)amino)acrylate (608 mg, 2.02 mmol, 2 equiv), $[Ir(ppy)_2(dtbbpy)]PF_6$ (10.4 mg, 0.011 mmol, 0.01 equiv) and Hantzsch ester (379 mg, 1.50 mmol, 1.5 equiv) provided the product (361 mg, 91% yield) as an off-white solid after purification by flash column chromatography (50% – 75% ethyl acetate/hexanes).

Mp: 163 °C (decomp.)
^{1}H NMR (400 MHz, $CDCl_3$) *δ* 10.58 (s, 1H), 8.13 (d, J = 2.9 Hz, 1H), 7.15 (dd, J = 8.5, 2.8 Hz, 1H), 6.99 (d, J = 8.4 Hz, 1H), 5.29 (dd, J = 10.2, 4.8 Hz, 1H), 3.69 (s, 3H), 3.51 (dd, J = 14.1, 4.9 Hz, 1H), 3.26 (dd, J = 14.2, 10.2 Hz, 1H), 1.34 (s, 18H).
^{13}C NMR (100 MHz, $CDCl_3$) *δ* 170.5, 153.3, 151.5, 147.6, 136.5, 125.2, 125.0, 83.2, 58.4, 52.3, 36.8, 27.7.
FTIR (neat) ν_{max}: 3002, 2980, 2950, 2933, 2612, 1744, 1729, 1697, 1573, 1364, 1280, 1232, 1253, 1142, and 1115 cm^{-1}.
HRMS (NSI) *m/z*: $[M+H]^+$ calcd. for $C_{20}H_{31}O_6N_2$, 397.1969; found, 397.1967.

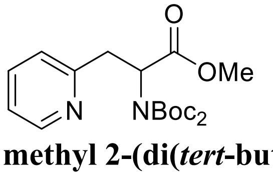

methyl 2-(di(*tert*-butoxycarbonyl)amino)-3-(pyridin-2-yl)propanoate (2):

1 mmol scale:
Following the general procedure, the reaction of 2-iodopyridine (207 mg, 1.01 mmol, 1 equiv), methyl 2-(di(*tert*-butoxycarbonyl)amino)acrylate (610 mg, 2.03 mmol, 2 equiv), $[Ir(ppy)_2(dtbbpy)]PF_6$ (9.9 mg, 0.011 mmol, 0.01 equiv) and Hantzsch ester (344 mg, 1.36 mmol, 1.3 equiv) provided the product (371 mg, 97% yield) as a clear, colorless crystalline solid after purification by flash column chromatography (10% – 50% ethyl acetate/hexanes).

25-mmol scale:
A 250-mL Schlenk flask equipped with a stir bar was charged with Hantzsch ester (6.33 g, 25 mmol, 1.0 equiv), $[Ir(ppy)_2(dtbbpy)]PF_6$ (22.9 mg, 0.025 mmol, 0.001 equiv), methyl-2-(di(*tert*-butoxycarbonyl)amino)but-2-enoate (9.03, 30 mmol, 1.2 equiv), 2-bromopyridine (3.95 g, 25 mmol, 1.0 equiv), and degassed DMSO/H_2O (5/1, V/V; 230 mL). The tube was connected to a N_2 line, and N_2 was streamed over the headspace of the reaction for 10 minutes before sealing with a rubber septum. The suspension was stirred under irradiation with blue LEDs for 18 hours while being cooled with a fan. The reaction was quenched with saturated sodium bicarbonate solution (1200 mL) and extracted with ethyl acetate (3 x 250 mL). The extracts were combined, passed through a silica plug, and concentrated by rotary evaporation. The residue was purified by flash column chromatography (5 – 60% ethyl acetate/hexanes) to afford the title compound (8.0 g, 84% yield) as a white crystalline solid.

Mp: 49 – 51 °C
^{1}H NMR (300 MHz, $CDCl_3$) δ 8.45 (d, J = 4.8 Hz, 1H), 7.50 (td, J = 7.7, 1.9 Hz, 1H), 7.09 – 7.00 (m, 2H), 5.45 (dd, J = 9.3, 5.2 Hz, 1H), 3.65 (s, 3H), 3.57 (dd, J = 14.2, 5.1 Hz, 1H), 3.25 (dd, J = 14.2, 9.4 Hz, 1H), 1.34 (s, 18H).
^{13}C NMR (75 MHz, $CDCl_3$) δ 170.8, 157.9, 151.5, 149.3, 136.1, 123.8, 121.4, 82.8, 58.1, 52.2, 38.7, 27.8.
FTIR (neat) ν_{max}: 3002, 2977, 2950, 2936, 1742, 1724, 1689, 1378, 1365, 12533, 1234, 1163, 1121, 1010, and 776 cm^{-1}.
HRMS (NSI) *m/z*: $[M+H]^+$ calcd. for $C_{19}H_{29}O_6N_2$, 381.2020; found, 381.2018.

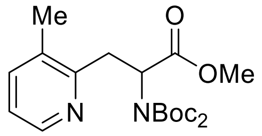

methyl 2-(di(*tert*-butoxycarbonyl)amino)-3-(3-methylpyridin-2-yl)propanoate (3):
Following the general procedure, the reaction of 2-bromo-3-methylpyridine (175 mg, 1.02 mmol, 1 equiv), methyl 2-(di(*tert*-butoxycarbonyl)amino)acrylate (604 mg, 2.03 mmol, 2 equiv), $[Ir(ppy)_2(dtbbpy)]PF_6$ (9.9 mg, 0.011 mmol, 0.01 equiv) and Hantzsch ester (344 mg, 1.36 mmol, 1.3 equiv) provided the product (376 mg, 94% yield) as a pale yellow oil after purification by flash column chromatography (2% – 6% tetrahydrofuran/dichloromethane).

^{1}H NMR (300 MHz, $CDCl_3$) δ 8.33 – 8.29 (m, 1H), 7.38 – 7.28 (m, 1H), 6.97 (dd, J = 7.6, 4.8 Hz, 1H), 5.64 (dd, J = 8.9, 5.1 Hz, 1H), 3.69 (s, 3H), 3.58 (dd, J = 14.8, 5.1 Hz, 1H), 3.34 (dd, J = 14.8, 8.9 Hz, 1H), 2.27 (s, 3H), 1.38 (s, 18H).
^{13}C NMR (75 MHz, $CDCl_3$) δ 171.1, 156.4, 151.6, 146.6, 137.4, 131.6, 121.3, 82.8, 57.9, 52.2, 35.3, 27.8, 18.7.
FTIR (neat) ν_{max}: 2979, 2952, 2935, 1793, 1743, 1698, 1575, 1451, 1436 ,1366, 1227, 1167, 1141, 1116, and 778 cm^{-1}.
HRMS (NSI) *m/z*: $[M+H]^+$ calcd. for $C_{20}H_{31}O_6N_2$, 395.2177; found, 395.2174.

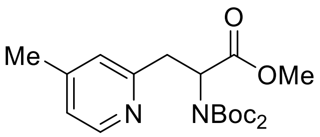

methyl 2-(di(*tert*-butoxycarbonyl)amino)-3-(4-methylpyridin-2-yl)propanoate (4): Following the general procedure, the reaction of 2-bromo-4-methylpyridine (173 mg, 1.01 mmol, 1 equiv), methyl 2-(di(*tert*-butoxycarbonyl)amino)acrylate (610 mg, 2.03 mmol, 2 equiv), $[Ir(ppy)_2(dtbbpy)]PF_6$ (9.6 mg, 0.011 mmol, 0.01 equiv) and Hantzsch ester (331 mg, 1.31 mmol, 1.3 equiv) provided the product (377 mg, 96% yield) as a pale yellow oil after purification by flash column chromatography (2% – 6% tetrahydrofuran/dichloromethane).

^{1}H NMR (300 MHz, $CDCl_3$) δ 8.33 (d, J = 5.8 Hz, 1H), 6.89 (s, 2H), 5.46 (dd, J = 9.3, 5.1 Hz, 1H), 3.68 (s, 3H), 3.55 (dd, J = 14.2, 5.1 Hz, 1H), 3.22 (dd, J = 14.2, 9.4 Hz, 1H), 2.24 (s, 3H), 1.37 (s, 18H).
^{13}C NMR (75 MHz, $CDCl_3$) δ 170.9, 157.7, 151.5, 149.1, 147.1, 124.7, 122.4, 82.8, 58.2, 52.2, 38.6, 27.8, 20.8.
FTIR (neat) ν_{max}: 2978, 2952, 2935, 1794, 1740, 1606, 1366, 1270, 1251, 1227, 1166, 1138, 852, and 778 cm^{-1}.
HRMS (NSI) *m/z*: $[M+H]^+$ calcd. for $C_{20}H_{31}O_6N_2$, 395.2177; found, 395.2175.

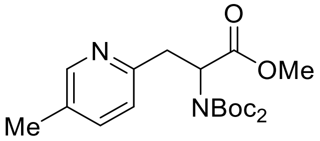

methyl 2-(di(*tert*-butoxycarbonyl)amino)-3-(5-methylpyridin-2-yl)propanoate (5): Following the general procedure, the reaction of 2-bromo-5-methylpyridine (175 mg, 1.02 mmol, 1 equiv), methyl 2-(di(*tert*-butoxycarbonyl)amino)acrylate (604 mg, 2.01 mmol, 2 equiv), $[Ir(ppy)_2(dtbbpy)]PF_6$ (9.2 mg, 0.010 mmol, 0.01 equiv) and Hantzsch ester (329 mg, 1.30 mmol, 1.3 equiv) provided the product (373 mg, 93% yield) as a pale yellow oil after purification by flash column chromatography (3% – 8% tetrahydrofuran/dichloromethane).

^{1}H NMR (300 MHz, $CDCl_3$) δ 8.38 – 8.24 (m, 1H), 7.45 – 7.31 (m, 1H), 6.98 (dd, J = 7.8, 0.8 Hz, 1H), 5.44 (dd, J = 9.4, 5.2 Hz, 1H), 3.69 (s, 3H), 3.56 (dd, J = 14.1, 5.2 Hz, 1H), 3.25 (dd, J = 14.1, 9.4 Hz, 1H), 2.24 (s, 3H), 1.38 (s, 18H).
^{13}C NMR (75 MHz, $CDCl_3$) δ 170.8, 154.9, 151.5, 149.7, 136.7, 130.6, 123.3, 82.8, 58.3, 52.2, 38.3, 27.8, 17.9.
FTIR (neat) ν_{max}: 2978, 2952, 2935, 1793, 174, 1710, 1366, 1270, 1167, 1137, and 808 cm^{-1}.
HRMS (NSI) *m/z*: $[M+H]^+$ calcd. for $C_{19}H_{30}O_6N_2$, 395.2177; found, 395.2173.

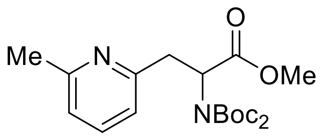

methyl 2-(di(*tert*-butoxycarbonyl)amino)-3-(6-methylpyridin-2-yl)propanoate (6):
Following the general procedure, the reaction of 2-bromo-6-methylpyridine (175 mg, 1.02 mmol, 1 equiv), methyl 2-(di(*tert*-butoxycarbonyl)amino)acrylate (606 mg, 2.01 mmol, 2 equiv), $[Ir(ppy)_2(dtbbpy)]PF_6$ (10.1 mg, 0.011 mmol, 0.01 equiv) and Hantzsch ester (334 mg, 1.30 mmol, 1.3 equiv) provided the product (388 mg, 97% yield) as a pale yellow oil after purification by flash column chromatography (3% – 8% tetrahydrofuran/dichloromethane).

^{1}H NMR (400 MHz, $CDCl_3$) δ 7.42 (t, J = 7.6 Hz, 1H), 6.90 (dd, J = 17.4, 7.6 Hz, 2H), 5.44 (dd, J = 9.7, 5.1 Hz, 1H), 3.69 (s, 3H), 3.53 (dd, J = 14.0, 5.1 Hz, 1H), 3.26 (dd, J = 13.9, 9.7 Hz, 1H), 2.46 (s, 3H), 1.37 (d, J = 0.9 Hz, 18H).
^{13}C NMR (75 MHz, $CDCl_3$) δ 170.8, 157.8, 157.1, 136.4, 120.9, 120.8, 82.8, 58.3, 52.2, 38.6, 27.8, 24.4.
FTIR (neat) ν_{max}: 3000, 2980, 2957, 2933, 1743, 172, 1689, 1579, 1456, 1430, 1377, 1364, 1278, 1246, 1231, 1166, 1011, and 788 cm^{-1}.
HRMS (NSI) *m/z*: $[M+H]^+$ calcd. for $C_{20}H_{31}O_6N_2$, 395.2177; found, 395.2170.

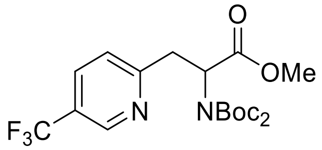

methyl 2-(di(*tert*-butoxycarbonyl)amino)-3-(5-(trifluoromethyl)pyridin-2-yl)propan-oate (7):
Following the general procedure, the reaction of 2-bromo-5-(trifluoromethyl)pyridine (225 mg, 1.00 mmol, 1 equiv), methyl 2-(di(*tert*-butoxycarbonyl)amino)acrylate (605 mg, 2.01 mmol, 2 equiv), $[Ir(ppy)_2(dtbbpy)]PF_6$ (9.6 mg, 0.011 mmol, 0.01 equiv) and Hantzsch ester (329 mg, 1.30 mmol, 1.3 equiv) provided the product (420 mg, 94% yield) as a pale yellow oil after purification by flash column chromatography (3% – 12% tetrahydrofuran/hexanes).

^{1}H NMR (300 MHz, $CDCl_3$) δ 8.76 – 8.68 (m, 1H), 7.76 (dd, *J* = 8.1, 2.2 Hz, 1H), 7.28 – 7.16 (m, 1H), 5.50 (dd, *J* = 8.7, 5.8 Hz, 1H), 3.71 – 3.59 (m, 4H), 3.34 (dd, *J* = 14.3, 8.8 Hz, 1H), 1.37 (s, 18H).
^{13}C NMR (75MHz, $CDCl_3$) δ 177.5, 170.7, 151.9, 149.9, 147.1, 140.0, 128.8, 121.7, 83.3, 57.7, 52.3, 39.3, 32.4, 27.8, 27.4.
^{19}F NMR (282 MHz, $CDCl_3$) δ -62.45.
FTIR (neat) ν_{max}: 2981, 2954, 2936, 1793, 1745, 1700, 1608, 1367, 1381, 1271, 1161, 1127, 1017, and 756 cm^{-1}.
HRMS (NSI) *m/z*: $[M+H]^+$ calcd. for $C_{20}H_{28}O_6N_2F_3$, 449.1894; found, 449.1891.

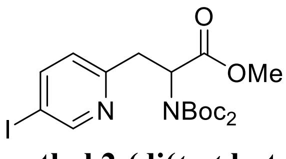

methyl 2-(di(*tert*-butoxycarbonyl)amino)-3-(5-iodopyridin-2-yl)propanoate (8): Following the general procedure, the reaction of 2,5-diiodopyridine (333 mg, 1.01 mmol, 1 equiv), methyl 2-(di(*tert*-butoxycarbonyl)amino)acrylate (601 mg, 2.00 mmol, 2 equiv), $[Ir(ppy)_2(dtbbpy)]PF_6$ (10.1 mg, 0.011 mmol, 0.01 equiv) and Hantzsch ester (321 mg, 1.27 mmol, 1.3 equiv) provided the product (378 mg, 74% yield) as white solid after purification by flash column chromatography (0% – 30% tetrahydrofuran/hexanes).

Mp: 59 – 62 °C
^{1}H NMR (300 MHz, $CDCl_3$) δ 7.74 8.66 (dd, J = 2.2, 0.7 Hz, 1H), 7.81 (dd, J = 8.1, 2.2 Hz, 1H), 6.90 (d, J = 8.2 Hz, 1H), 5.41 (dd, J = 9.0, 5.6 Hz, 1H), 3.66 (s, 3H), 3.51 (dd, J = 14.2, 5.6 Hz, 1H), 3.20 (dd, J = 14.2, 9.0 Hz, 1H), 1.37 (s, 18H).
^{13}C NMR (75MHz, $CDCl_3$) δ 170.6, 156.9, 155.2, 151.5, 144.3, 125.7, 90.7, 83.0, 57.8, 52.3, 38.1, 27.8.
FTIR (neat) ν_{max}: 3005, 2980, 2968, 2945, 1746, 1728, 1697, 1363, 1273, 1163, 1128, and 760 cm^{-1}.
HRMS (NSI) *m/z*: $[M+H]^+$ calcd. for $C_{19}H_{28}O_6N_2I$, 507.0987; found, 507.0976.

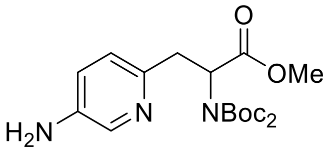

methyl 3-(5-aminopyridin-2-yl)-2-(di(*tert*-butoxycarbonyl)amino)propanoate (9): Following the general procedure, the reaction of 6-iodopyridin-3-amine (220 mg, 1.00 mmol, 1 equiv), methyl 2-(di(*tert*-butoxycarbonyl)amino)acrylate (603 mg, 2.00 mmol, 2 equiv), $[Ir(ppy)_2(dtbbpy)]PF_6$ (9.2 mg, 0.011 mmol, 0.01 equiv) and Hantzsch ester (379 mg, 1.50 mmol, 1.5 equiv) provided the product (280 mg, 71% yield) as an off-white solid after purification by flash column chromatography (50% – 100% ethyl acetate/hexanes).

Mp: 80 – 82 °C
^{1}H NMR (500 MHz, $CDCl_3$) δ 7.95 (s, 1H), 6.82 (s, 2H), 5.33 (dd, J = 9.6, 5.1 Hz, 1H), 3.72 (s, 2H), 3.66 (s, 4H), 3.44 (dd, J = 14.2, 5.1 Hz, 1H), 3.14 (dd, J = 14.2, 9.6 Hz, 1H), 1.36 (s, 18H).
^{13}C NMR (125 MHz, $CDCl_3$) δ 171.0, 151.5, 147.1, 141.0, 136.9, 123.8, 121.9, 82.8, 58.5, 52.1, 37.7, 27.8.
FTIR (neat) ν_{max}: 3452, 3367, 2983, 2970, 2943, 1730, 1718, 1488, 1366, 1218, 1141, and 1115 cm^{-1}.
HRMS (NSI) *m/z*: $[M+H]^+$ calcd. for $C_{19}H_{30}O_6N_3$, 396.2129; found, 396.2113.

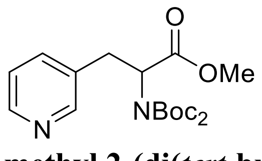

methyl 2-(di(*tert*-butoxycarbonyl)amino)-3-(pyridin-3-yl)propanoate (10):
Following the general procedure, the reaction of 3-iodopyridine (205 mg, 1.00 mmol, 1 equiv), methyl 2-(di(*tert*-butoxycarbonyl)amino)acrylate (609 mg, 2.02 mmol, 2 equiv), $[Ir(ppy)_2(dtbbpy)]PF_6$ (10.2 mg, 0.011 mmol, 0.01 equiv) and Hantzsch ester (379 mg, 1.50 mmol, 1.5 equiv) provided the product (279 mg, 73% yield) as a clear, colorless crystalline solid after purification by flash column chromatography (20% – 50% ethyl acetate/hexanes).

Mp: 76 – 78 °C
^{1}H NMR (400 MHz, $CDCl_3$) δ 8.36 (dd, J = 8.4, 3.5 Hz, 2H), 7.44 (d, J = 7.8 Hz, 1H), 7.11 (dd, J = 7.8, 4.8 Hz, 1H), 5.06 (dd, J = 10.3, 5.2 Hz, 1H), 3.66 (s, 3H), 3.34 (dd, J = 14.2, 5.2 Hz, 1H), 3.15 (dd, J = 14.3, 10.3 Hz, 1H), 1.30 (s, 18H).
^{13}C NMR (100 MHz, $CDCl_3$) δ 170.3, 151.5, 150.7, 147.9, 136.9, 132.9, 123.1, 83.2, 58.6, 52.3, 33.3, 27.7.
FTIR (neat) ν_{max}: 2975, 2951, 1793, 1739, 1392, 1368, 1138, 1112 1103, and 718 cm^{-1}.
HRMS (NSI) m/z: [M+H]+ calcd. for $C_{19}H_{29}O_6N_2$, 381.2020; found, 381.2015.

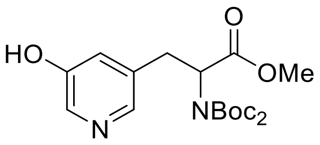

methyl 2-(di(*tert*-butoxycarbonyl)amino)-3-(5-hydroxypyridin-3-yl)propanoate (11):
Following the general procedure, the reaction of 5-iodopyridin-3-ol (220 mg, 1.00 mmol, 1 equiv), methyl 2-(di(*tert*-butoxycarbonyl)amino)acrylate (610 mg, 2.03 mmol, 2 equiv), $[Ir(ppy)_2(dtbbpy)]PF_6$ (9.4 mg, 0.010 mmol, 0.01 equiv) and Hantzsch ester (371 mg, 1.47 mmol, 1.5 equiv) provided the product (265 mg, 67% yield) as an off-white solid oil after purification by flash column chromatography (50% – 100% ethyl acetate/hexanes).

Mp: 112 – 116 °C
^{1}H NMR (300 MHz, $CDCl_3$) δ 8.10 (d, J = 2.6 Hz, 1H), 7.94 (d, J = 1.7 Hz, 1H), 7.12 (d, J = 2.2 Hz, 1H), 5.14 (dd, J = 10.2, 5.0 Hz, 1H), 3.74 (s, 3H), 3.41 (dd, J = 14.2, 5.0 Hz, 1H), 3.16 (dd, J = 14.2, 10.2 Hz, 1H), 1.38 (s, 18H).
^{13}C NMR (75MHz, $CDCl_3$) δ 170.3, 154.8, 151.6, 140.2, 135.3, 134.9, 125.4, 83.6, 58.8, 52.4, 33.2, 27.7.
FTIR (neat) ν_{max}: 2980, 2581, 1744, 1696, 1437, 1366, 1269, 1222, and 1166 cm^{-1}.
HRMS (NSI) *m/z*: $[M+H]^+$ calcd. for $C_{19}H_{29}O_7N_2$, 397.1969; found, 397.1962.

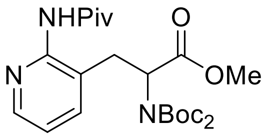

methyl 2-(di(*tert*-butoxycarbonyl)amino)-3-(2-pivalamidopyridin-3-yl)propanoate (12): Following the general procedure, the reaction of N-(3-iodopyridin-2-yl)pivalamide (302 mg, 0.99 mmol, 1 equiv), methyl 2-(di(*tert*-butoxycarbonyl)amino)acrylate (609 mg, 2.02 mmol, 2 equiv), $[Ir(ppy)_2(dtbbpy)]PF_6$ (9.9 mg, 0.010 mmol, 0.01 equiv) and Hantzsch ester (380 mg, 1.50 mmol, 1.5 equiv) provided the product (347 mg, 73% yield) as a white solid after purification by flash column chromatography (20% – 100% ethyl acetate/hexanes).

Mp: 104 – 107 °C
^{1}H NMR (300 MHz, $CDCl_3$) δ 8.56 (s, 1H), 8.30 (dd, J = 4.8, 1.8 Hz, 1H), 7.49 (dd, J = 7.6, 1.9 Hz, 1H), 7.05 (dd, J = 7.6, 4.8 Hz, 1H), 5.21 (dd, J = 8.5, 6.0 Hz, 1H), 3.66 (s, 3H), 3.46 (dd, J = 14.5, 6.0 Hz, 1H), 3.01 (dd, J = 14.5, 8.6 Hz, 1H), 1.34 (s, 18H), 1.29 (s, 9H).
^{13}C NMR (75MHz, $CDCl_3$) δ 177.5, 170.7, 151.9, 149.9, 147.1, 140.0, 128.8, 121.7, 83.3, 57.7, 52.3, 39.3, 32.4, 27.8, 27.4.
FTIR (neat) ν_{max}: 3160, 2970, 1749, 1738, 1697, 1437, 1365, and 1140 cm^{-1}.
HRMS (NSI) *m/z*: $[M+H]^+$ calcd. for $C_{24}H_{38}O_7N_3$, 480.2704; found, 480.2694.

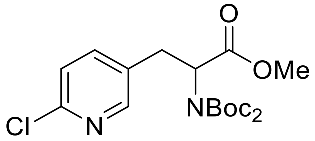

methyl 2-(di(*tert*-butoxycarbonyl)amino)-3-(6-chloropyridin-3-yl)propanoate (13): Following the general procedure, the reaction of 2-chloro-5-iodopyridine (240 mg, 1.00 mmol, 1 equiv), methyl 2-(di(*tert*-butoxycarbonyl)amino)acrylate (610 mg, 2.03 mmol, 2 equiv), $[Ir(ppy)_2(dtbbpy)]PF_6$ (9.7 mg, 0.011 mmol, 0.01 equiv) and Hantzsch ester (379 mg, 1.50 mmol, 1.5 equiv) provided the product (318 mg, 77% yield) as a clear, colorless crystalline solid after purification by flash column chromatography (3% – 25% tetrahydrofuran/hexanes).

Mp: 87 – 90 °C
^{1}H NMR (300 MHz, $CDCl_3$) δ 8.18 (d, J = 1.9 Hz, 1H), 7.49 (dd, J = 8.2, 2.5 Hz, 1H), 7.22 (d, J = 7.8 Hz, 1H), 5.09 (dd, J = 10.0, 5.4 Hz, 1H), 3.73 (s, 3H), 3.38 (dd, J = 14.3, 5.4 Hz, 1H), 3.20 (dd, J = 14.2, 10.0 Hz, 1H), 1.39 (s, 18H).
^{13}C NMR (75MHz, $CDCl_3$) δ 170.2, 151.7, 150.5, 149.8, 139.9, 132.0, 123.8, 83.5, 58.4, 52.4, 32.6, 27.8.
FTIR (neat) ν_{max}: 3007, 1970, 1954, 2937, 2916, 2848, 1743, 1729, 1690, 1340, 1274, 1201, 1161, 1019, and 758 cm^{-1}.
HRMS (NSI) *m/z*: $[M+H]^+$ calcd. for $C_{19}H_{28}O_6N_2Cl$, 415.1644; found, 415.1638.

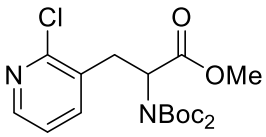

methyl 2-(di(*tert*-butoxycarbonyl)amino)-3-(2-chloropyridin-3-yl)propanoate (14): Following the general procedure, the reaction of 2-chloro-3-iodopyridine (239 mg, 1.00 mmol, 1 equiv), methyl 2-(di(*tert*-butoxycarbonyl)amino)acrylate (611 mg, 2.03 mmol, 2 equiv), [Ir(ppy)$_2$(dtbbpy)]PF$_6$ (10.2 mg, 0.011 mmol, 0.01 equiv) and Hantzsch ester (383 mg, 1.51 mmol, 1.5 equiv) provided the product (306 mg, 74% yield) as a white crystalline solid after purification by flash column chromatography (10% – 30% ethyl acetate/hexanes).

Mp: 71 – 74 °C
^{1}H NMR (300 MHz, CDCl$_3$) δ 8.23 – 8.18 (m, 1H), 7.45 (dd, *J* = 7.5, 1.9 Hz, 1H), 7.12 (dd, *J* = 7.5, 4.8 Hz, 1H), 5.24 (dd, *J* = 10.8, 4.3 Hz, 1H), 3.71 (s, 3H), 3.58 (dd, *J* = 14.2, 4.3 Hz, 1H), 3.26 (dd, *J* = 14.2, 10.8 Hz, 1H), 1.33 (s, 18H).
^{13}C NMR (75MHz, CDCl$_3$) δ 170.2, 151.7, 151.2, 148.0, 140.4, 132.1, 122.5, 83.3, 56.9, 52.4, 33.7, 27.7.
FTIR (neat) ν_{max}: 2980, 2952, 2936, 1794, 1745, 1696, 1367, 1137, 1126, and 748 cm^{-1}.
HRMS (NSI) *m/z*: [M+H]$^+$ calcd. for $C_{19}H_{28}O_6N_2Cl$, 415.1630; found, 415.1623.

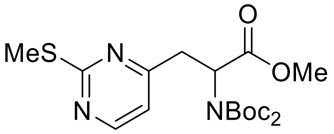

methyl 2-(di(*tert*-butoxycarbonyl)amino)-3-(2-(methylthio)pyrimidin-4-yl) propanoate (15):
Following the general procedure, the reaction of 4-iodo-2-(methylthio)pyrimidine (249 mg, 0.99 mmol, 1 equiv), methyl 2-(di(*tert*-butoxycarbonyl)amino)acrylate (610 mg, 2.03 mmol, 2 equiv), [Ir(ppy)$_2$(dtbbpy)]PF$_6$ (9.4 mg, 0.010 mmol, 0.01 equiv) and Hantzsch ester (370 mg, 1.46 mmol, 1.5 equiv) provided the product (338 mg, 80% yield) as a white crystalline solid after purification by flash column chromatography (5% – 50% ethyl acetate/hexanes).

Mp: 71 – 75 °C
^{1}H NMR (400 MHz, CDCl$_3$) δ 8.32 (d, J = 5.0 Hz, 1H), 6.78 (d, J = 5.0 Hz, 1H), 5.46 (dd, J = 9.2, 5.4 Hz, 1H), 3.68 (s, 3H), 3.45 (dd, J = 14.2, 5.5 Hz, 1H), 3.19 (dd, J = 14.2, 9.2 Hz, 1H), 2.47 (s, 3H), 1.38 (s, 18H).
^{13}C NMR (75 MHz, CDCl$_3$) *δ* 172.3, 170.4, 166.8, 156.9, 151.5, 116.2, 83.2, 57.0, 52.3, 37.9, 27.8, 13.9.
FTIR (neat) ν_{max}: 2983, 2940, 1948, 1703, 1565, 1550, 1451, 1296, 1162, 1136, and 778 cm^{-1}.
HRMS (NSI) *m/z*: [M+H]$^+$ calcd. for $C_{17}H_{28}O_6N_3S$, 428.1850; found, 428.1841.

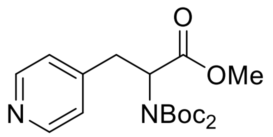

methyl 2-(di(*tert*-butoxycarbonyl)amino)-3-(pyridin-4-yl)propanoate (16):
Following the general procedure, the reaction of 4-iodopyridine (205 mg, 1.00 mmol, 1 equiv), methyl 2-(di(*tert*-butoxycarbonyl)amino)acrylate (609 mg, 2.02 mmol, 2 equiv), [Ir(ppy)$_2$(dtbbpy)]PF$_6$ (10.2 mg, 0.012 mmol, 0.01 equiv) and Hantzsch ester (376 mg, 1.50 mmol, 1.5 equiv) provided the product (129 mg, 34% yield) as a white crystalline solid after purification by flash column chromatography (10% – 80% ethyl acetate/hexanes).

Mp: 97 – 100 °C
^{1}H NMR (500 MHz, CDCl3) δ 8.49 – 8.41 (m, 2H), 7.10 (d, J = 5.7 Hz, 2H), 5.15 (dd, J = 10.1, 5.3 Hz, 1H), 3.72 (s, 3H), 3.40 (dd, J = 14.0, 5.3 Hz, 1H), 3.19 (dd, J = 14.0, 10.2 Hz, 1H), 1.37 (s, 18H).
^{13}C NMR (125 MHz, CDCl$_3$) *δ* 170.3, 151.6, 149.6, 146.7, 124.8, 83.3, 58.2, 52.4, 35.6, 27.8.
FTIR (neat) ν_{max}: 3005, 2970, 2948, 1748, 1736, 1690, 1366, 1228, 1217, and 1140 cm^{-1}.
HRMS (NSI) *m/z*: [M+H]$^+$ calcd. for $C_{19}H_{29}O_6N_2$, 381.2020; found, 381.2017.

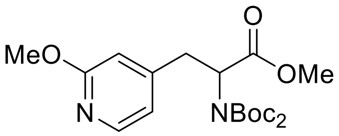

methyl 2-(di(*tert*-butoxycarbonyl)amino)-3-(5-methoxypyridin-3-yl)propanoate (17):
Following the general procedure, the reaction of 4-bromo-2-methoxypyridine (195 mg, 1.04 mmol, 1 equiv), methyl 2-(di(*tert*-butoxycarbonyl)amino)acrylate (604 mg, 2.01 mmol, 2 equiv), [Ir(ppy)$_2$(dtbbpy)]PF$_6$ (9.2 mg, 0.010 mmol, 0.01 equiv) and Hantzsch ester (385 mg, 1.52 mmol, 1.5 equiv) provided the product (280 mg, 66% yield) as a white crystalline solid after purification by flash column chromatography (5% – 50% ethyl acetate/hexanes).

Mp: 69 – 72 °C
^{1}H NMR (300 MHz, CDCl$_3$) δ 8.00 (dd, J = 5.3, 0.7 Hz, 1H), 6.67 (dd, J = 5.3, 1.5 Hz, 1H), 6.52 (dd, J = 1.5, 0.7 Hz, 1H), 5.12 (dd, J = 10.2, 5.1 Hz, 1H), 3.84 (s, 3H), 3.70 (s, 3H), 3.32 (dd, J = 13.9, 5.1 Hz, 1H), 3.12 (dd, J = 13.9, 10.2 Hz, 1H), 1.36 (s, 18H).
^{13}C NMR (75MHz, CDCl$_3$) *δ* 170.3, 164.3, 151.6, 149.3, 146.6, 118.1, 111.5, 83.2, 58.2, 53.2, 52.3, 35.4, 27.7.
FTIR (neat) ν_{max}: 2979, 2950, 1745, 1697, 1613, 1561, 1380, 1270, 1244, 1136, 1112, and 774 cm^{-1}.
HRMS (NSI) *m/z*: [M+H]$^+$ calcd. for $C_{20}H_{31}O_7N_2$, 411.2126; found, 411.2118.

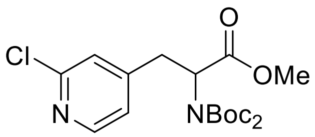

methyl 2-(di(*tert*-butoxycarbonyl)amino)-3-(2-chloropyridin-4-yl)propanoate (18): Following the general procedure, the reaction of 2-chloro-4-iodopyridine (244 mg, 1.02 mmol, 1 equiv), methyl 2-(di(*tert*-butoxycarbonyl)amino)acrylate (610 mg, 2.03 mmol, 2 equiv), $[Ir(ppy)_2(dtbbpy)]PF_6$ (9.2 mg, 0.010 mmol, 0.01 equiv) and Hantzsch ester (390 mg, 1.54 mmol, 1.5 equiv) provided the product (352 mg, 83% yield) as a white crystalline solid after purification by flash column chromatography (10% – 30% ethyl acetate/hexanes).

Mp: 104 – 108 °C
^{1}H NMR (400 MHz, $CDCl_3$) δ 8.23 (d, *J* = 5.1 Hz, 1H), 7.13 (s, 1H), 7.04 (dd, *J* = 5.1, 1.5 Hz, 1H), 5.12 (dd, *J* = 10.0, 5.4 Hz, 1H), 3.71 (s, 3H), 3.38 (dd, *J* = 14.0, 5.4 Hz, 1H), 3.18 (dd, *J* = 14.0, 10.0 Hz, 1H), 1.38 (s, 18H).
^{13}C NMR (75MHz, $CDCl_3$) δ 170.0, 151.7, 151.4, 150.1, 149.4, 125.2, 123.6, 83.5, 57.9, 52.5, 35.3, 27.8.
FTIR (neat) ν_{max}: 3054, 3001, 2982, 2972, 1743, 1727, 1692, 1596, 1375, 1275, 1139, 1128, and 1010 cm^{-1}.
HRMS (NSI) *m/z*: $[M+H]^+$ calcd. for $C_{19}H_{28}O_6N_2Cl$, 415.1630; found, 415.1626.

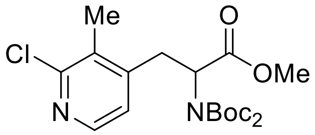

methyl 2-(di(*tert*-butoxycarbonyl)amino)-3-(2-chloro-3-methylpyridin-4-yl) propanoate (19):
Following the general procedure, the reaction of 2-chloro-4-iodo-3-methylpyridine (250 mg, 0.99 mmol, 1 equiv), methyl 2-(di(*tert*-butoxycarbonyl)amino)acrylate (600 mg, 1.99 mmol, 2 equiv), $[Ir(ppy)_2(dtbbpy)]PF_6$ (9.0 mg, 0.010 mmol, 0.01 equiv) and Hantzsch ester (381 mg, 1.51 mmol, 1.5 equiv) provided the product (330 mg, 78% yield) as a white crystalline solid after purification by flash column chromatography (0% – 30% tetrahydrofuran/hexanes).

Mp: 83 – 86 °C
^{1}H NMR (300 MHz, $CDCl_3$) *δ* 8.05 (dd, *J* = 4.9, 0.7 Hz, 1H), 6.93 (d, *J* = 4.9 Hz, 1H), 5.10 (dd, *J* = 10.7, 4.7 Hz, 1H), 3.71 (s, 3H), 3.43 (dd, *J* = 14.1, 4.7 Hz, 1H), 3.28 (dd, *J* = 14.1, 10.7 Hz, 1H), 2.33 (s, 3H), 1.33 (s, 18H).
^{13}C NMR (75MHz, $CDCl_3$) *δ* 170.0, 152.3, 151.6, 147.9, 146.1, 131.6, 124.7, 83.4, 57.4, 52.5, 33.7, 27.7, 15.6.
FTIR (neat) ν_{max}: 2996, 2980, 2955, 2936, 1752, 1741, 1706, 1365, 1249, 1136, 1113, and 1015 cm^{-1}.
HRMS (NSI) *m/z*: $[M+H]^+$ calcd. for $C_{20}H_{30}O_6N_2Cl$, 429.1787; found, 429.1779.

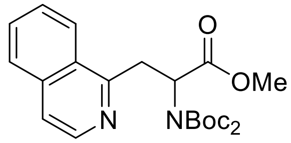

methyl 2-(di(*tert*-butoxycarbonyl)amino)-3-(isoquinolin-1-yl)propanoate (20):
Following the general procedure, the reaction of 1-iodoisoquinoline (260 mg, 1.02 mmol, 1 equiv), methyl 2-(di(*tert*-butoxycarbonyl)amino)acrylate (610 mg, 2.03 mmol, 2 equiv), $[Ir(ppy)_2(dtbbpy)]PF_6$ (9.4 mg, 0.010 mmol, 0.01 equiv) and Hantzsch ester (371 mg, 1.47 mmol, 1.5 equiv) provided the product (381 mg, 87% yield) as a colorless oil after purification by flash column chromatography (1% – 10% tetrahydrofuran/dichloromethane).

^{1}H NMR (300 MHz, $CDCl_3$) δ 8.43 (d, J = 5.7 Hz, 1H), 8.14 (d, J = 8.4 Hz, 1H), 7.79 (d, J = 8.5 Hz, 1H), 7.71 – 7.62 (m, 1H), 7.57 (ddd, J = 8.3, 6.9, 1.3 Hz, 1H), 7.51 (d, J = 5.7 Hz, 1H), 5.77 (dd, J = 9.1, 4.7 Hz, 1H), 4.15 (dd, J = 15.1, 4.7 Hz, 1H), 3.93 (dd, J = 15.1, 9.1 Hz, 1H), 3.76 (s, 3H), 1.34 (s, 18H).
^{13}C NMR (75MHz, $CDCl_3$) δ 171.0, 158.1, 151.7, 141.9, 136.1, 129.8, 127.5, 127.2, 127.1, 124.9, 119.4, 82.8, 58.2, 52.3, 35.1, 27.8.
FTIR (neat) ν_{max}: 2978, 1793, 1741, 1696, 1366, 1381, 1273, 1251, 1226, 1135, 1109, and 764 cm^{-1}.
HRMS (NSI) *m/z*: $[M+H]^+$ calcd. for $C_{23}H_{31}O_6N_2$, 431.2177; found, 431.2168.

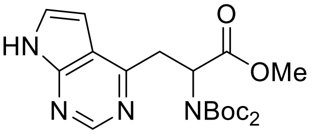

methyl 2-(di(*tert*-butoxycarbonyl)amino)-3-(7H-pyrrolo[2,3-d]pyrimidin-4-yl) propanoate (21):
Following the general procedure, the reaction of 4-bromo-7H-pyrrolo[2,3-d]pyrimidine (199 mg, 1.01 mmol, 1 equiv), methyl 2-(di(*tert*-butoxycarbonyl)amino)acrylate (611 mg, 2.03 mmol, 2 equiv), $[Ir(ppy)_2(dtbbpy)]PF_6$ (9.o mg, 0.010 mmol, 0.01 equiv) and Hantzsch ester (381 mg, 1.51 mmol, 1.5 equiv) provided the product (401 mg, 95% yield) as an off-white crystalline solid after purification by flash column chromatography (5% – 50% ethyl acetate/hexanes).

Mp: 112 – 114 °C
^{1}H NMR (300 MHz, $CDCl_3$) δ 11.84 (s, 1H), 8.82 (s, 1H), 7.35 (dd, J = 3.6, 2.2 Hz, 1H), 6.56 (dd, J = 3.6, 1.8 Hz, 1H), 5.72 (dd, J = 9.0, 5.1 Hz, 1H), 3.88 (dd, J = 14.4, 5.2 Hz, 1H), 3.72 (s, 3H), 3.67 (dd, J = 14.4, 9.0 Hz, 1H), 1.35 (s, 18H).
^{13}C NMR (75MHz, $CDCl_3$) δ 170.7, 159.1, 151.6, 151.4, 150.7, 125.5, 118.4, 99.5, 83.1, 57.5, 52.4, 36.0, 27.8.
FTIR (neat) ν_{max}: 3127, 3002, 2974, 2852, 1745, 1691, 1583, 1379, 1346, 1142, 1119, 971, and 748 cm^{-1}.
HRMS (NSI) *m/z*: $[M+H]^+$ calcd. for $C_{20}H_{29}O_6N_4$, 421.2082; found, 421.2073.

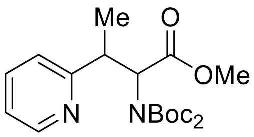

methyl 2-(di(tert-butoxycarbonyl)amino)-3-(pyridin-2-yl)butanoate (22):
Following the general procedure, the reaction of 2-bromopyridine (161 mg, 1.02 mmol, 1 equiv), methyl-2-(di(tert-butoxycarbonyl)amino)but-2-enoate (638 mg, 2.02 mmol, 2 equiv), $[Ir(ppy)_2(dtbbpy)]PF_6$ (9.4 mg, 0.010 mmol, 0.01 equiv) and Hantzsch ester (329 mg, 1.30 mmol, 1.3 equiv) provided an inseparable 3:1 mixture of diastereomers (261 mg, 66% yield) as a white crystalline solid after purification by flash column chromatography (10% – 60% ethyl acetate/hexanes).

For the mixture of diastereomers:
Mp: 84 – 88 °C
^{1}H NMR (600 MHz, $CDCl_3$) δ 8.56 – 8.49 (m, $1H_{dr1}$ + $1H_{dr2}$), 7.60 (td, J = 7.6, 1.9 Hz, $1H_{dr1}$), 7.55 (td, J = 7.6, 1.9 Hz, $1H_{dr2}$), 7.25 (d, J = 7.9 Hz, $1H_{dr1}$), 7.12 (d, J = 7.8 Hz, $1H_{dr2}$), 7.09 (m, $1H_{dr1}$+$1H_{dr2}$), 5.95 (d, J = 9.8 Hz, $1H_{dr1}$), 5.17 (d, J = 9.7 Hz, $1H_{dr2}$), 3.87 (dq, J = 9.7, 6.7 Hz, $1H_{dr2}$), 3.76 (m, $1H_{dr1}$ + $3H_{dr2}$), 3.58 (s, $3H_{dr1}$), 1.58 (d, J = 7.2, $1H_{dr2}$) 1.56 (s, $18H_{dr1}$), 1.42 (s, $18H_{dr2}$), 1.18 (d, J = 7.2 Hz, $3H_{dr1}$).
^{13}C NMR (150 MHz, $CDCl_3$) δ 171.0, 171.0, 164.2, 162.6, 152.3, 151.7, 149.3, 149.0, 136.1, 135.9, 123.2, 123.1, 121.3, 121.1, 83.1, 82.6, 62.1, 61.1, 52.1, 42.7, 28.0, 27.9, 20.2, 18.5
FTIR (neat) ν_{max}: 2979, 2936, 1793, 1745, 1699, 1523, 1365, 1143, 1122, 1104, 845, 758, and 749 cm^{-1}.
HRMS (NSI) *m/z*: $[M+H]^+$ calcd. for $C_{20}H_{31}O_6N_2$, 395.2177; found, 395.2170.

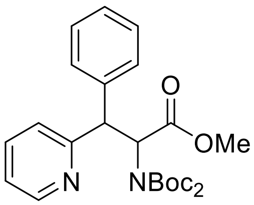

methyl 2-(di(tert-butoxycarbonyl)amino)-3-phenyl-3-(pyridin-2-yl)propanoate (23):
Following the general procedure, the reaction of 2-bromopyridine (158 mg, 1.00 mmol, 1 equiv), methyl 2-(di(tert-butoxycarbonyl)amino)-3-phenylacrylate (750 mg, 2.00 mmol, 2 equiv), $[Ir(ppy)_2(dtbbpy)]PF_6$ (10.0 mg, 0.011 mmol, 0.01 equiv) and Hantzsch ester (340 mg, 1.34 mmol, 1.3 equiv) provided an inseparable 4:1 mixture of diastereomers (246 mg, 54% yield) as a colorless oil after purification by flash column chromatography (10% – 60% ethyl acetate/hexanes).

For the mixture of diastereomers:
^{1}H NMR (600 MHz, C_6D_6) δ 8.44 (ddd, J = 4.9, 1.9, 0.9 Hz, $1H_{dr1}$), 8.40 (ddd, J = 4.8, 1.9, 0.9 Hz, $1H_{dr2}$), 7.88 – 7.82 (m, $2H_{dr2}$), 7.60 (d, J = 6.9 Hz, $2H_{dr1}$), 7.27 (d, J = 10.2 Hz, $1H_{dr1}$), 7.13 (t, J = 7.7 Hz, $2H_{dr2}$), 7.10 (d, J = 7.9 Hz, $1H_{dr2}$), 7.07 (t, J = 7.7 Hz, $2H_{dr1}$), 7.01 (d, J=8.1, $1H_{dr1}$), 6.99 – 6.93 (m, $2H_{dr1}$ + $2H_{dr2}$), 6.54 (ddd, J = 7.3, 4.9, 1.3 Hz, $1H_{dr1}$), 6.50 (ddd, J = 7.5, 4.8, 1.2 Hz, $1H_{dr2}$), 6.42 (d, J = 10.2 Hz, $1H_{dr2}$), 5.46 (d, J = 10.3 Hz,

1H$_{dr2}$), 5.35 (d, J = 10.2 Hz, 1H$_{dr1}$), 3.19 (s, 3H$_{dr1}$), 3.17 (s, 3H$_{dr2}$), 1.38 (s, 18H$_{dr2}$), 1.34 (s, 18H$_{dr1}$).
^{13}C NMR (150 MHz, C_6D_6) δ 170.4, 170.1, 161.9, 161.2, 152.4, 152.1, 149.0, 148.5, 142.3, 140.5, 135.8, 135.5, 129.3, 129.0, 128.2, 128.0, 126.6, 126.5, 124.2, 123.7, 120.9, 120.8, 81.7, 81.7, 61.7, 60.1, 54.8, 53.7, 51.2, 27.6, 27.6.
FTIR (neat) ν_{max}: 2970, 2941, 1753, 1736, 1719, 1367, 1227, 1139, 1108, 770, and 755 cm^{-1}.
HRMS (NSI) *m/z*: $[M+H]^+$ calcd. for $C_{25}H_{33}O_6N_2$, 457.2333; found, 457.2325.

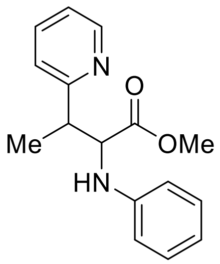

methyl 2-(phenylamino)-3-(pyridin-2-yl)butanoate (24):
Following the general procedure, the reaction of 2-bromopyridine (158 mg, 1.00 mmol, 1.0 equiv), methyl 2-(phenylamino)but-2-enoate (380 mg, 2.00 mmol, 2.0 equiv), $[Ir(ppy)_2(dtbbpy)]PF_6$ (10.0 mg, 0.011 mmol, 0.01 equiv) and Hantzsch ester (325 mg, 1.28 mmol, 1.30 equiv) provided an inseparable 5:4 mixture of diastereomers (186 mg, 69% yield) as a yellow crystalline solid after purification by flash column chromatography (5% – 50% ethyl acetate/hexanes).

For the mixture of diastereomers:
Mp: 62 – 64 °C
^{1}H NMR (500 MHz, $CDCl_3$) δ 8.64 – 8.54 (m, 1H$_{dr1}$ + 1H$_{dr2}$), 7.65 – 7.52 (m, 1H$_{dr1}$ + 1H$_{dr2}$), 7.23 – 7.07 (m, 4H$_{dr1}$ + 4H$_{dr2}$), 6.70 (m, 1H$_{dr1}$ + 1H$_{dr2}$), 6.63 (d, J = 7.6 Hz, 2H$_{dr1}$), 6.58 – 6.51 (d, J = 8.0 Hz, 2H$_{dr2}$), 4.97 (d, J = 8.9 Hz, 1H$_{dr1}$), 4.61 (d, J = 8.3 Hz, 1H$_{dr2}$), 4.42 (m, 1H$_{dr1}$ + 1H$_{dr2}$), 3.62 (s, 3H$_{dr1}$), 3.59 (s, 3H$_{dr2}$), 3.53 – 3.40 (m, 1H$_{dr1}$ + 1H$_{dr2}$), 1.49 (d, J = 7.1 Hz, 3H$_{dr2}$), 1.44 (d, J = 7.0 Hz, 3H$_{dr1}$).
^{13}C NMR (125 MHz, $CDCl_3$) δ 173.8, 173.5, 161.7, 161.6, 149.2, 149.1, 147.2, 147.1, 136.5, 136.5, 129.2, 129.1, 122.4, 122.3, 121.9, 118.2, 117.8, 113.6, 113.2, 61.9, 61.7, 51.9, 51.8, 44.3, 43.7, 17.4, 15.4.
FTIR (neat) ν_{max}: 3373, 3053, 2969, 2950, 1732, 1601, 1590, 1507, 1149, 908, 729, and 691 cm^{-1}.
HRMS (NSI) *m/z*: $[M+H]^+$ calcd. for $C_{16}H_{19}O_2N_2$, 271.1441; found, 271.1439
.

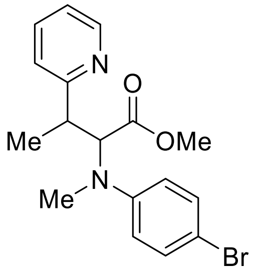

methyl 2-((4-bromophenyl)(methyl)amino)-3-(pyridin-2-yl)butanoate (25):
Following the general procedure, the reaction of 2-bromopyridine (160 mg, 1.01 mmol, 1 equiv), methyl 2-((4-bromophenyl)(methyl)amino)but-2-enoate (570 mg, 2.01 mmol, 2 equiv), $[Ir(ppy)_2(dtbbpy)]PF_6$ (9.1 mg, 0.010 mmol, 0.01 equiv) and Hantzsch ester (325 mg, 1.28 mmol, 1.3 equiv) provided an inseparable 4:3 mixture of diastereomers (311 mg, 86% yield) as a colorless oil after purification by flash column chromatography (10% – 50% ethyl acetate/hexanes).

For the mixture of diastereomers:
^{1}**H NMR** (500 MHz, $CDCl_3$) 8.56 – 8.52 (m, $1H_{dr1}$), 8.44 (m, $1H_{dr2}$), 7.60 (td, J = 7.7, 1.8 Hz, $1H_{dr1}$), 7.49 (td, J = 7.6, 1.8 Hz, $1H_{dr2}$), 7.40 – 7.29 (m, $2H_{dr1}$), 7.23 (d, J = 7.8 Hz, $1H_{dr1}$), 7.18 (d, J = 9.0 Hz, $2H_{dr2}$), 7.12 (ddd, J = 7.5, 4.9, 1.2 Hz, $1H_{dr1}$), 7.06 (d, J = 7.8 Hz, $1H_{dr2}$), 7.03 (ddd, J = 7.5, 4.8, 1.2 Hz, $1H_{dr2}$), 6.85 (d, J = 9.1 Hz, $2H_{dr1}$), 6.59 (d, J = 9.0 Hz, $2H_{dr2}$), 4.98 (d, J = 10.8 Hz, $1H_{dr1}$), 4.78 (d, J = 11.0 Hz, $1H_{dr2}$), 3.72 (s, $3H_{dr2}$), 3.67 – 3.57 (m, $1H_{dr1}$ + $1H_{dr2}$), 3.46 (s, $3H_{dr1}$), 2.93 (s, $3H_{dr1}$), 2.72 (s, $3H_{dr2}$), 1.32 (d, J = 6.7 Hz, $3H_{dr2}$), 1.21 (d, J = 7.0 Hz, $3H_{dr1}$).
^{13}**C NMR** (125 MHz, $CDCl_3$) δ 171.4, 171.4, 162.7, 161.9, 149.3, 149.2, 148.8, 136.4, 136.2, 131.8, 131.4, 123.4, 122.4, 121.7, 121.6, 115.4, 115.0, 109.6, 109.5, 66.7, 65.5, 51.7, 51.5, 41.7, 41.6, 33.1, 32.7, 18.5, 18.2.
FTIR (neat) ν_{max}: 2990, 2948, 2904, 2817, 1718, 1648, 1490, 1434, 1252, 1202, 1120, 1108, 1042, 811, 776, and 766 cm^{-1}.
HRMS (NSI) *m/z*: $[M+H]^+$ calcd. for $C_{17}H_{20}O_2N_2Br$, 363.0703; found, 363.0707.

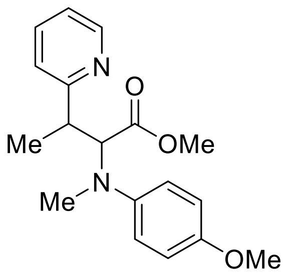

methyl 2-((4-methoxyphenyl)(methyl)amino)-3-(pyridin-2-yl)butanoate 26):
Following the general procedure, the reaction of 2-bromopyridine (159 mg, 1.00 mmol, 1 equiv), methyl 2-((4-methoxyphenyl)(methyl)amino)but-2-enoate (477 mg, 2.03 mmol, 2 equiv), $[Ir(ppy)_2(dtbbpy)]PF_6$ (10.1 mg, 0.011 mmol, 0.01 equiv) and Hantzsch ester (335 mg, 1.32 mmol, 1.3 equiv) provided an inseparable 3:1 mixture of diastereomers (182mg, 58% yield) as a colorless oil after purification by flash column chromatography (5% – 40% ethyl acetate/hexanes).

For the mixture of diastereomers:
^{1}H NMR (600 MHz, $CDCl_3$) δ 8.53 (ddd, J = 4.9, 1.9, 0.9 Hz, $1H_{dr2}$), 8.49 (ddd, J = 4.9, 1.9, 0.9 Hz, $1H_{dr1}$), 7.59 (td, J = 7.7, 1.9 Hz, $1H_{dr2}$), 7.50 (td, J = 7.6, 1.8 Hz, $1H_{dr1}$), 7.22 (dt, J = 7.8, 1.1 Hz, $1H_{dr2}$), 7.13 – 7.07 (m, $1H_{dr1}$ + $1H_{dr2}$), 7.04 (ddd, J = 7.6, 4.8, 1.1 Hz, $1H_{dr1}$), 6.98 – 6.92 (m, $2H_{dr2}$), 6.87 – 6.83 (m, $2H_{dr2}$), 6.75 – 6.65 (m, $4H_{dr1}$), 4.85 (d, J = 11.0 Hz, $1H_{dr2}$), 4.68 (d, J = 11.1 Hz, $1H_{dr1}$), 3.77 (s, $3H_{dr2}$), 3.64 – 3.55 (m, $1H_{dr1}$ + $1H_{dr2}$), 3.43 (s, $3H_{dr2}$), 2.91 (s, $3H_{dr2}$), 2.71 (s, $3H_{dr1}$), 1.31 (d, J = 6.7 Hz, $3H_{dr1}$), 1.27 (d, J = 7.0 Hz, $3H_{dr2}$).
^{13}C NMR (150 MHz, $CDCl_3$) δ 174.2, 174.1, 171.7, 163.1, 162.5, 152.4, 152.2, 149.2, 149.1, 144.9, 144.6, 136.3, 136.1, 123.4, 122.6, 121.5, 121.5, 116.2, 115.5, 114.6, 114.1, 83.3, 82.7, 68.5, 67.0, 55.6, 55.5, 52.9, 52.8, 51.4, 51.2, 41.6, 41.6, 33.3, 33.1, 25.7, 25.6, 18.4, 18.3, 8.0, 7.8.
FTIR (neat) ν_{max}: 3043, 2949, 2832, 1730, 1589, 1509, 1242, 1165, 1034, 991, 817, 786, and 749 cm^{-1}.
HRMS (NSI) *m/z*: $[M+H]^+$ calcd. for $C_{18}H_{23}O_3N_2$, 315.1703; found, 315.1703.

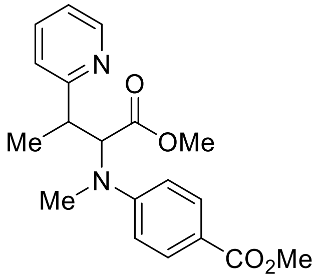

methyl 4-((1-methoxy-1-oxo-3-(pyridin-2-yl)butan-2-yl)(methyl)amino)benzoate (27):
Following the general procedure, the reaction of 2-bromopyridine (158mg, 1.00 mmol, 1 equiv), methyl 4-((1-methoxy-1-oxobut-2-en-2-yl)(methyl)amino)benzoate (530 mg, 2.02 mmol, 2 equiv), $[Ir(ppy)_2(dtbbpy)]PF_6$ (9.1 mg, 0.010 mmol, 0.01 equiv) and Hantzsch ester (340 mg, 1.34 mmol, 1.3 equiv) provided an inseparable 5:2 mixture of diastereomers (267 mg, 78% yield) as a low-melting white solid after purification by flash column chromatography (5% – 50% ethyl acetate/hexanes).

For the mixture of diastereomers:
^{1}H NMR (600 MHz, $CDCl_3$) δ 8.56 (ddd, J = 4.9, 1.8, 0.9 Hz, $1H_{dr1}$), 8.40 (ddd, J = 4.9, 1.8, 0.9 Hz, $1H_{dr2}$), 7.99 – 7.91 (m, $2H_{dr1}$), 7.82 – 7.77 (m, $1H_{dr2}$), 7.62 (td, J = 7.6, 1.8 Hz, $1H_{dr1}$), 7.47 (td, J = 7.7, 1.8 Hz, $1H_{dr2}$), 7.25 (d, J = 7.5 Hz, $1H_{dr1}$), 7.14 (ddd, J = 7.6, 4.8, 1.1 Hz, $1H_{dr1}$), 7.05 (dd, J = 7.7, 1.1 Hz, $1H_{dr1}$), 7.00 (ddd, J = 7.6, 4.8, 1.1 Hz, $1H_{dr2}$), 6.96 (d, J = 9.0 Hz, $2H_{dr1}$), 6.69 (d, J = 9.0 Hz, $2H_{dr2}$), 5.20 (d, J = 10.7 Hz, $1H_{dr1}$), 4.96 (d, J = 10.9 Hz, $1H_{dr2}$), 3.87 (s, $3H_{dr1}$), 3.83 (s, $3H_{dr2}$), 3.75 (s, $3H_{dr2}$), 3.71 – 3.61 (m, $1H_{dr1}$ + $1H_{dr2}$), 3.49 (s, $3H_{dr1}$), 3.04 (s, $3H_{dr1}$), 2.83 (s, $3H_{dr2}$), 1.36 (d, J = 6.7 Hz, $3H_{dr2}$), 1.19 (s, $3H_{dr1}$).
^{13}C NMR (150 MHz, $CDCl_3$) δ 171.3, 171.2, 167.2, 162.5, 161.5, 153.4, 152.9, 149.3, 149.2, 136.5, 136.3, 131.3, 130.9, 123.4, 122.3, 121.8, 121.7, 118.5, 118.4, 112.0, 111.9, 65.6, 64.7, 51.9, 51.7, 51.5, 51.5, 41.9, 41.7, 33.2, 32.9, 18.5, 18.1.
FTIR (neat) ν_{max}: 2949, 2839, 1735, 1705, 1602, 1518, 1433, 1276, 1186, 1110, 769, and 747 cm^{-1}.

HRMS (NSI) *m/z*: $[M+H]^+$ calcd. for $C_{19}H_{23}O_4N_2$, 343.1652; found, 343.1651.

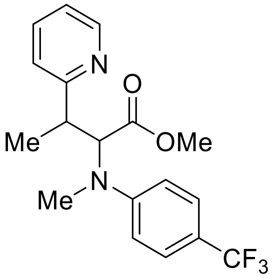

methyl 2-(methyl(4-(trifluoromethyl)phenyl)amino)-3-(pyridin-2-yl)butanoate (28): Following the general procedure, the reaction of 2-bromopyridine (158 mg, 1.00 mmol, 1 equiv), methyl 2-(methyl(4-(trifluoromethyl)phenyl)amino)but-2-enoate (551 mg, 2.02 mmol, 2 equiv), $[Ir(ppy)_2(dtbbpy)]PF_6$ (9.0 mg, 0.010 mmol, 0.01 equiv) and Hantzsch ester (329 mg, 1.30 mmol, 1.3 equiv) provided an inseparable 5:2 mixture of diastereomers (274 mg, 78% yield) as a colorless oil after purification by flash column chromatography (0% – 25% ethyl acetate/hexanes).

For the mixture of diastereomers:
^{1}H NMR (600 MHz, $CDCl_3$) 8.55 (dd, *J* = 5.0, 1.7 Hz, $1H_{dr1}$), 8.41 (dd, *J* = 4.9, 1.7 Hz, $1H_{dr2}$), 7.62 (td, *J* = 7.6, 1.8 Hz, $1H_{dr1}$), 7.50 (d, *J* = 8.6 Hz, $2H_{dr1}$ + $1H_{dr2}$), 7.34 (d, *J* = 8.6 Hz, $2H_{dr2}$), 7.27 – 7.22 (m, $1H_{dr1}$), 7.16 – 7.12 (m, $1H_{dr2}$), 7.01 (m, $2H_{dr1}$ + $1H_{dr2}$), 6.74 (d, *J* = 8.6 Hz, $2H_{dr2}$), 5.15 (d, *J* = 10.8 Hz, $1H_{dr1}$), 4.92 (d, *J* = 11.0 Hz, $1H_{dr2}$), 3.75 (s, $3H_{dr2}$), 3.64 (dq, *J* = 10.5, 7.2 Hz, $1H_{dr1}$ + $1H_{dr2}$), 3.49 (s, $3H_{dr1}$), 3.02 (s, $3H_{dr1}$), 2.80 (s, $3H_{dr2}$), 1.35 (d, *J* = 6.7 Hz, $3H_{dr2}$), 1.20 (d, *J* = 7.0 Hz, $3H_{dr1}$).
^{13}C NMR (150 MHz, $CDCl_3$) δ 171.3, 171.3, 162.5, 161.6, 152.3, 151.9, 149.3, 136.5, 136.3, 126.5 (q, *J* = 3.7 Hz), 126.0 (q, *J* = 3.7 Hz), 124.9 (q, *J* = 268.6), 124.9 (q, *J* = 268.7), 123.4, 122.4, 121.8, 118.9 (q, *J* = 32), 118.8 (q, *J* = 33), 112.5, 112.3, 65.9, 64.9, 51.9, 51.7, 41.8, 33.2, 32.8, 18.5, 18.2.
^{19}F NMR (282 MHz, CDCl3) δ -61.01.
FTIR (neat) ν_{max}: 2971, 2953, 2830, 1735, 1614, 1526, 1454, 1326, 1296, 1163, 1104, 1069, and 819 cm^{-1}.
HRMS (NSI) *m/z*: $[M+H]^+$ calcd. for $C_{18}H_{20}O_2N_2F_3$, 353.1471; found, 353.1480.

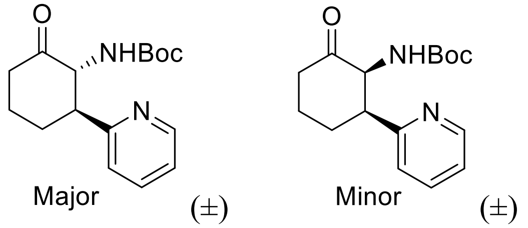

tert-butyl (2-oxo-6-(pyridin-2-yl)cyclohexyl)carbamate (29): Following the general procedure, 2-bromopyridine (160 mg, 1.01 mmol, 1 equiv) was reacted with tert-butyl (6-oxocyclohex-1-en-1-yl)carbamate (421 mg, 2.00 mmol, 2 equiv), $[Ir(ppy)_2(dtbbpy)]PF_6$ (9.0 mg, 0.010 mmol, 0.01 equiv) and Hantzsch ester (331 mg, 1.31 mmol, 1.3 equiv). Analysis of the crude ^{1}H NMR spectrum indicated a mixture of stereoisomers (cis:trans = 2:9), and purification by flash column chromatography (5% – 100% ethyl acetate/hexanes) provided the title compounds (trans, 170 mg, 59% yield; cis, 52 mg, 18% yield).

Major isomer (trans):
R_f: 0.3 (50% ethyl acetate/hexanes)
Mp: 181 – 185 °C
^{1}H NMR (300 MHz, $CDCl_3$) δ 8.67 – 8.31 (m, 1H), 7.59 (td, J = 7.7, 1.8 Hz, 1H), 7.20 (d, J = 7.9 Hz, 1H), 7.12 (ddd, J = 7.5, 4.8, 1.1 Hz, 1H), 5.00 (s, 1H), 4.69 – 4.51 (m, 1H), 2.97 – 2.83 (m, 1H), 2.63 – 2.43 (m, 2H), 2.32 – 1.89 (m, 3H), 1.74 (dddd, J = 16.9, 9.9, 8.6, 4.1 Hz, 1H), 1.21 (s, 9H).
^{13}C NMR (75 MHz, $CDCl_3$) δ 207.1, 160.2, 155.1, 149.2, 136.3, 122.3, 121.9, 79.3, 62.5, 54.6, 41.0, 31.5, 28.0, 26.1.
FTIR (neat) ν_{max}: 3206, 3025, 2978, 2935, 2864, 1737, 1714, 1686, 1555, 169, 1014, 785, and 735 cm^{-1}.
HRMS (NSI) *m/z*: $[M+H]^+$ calcd. for $C_{116}H_{23}O_3N_2$, 291.1703; found, 291.1699.

Minor isomer (cis):
R_f: 0.7 (50% ethyl acetate/hexanes)
Mp: 92 – 95 °C
^{1}H NMR (300 MHz, $CDCl_3$) δ 8.52 – 8.22 (m, 1H), 7.58 (td, J = 7.7, 1.9 Hz, 1H), 7.09 (dtd, J = 7.5, 5.2, 1.1 Hz, 2H), 5.48 (d, J = 7.3 Hz, 1H), 4.44 (ddd, J = 7.2, 5.7, 1.2 Hz, 1H), 3.98 (dt, J = 5.9, 3.9 Hz, 1H), 2.74 – 2.50 (m, 1H), 2.46 – 2.12 (m, 2H), 2.09 – 1.88 (m, 1H), 1.73 (dq, J = 11.2, 3.3 Hz, 2H), 1.34 (s, 9H).
^{13}C NMR (75 MHz, $CDCl_3$) δ 206.0, 160.80, 155.7, 148.2, 136.5, 123.3, 121.5, 79.3, 60.0, 48.7, 40.2, 30.7, 28.2, 20.8.
FTIR (neat) ν_{max}: 3448, 3005, 2969, 2944, 1737, 1719, 1694, 1494, 1365, 1352, 1229, and 770 cm^{-1}.
HRMS (NSI) *m/z*: $[M+H]^+$ calcd. for $C_{16}H_{23}O_3N_2$, 291.1703; found, 291.1698.

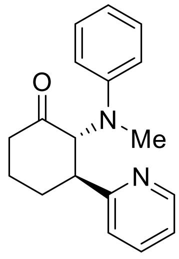

(±)

2-(methyl(phenyl)amino)-3-(pyridin-2-yl)cyclohexan-1-one (30): Following the general procedure, the reaction of 2-bromopyridine (160 mg, 1.01 mmol, 1 equiv), 2-(methyl(phenyl)amino)cyclohex-2-en-1-one (401 mg, 2.00 mmol, 2 equiv), $[Ir(ppy)_2(dtbbpy)]PF_6$ (9.5 mg, 0.010 mmol, 0.01 equiv) and Hantzsch ester (330 mg, 1.30 mmol, 1.3 equiv) provided the product (196 mg, 70% yield) as a white solid after purification by flash column chromatography (10% – 80% ethyl acetate/hexanes).

Mp: 111 – 115 °C
^{1}H NMR (300 MHz, $CDCl_3$) δ 8.52 – 8.37 (m, 1H), 7.48 (tdd, J = 7.7, 1.9, 1.0 Hz, 1H), 7.13 – 6.90 (m, 4H), 6.59 (td, J = 7.3, 1.0 Hz, 1H), 6.57 – 6.53 (m, 2H), 4.97 (d, J = 11.6 Hz, 1H), 3.60 – 3.27 (m, 1H), 2.77 (d, J = 1.0 Hz, 3H), 2.63 – 2.47 (m, 2H), 2.26 – 2.09 (m, 3H), 1.86 – 1.72 (m, 1H).

^{13}**C NMR** (75 MHz, $CDCl_3$) δ 196.4, 149.0, 144.6, 143.1, 128.8, 118.4, 114.7, 39.5, 39.3, 26.0, 22.9.
FTIR (neat) ν_{max}: 2950, 2933, 2866, 1710, 1596, 1505, 745, and 690 cm^{-1}.
HRMS (NSI) *m/z*: $[M+H]^+$ calcd. for $C_{18}H_{21}ON_2$, 281.1648; found, 281.1647.

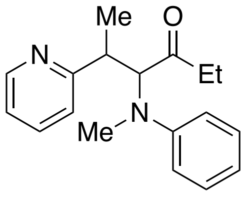

4-(methyl(phenyl)amino)-5-(pyridin-2-yl)hexan-3-one (31):
Following the general procedure, the reaction of 2-bromopyridine (159 mg, 1.00 mmol, 1 equiv), 4-(methyl(phenyl)amino)hex-4-en-3-one (406 mg, 2.00 mmol, 2 equiv), $[Ir(ppy)_2(dtbbpy)]PF_6$ (9.2 mg, 0.010 mmol, 0.01 equiv) and Hantzsch ester (325 mg, 1.28 mmol, 1.3 equiv) provided the product (180 mg, 64% yield) as a yellow oil after purification by flash column chromatography (0% – 30% ethyl acetate/hexanes).

^{1}**H NMR** (400 MHz, $CDCl_3$) δ 8.46 (ddd, *J* = 4.9, 1.9, 0.9 Hz, 1H), 7.57 (td, *J* = 7.8, 1.8 Hz, 1H), 7.36 – 7.26 (m, 2H), 7.24 (dd, *J* = 7.7, 1.1 Hz, 1H), 7.06 (ddd, *J* = 7.6, 4.9, 1.3 Hz, 1H), 7.00 (d, *J* = 8.2 Hz, 2H), 6.84 – 6.70 (m, 1H), 5.24 (d, *J* = 10.6 Hz, 1H), 3.62 (dq, *J* = 10.6, 7.1 Hz, 1H), 2.81 (s, 3H), 2.37 (dq, *J* = 17.7, 7.3 Hz, 1H), 2.21 (dq, *J* = 17.7, 7.3 Hz, 1H), 1.14 (d, *J* = 7.1 Hz, 3H), 0.96 – 0.70 (m, 3H).
^{13}**C NMR** (100 MHz, $CDCl_3$) δ 208.4, 163.8, 149.8, 136.3, 129.4, 123.7, 121.2, 117.3, 112.7, 68.8, 39.6, 34.4, 32.6, 18.5, 7.4.
FTIR (neat) ν_{max}: 3061, 3027, 2976, 2937, 2906, 2816, 1682, 1625, 1597, 1499, 1355, 1339, 1310, 1221, 1184, 1164, 1109, 747, and 692 cm^{-1}.
HRMS (NSI) *m/z*: $[M+H]^+$ calcd. for $C_{19}H_{23}ON_2$, 283.1805; found, 283.1804.

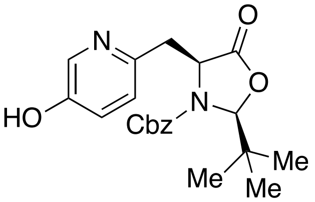

benzyl (2*S*,4*S*)-2-(*tert*-butyl)-4-((5-hydroxypyridin-2-yl)methyl)-5-oxooxazolidine-3-carboxylate (33):
Following the general procedure, the reaction of 6-bromopyridin-3-ol (43 mg, 0.25 mmol, 1 equiv), **32** (145 mg, 0.50 mmol, 2 equiv), $[Ir(ppy)_2(dtbbpy)]PF_6$ (2 mg, 0.0025 mmol, 0.01 equiv) and Hantzsch ester (82 mg, 0.33 mmol, 1.3 equiv) provided the product (55 mg, 57% yield) as a colorless oil after purification by flash column chromatography (10% – 50% ethyl acetate/hexanes).

^{1}**H NMR** (500 MHz, $CDCl_3$) δ 8.06 (s, 1H), 7.36 – 7.27 (m, 3H), 7.24 – 7.20 (m, 2H), 7.12 (q, *J* = 8.5 Hz, 2H), 5.57 (s, 1H), 5.06 (d, *J* = 12.0 Hz, 1H), 4.91 (t, *J* = 12.0 Hz, 1H), 4.75 (t, *J* = 6.9 Hz, 1H), 3.32 (ddd, *J* = 56.7, 14.1, 7.1 Hz, 2H), 1.02 (s, 9H).

^{13}C NMR (125 MHz, $CDCl_3$) δ 171.8, 156.0, 153.2, 146.9, 136.0, 135.2, 128.7, 128.7, 128.5, 125.5, 125.3, 96.6, 68.5, 58.0, 39.5, 37.2, 25.1.
FTIR (neat) ν_{max}: 3066, 3033, 2960, 2925, 2872, 1790, 1717, 1575, 1481, 1392, 1346, 1335, 1272, 1228, 1198, 1172, 1121, 1037, 976, 908, 839, 720, and 668cm^{-1}.
HRMS (NSI) *m/z*: $[M+H]^+$ calcd. for $C_{23}H_{25}O_5N_2$, 385.1758; found, 385.17753.

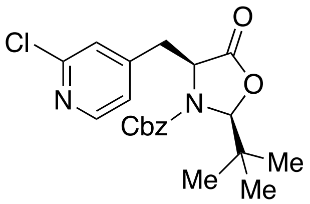

benzyl (2*S*,4*S*)-2-(*tert*-butyl)-4-((2-chloropyridin-4-yl)methyl)-5-oxooxazolidine-3-carboxylate (34):
Following the general procedure, the reaction of 2-chloro-4-iodopyridine (60 mg, 0.25 mmol, 1 equiv), **32** (145 mg, 0.50 mmol, 2 equiv), $[Ir(ppy)_2(dtbbpy)]PF_6$ (2 mg, 0.0025 mmol, 0.01 equiv) and Hantzsch ester (82 mg, 0.33 mmol, 1.3 equiv) provided the product (75 mg, 75% yield) as a colorless oil after purification by flash column chromatography (5% – 30% ethyl acetate/hexanes).

^{1}H NMR (500 MHz, $CDCl_3$) δ 8.20 (d, *J* = 5.0 Hz, 1H), 7.41 – 7.38 (m, 3H), 7.27 (dd, *J* = 6.6, 2.8 Hz, 2H), 7.20 (s, 1H), 7.08 – 7.02 (m, 1H), 5.58 (s, 1H), 5.07 (dd, *J* = 70.4, 11.7 Hz, 2H), 4.43 (dd, *J* = 7.6, 5.4 Hz, 1H), 3.19 – 3.02 (m, 2H), 1.00 (s, 9H).
^{13}C NMR (125 MHz, $CDCl_3$) δ 171.4, 155.7, 151.8, 149.7, 149.1, 134.8, 129.1, 129.0, 128.8, 125.1, 123.5, 96.6, 68.9, 58.1, 38.4, 37.3, 25.0.
FTIR (neat) ν_{max}: 3063, 3033, 2970, 2873, 1790, 1720, 1594, 1549, 1481, 1388, 1341, 1305, 1230, 1200, 1173, 1122, 1087, 1036, 980, 899, 878, 839, 746, and 698 cm^{-1}.
HRMS (NSI) *m/z*: $[M+H]^+$ calcd. for $C_{21}H_{24}O_4N_2Cl$, 403.1419; found, 403.1414.

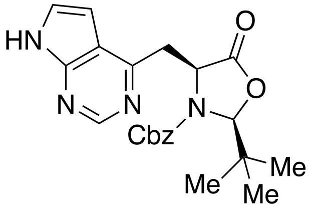

benzyl (2*S*,4*S*)-4-((7*H*-pyrrolo[2,3-*d*]pyrimidin-4-yl)methyl)-2-(*tert*-butyl)-5-oxooxazolidine-3-carboxylate (35):
Following the general procedure, the reaction of 4-bromo-5*H*-pyrrolo[3,2-*d*]pyrimidine (50 mg, 0.25 mmol, 1 equiv), **32** (145 mg, 0.50 mmol, 2 equiv), $[Ir(ppy)_2(dtbbpy)]PF_6$ (2 mg, 0.0025 mmol, 0.01 equiv) and Hantzsch ester (82 mg, 0.33 mmol, 1.3 equiv) provided the product (82 mg, 80% yield) as a pale yellow oil after purification by flash column chromatography (20% – 70% ethyl acetate/hexanes).

^{1}H NMR (500 MHz, $CDCl_3$) δ 11.04 (s, 1H), 8.78 (s, 1H), 7.31 (dd, *J* = 3.4, 2.3 Hz, 1H), 7.25 (d, *J* = 2.9 Hz, 3H), 7.12 (s, 2H), 6.57 (s, 1H), 5.63 (s, 1H), 5.19 (t, *J* = 6.8 Hz, 1H), 5.02 (d, *J* = 12.1 Hz, 1H), 4.76 (d, *J* = 11.0 Hz, 1H), 3.70 – 3.51 (m, 2H), 1.07 (s, 9H).

^{13}C NMR (125 MHz, $CDCl_3$) δ 171.8, 157.8, 155.8, 151.5, 150.9, 135.0, 128.6, 128.5, 128.3, 125.5, 117.9, 99.5, 96.7, 68.2, 56.7, 39.0, 37.3, 25.1.
FTIR (neat) ν_{max}: 3202, 3133, 2969, 1792, 1721, 1585, 1393, 1349, 1307, 1230, 1194, 1119, 1043, 976, 903, 733, and 698cm^{-1}.
HRMS (NSI) *m/z*: $[M+H]^+$ calcd. for $C_{22}H_{25}O_4N_4$, 409.1870; found, 409.1866.

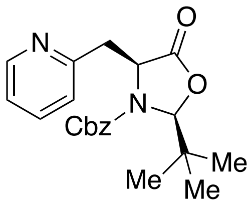

benzyl (2*S*,4*S*)-2-(*tert*-butyl)-5-oxo-4-(pyridin-2-ylmethyl)oxazolidine-3-carboxylate (36):
Following the general procedure, the reaction of 2-bromopyridine (39 mg, 0.25 mmol, 1 equiv), **32** (145 mg, 0.50 mmol, 2 equiv), $[Ir(ppy)_2(dtbbpy)]PF_6$ (2 mg, 0.0025 mmol, 0.01 equiv) and Hantzsch ester (82 mg, 0.33 mmol, 1.3 equiv) provided the product (63 mg, 68% yield) as a colorless oil after purification by flash column chromatography (10% – 40% ethyl acetate/hexanes).

^{1}H NMR (500 MHz, $CDCl_3$) δ 8.49 (d, *J* = 4.2 Hz, 1H), 7.53 (td, *J* = 7.7, 1.8 Hz, 1H), 7.32 (td, *J* = 4.8, 1.8 Hz, 3H), 7.24 (dd, *J* = 6.9, 2.6 Hz, 2H), 7.16 (d, *J* = 7.7 Hz, 1H), 7.08 (dd, *J* = 7.1, 5.3 Hz, 1H), 5.59 (s, 1H), 5.08 (d, *J* = 12.2 Hz, 1H), 4.97 (t, *J* = 6.9 Hz, 1H), 4.85 (d, *J* = 11.9 Hz, 1H), 3.38 (dd, *J* = 14.0, 6.9 Hz, 1H), 3.30 (dd, *J* = 14.0, 6.9 Hz, 1H) 1.03 (s, 9H).
^{13}C NMR (125 MHz, $CDCl_3$) δ 172.0, 156.7, 155.9, 149.4, 136.2, 135.3, 128.6, 128.4, 128.3, 123.7, 121.9, 96.4, 68.1, 57.5, 41.4, 37.2, 25.0.
FTIR (neat) ν_{max}: 3064, 3034, 3010, 2970, 2873, 1791, 1716, 1593, 1475, 1438, 1391, 1347, 1305, 1231, 1173, 1190, 1121, 1036, 977, 932, 825, 731, and 697 cm^{-1}.
HRMS (NSI) *m/z*: $[M+H]^+$ calcd. for $C_{21}H_{25}O_4N_2$, 369.1809; found, 369.1804.

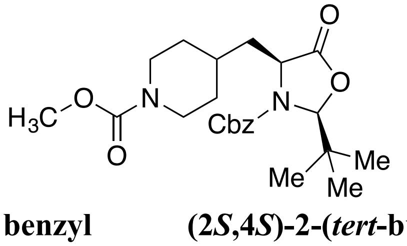

benzyl (2*S*,4*S*)-2-(*tert*-butyl)-4-((1-(methoxycarbonyl)piperidin-4-yl)methyl)-5-oxooxazolidine-3-carboxylate (38):
Following the general procedure, the reaction of 4-(1,3-dioxoisoindolin-2-yl) 1-methyl piperidine-1,4-dicarboxylate (83 mg, 0.25 mmol, 1 equiv), **32** (145 mg, 0.5 mmol, 2 equiv), $[Ir(ppy)_2(dtbbpy)]PF_6$ (2 mg, 0.0025 mmol, 0.01 equiv) and Hantzsch ester (82 mg, 0.33 mmol, 1.3 equiv) provided the product (93 mg, 86% yield) as a white solid after purification by flash column chromatography (5% – 30% ethyl acetate/hexanes).

^{1}H NMR (500 MHz, $CDCl_3$) δ 7.39 – 7.34 (m, 5H), 5.59 – 5.39 (m, 3H), 4.66 (d, *J* = 11.0 Hz, 1H), 4.06 – 3.52 (m, 7H), 2.52 – 2.20 (m, 2H), 1.82 (dd, *J* = 14.1, 7.6 Hz, 1H), 1.65 – 1.35 (m, 3H), 1.06 (s, 9H), 0.93 – 0.66 (m, 1H).
^{13}C NMR (125 MHz, $cdcl_3$) δ 168.5, 156.0, 153.9, 134.6, 129.7, 129.1, 128.8, 95.6, 71.7, 68.1, 52.5, 44.1, 43.6, 38.8, 37.1, 31.5, 26.9.
FTIR (neat) ν_{max}: 2959, 2852, 1792, 1724, 1701, 1472, 1448, 1396, 1309, 1258, 1193, 1125, 1043, 966, 916, 757, 731, and 699cm^{-1}.
HRMS (NSI) *m/z*: $[M+H]^+$ calcd. for $C_{23}H_{33}O_6N_2$, 433.2333; found, 433.2333.

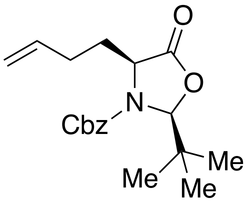

benzyl (2*S*,4*S*)-4-(but-3-en-1-yl)-2-(*tert*-butyl)-5-oxooxazolidine-3-carboxylate (39):
Following the general procedure, the reaction of 3-bromoprop-1-ene (22 μL, 0.25 mmol, 1 equiv), **32** (145 mg, 0.50 mmol, 2 equiv), $[Ir(ppy)_2(dtbbpy)]PF_6$ (2 mg, 0.0025 mmol, 0.01 equiv) and Hantzsch ester (82 mg, 0.33 mmol, 1.3 equiv) provided the product (35 mg, 42% yield) as a colorless oil after purification by flash column chromatography (5% – 30% ethyl acetate/hexanes).

^{1}H NMR (500 MHz, $CDCl_3$) δ 7.41 – 7.34 (m, 5H), 5.76 (td, *J* = 16.7, 6.7 Hz, 1H), 5.55 (s, 1H), 5.22 – 5.12 (td *J* = 16.9, 11.9 Hz , 2H), 5.06 (d, *J* = 17.1 Hz, 1H), 4.98 (d, *J* = 11.2 Hz, 1H), 4.30 (dd, *J* = 7.8, 6.7 Hz, 1H), 2.34 (m, 2H), 2.06 – 1.98 (m, 1H), 1.90 (m, 1H), 0.96 (s, 9H).
^{13}C NMR (125 MHz, $CDCl_3$) δ 172.7, 156.1, 137.0, 135.3, 128.8, 128.8, 128.6, 115.9, 96.4, 68.5, 56.5, 37.1, 32.5, 30.3, 25.0.
FTIR (neat) ν_{max}: 3068, 3034, 2960, 2873, 1790, 1716, 1641, 1391, 1324, 1282, 1195, 1117, 1041, 979, 914, and 968 cm^{-1}.
HRMS (NSI) m/z: [M+H]+ calcd. for $C_{19}H_{26}O_4N$, 332.1856; found, 332.1859.

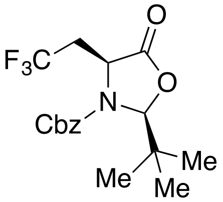

benzyl (2*S*,4*S*)-2-(*tert*-butyl)-5-oxo-4-(2,2,2-trifluoroethyl)oxazolidine-3-carboxylate (40):
A 20-mL screw-top test tube equipped with a stir bar was charged with **32** (289 mg, 1.0 mmol, 2 equiv), $[Ir(ppy)_2(dtbbpy)]PF_6$ (5 mg, 0.005 mmol, 0.01 equiv) and Hantzsch ester (164 mg, 0.65 mmol. 1.3 equiv). The tube was sealed PTFE/silicon septum and connected to a Schlenk line. The atmosphere was exchanged by applying vacuum and backfilling with N_2 (this process was conducted a total of three times). The reaction vial was disconnected from the nitrogen line and massed.

The trifluoromethyl iodide lecture bottle outlet was fitted with a rubber septum, and a cannula needle (cooled to -78 °C) was used to condense trifluoromethyl iodide into the reaction tube. The reaction tube and contents were massed once more to measure the loading of trifluoromethyl iodide (861 mg, 4.4 mmol, 4.4 equiv). The reaction was then stirred for 18 hours under irradiation by blue LEDs. The reaction was quenched with aqueous sodium bicarbonate then extracted with ethyl acetate (5 x 25 mL). The combined extracts were dried over sodium sulfate then concentrated by rotary evaporation to provide the product (222 mg, 93% yield) as a colorless oil after purification by flash column chromatography (10% – 50% ethyl acetate/hexanes).

^{1}H NMR (500 MHz, DMSO-d_6, 80 °C) δ 7.44 – 7.34 (m, 7H), 5.43 – 5.34 (m, 2H), 4.92 (s, 1H), 3.58 (s, 1H), 3.43 (dq, *J* = 15.5, 10.2 Hz, 1H), 3.17 (s, 1H), 0.94 (s, 9H).
^{13}C NMR (126 MHz, DMSO-d_6, 80 °C) δ 165.3, 152.5, 133.8, 128.4, 128.3, 128.1, 124.3 (q, *J* = 278.6 Hz), 95.9, 68.0, 66.7, 37.6, 33.8 (q, *J* = 28.5), 26.0.
^{19}F NMR (282 MHz, cdcl$_3$) δ -59.92.
FTIR (neat) ν_{max}: 3035, 2965, 1799, 1732, 1483, 1394, 1333, 1290, 1230, 1200, 1138, 1117, 1044, 1018, 976, 916, 819, 798, 767, 755, and 696 cm^{-1}.
HRMS (NSI) *m/z*: [M+H]$^+$ calcd. for $C_{17}H_{21}O_4NF_3$, 360.1417; found, 360.1421.

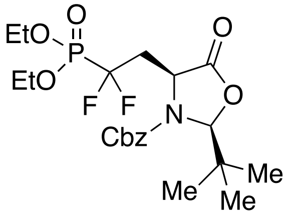

benzyl (2*S*,4*S*)-2-(*tert*-butyl)-4-(2-(diethoxyphosphoryl)-2,2-difluoroethyl)-5-oxooxazolidine-3-carboxylate (41):
Following the general procedure, the reaction of 2-bromo-2,2-diethyl (bromodifluoromethyl)phosphonate (89 μL, 0.5 mmol, 1 equiv), **32** (289 mg, 1.0 mmol, 2 equiv), [Ir(ppy)$_2$(dtbbpy)]PF$_6$ (5 mg, 0.005 mmol, 0.01 equiv) and Hantzsch ester (164 mg, 0.65 mmol, 1.3 equiv) provided the product (222 mg, 93% yield) as a colorless oil after purification by flash column chromatography (10% – 50% ethyl acetate/hexanes).

^{1}H NMR (500 MHz, CDCl$_3$) δ 7.37 – 7.29 (m, 5H), 5.59 (s, 1H), 5.24 – 5.11 (m, 2H), 4.84 (t, *J* = 5.9 Hz, 1H), 4.24 (tddd, *J* = 12.4, 6.8, 5.2, 2.7 Hz, 4H), 2.77 – 2.48 (m, 2H), 1.35 (td, *J* = 7.0, 1.0 Hz, 6H), 0.95 (s, 9H).
^{13}C NMR (150 MHz, CDCl$_3$) δ 171.1, 155.6, 135.1, 128.6, 128.5, 121.0, 119.6, 119.3, 117.9, 117.6, 116.1, 96.8, 68.5, 64.9, 64.8, 64.8, 64.8, 51.1, 51.1, 51.0, 51.0, 37.3, 37.1, 37.0, 36.9, 24.8, 16.4, 16.3.
^{19}F NMR (282 MHz, CDCl$_3$) δ -113.20 (dddd, *J* = 299.8, 103.3, 32.6, 7.5 Hz).
^{31}P NMR (243 MHz, CDCl$_3$) δ 5.81 (tt, *J* = 104.4, 7.6 Hz).
FTIR (neat) ν_{max}: 2976, 2875, 1796, 1720, 1482, 1392, 1291, 1270, 1236, 1197, 1177, 1105, 1010, 977, 791, 732, and 698 cm^{-1}.
HRMS (NSI) *m/z*: [M+H]$^+$ calcd. for $C_{21}H_{31}O_7NF_2P$, 478.1801; found, 478.1802.

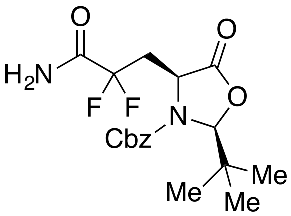

benzyl (2*S*,4*S*)-4-(3-amino-2,2-difluoro-3-oxopropyl)-2-(*tert*-butyl)-5-oxooxazolidine-3-carboxylate (42):

Following the general procedure, the reaction of 2-bromo-2,2-difluoroacetamide (43 mg, 0.25 mmol, 1 equiv), **32** (145 mg, 0.50 mmol, 2 equiv), [Ir(ppy)$_2$(dtbbpy)]PF$_6$ (2 mg, 0.0025 mmol, 0.01 equiv) and Hantzsch ester (82 mg, 0.33 mmol, 1.3 equiv) provided the product (58 mg, 60% yield) as a colorless oil after purification by flash column chromatography (5% – 30% ethyl acetate/hexanes).

^{1}H NMR (500 MHz, CDCl$_3$) δ 7.40 – 7.31 (m, 5H), 6.40 (s, 1H), 6.13 (s, 1H), 5.58 (s, 1H), 5.26 – 5.08 (m, 2H), 4.70 (dd, *J* = 7.5, 5.9 Hz, 1H), 2.82 – 2.60 (m, 2H), 0.96 (s, 9H).
^{13}C NMR (125 MHz, CDCl$_3$) δ 171.0, 165.4 (t, *J* = 29.0 Hz), 155.9, 135.1, 128.8, 128.8, 128.7, 116.0 (t, *J* = 254.8 Hz), 97.1, 68.8, 52.3, 37.0, 36.7 (t, *J* = 24.6 Hz), 24.9.
^{19}F NMR (376 MHz, CDCl$_3$) δ -101.11 – -104.68 (m).
FTIR (neat) ν_{max}: 3446, 3354, 3198, 2964, 2875, 1793, 1719, 1607, 1394, 1319, 1196, 1037, 1014, 909. 731, and 698 cm^{-1}.
HRMS (NSI) *m/z*: [M+H]$^+$ calcd. for $C_{18}H_{23}O_5N_2F_2$, 385.1570; found, 385.1570.

VI. Deprotection Procedures and Characterization Data

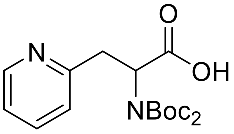

methyl 2-(di(*tert*-butoxycarbonyl)amino)-3-(pyridin-2-yl)propanoic acid (2A):

To a stirring solution of methyl 2-(di(*tert*-butoxycarbonyl)amino)-3-(pyridin-2-yl)propanoate (**2**) (190 mg, 0.5 mmol, 1 equiv) in THF/H_2O (3:2, 5 mL) was added LiOH (24 mg, 1.0 mmol, 2.0 equiv). The resultant solution was stirred until consumption of starting material was observed by thin layer chromatography. The reaction mixture was extracted once with ethyl acetate, and the organic extract was discarded. The aqueous phase was gently acidified with 0.1 M HCl to pH 4 and extracted with ethyl acetate (5 x 5 mL). The combined extracts were dried over sodium sulfate, filtered, and concentrated to provide the product (172 mg, 94% yield) as a clear, colorless crystalline solid.

Mp: 164 °C (decomp.)
^{1}H NMR (300 MHz, CDCl$_3$) *δ* 8.53 (dt, *J* = 5.0, 1.4 Hz, 1H), 7.75 (td, *J* = 7.7, 1.8 Hz, 1H), 7.41 – 7.12 (m, 2H), 5.21 (dd, *J* = 7.9, 4.7 Hz, 1H), 3.93 (dd, *J* = 15.3, 7.9 Hz, 1H), 3.17 (dd, *J* = 15.3, 4.7 Hz, 1H), 1.45 (s, 18H).
^{13}C NMR (75 MHz, CDCl$_3$) *δ* 171.6, 158.0, 152.0, 147.1, 138.6, 124.4, 122.4, 83.2, 58.2, 39.2, 27.9.
FTIR (neat) ν_{max}: 3456, 3068, 2970, 2930, 2853, 1748, 1708, 1366, 1108, 1062, and 778 cm^{-1}.

HRMS (NSI) *m/z*: $[M+H]^+$ calcd. for $C_{18}H_{27}O_6N_2$, 367.1864; found, 367.1859.

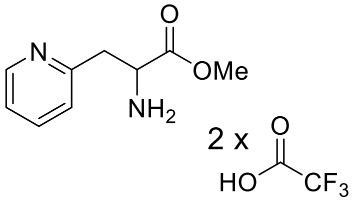

2-(2-ammonio-3-methoxy-3-oxopropyl)pyridin-1-ium ditrifluoroacetate (2B):
To a stirring solution of of methyl 2-(di(*tert*-butoxycarbonyl)amino)-3-(pyridin-2-yl)propanoate (**2**) (190 mg, 0.5 mmol, 1 equiv) in dichloromethane (2 mL) was added trifluoroacetic acid (1.5 mL) dropwise. The resultant solution was allowed to continue stirring, and after 10 minutes consumption of starting material was observed by thin layer chromatography. The reaction mixture was concentrated directly by rotary evaporation. The solution was re-dissolved in dichloromethane and concentrated once more to quantitatively provide the product as a pale, yellow low-melting solid (203 mg, >99% yield).

^{1}H NMR (300 MHz, D_3COD) δ 8.78 (dd, J = 5.7, 1.6 Hz, 1H), 8.44 (td, J = 7.9, 1.7 Hz, 1H), 7.96 (d, J = 8.0 Hz, 1H), 7.89 (td, J = 5.9, 2.8 Hz, 1H), 5.73 (s, 4H), 4.65 (t, J = 7.1 Hz, 1H), 3.77 (s, 3H), 3.69 (t, J = 7.2 Hz, 2H).
^{13}C NMR (75 MHz, D_3COD) δ 167.8, 159.8 (q, J = 37.9 Hz), 151.4, 144.9, 143.1, 127.4, 125.3, 117.6 (q, J = 289.1 Hz), 52.6, 51.5, 33.6, 26.2.
^{19}F NMR (282 MHz, D_3COD) δ -77.45.
FTIR (neat) ν_{max}: 2964, 2563, 2111, 1746, 1666, 1651, 1172, 1157, 1127, 836, 798, and 720 cm^{-1}.
HRMS (NSI) *m/z*: $[M+H]^+$ calcd. for $C_9H_{13}O_2N_2$, 181.0972; found, 181.0967.

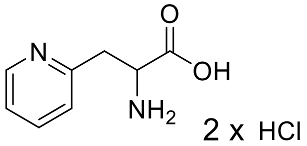

2-amino-3-(pyridin-2-yl)propanoic acid dihydrochloride (2C):
A 20-mL scintillation vial equipped with stir bar was charged with methyl 2-(di(*tert*-butoxycarbonyl)amino)-3-(pyridin-2-yl)propanoate (**2**) (190 mg, 0.5 mmol, 1 equiv), EtOH (5 mL) and 3N NaOH (5 mL), and the resultant solution was stirred until consumption of starting material was observed (~ 1 hour). The reaction mixture was acidified with 1M HCl and concentrated by rotary evaporation. The residue was reconstituted in EtOH, and precipitated NaCl was removed by vacuum filtration. The filtrate was concentrated under reduced pressure. Concentrated HCl (3 mL) was added dropwise to the residue and stirred 10 minutes. The mixture was concentrated directly to provide the product (119 mg, 96% yield) as a white crystalline solid.

^{1}H NMR (300 MHz, D_2O) δ 8.49 (d, J = 6.0 Hz, 1H), 8.32 (td, J = 8.0, 1.7 Hz, 1H), 7.83 (d, J = 8.1 Hz, 1H), 7.75 (dd, J = 7.7, 6.1 Hz, 1H), 4.34 (t, J = 7.4 Hz, 1H), 3.47 (d, J = 7.4 Hz, 2H).
^{13}C NMR (75 MHz, D_2O) δ 169.6, 149.6, 147.2, 141.4, 128.2, 126.1, 51.6, 33.2.

FTIR (neat) ν_{max}: 3399, 2780, 2934, 1702, 1567, 1477, 1403, 1367, 1243, 1139, 1102, 1101, 921, and 750 cm^{-1}.
HRMS (NSI) *m/z*: $[M+H]^+$ calcd. for $C_8H_{11}O_2N_2$, 167.0815; found, 167.0811.

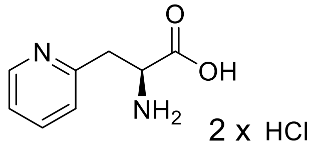

(*S*)-2-amino-3-(pyridin-2-yl)propanoic acid dihydrochloride (37):
To a round bottom flask equipped with a stir bar was added **23** (20.2 mg), and concentrated aqueous HCl (2 mL). The reaction was stirred at 80 °C for 30 minutes then concentrated by rotary evaporation to afford the product (12.8, 98%) as a white solid. The physical properties and spectral data are consistent with the values of the racemate (**2C**) reported herein, with the exception of optical rotation.
$[\alpha]_D^{20}$ +31.7 (*c* 0.1, 1 M HCl) (lit.,[8] +46.0 (*c* 0.1, 1 M HCl))

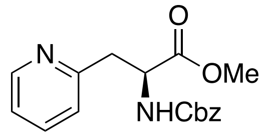

methyl (*S*)-2-(((benzyloxy)carbonyl)amino)-3-(pyridin-2-yl)propanoate (37A):
To a round bottom flask equipped with a stir bar was added **24**, Et_2O (5 mL), and MeOH (5 mL). The reaction was placed under nitrogen atmosphere then cooled to 0 °C. (Trimethylsilyl)diazomethane solution (2.0 M in ether, 80 μL, 0.16 mmol, 2.0 equiv) was added dropwise via syringe and the reaction was warmed to room temperature and stirred for 30 minutes. The reaction was quenched with AcOH (2 mL) then concentrated by rotary evaporation. The residue was dissolved in saturated aqueous sodium bicarbonate (1 mL) and THF (1 mL). The solution was set to stir and chilled to 0 °C. Benzyl chloroformate (12.5 μL, 0.09 mmol, 1.1 equiv) was added dropwise via syringe, and the reaction was warmed to room temperature and stirred until HPLC indicated the starting material had been consumed. The reaction was concentrated to remove THF then diluted with water (1 mL). The aqueous solution was extracted with EtOAc (3 x 2 mL), and the combined extracts were concentrated by rotary evaporation. The residue was purified by preparative HPLC to afford the product as a colorless oil. Chiral HPLC analysis (OD-H, 15% IPA/hexanes, 1.0 mL/min, 254 nm) indicated 97% ee for the major enantiomer (t_R (major) = 14.949 min, t_R (minor) = 21.299 min).

^{1}H NMR (500 MHz, $CDCl_3$) δ 8.47 (d, *J* = 4.7 Hz, 1H), 7.60 – 7.56 (m, 1H), 7.39 – 7.27 (m, 5H), 7.15 – 7.09 (m, 2H), 6.30 (d, *J* = 8.2 Hz, 1H), 5.15 – 5.05 (m, 2H), 4.76 (dt, *J* = 8.4, 5.3 Hz, 1H), 3.68 (s, 3H), 3.36 (dd, *J* = 14.9, 5.7 Hz 1H), 3.28 (dd, *J* = 14.9, 4.7 Hz 1H).

---

[8] Anaïs F. M. Noisier, Craig S. Harris, and Margaret A. Brimble *Chem. Commun.*, **2013**, *49*, 7744.

^{13}C NMR (125 MHz, $CDCl_3$) δ 172.1, 157.1, 156.1, 149.3, 136.7, 136.5, 128.5, 128.2, 128.2, 123.8, 121.9, 67.0, 53.4, 52.4, 39.0.
FTIR (neat) ν_{max}: 3333, 3032, 2951, 1716, 1593, 1507, 1436, 1340, 1210, 1050, 911, 752, and 670 cm^{-1}.
HRMS (NSI) *m/z*: $[M+H]^+$ calcd. for $C_{17}H_{19}O_4N_2$, 315.1339; found, 315.1335.

VII. ^{1}H and ^{13}C NMR Spectra

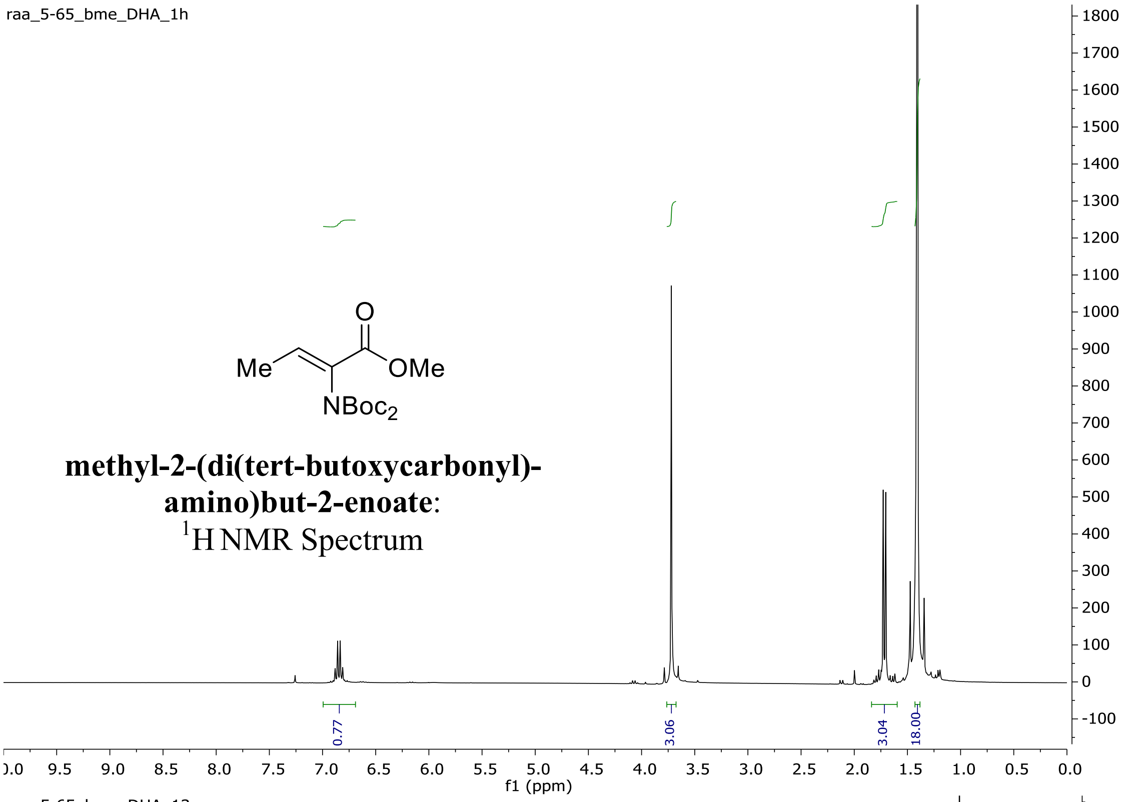

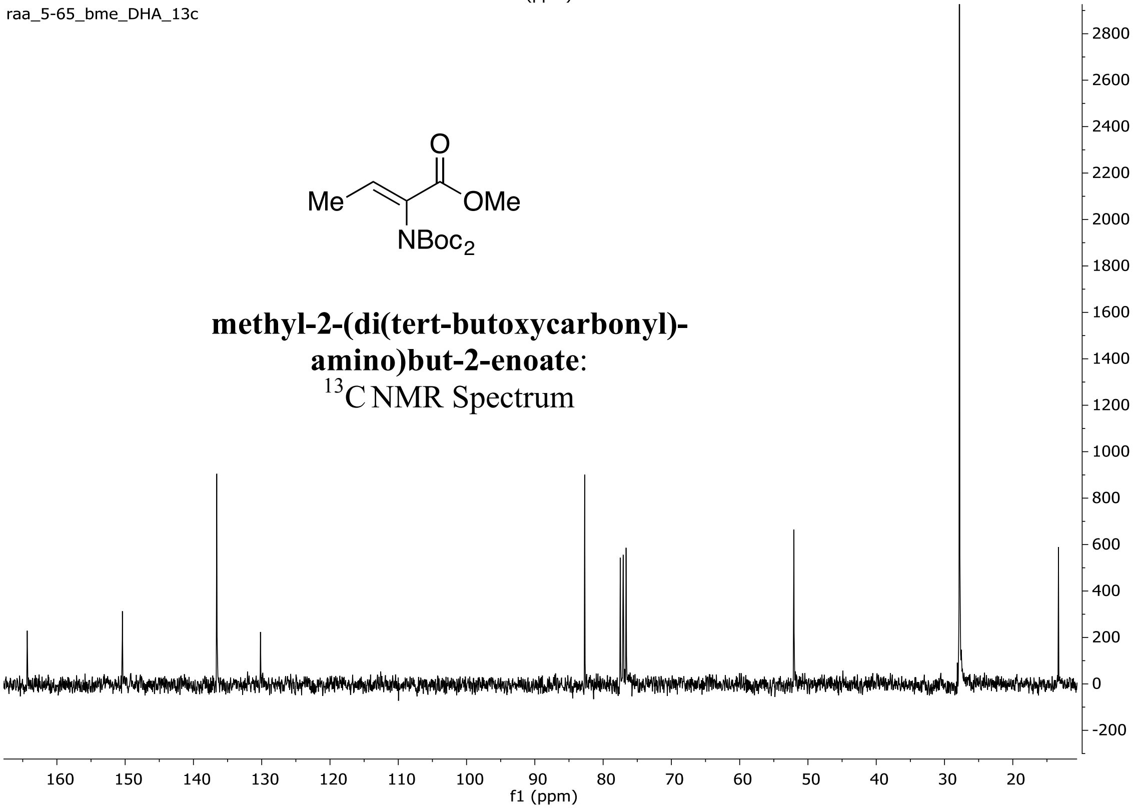

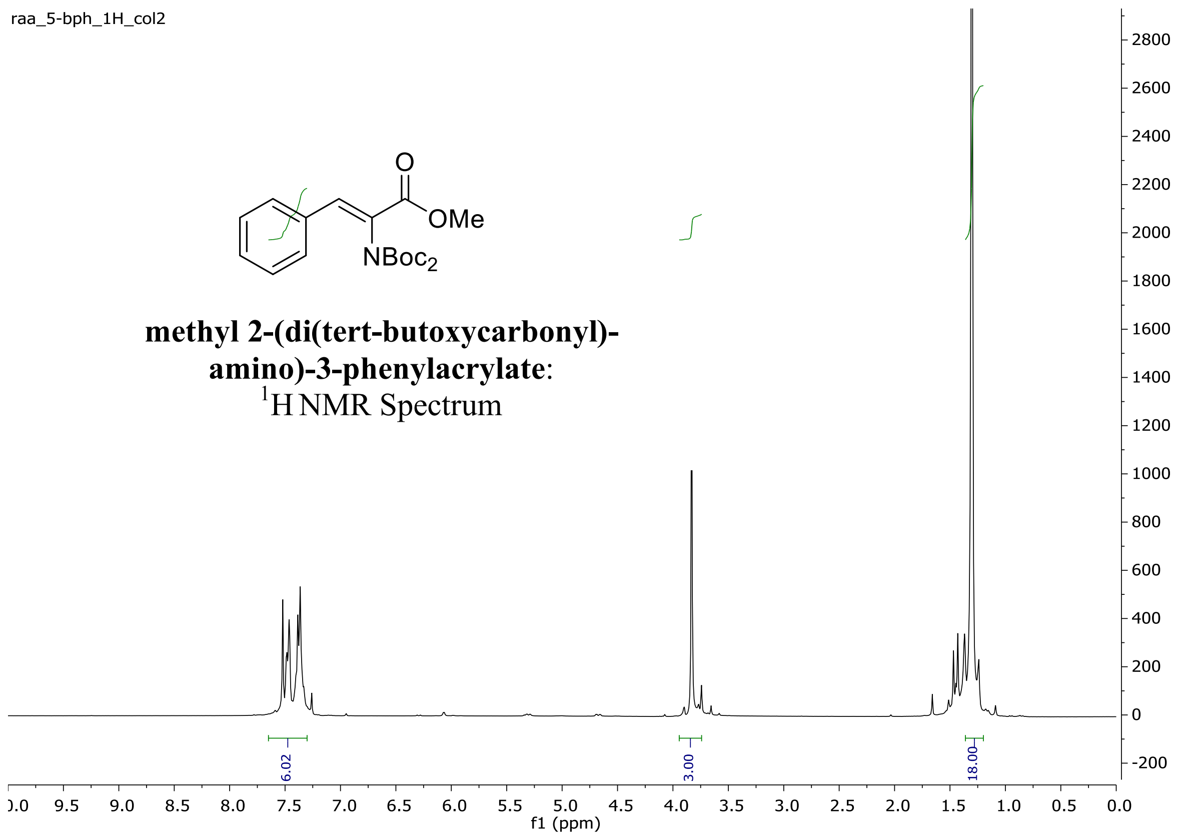
raa_5-bph_1H_col2
O
OMe
NBoc2
methyl 2-(di(tert-butoxycarbonyl)-amino)-3-phenylacrylate:
1H NMR Spectrum
6.02
3.00
18.00
f1 (ppm)

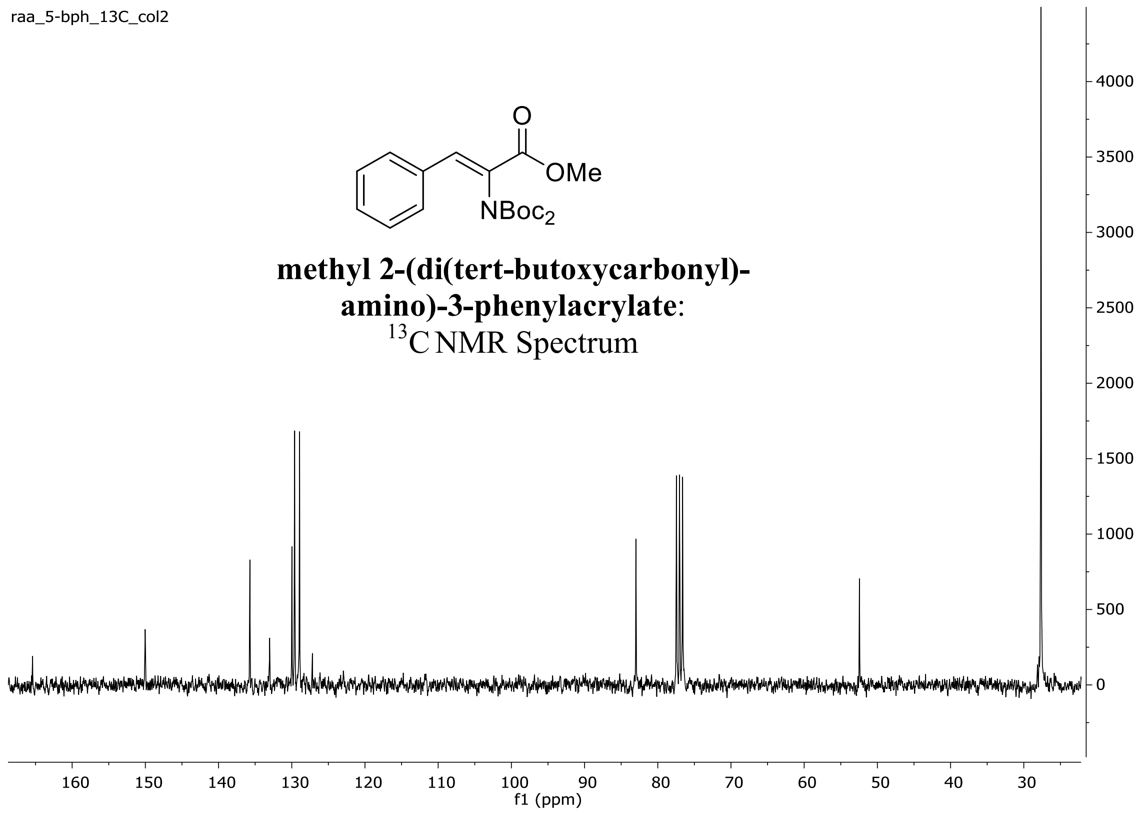
raa_5-bph_13C_col2
O
OMe
NBoc2
methyl 2-(di(tert-butoxycarbonyl)-amino)-3-phenylacrylate:
13C NMR Spectrum
f1 (ppm)

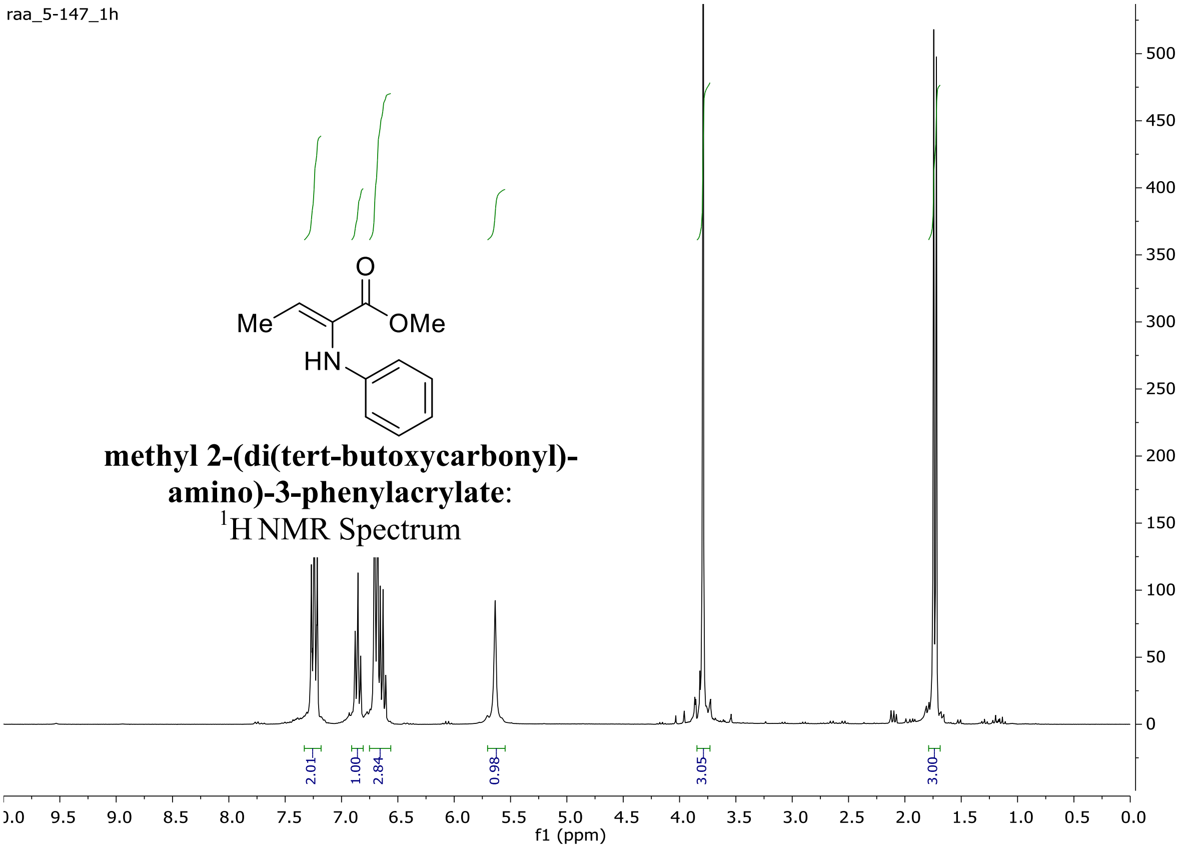
raa_5-147_1h
Me
O
OMe
HN
methyl 2-(di(tert-butoxycarbonyl)-amino)-3-phenylacrylate:
1H NMR Spectrum
2.01
1.00
2.84
0.98
3.05
3.00
f1 (ppm)

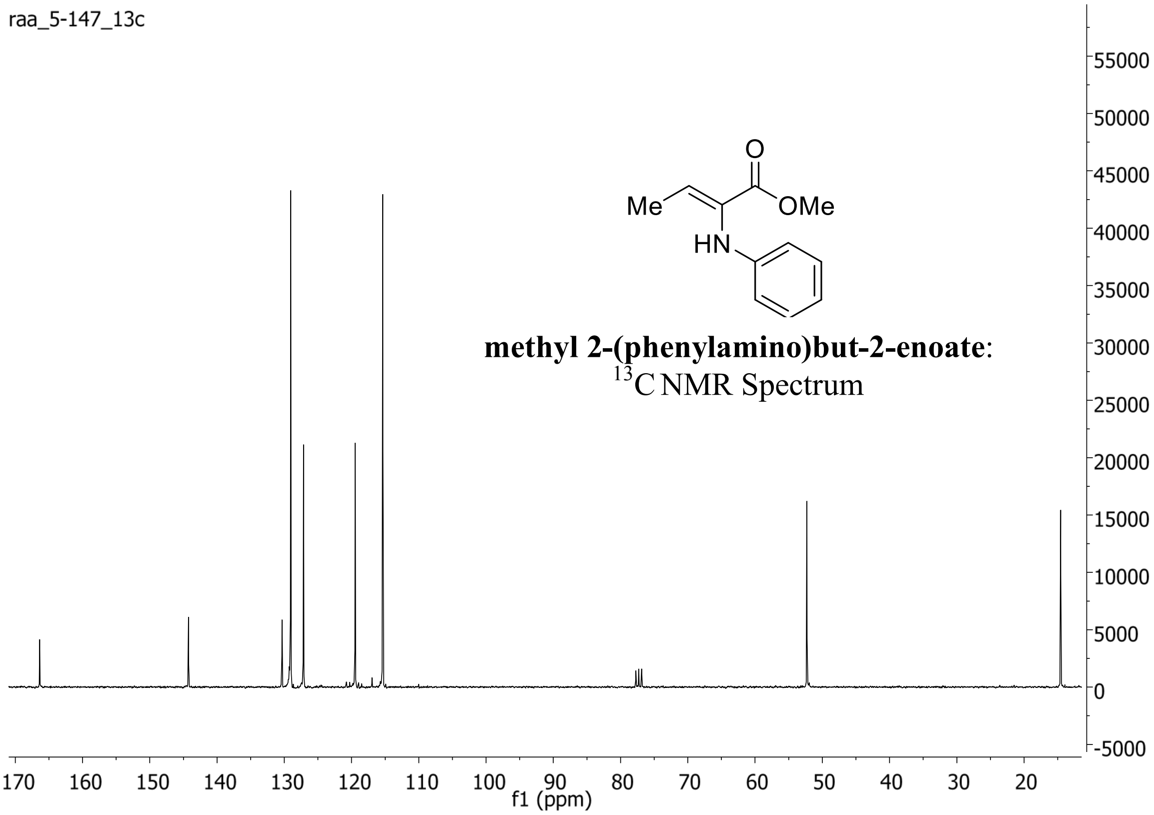
raa_5-147_13c
Me
O
OMe
HN
methyl 2-(phenylamino)but-2-enoate:
13C NMR Spectrum
f1 (ppm)

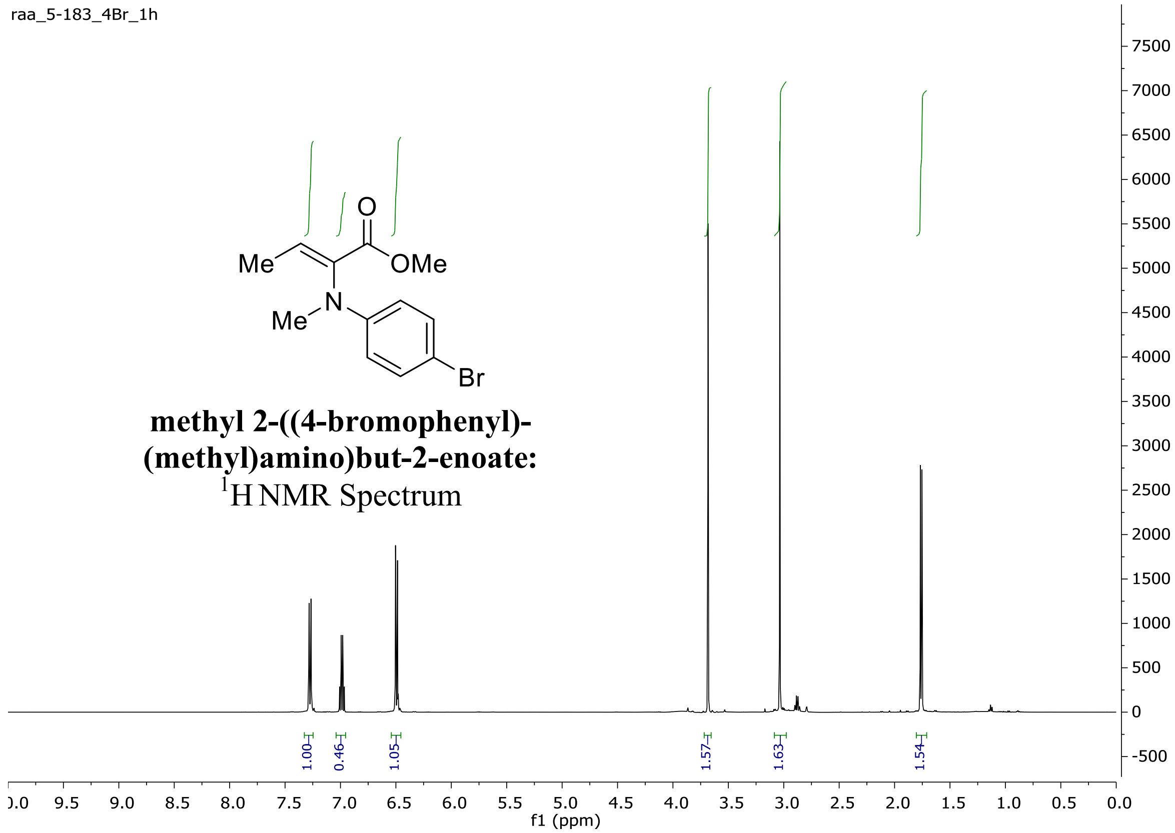
raa_5-183_4Br_1h
Me
O
OMe
N
Me
Br
methyl 2-((4-bromophenyl)-
(methyl)amino)but-2-enoate:
1H NMR Spectrum
1.00
0.46
1.05
1.57
1.63
1.54
f1 (ppm)

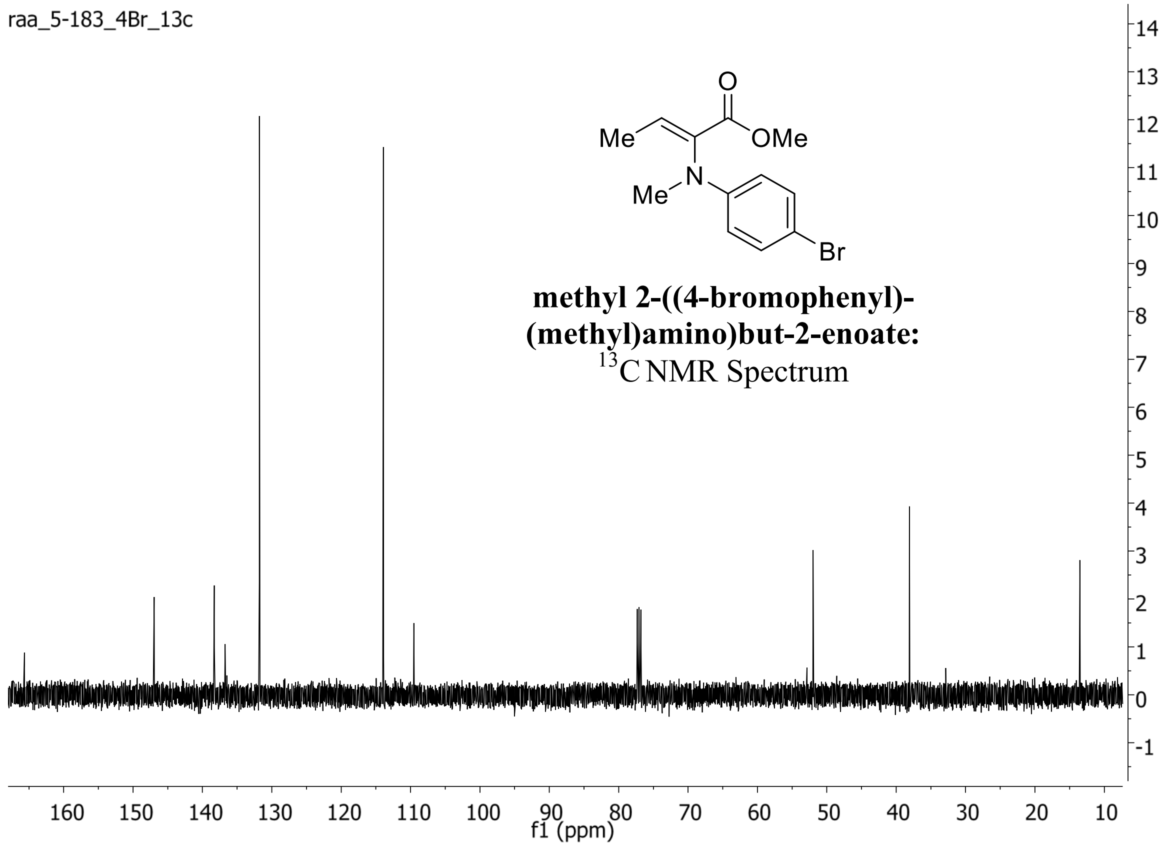
raa_5-183_4Br_13c
Me
O
OMe
N
Me
Br
methyl 2-((4-bromophenyl)-
(methyl)amino)but-2-enoate:
13C NMR Spectrum
f1 (ppm)

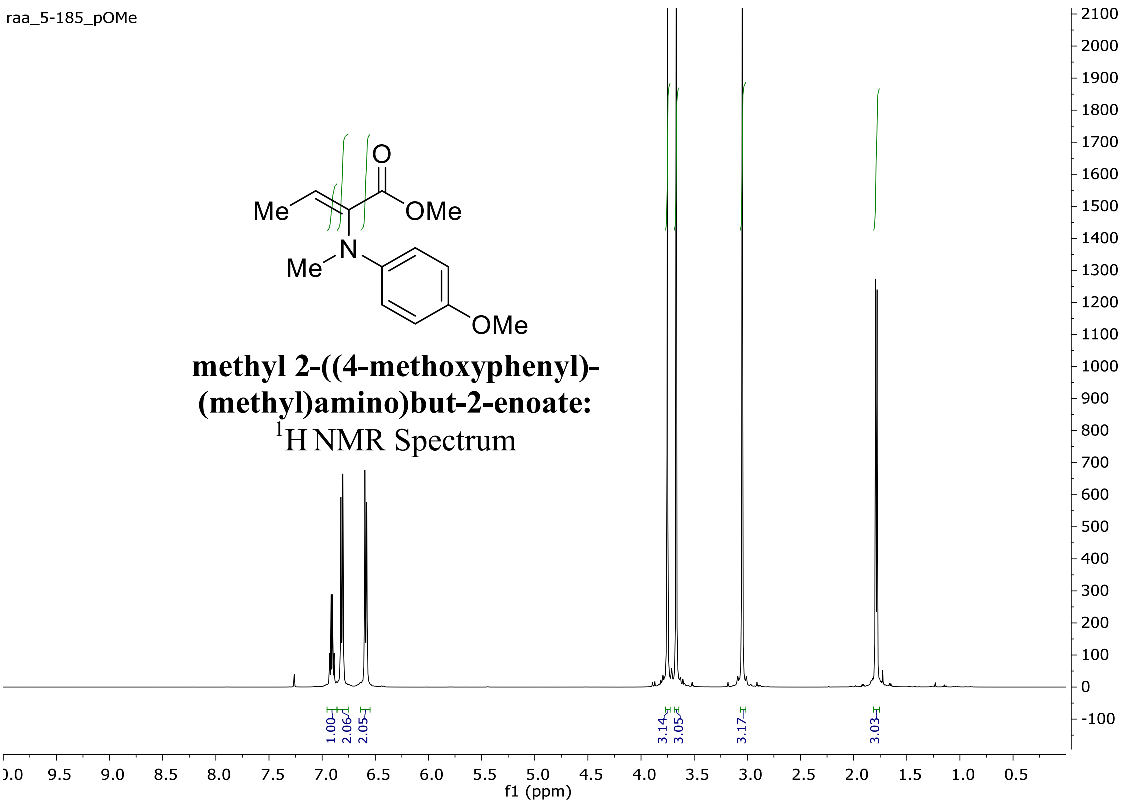
raa_5-185_pOMe
Me
O
OMe
N
Me
OMe
methyl 2-((4-methoxyphenyl)-
(methyl)amino)but-2-enoate:
1H NMR Spectrum
1.00
2.06
2.05
3.14
3.05
3.17
3.03
f1 (ppm)

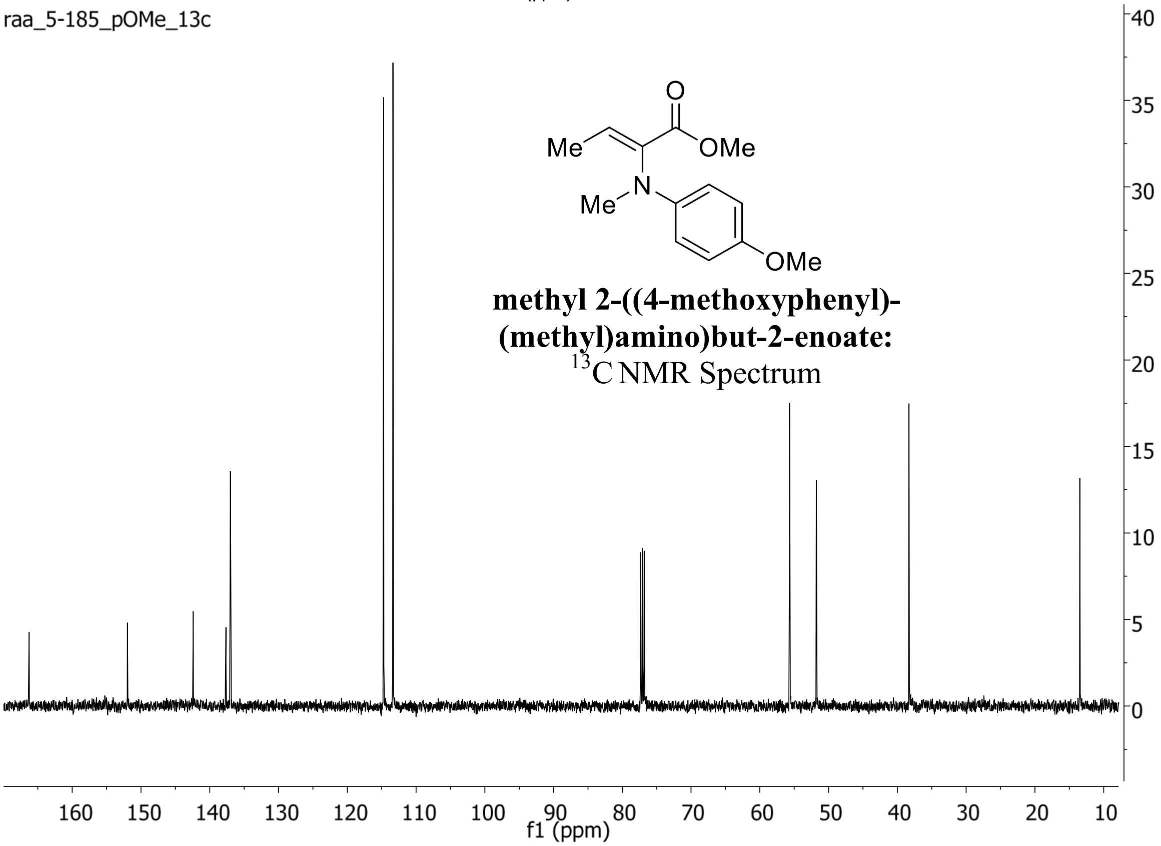
raa_5-185_pOMe_13c
Me
O
OMe
N
Me
OMe
methyl 2-((4-methoxyphenyl)-
(methyl)amino)but-2-enoate:
13C NMR Spectrum
f1 (ppm)

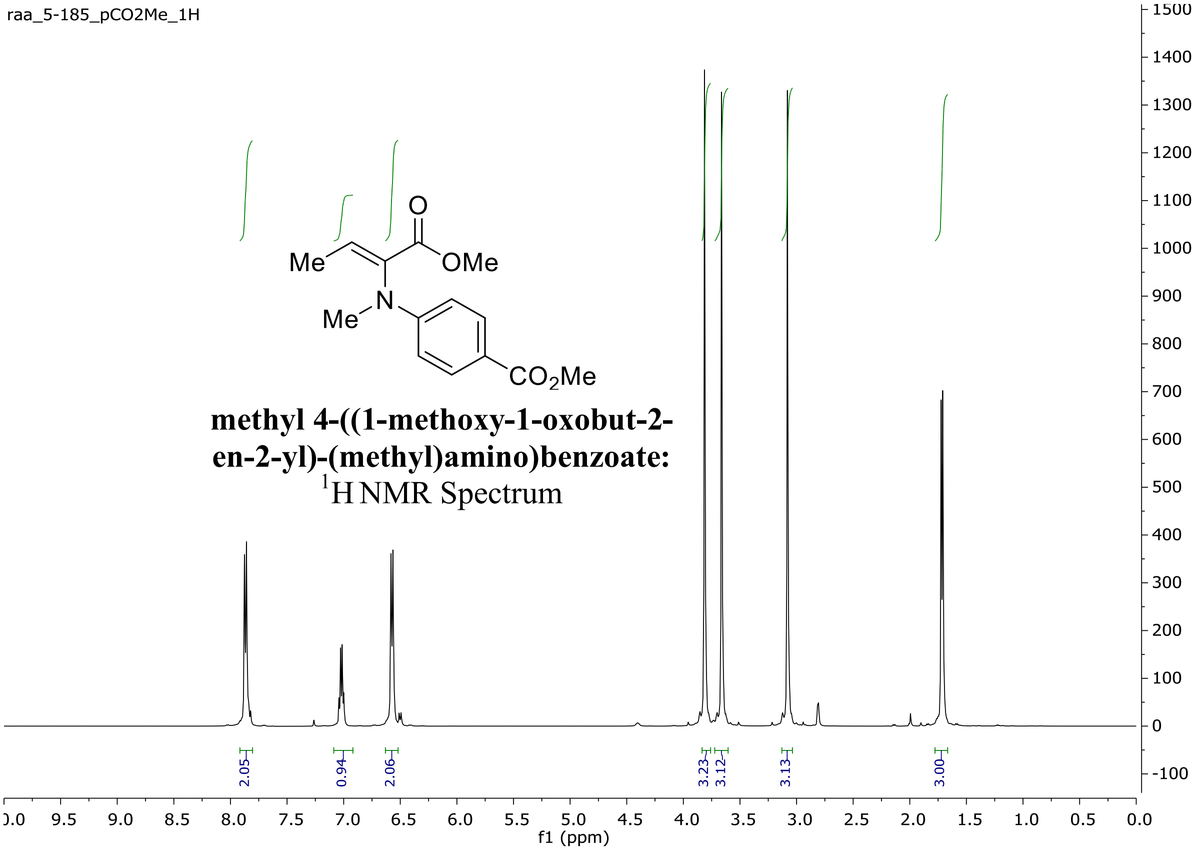
raa_5-185_pCO2Me_1H
methyl 4-((1-methoxy-1-oxobut-2-en-2-yl)-(methyl)amino)benzoate:
1H NMR Spectrum
Me
OMe
O
N
Me
CO2Me
2.05
0.94
2.06
3.23
3.12
3.13
3.00
f1 (ppm)

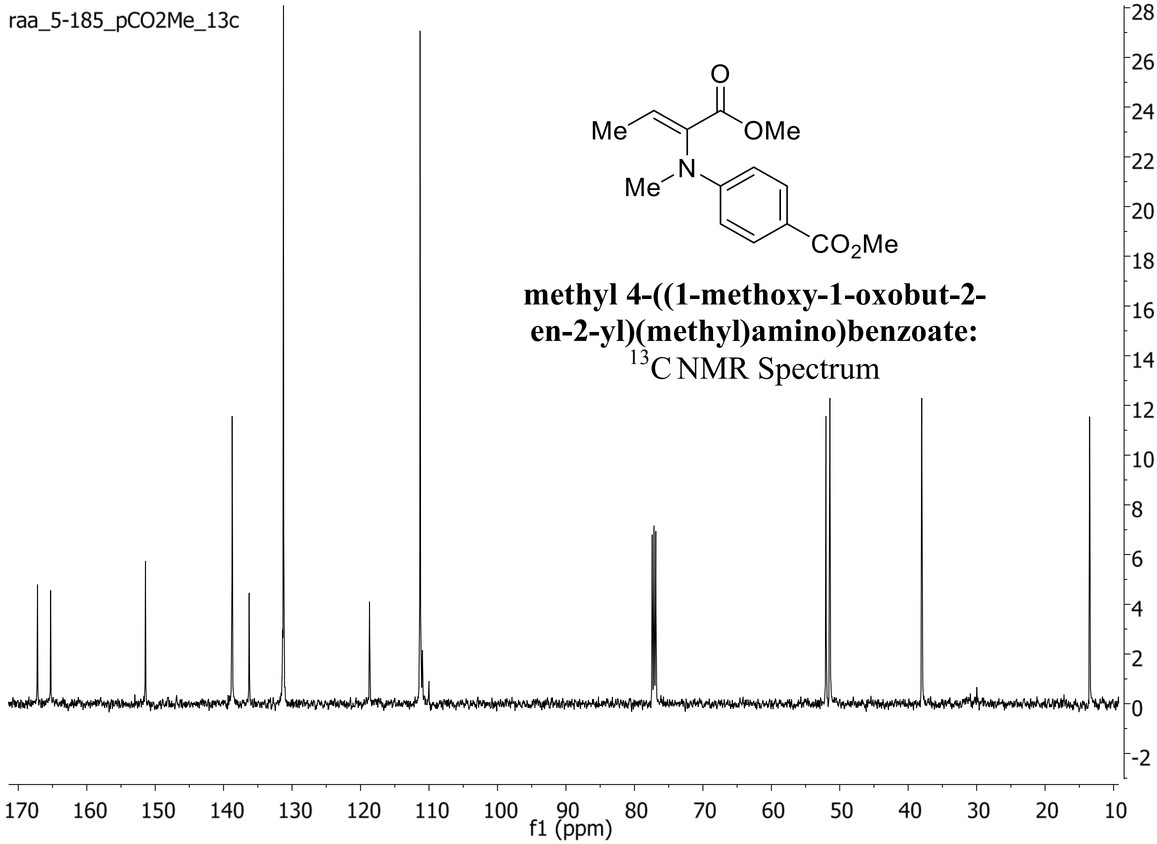
raa_5-185_pCO2Me_13c
methyl 4-((1-methoxy-1-oxobut-2-en-2-yl)(methyl)amino)benzoate:
13C NMR Spectrum
Me
OMe
O
N
Me
CO2Me
f1 (ppm)

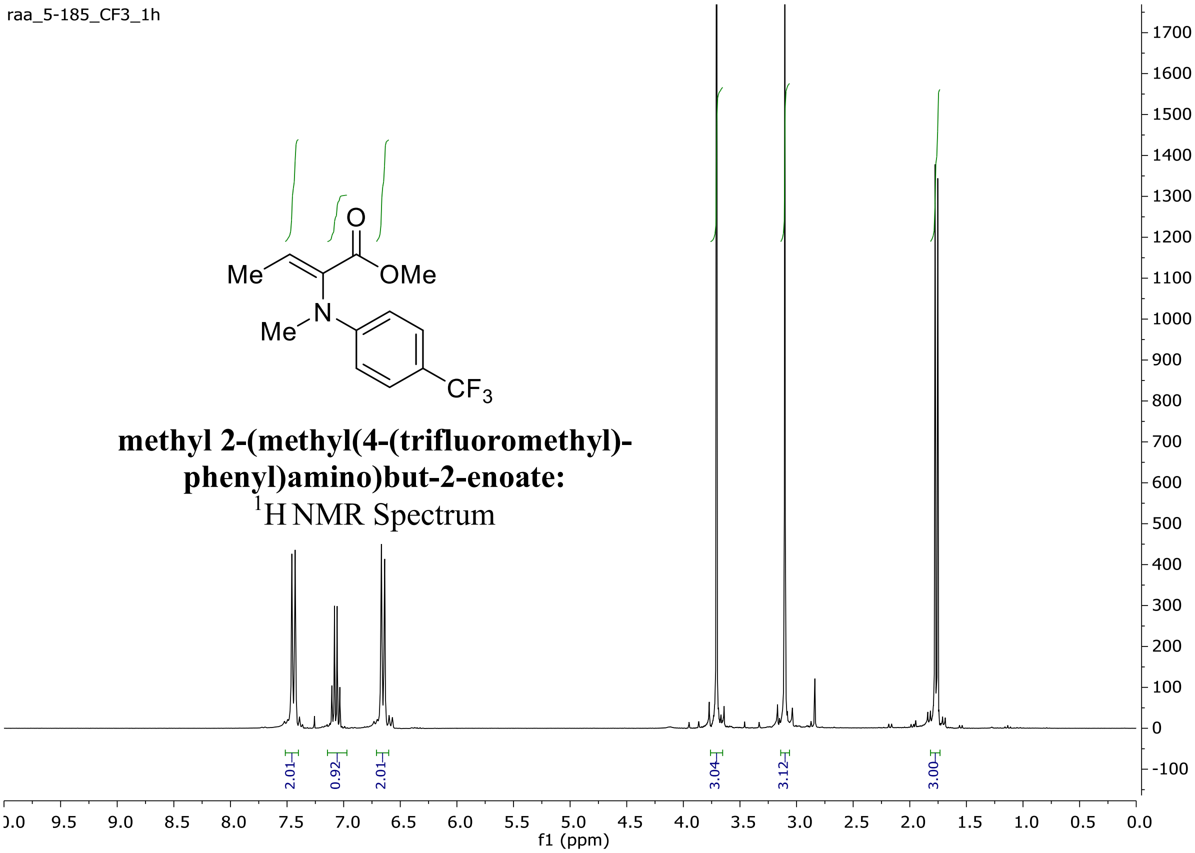
raa_5-185_CF3_1h
methyl 2-(methyl(4-(trifluoromethyl)-
phenyl)amino)but-2-enoate:
1H NMR Spectrum
Me
O
OMe
N
Me
CF3
2.01
0.92
2.01
3.04
3.12
3.00
f1 (ppm)

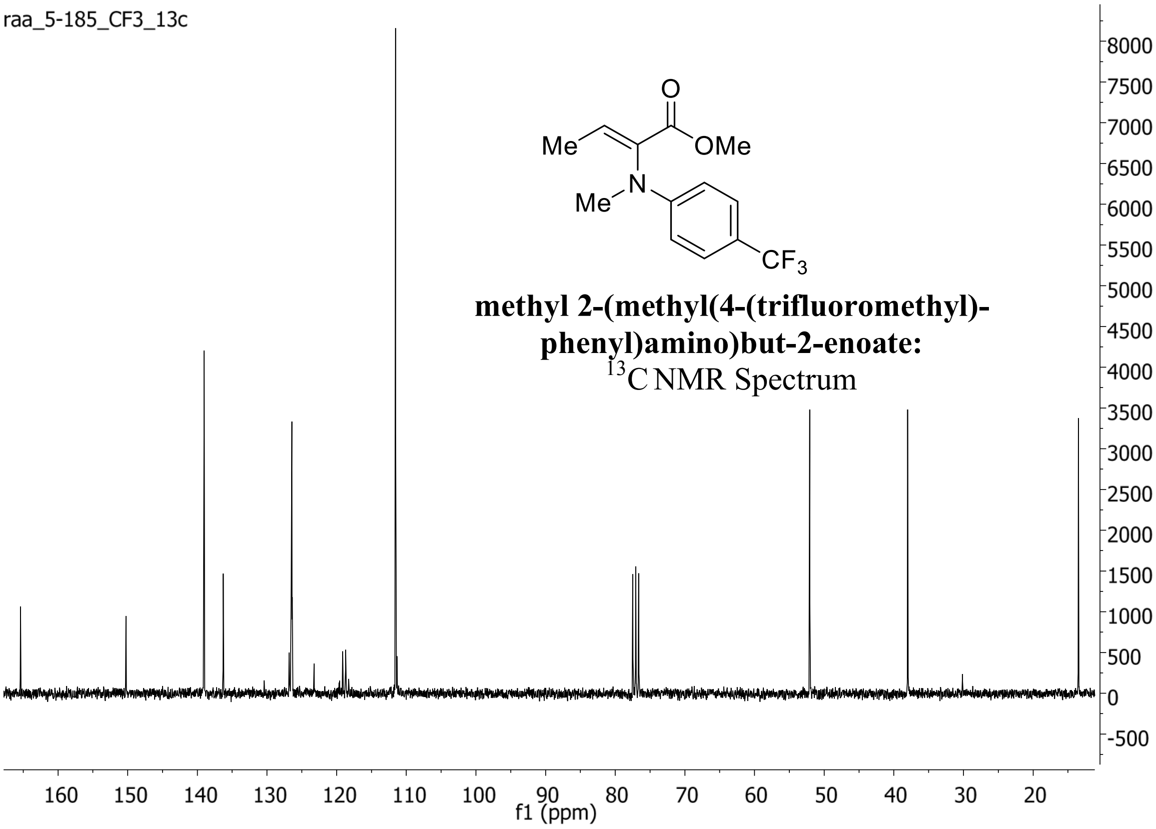
raa_5-185_CF3_13c
methyl 2-(methyl(4-(trifluoromethyl)-
phenyl)amino)but-2-enoate:
13C NMR Spectrum
Me
O
OMe
N
Me
CF3
f1 (ppm)

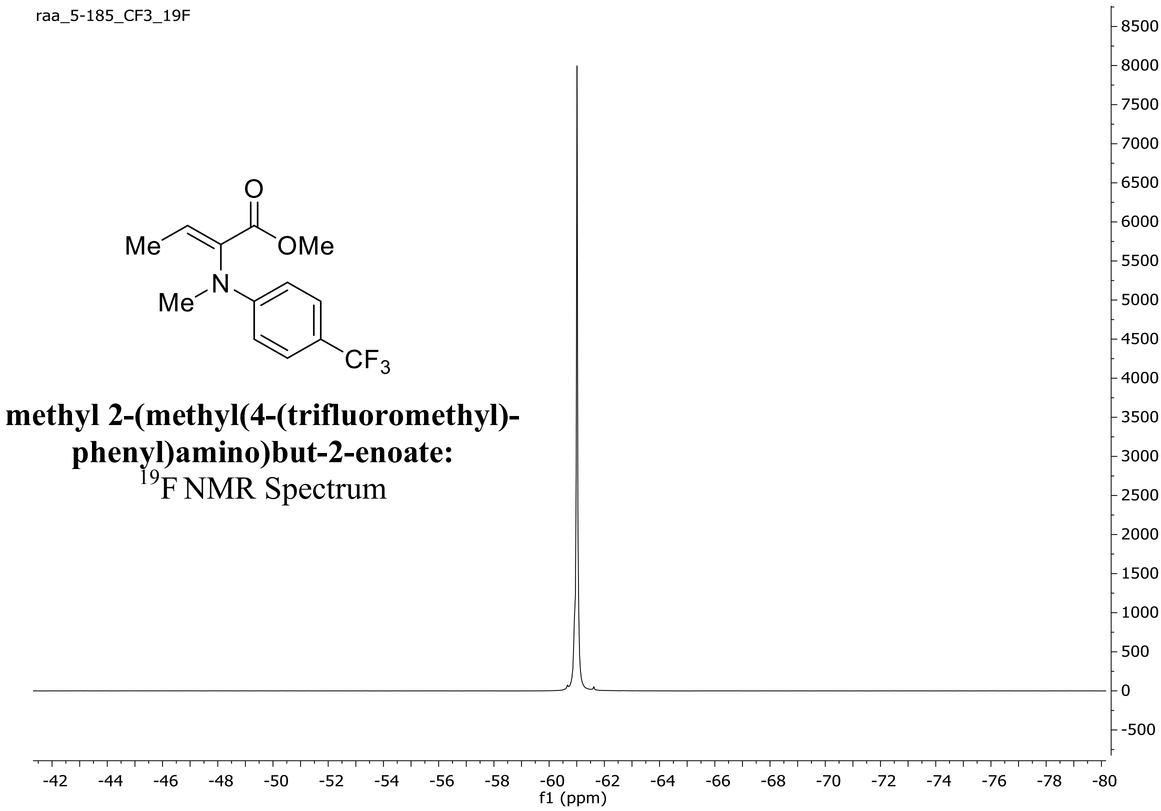

methyl 2-(methyl(4-(trifluoromethyl)-phenyl)amino)but-2-enoate:
^{19}F NMR Spectrum

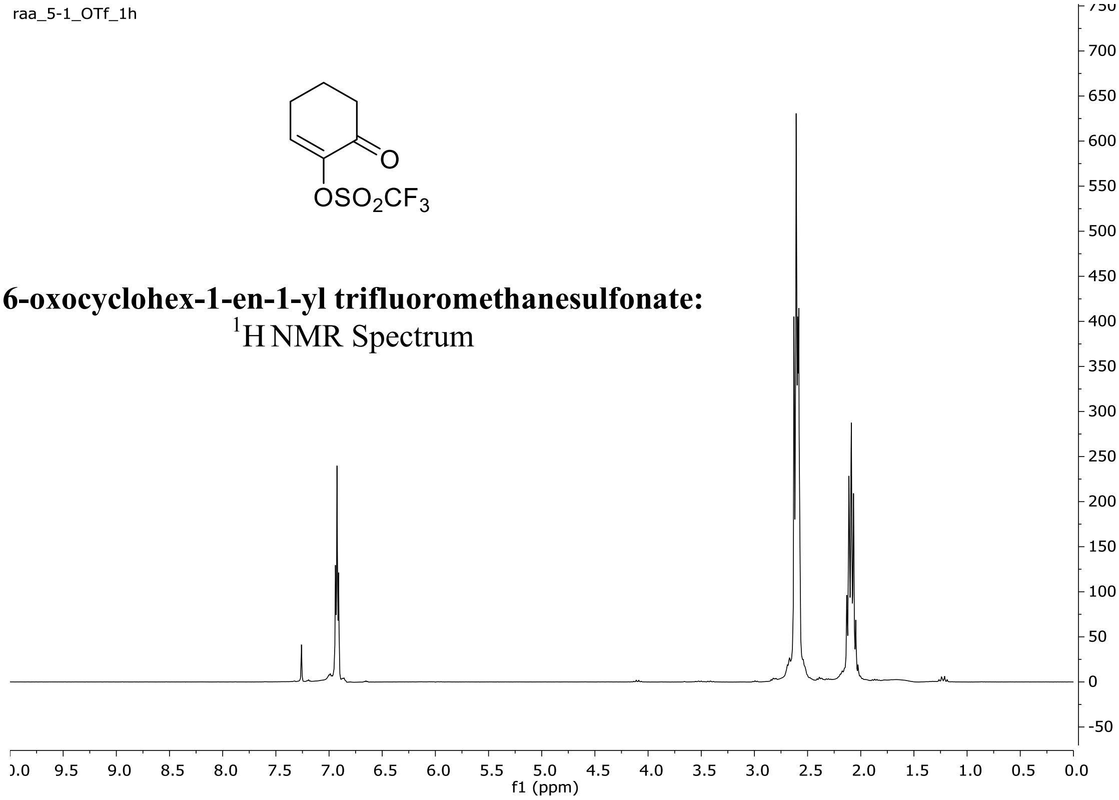

6-oxocyclohex-1-en-1-yl trifluoromethanesulfonate:
^{1}H NMR Spectrum

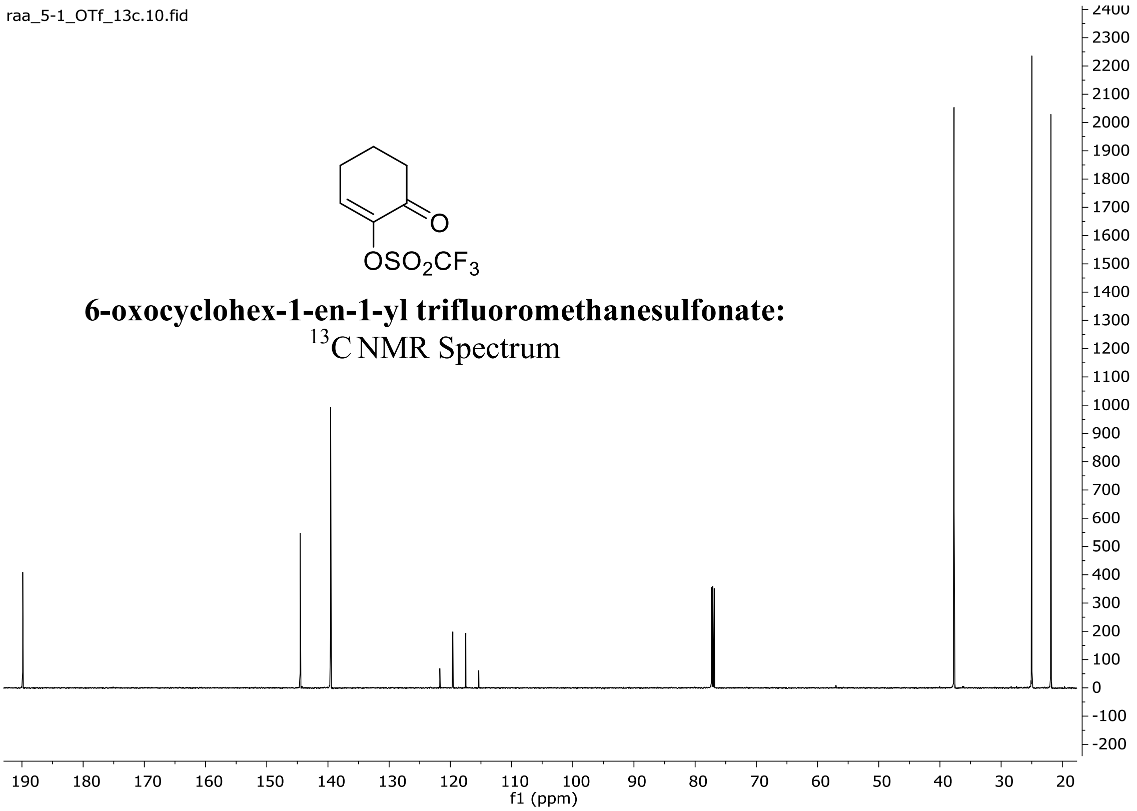
raa_5-1_OTf_13c.10.fid
O
OSO2CF3
6-oxocyclohex-1-en-1-yl trifluoromethanesulfonate:
13C NMR Spectrum
f1 (ppm)

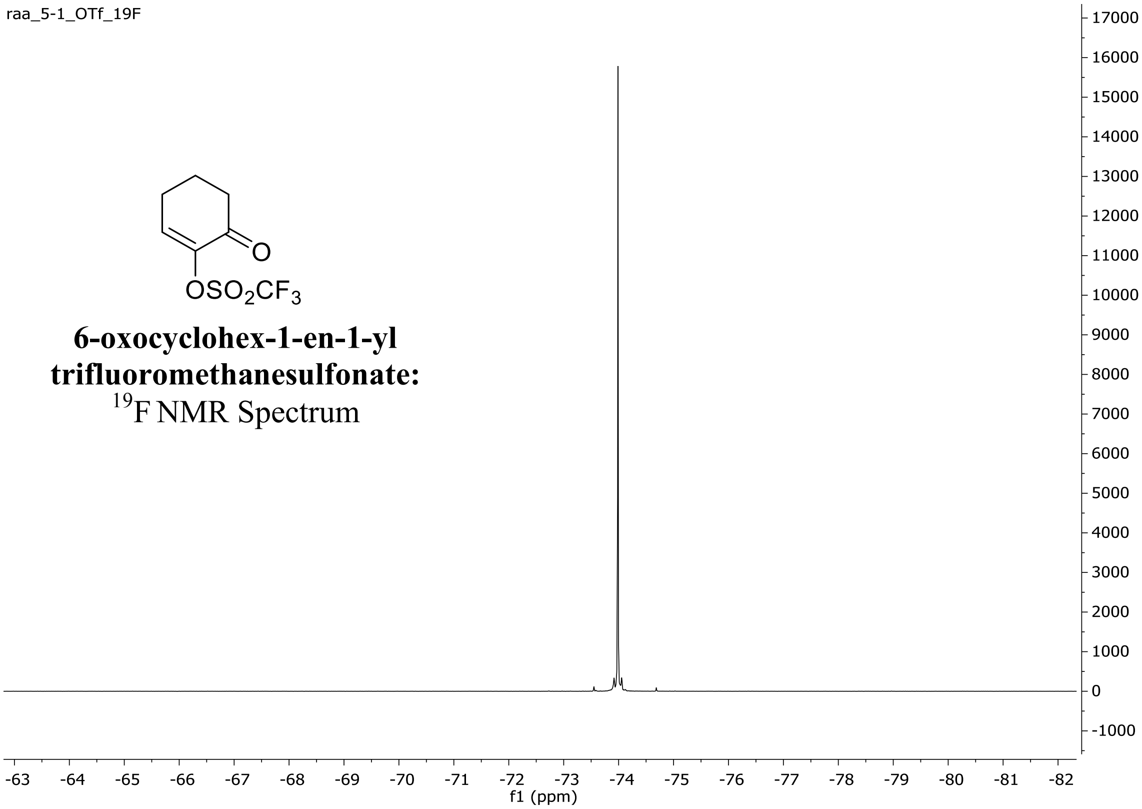
raa_5-1_OTf_19F
O
OSO2CF3
6-oxocyclohex-1-en-1-yl
trifluoromethanesulfonate:
19F NMR Spectrum
f1 (ppm)

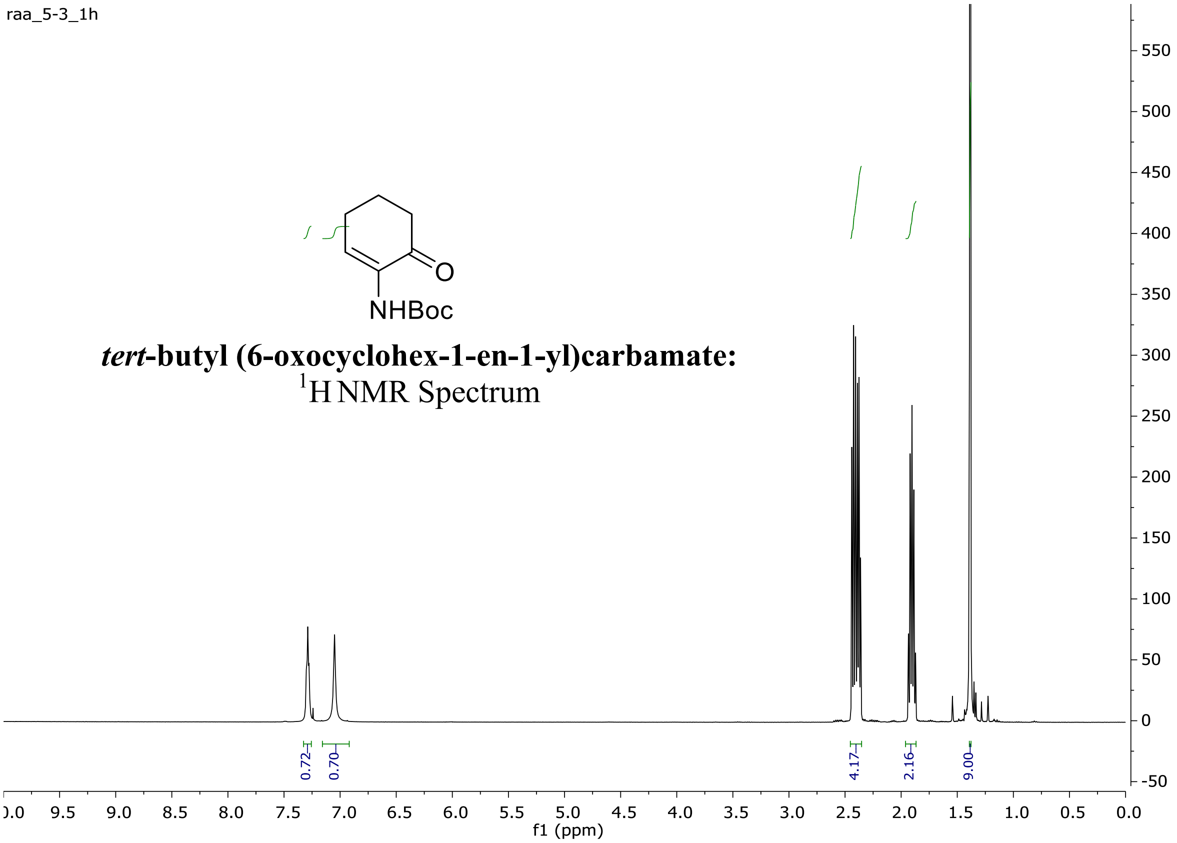
raa_5-3_1h
O
NHBoc
tert-butyl (6-oxocyclohex-1-en-1-yl)carbamate:
^{1}H NMR Spectrum
0.72
0.70
4.17
2.16
9.00
f1 (ppm)

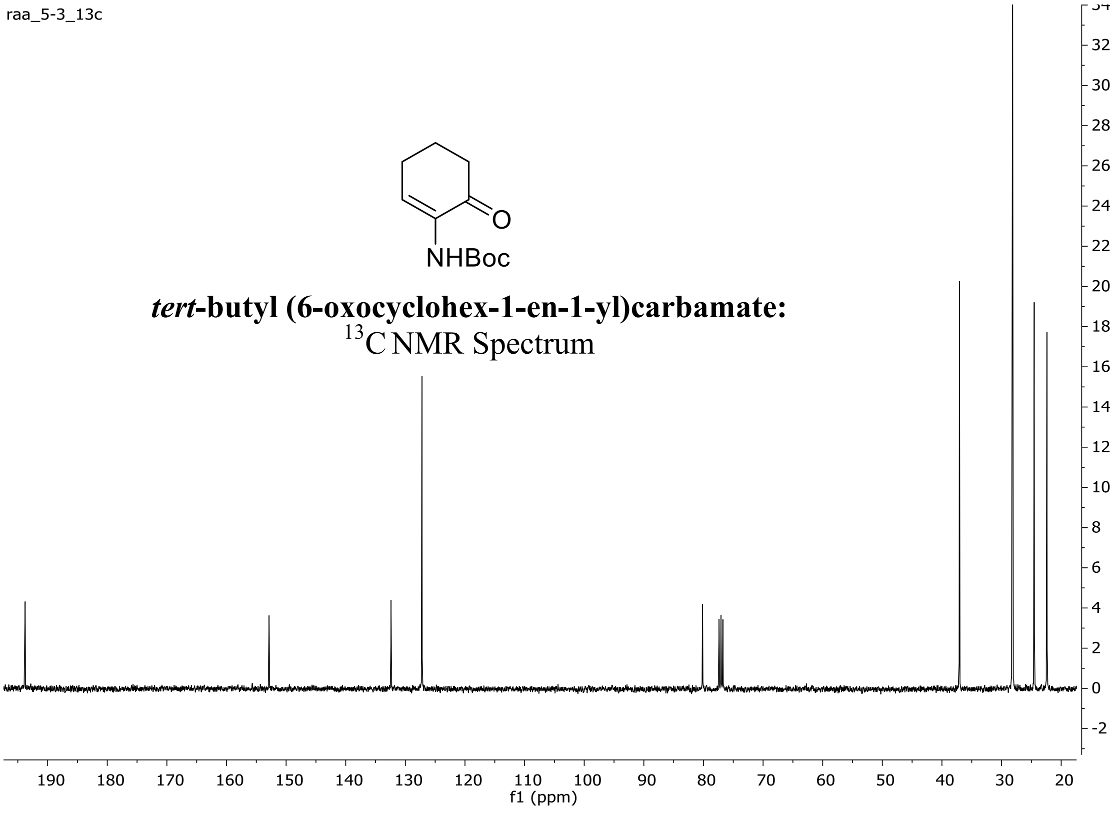
raa_5-3_13c
O
NHBoc
tert-butyl (6-oxocyclohex-1-en-1-yl)carbamate:
^{13}C NMR Spectrum
f1 (ppm)

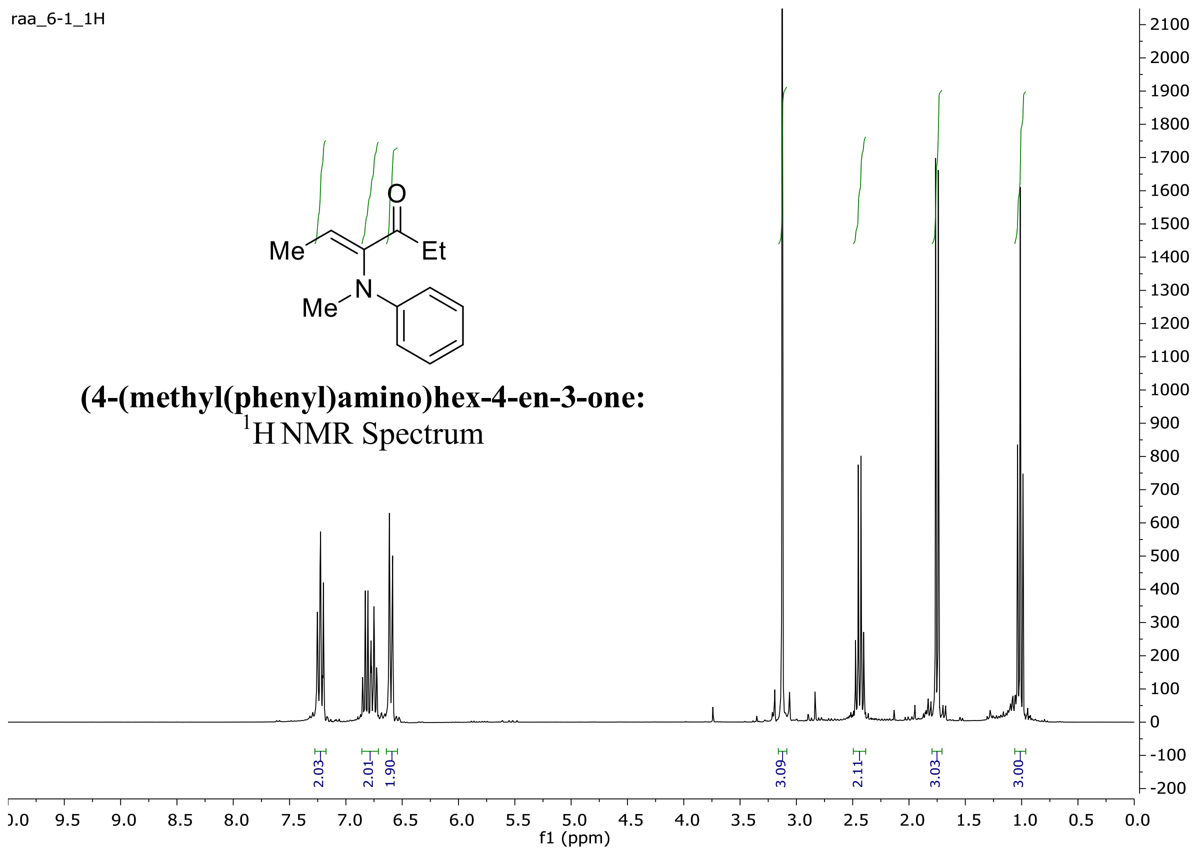

raa_6-1_1H
(4-(methyl(phenyl)amino)hex-4-en-3-one:
¹H NMR Spectrum
f1 (ppm)

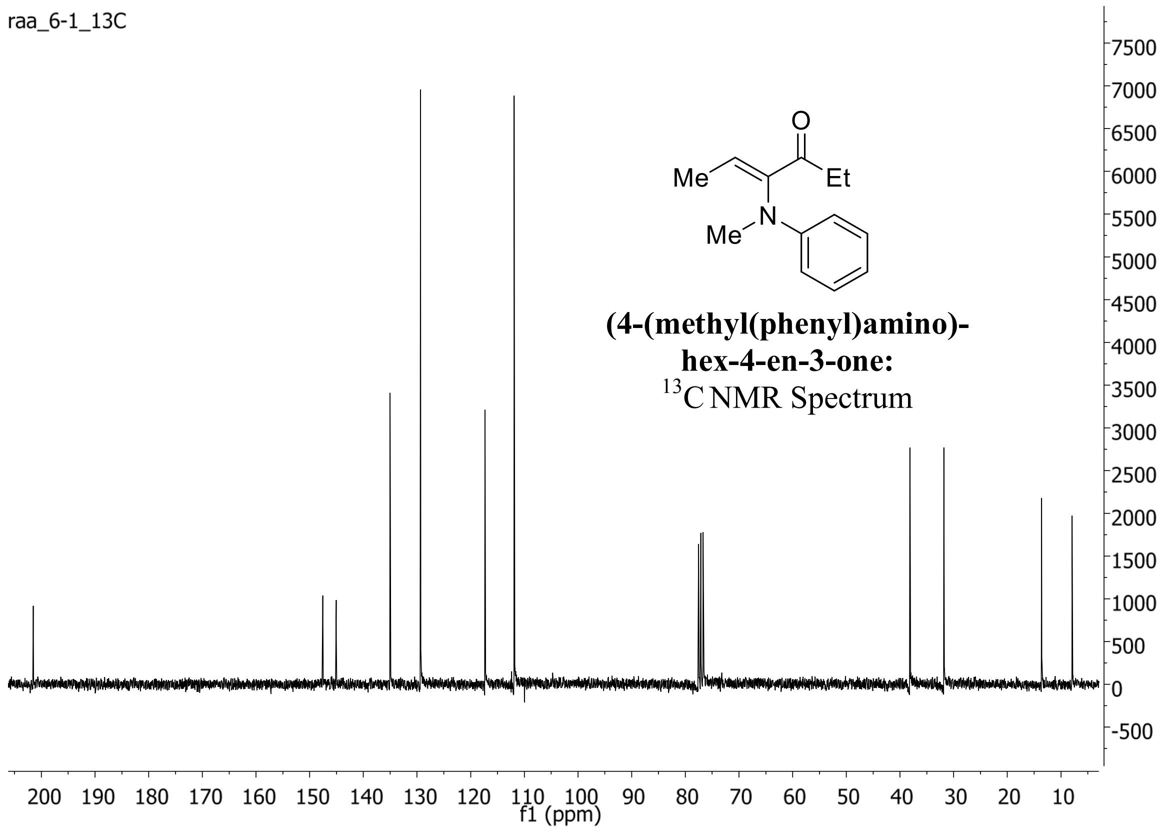

raa_6-1_13C
(4-(methyl(phenyl)amino)-
hex-4-en-3-one:
¹³C NMR Spectrum
f1 (ppm)

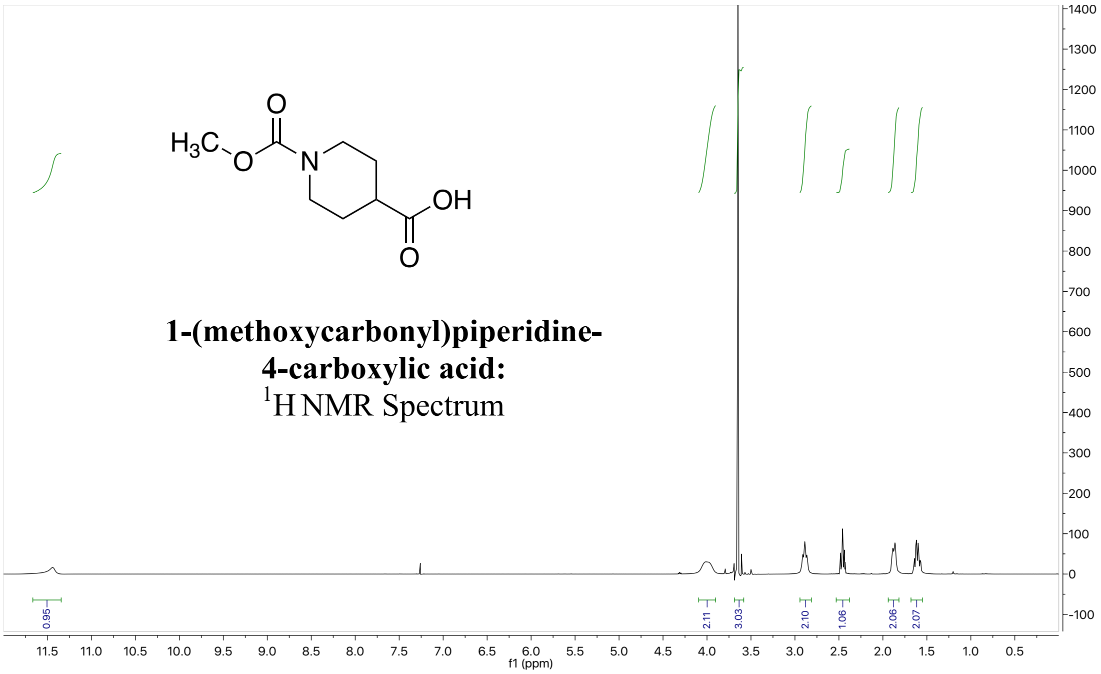
H3C O N O OH O
1-(methoxycarbonyl)piperidine-
4-carboxylic acid:
1H NMR Spectrum
0.95 2.11 3.03 2.10 1.06 2.06 2.07
f1 (ppm)

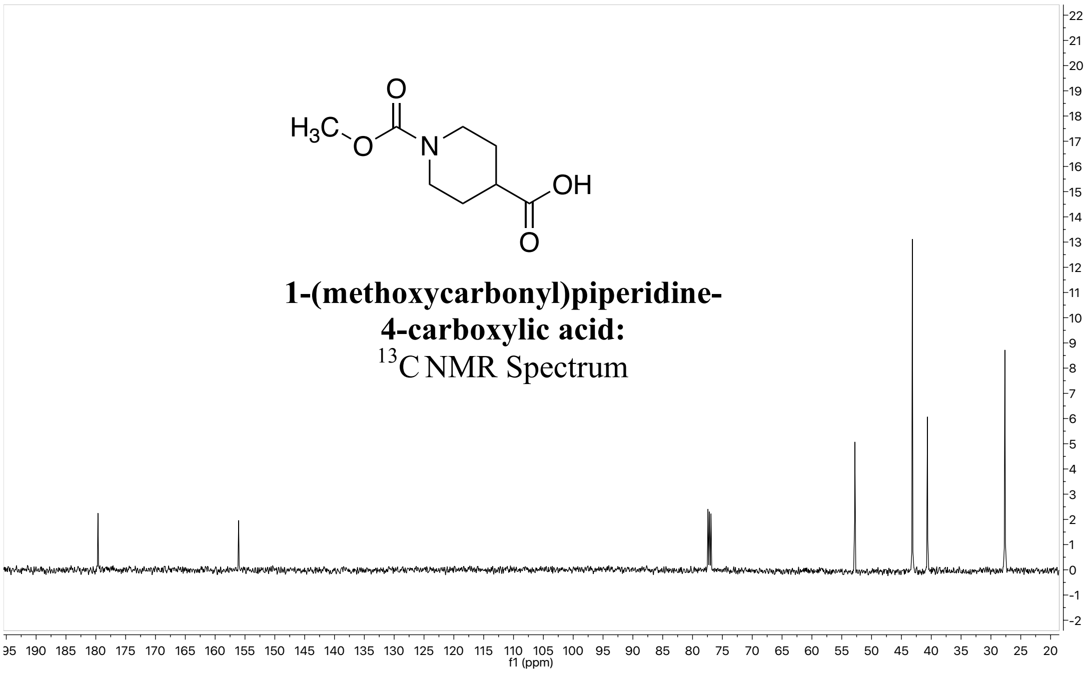
H3C O N O OH O
1-(methoxycarbonyl)piperidine-
4-carboxylic acid:
13C NMR Spectrum
f1 (ppm)

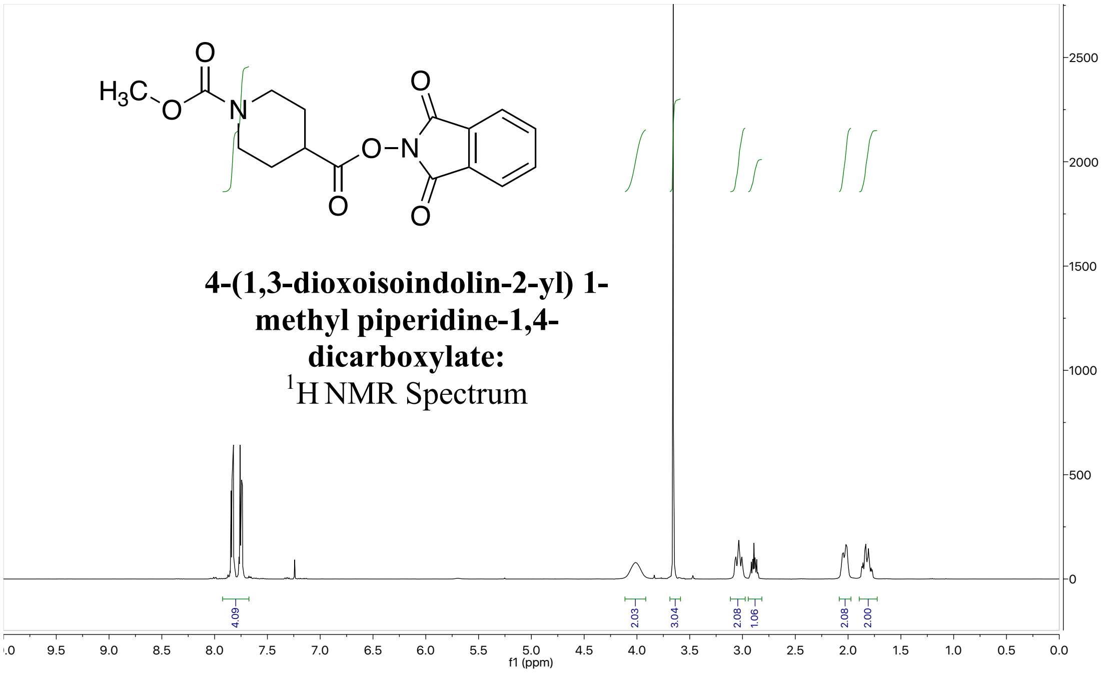
4-(1,3-dioxoisoindolin-2-yl) 1-methyl piperidine-1,4-dicarboxylate:
^{1}H NMR Spectrum
f1 (ppm)

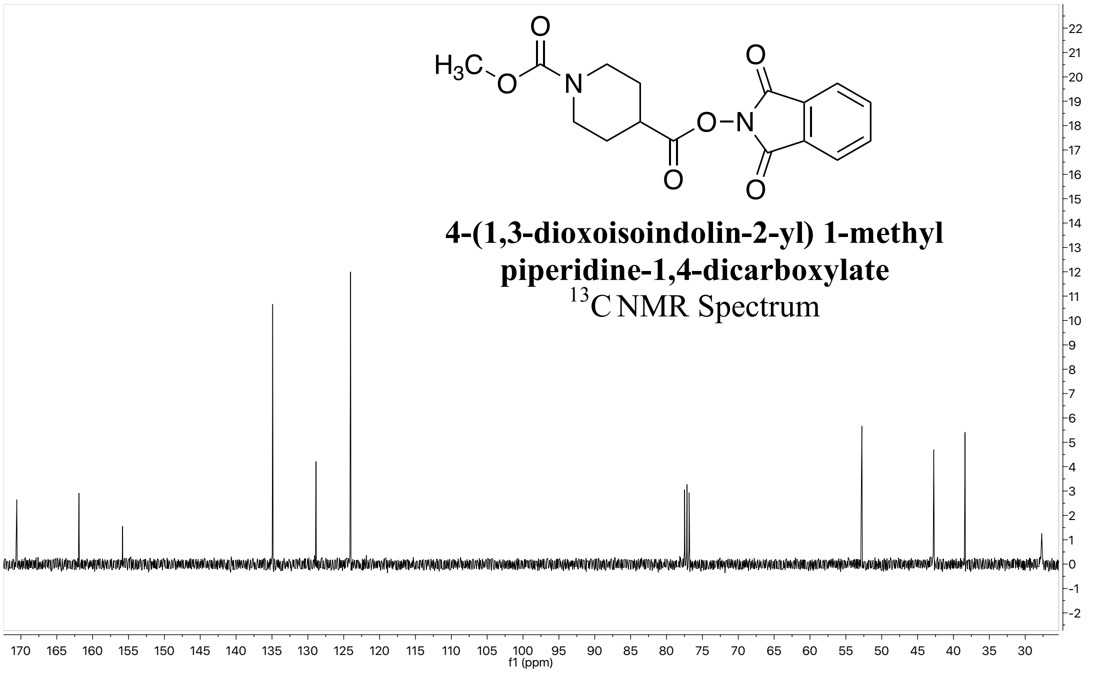
4-(1,3-dioxoisoindolin-2-yl) 1-methyl piperidine-1,4-dicarboxylate
^{13}C NMR Spectrum
f1 (ppm)

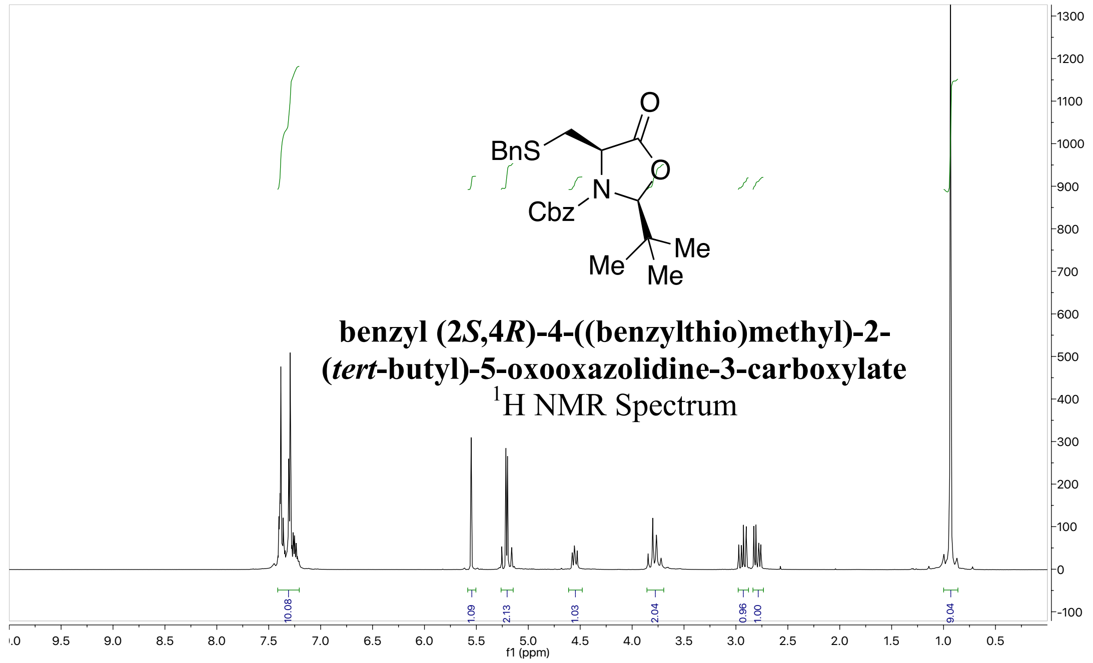

BnS
O
O
N
Cbz
Me
Me
Me
benzyl (2S,4R)-4-((benzylthio)methyl)-2-(tert-butyl)-5-oxooxazolidine-3-carboxylate
1H NMR Spectrum
10.08
1.09
2.13
1.03
2.04
0.96
1.00
9.04
f1 (ppm)

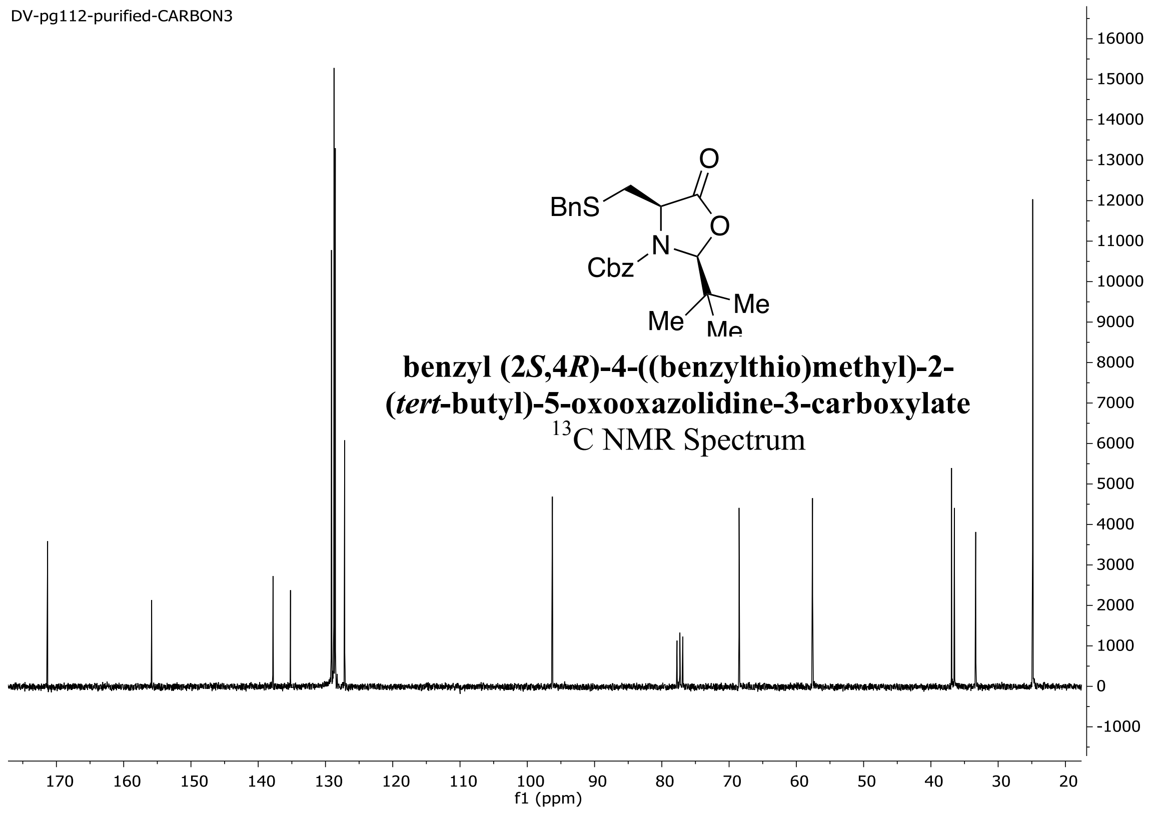

DV-pg112-purified-CARBON3
BnS
O
O
N
Cbz
Me
Me
benzyl (2S,4R)-4-((benzylthio)methyl)-2-(tert-butyl)-5-oxooxazolidine-3-carboxylate
13C NMR Spectrum
f1 (ppm)

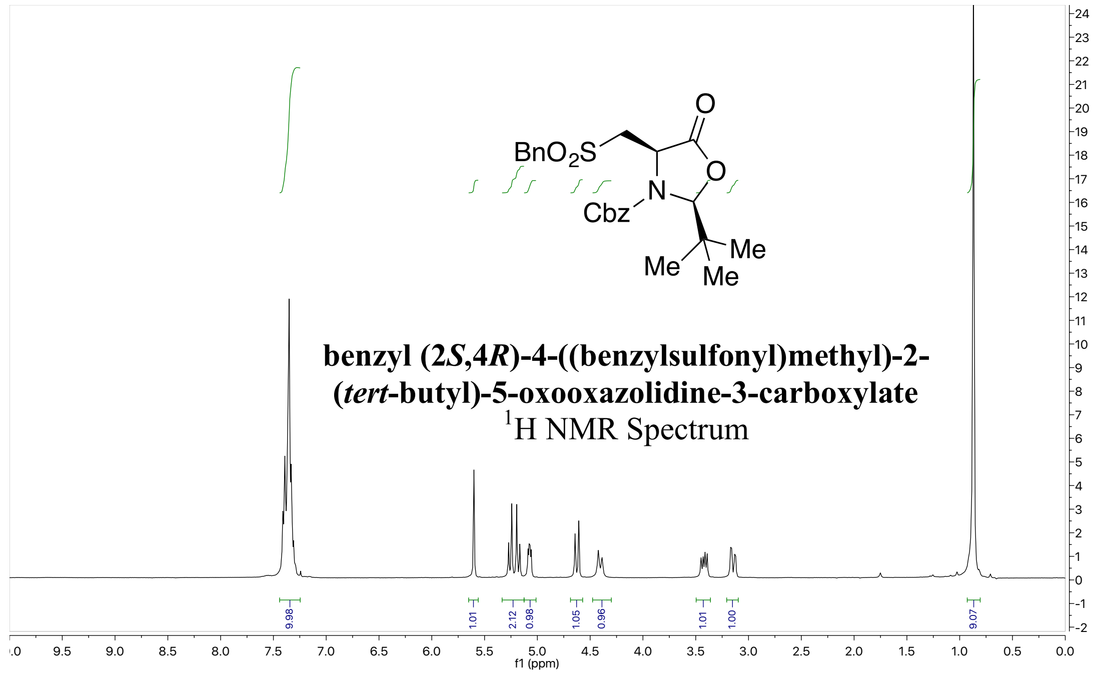
BnO2S
O
N
O
Cbz
Me
Me
Me
benzyl (2S,4R)-4-((benzylsulfonyl)methyl)-2-
(tert-butyl)-5-oxooxazolidine-3-carboxylate
1H NMR Spectrum
9.98
1.01
2.12
0.98
1.05
0.96
1.01
1.00
9.07
f1 (ppm)

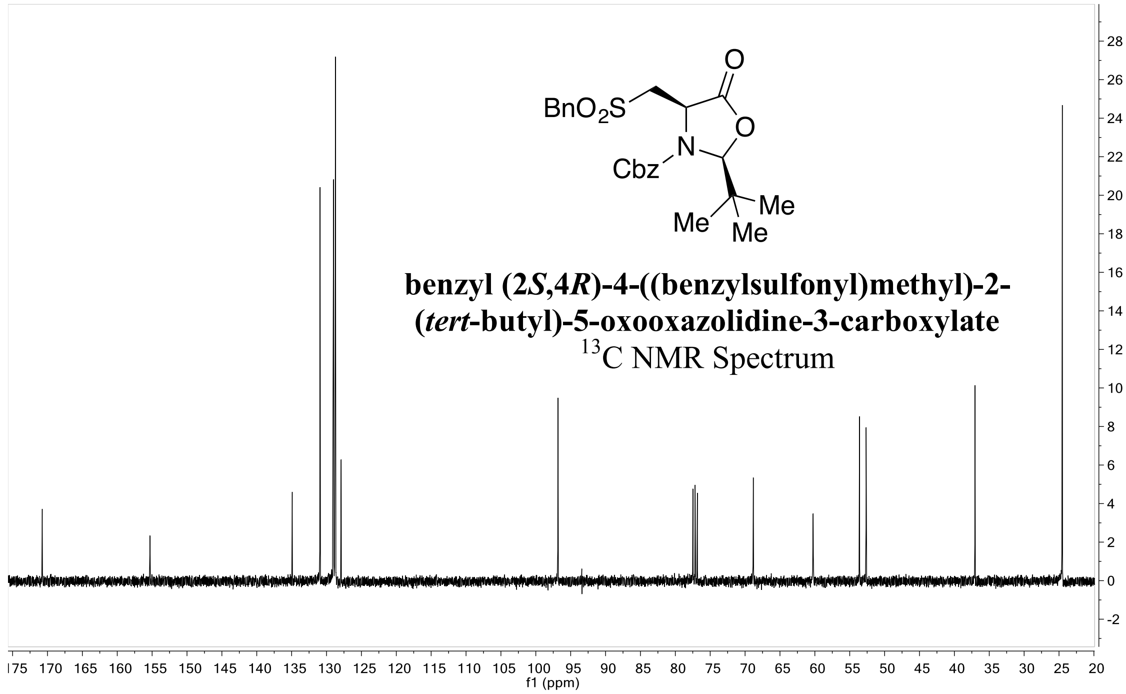
BnO2S
O
N
O
Cbz
Me
Me
Me
benzyl (2S,4R)-4-((benzylsulfonyl)methyl)-2-
(tert-butyl)-5-oxooxazolidine-3-carboxylate
13C NMR Spectrum
f1 (ppm)

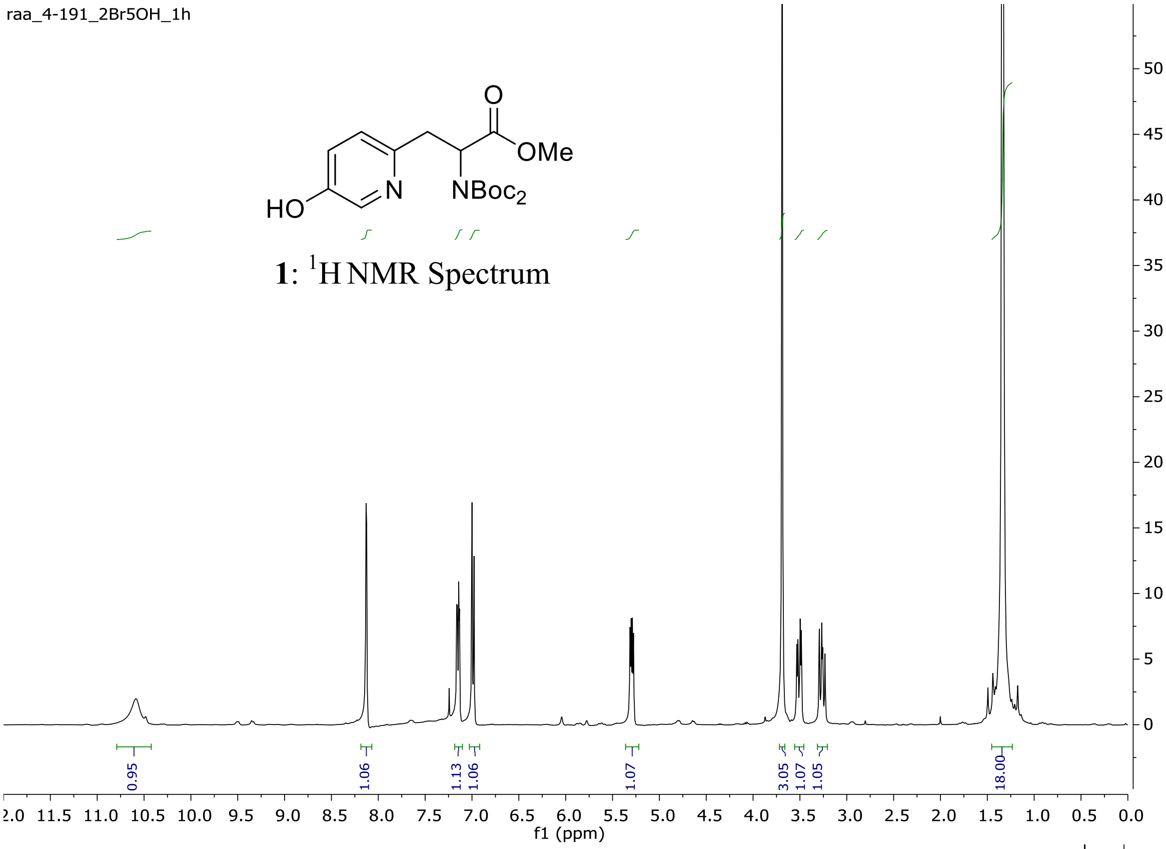
raa_4-191_2Br5OH_1h
O
OMe
HO
N
NBoc2
1: 1H NMR Spectrum
0.95
1.06
1.13
1.06
1.07
3.05
1.07
1.05
18.00
f1 (ppm)

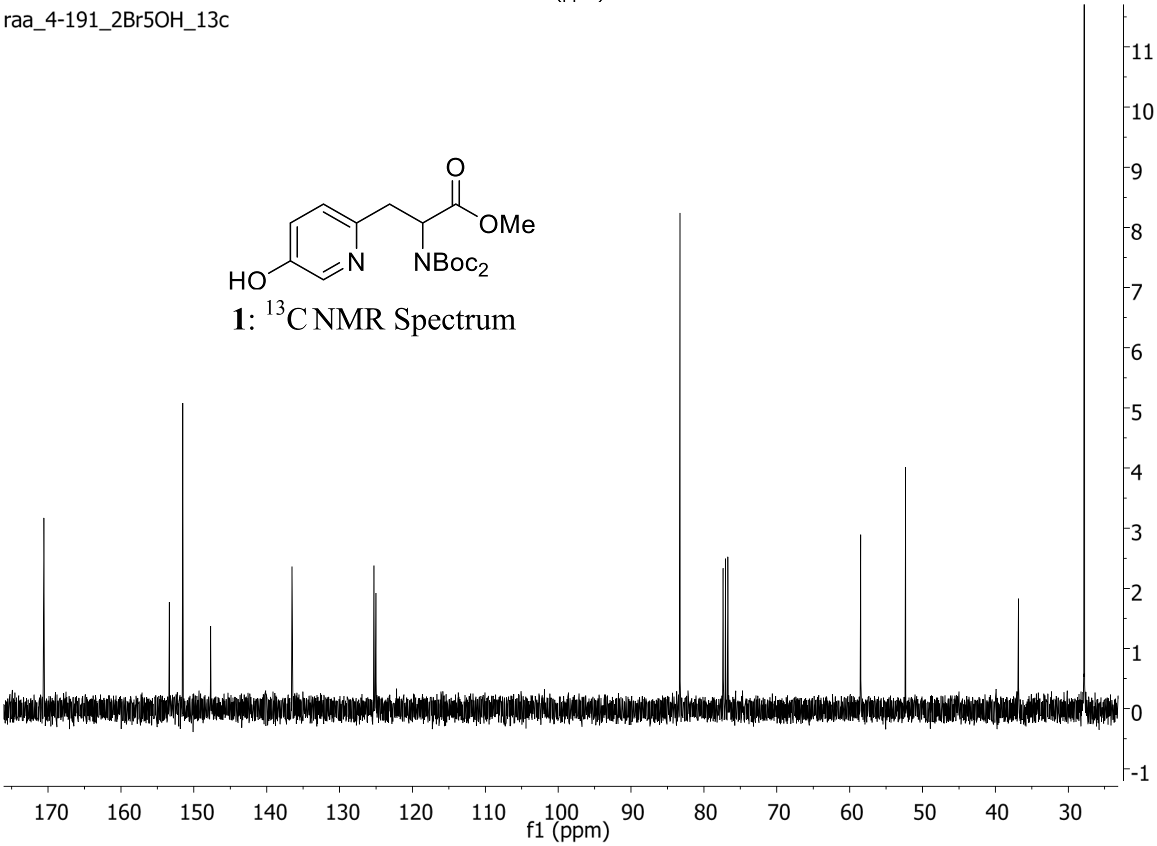
raa_4-191_2Br5OH_13c
O
OMe
HO
N
NBoc2
1: 13C NMR Spectrum
f1 (ppm)

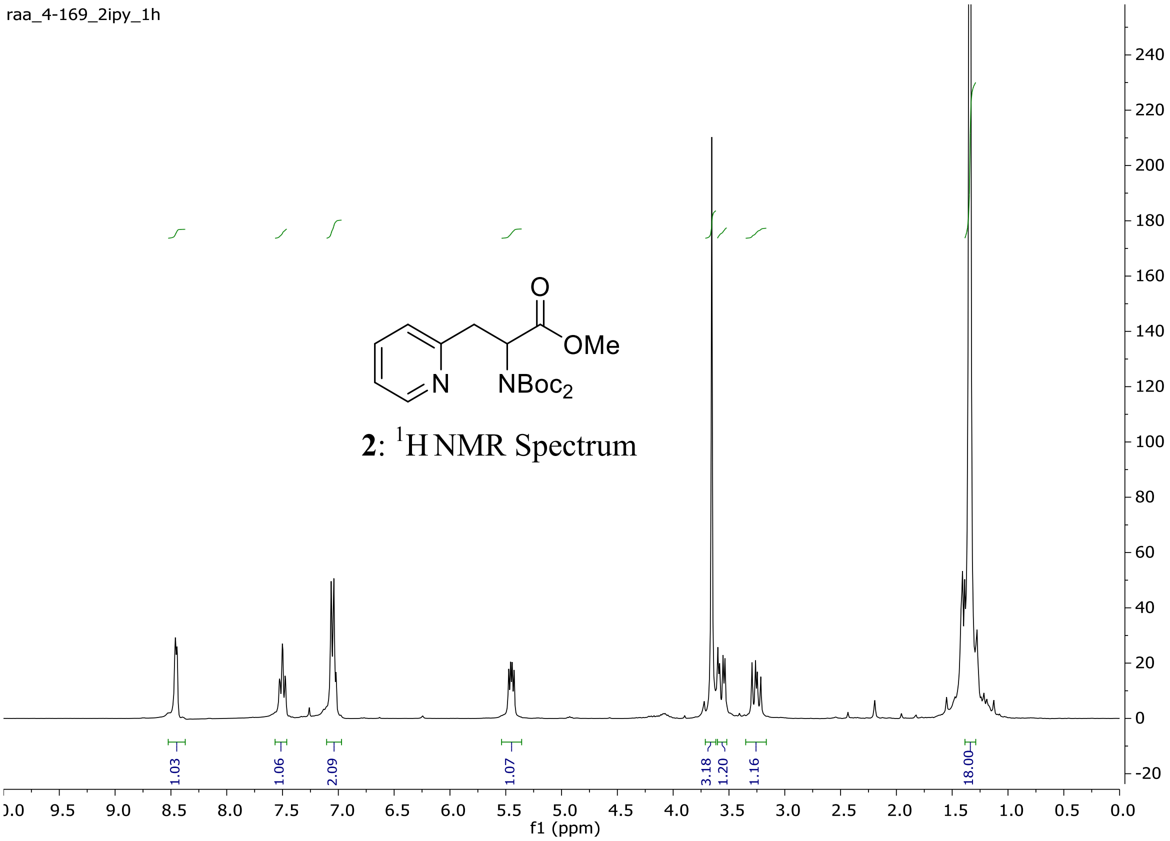
raa_4-169_2ipy_1h
O
OMe
N
NBoc2
2: 1H NMR Spectrum
1.03
1.06
2.09
1.07
3.18
1.20
1.16
18.00
f1 (ppm)

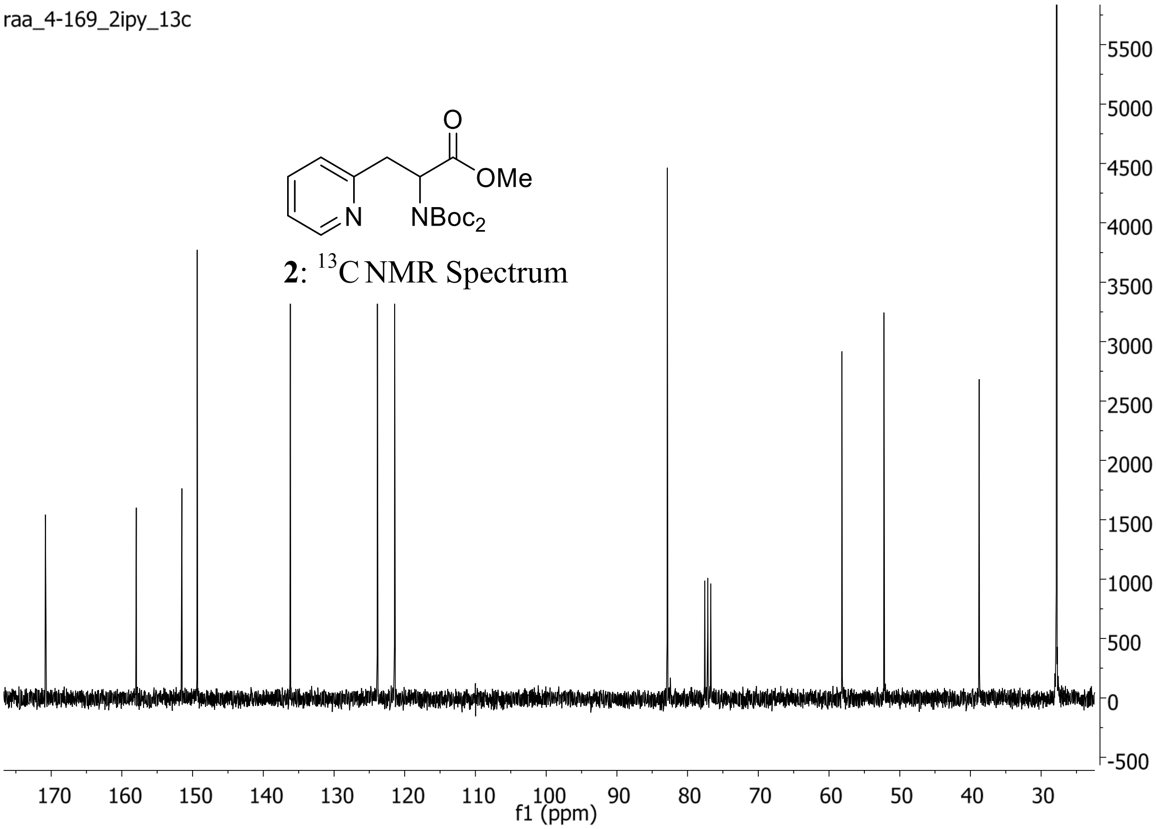
raa_4-169_2ipy_13c
O
OMe
N
NBoc2
2: 13C NMR Spectrum
f1 (ppm)

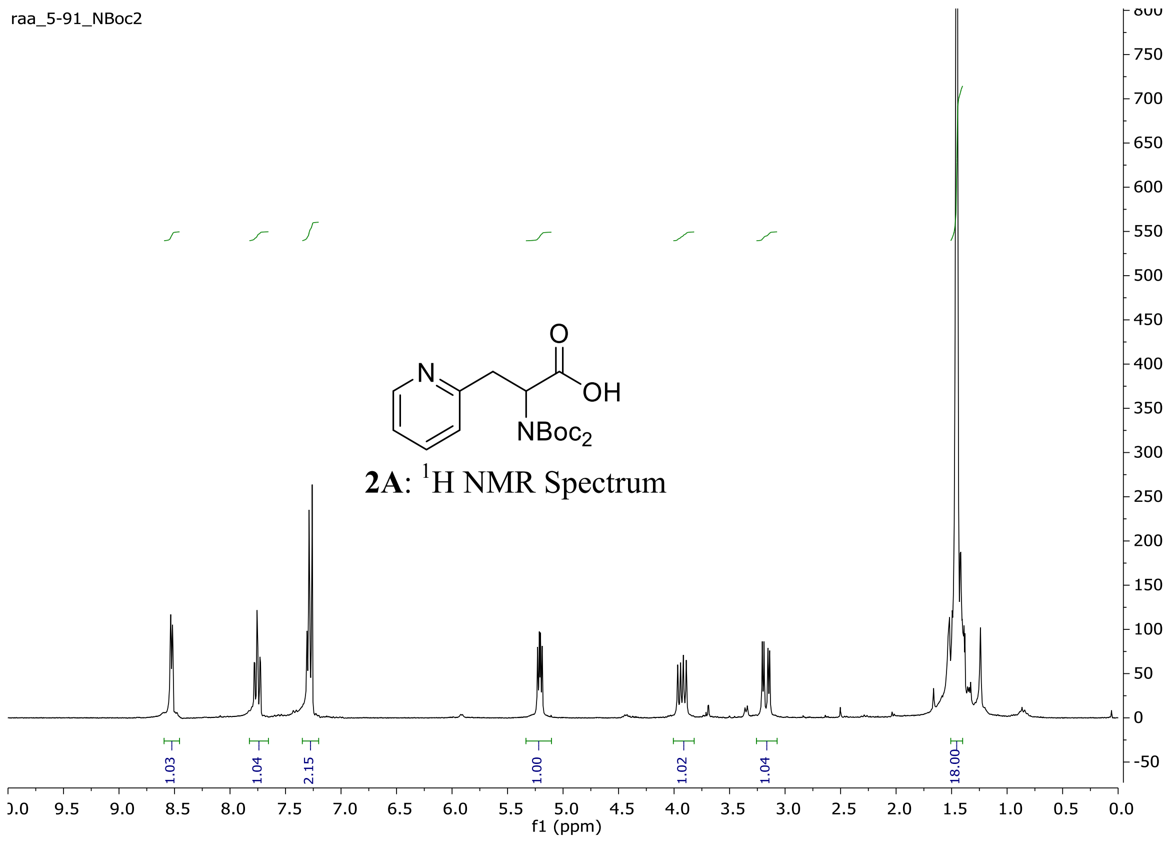
raa_5-91_NBoc2
NBoc2
2A: 1H NMR Spectrum
f1 (ppm)

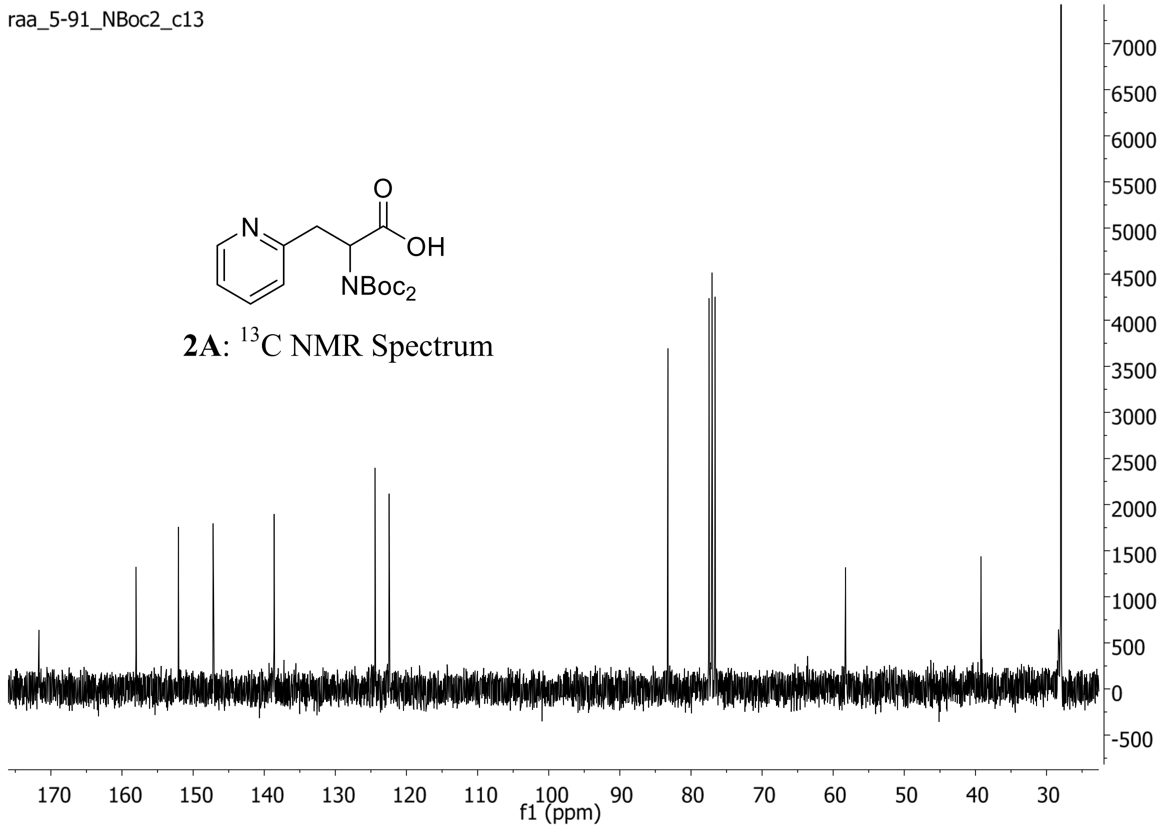
raa_5-91_NBoc2_c13
NBoc2
2A: 13C NMR Spectrum
f1 (ppm)

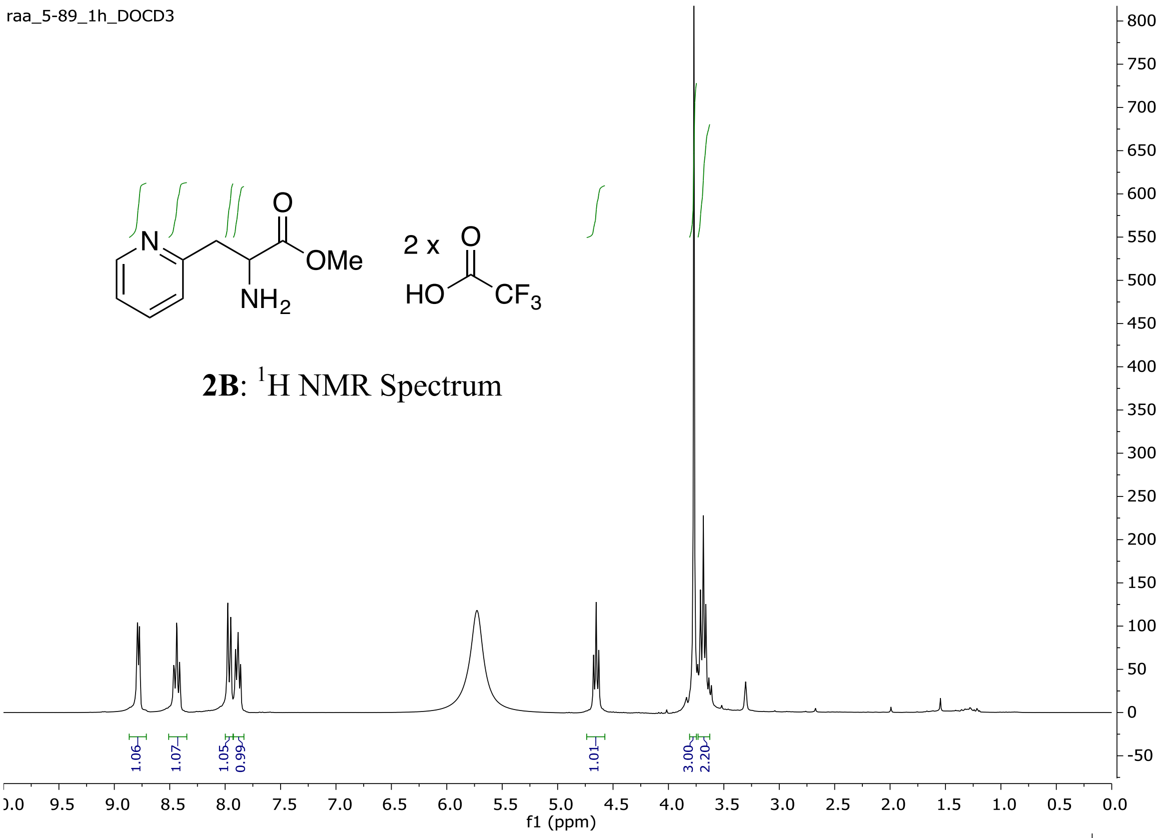
raa_5-89_1h_DOCD3
NH2
OMe
2 x
HO
CF3
2B: 1H NMR Spectrum
1.06
1.07
1.05
0.99
1.01
3.00
2.20
f1 (ppm)

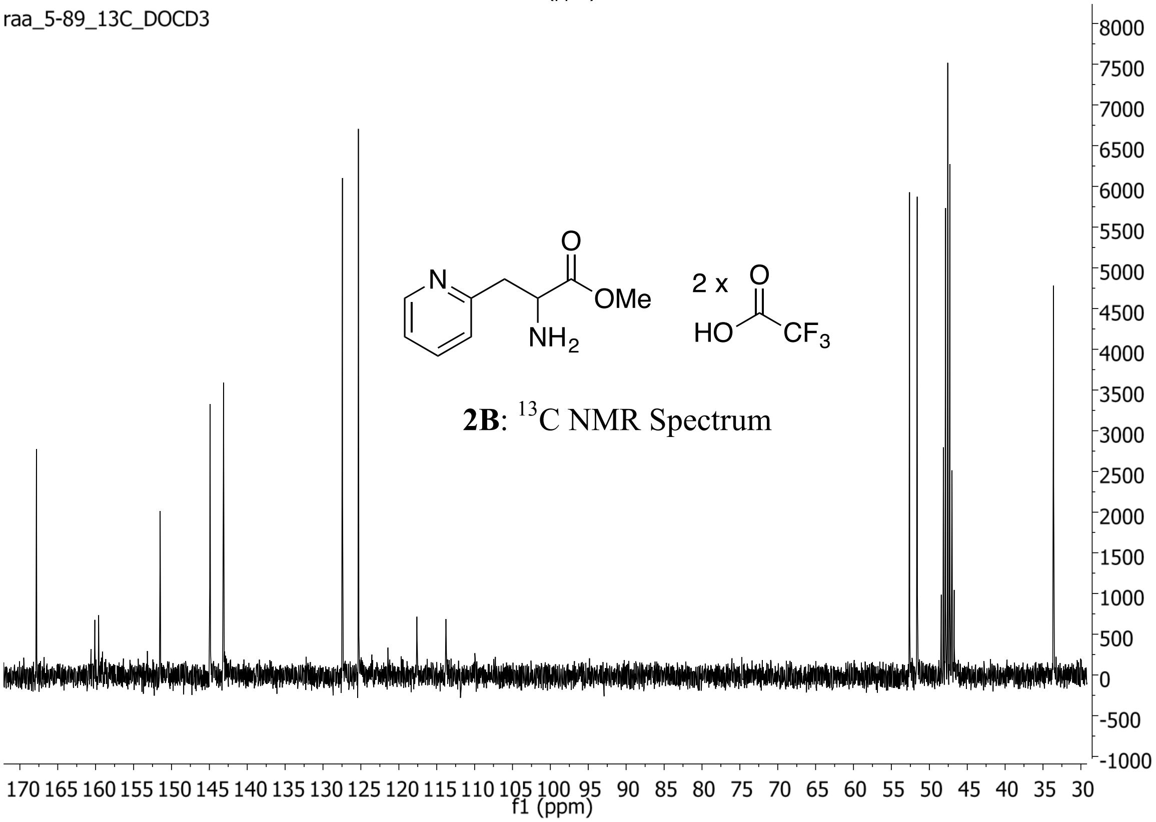
raa_5-89_13C_DOCD3
NH2
OMe
2 x
HO
CF3
2B: 13C NMR Spectrum
f1 (ppm)

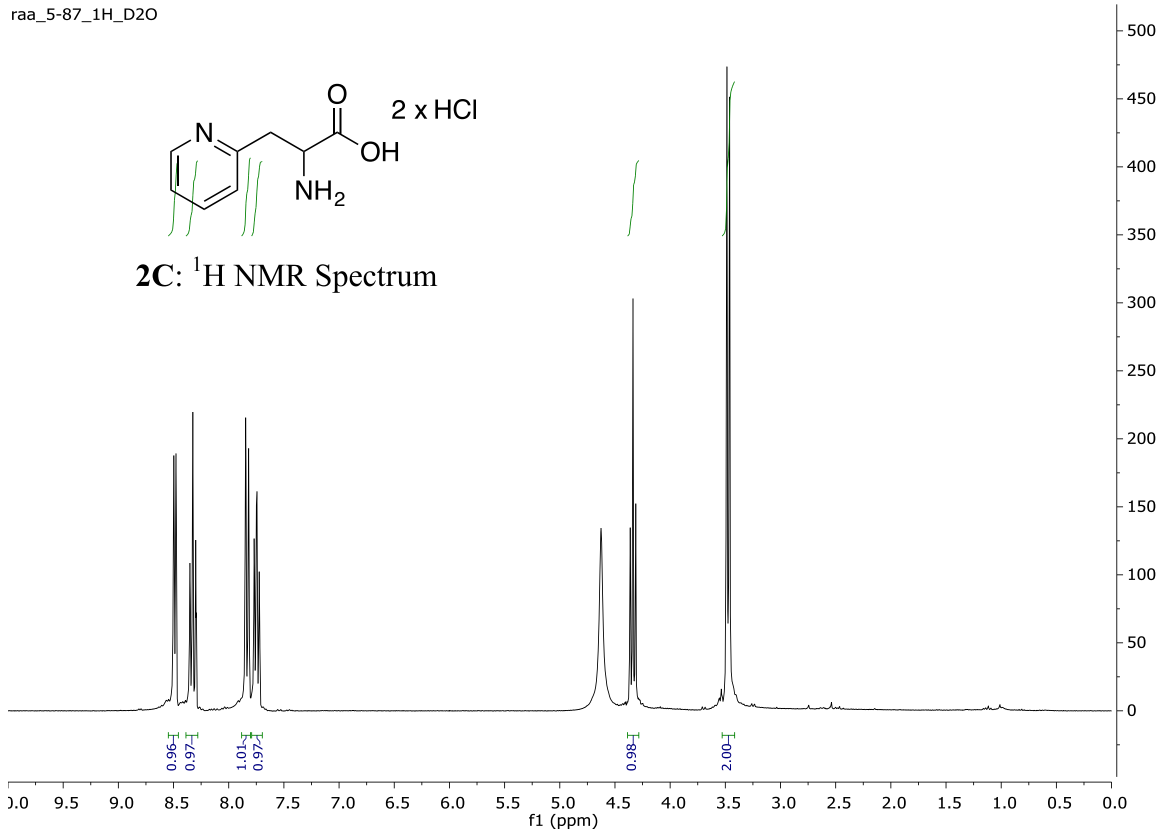
raa_5-87_1H_D2O
2 x HCl
2C: 1H NMR Spectrum
f1 (ppm)

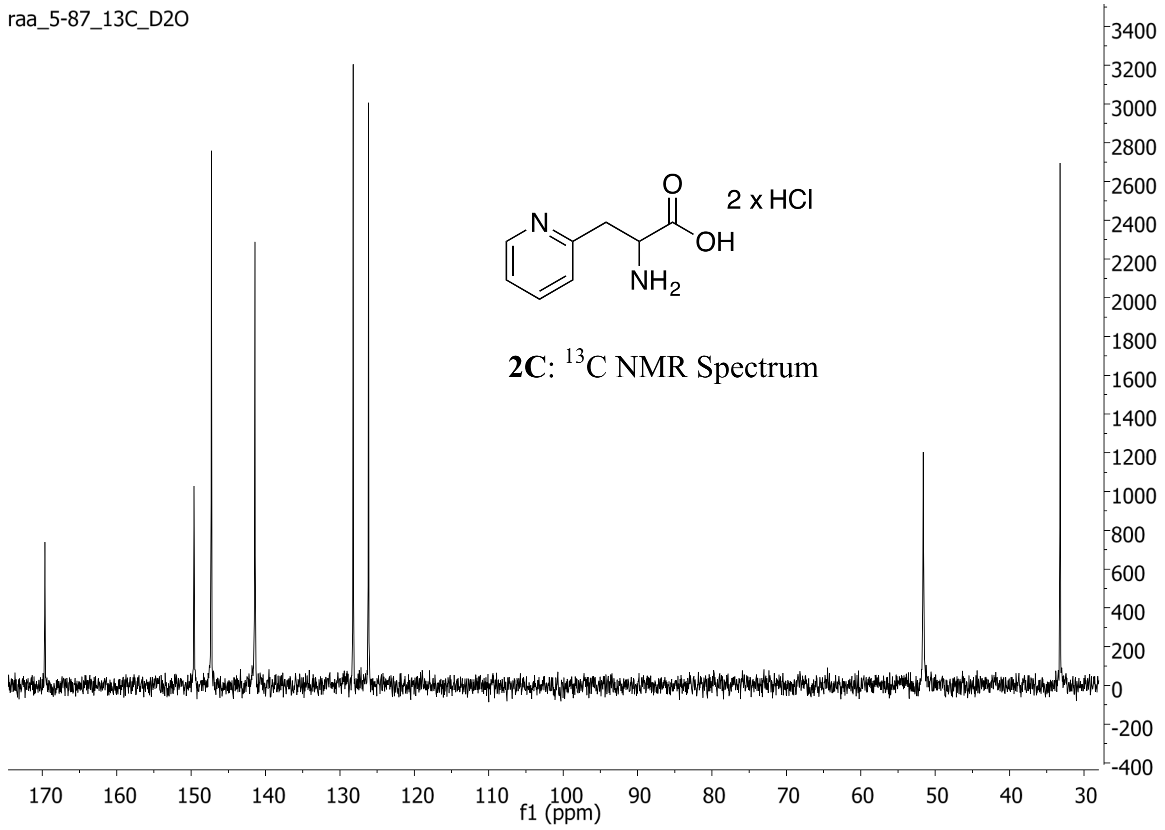
raa_5-87_13C_D2O
2 x HCl
2C: 13C NMR Spectrum
f1 (ppm)

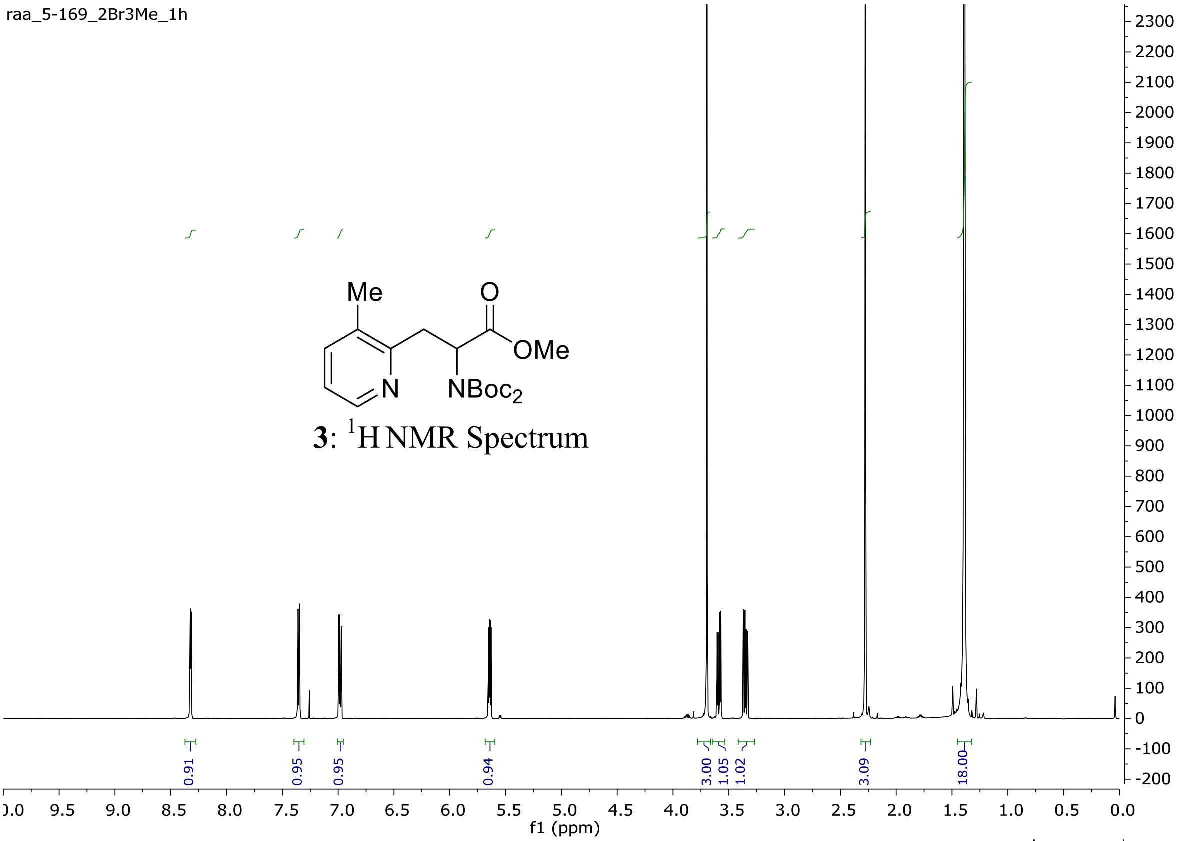
raa_5-169_2Br3Me_1h
Me
O
OMe
N
NBoc2
3: 1H NMR Spectrum
f1 (ppm)

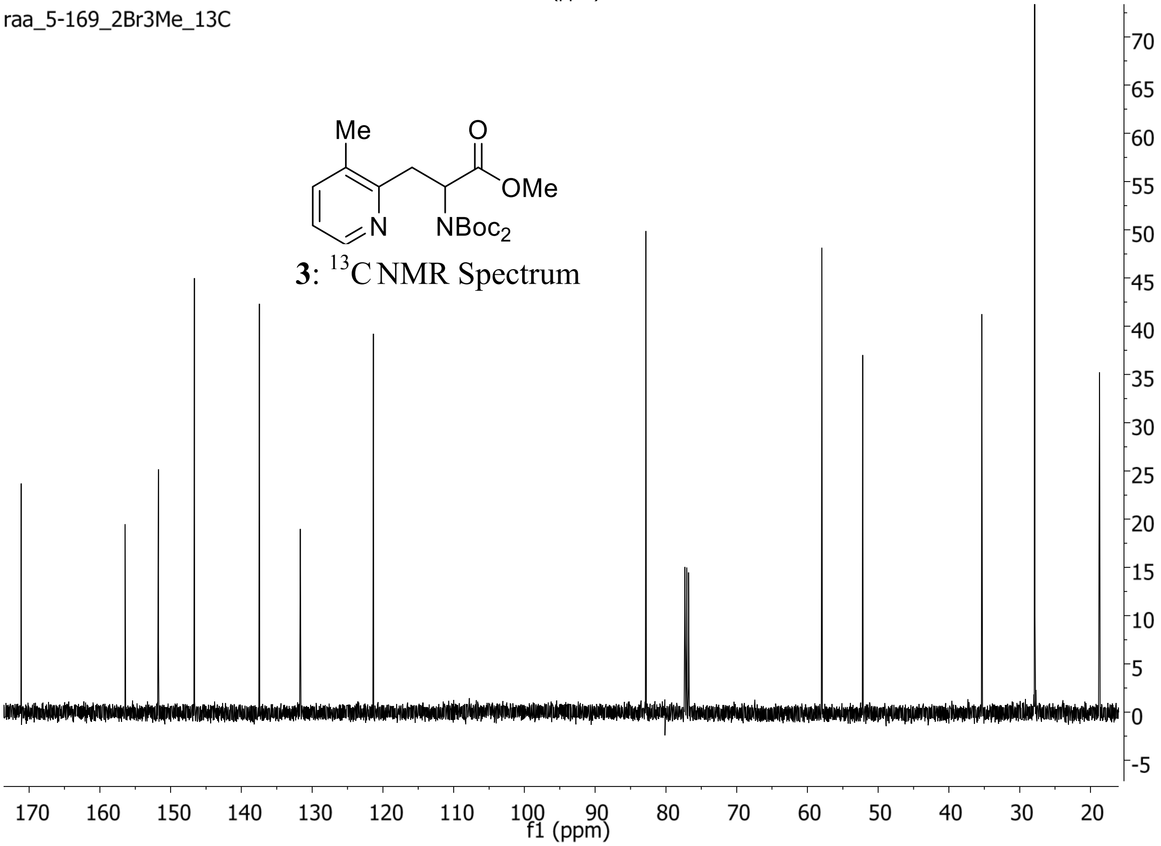
raa_5-169_2Br3Me_13C
Me
O
OMe
N
NBoc2
3: 13C NMR Spectrum
f1 (ppm)

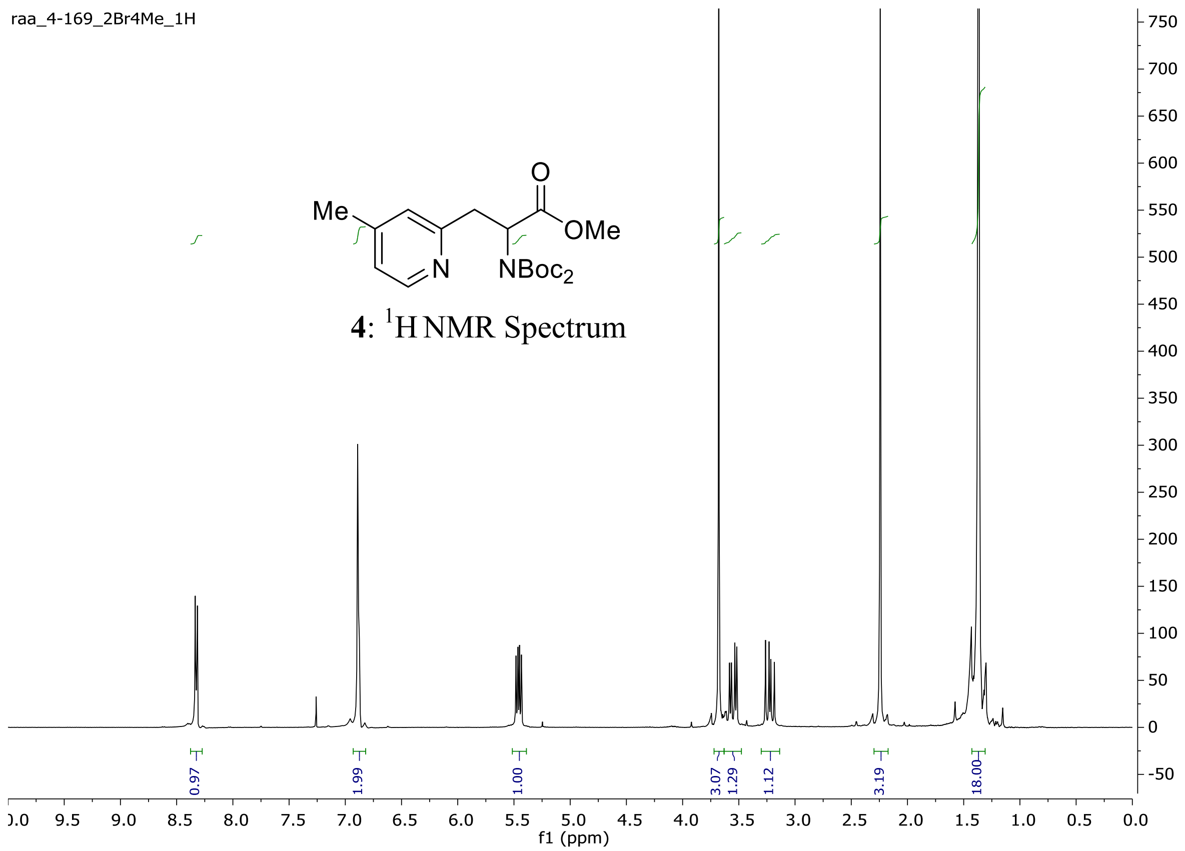
raa_4-169_2Br4Me_1H
Me
O
OMe
N
NBoc2
4: 1H NMR Spectrum
0.97
1.99
1.00
3.07
1.29
1.12
3.19
18.00
f1 (ppm)

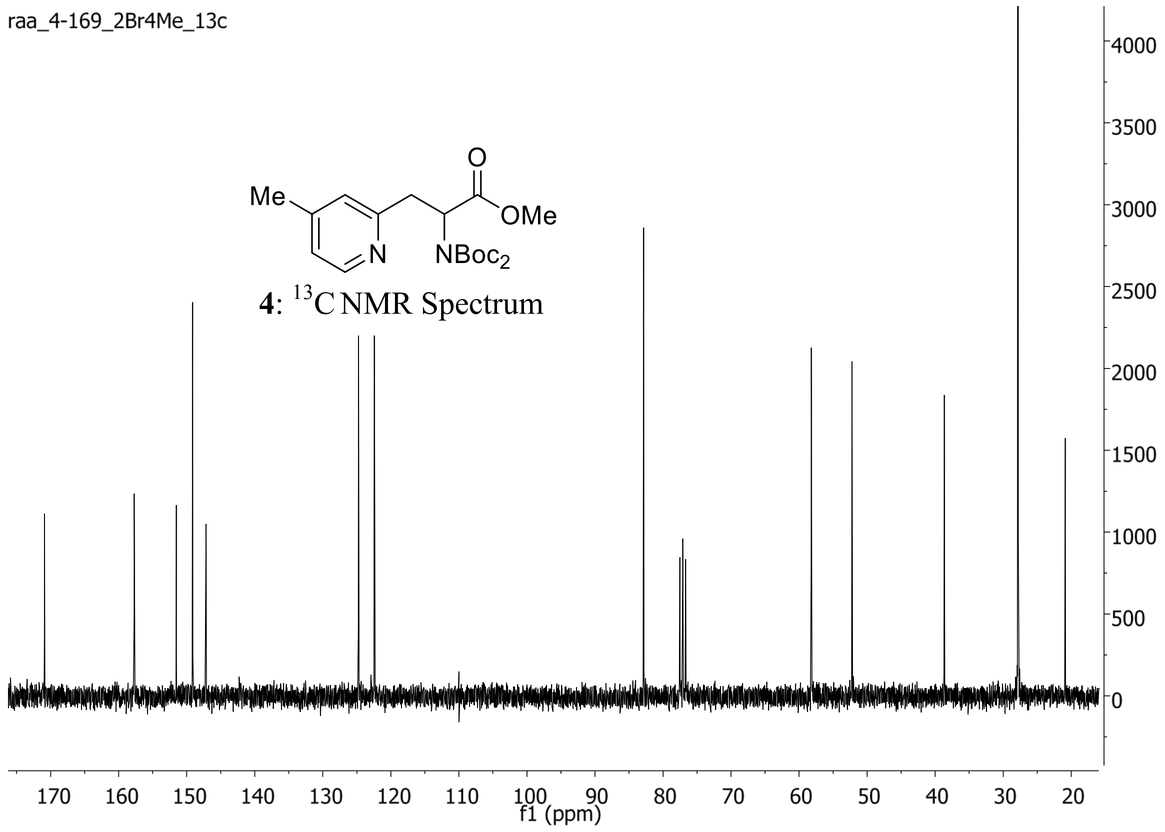
raa_4-169_2Br4Me_13c
Me
O
OMe
N
NBoc2
4: 13C NMR Spectrum
f1 (ppm)

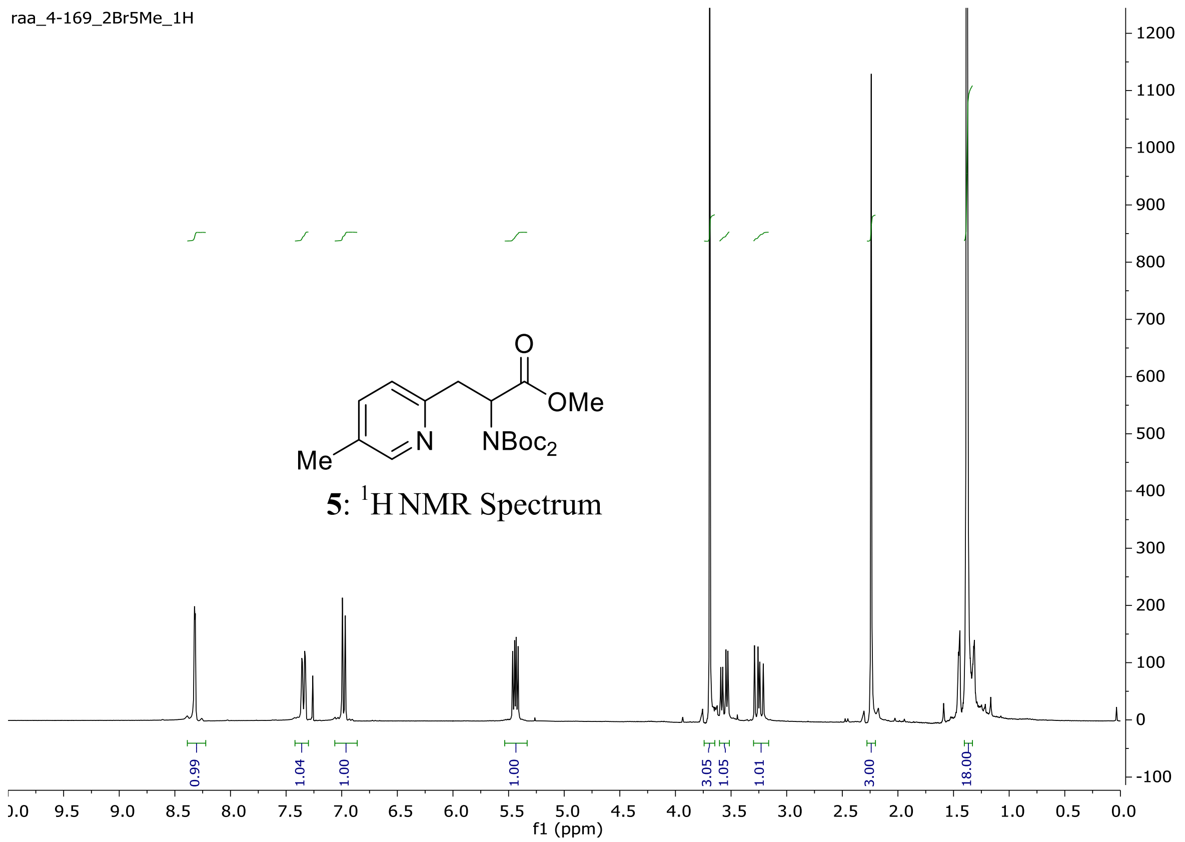

raa_4-169_2Br5Me_1H
O
OMe
N
NBoc2
Me
5: 1H NMR Spectrum
0.99
1.04
1.00
1.00
3.05
1.05
1.01
3.00
18.00
f1 (ppm)

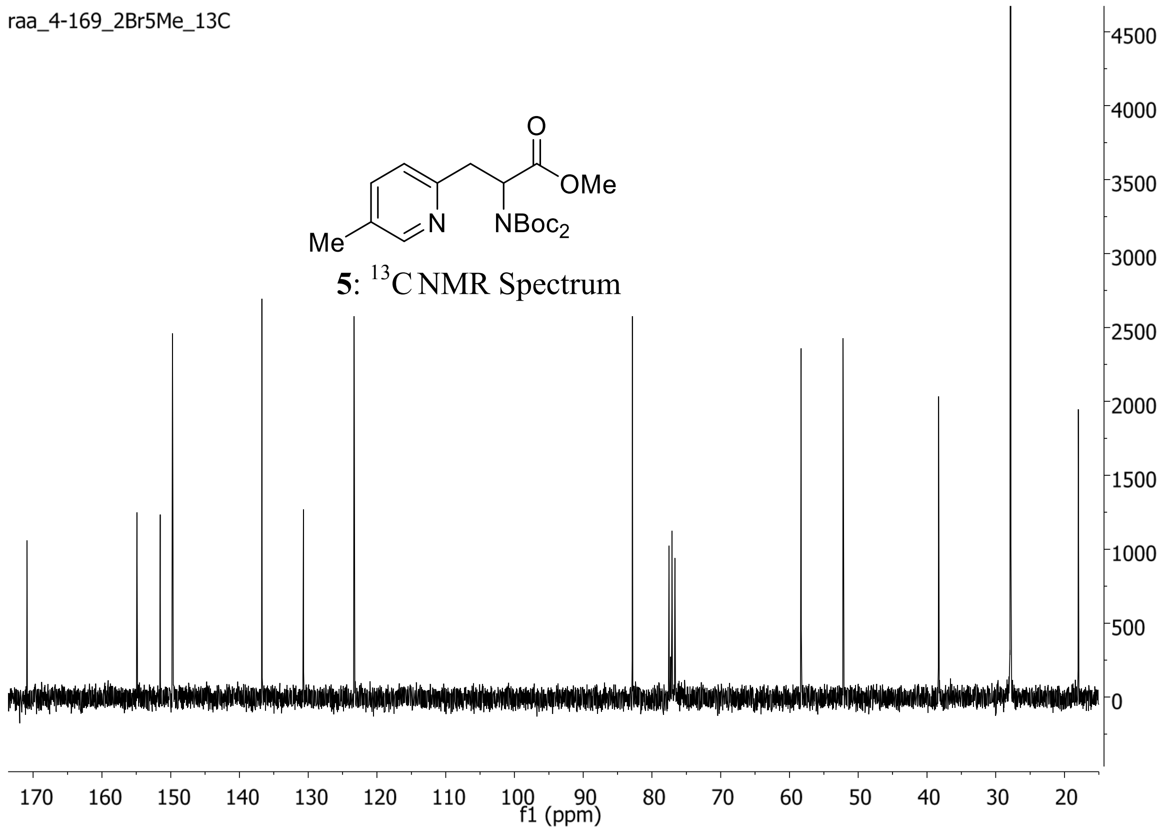

raa_4-169_2Br5Me_13C
O
OMe
N
NBoc2
Me
5: 13C NMR Spectrum
f1 (ppm)

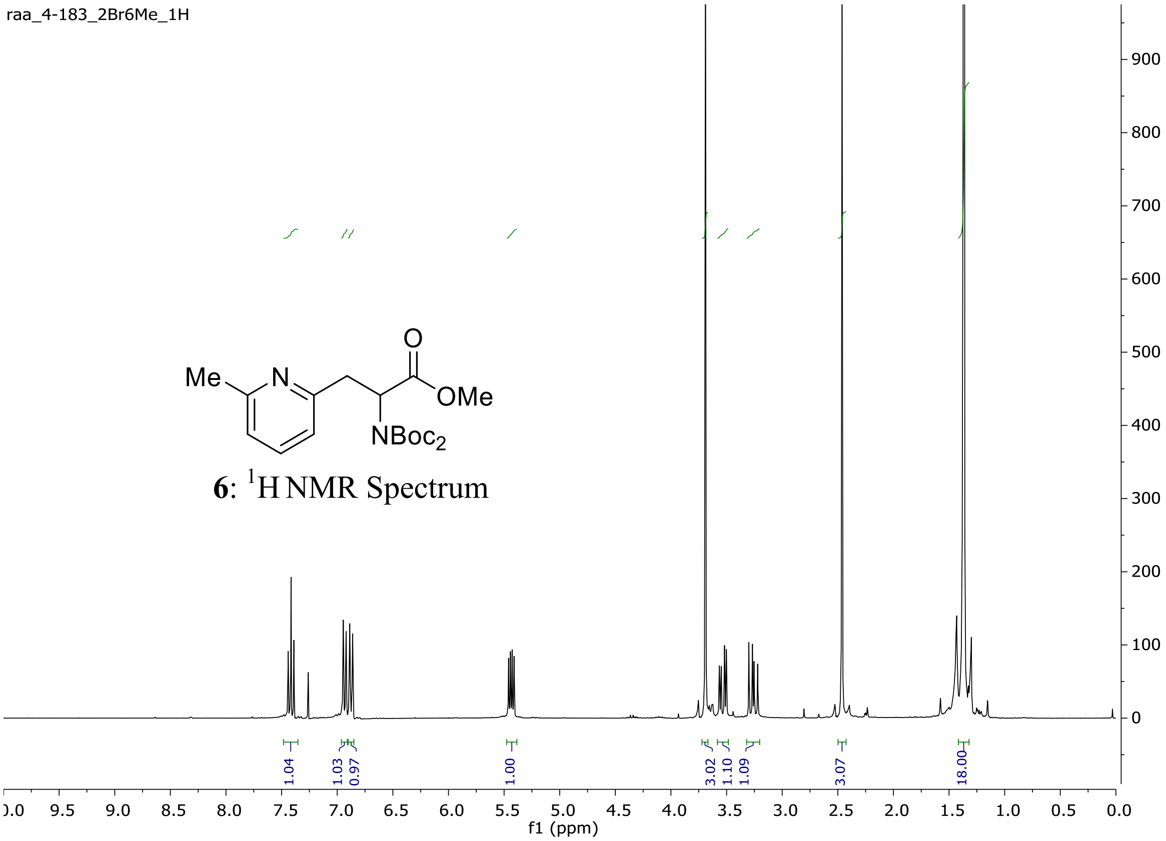

6: ^{1}H NMR Spectrum

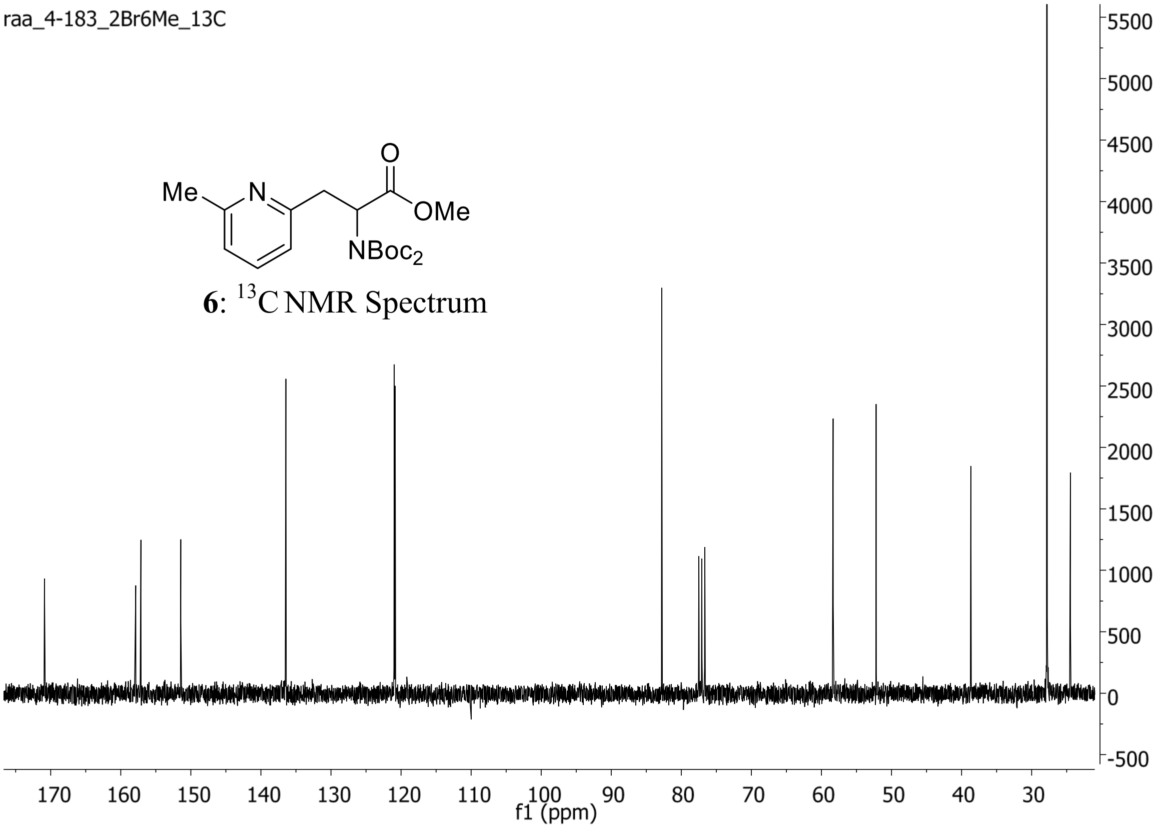

6: ^{13}C NMR Spectrum

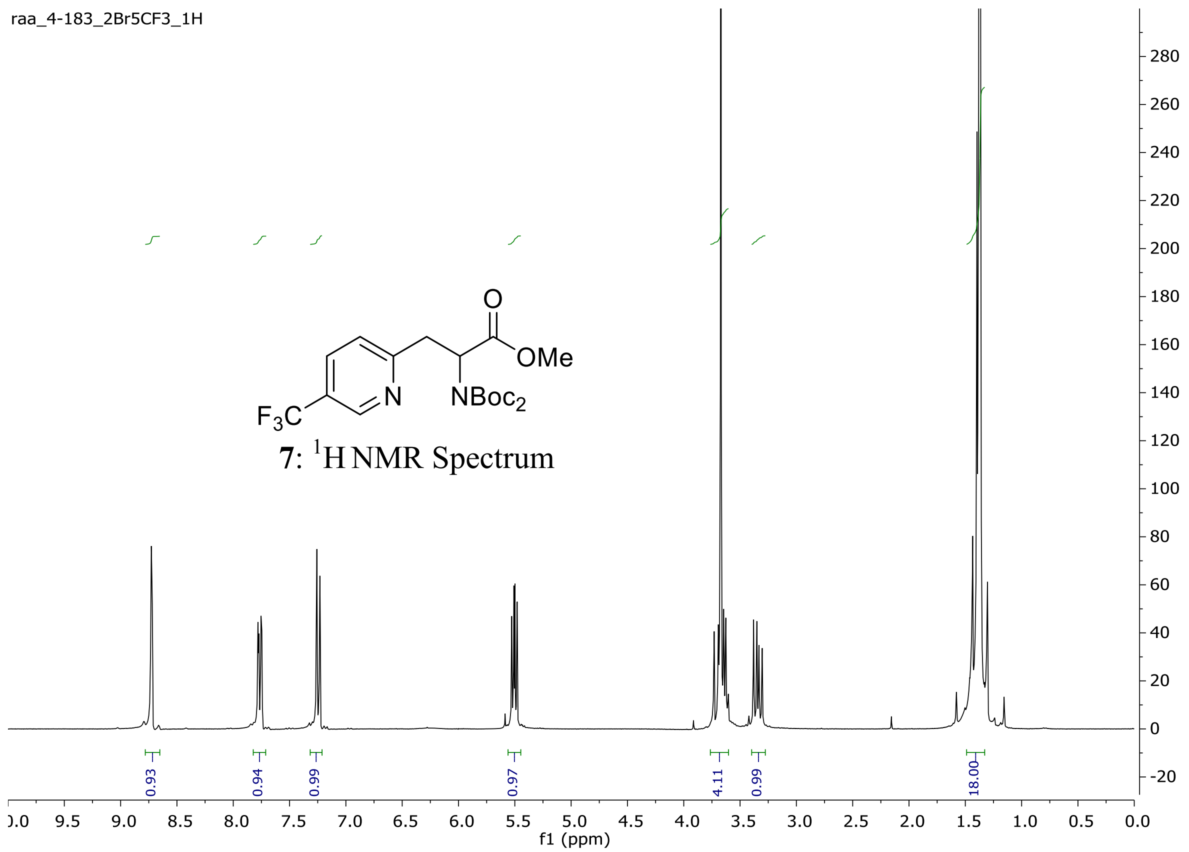
raa_4-183_2Br5CF3_1H
OMe
NBoc2
F3C
N
O
7: 1H NMR Spectrum
0.93
0.94
0.99
0.97
4.11
0.99
18.00
f1 (ppm)

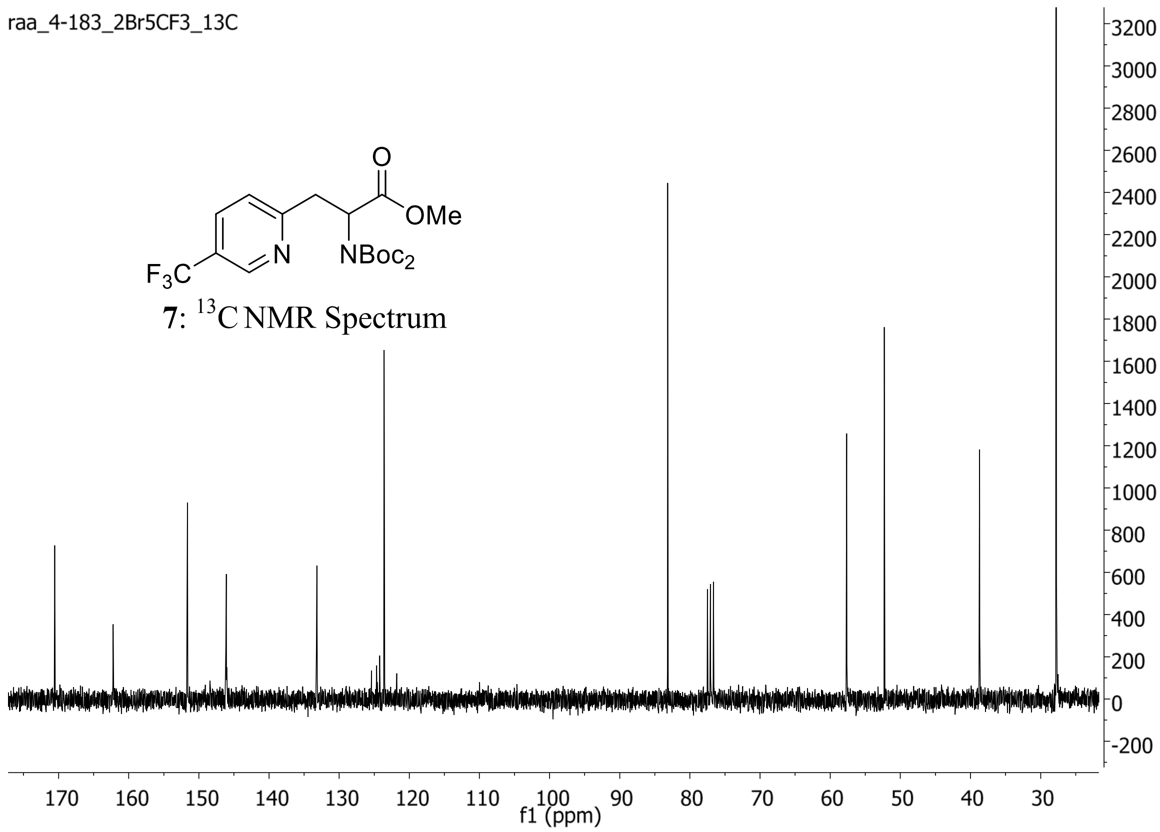
raa_4-183_2Br5CF3_13C
OMe
NBoc2
F3C
N
O
7: 13C NMR Spectrum
f1 (ppm)

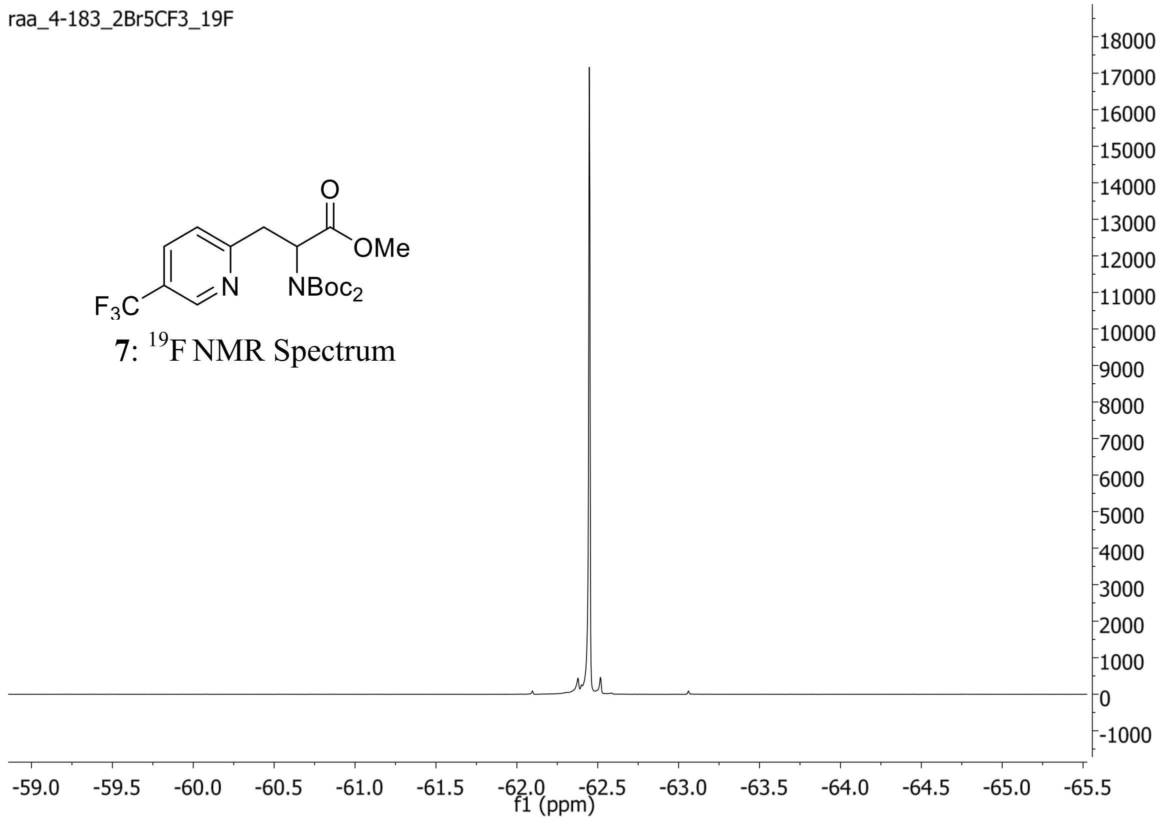

7: ^{19}F NMR Spectrum

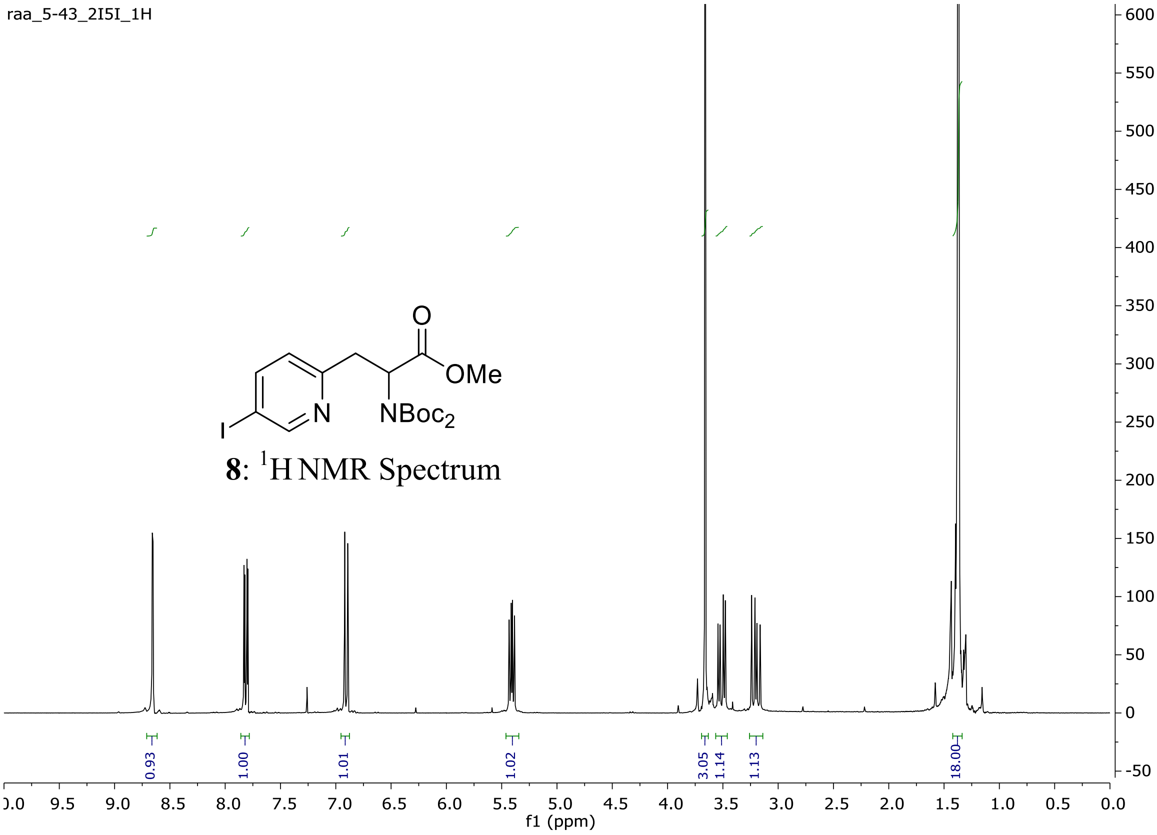
raa_5-43_2I5I_1H
O
OMe
N
NBoc2
I
8: 1H NMR Spectrum
0.93
1.00
1.01
1.02
3.05
1.14
1.13
18.00
f1 (ppm)

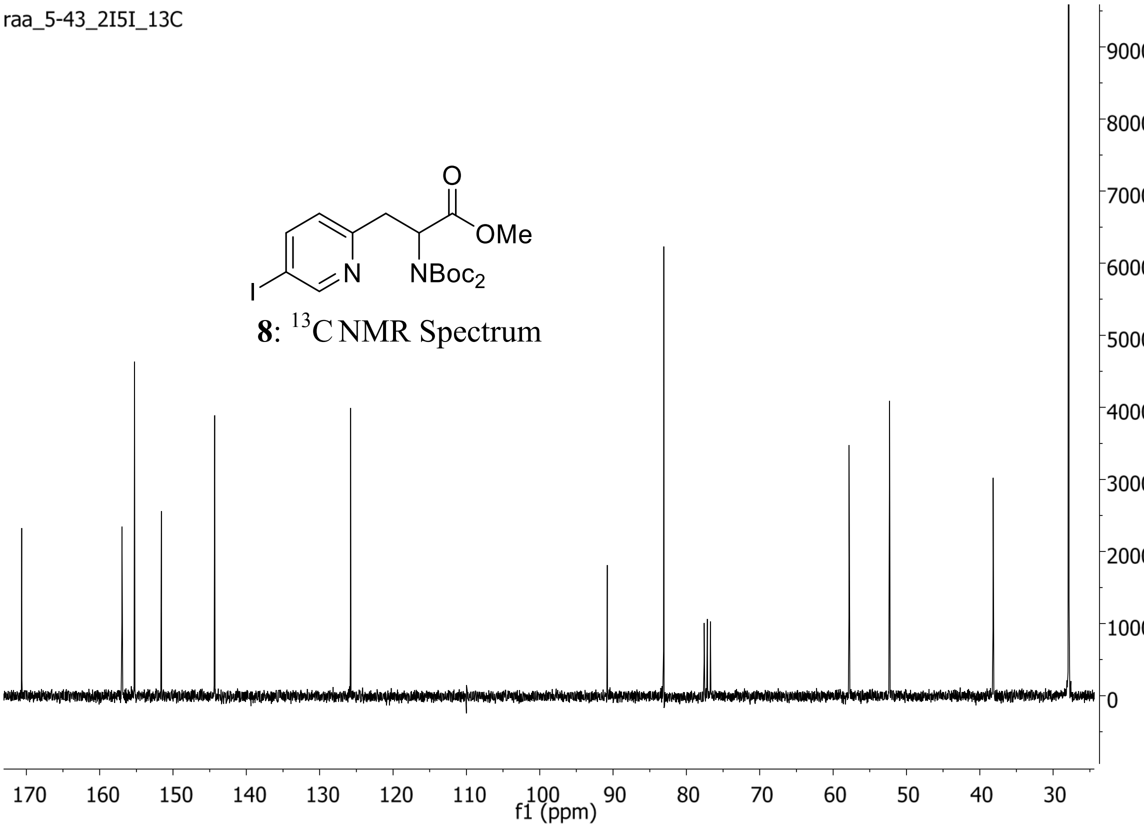
raa_5-43_2I5I_13C
O
OMe
N
NBoc2
I
8: 13C NMR Spectrum
f1 (ppm)

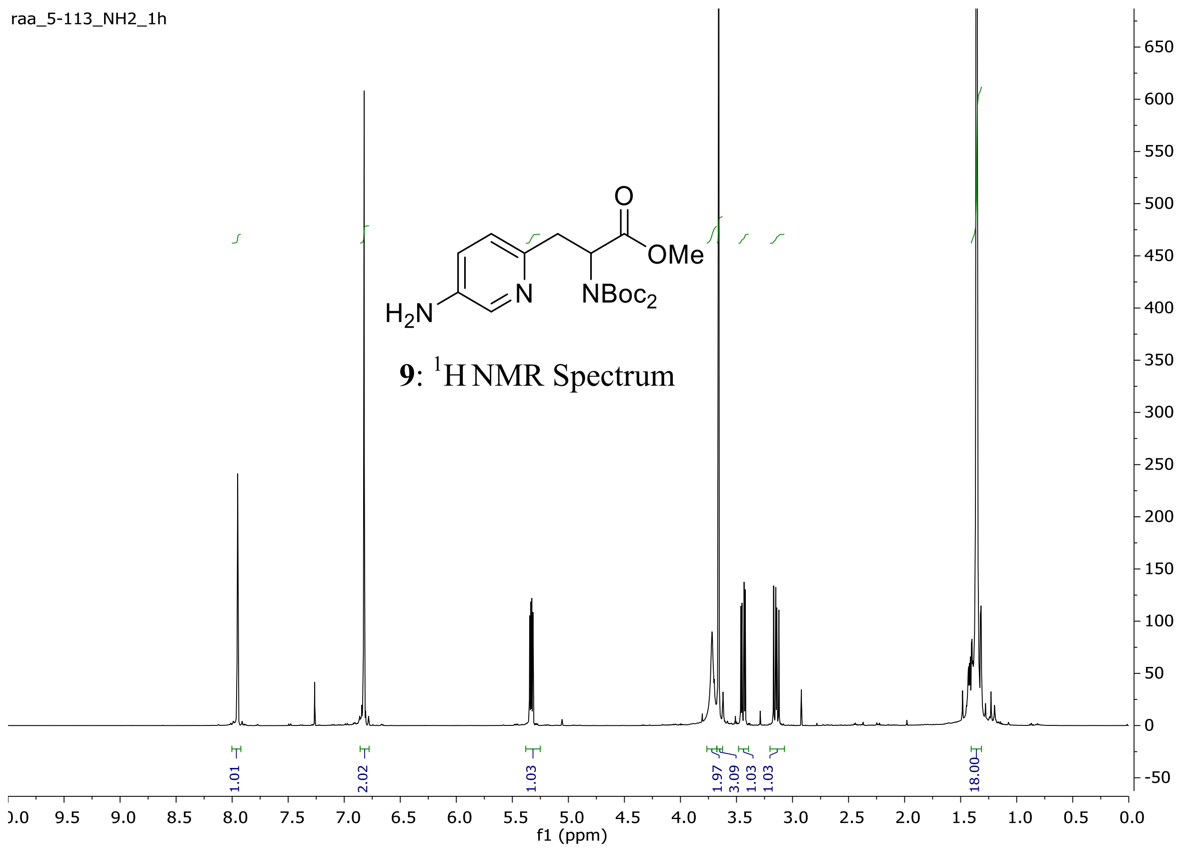
raa_5-113_NH2_1h
O
OMe
H_2N
N
$NBoc_2$
9: ^{1}H NMR Spectrum
1.01
2.02
1.03
1.97
3.09
1.03
1.03
18.00
f1 (ppm)

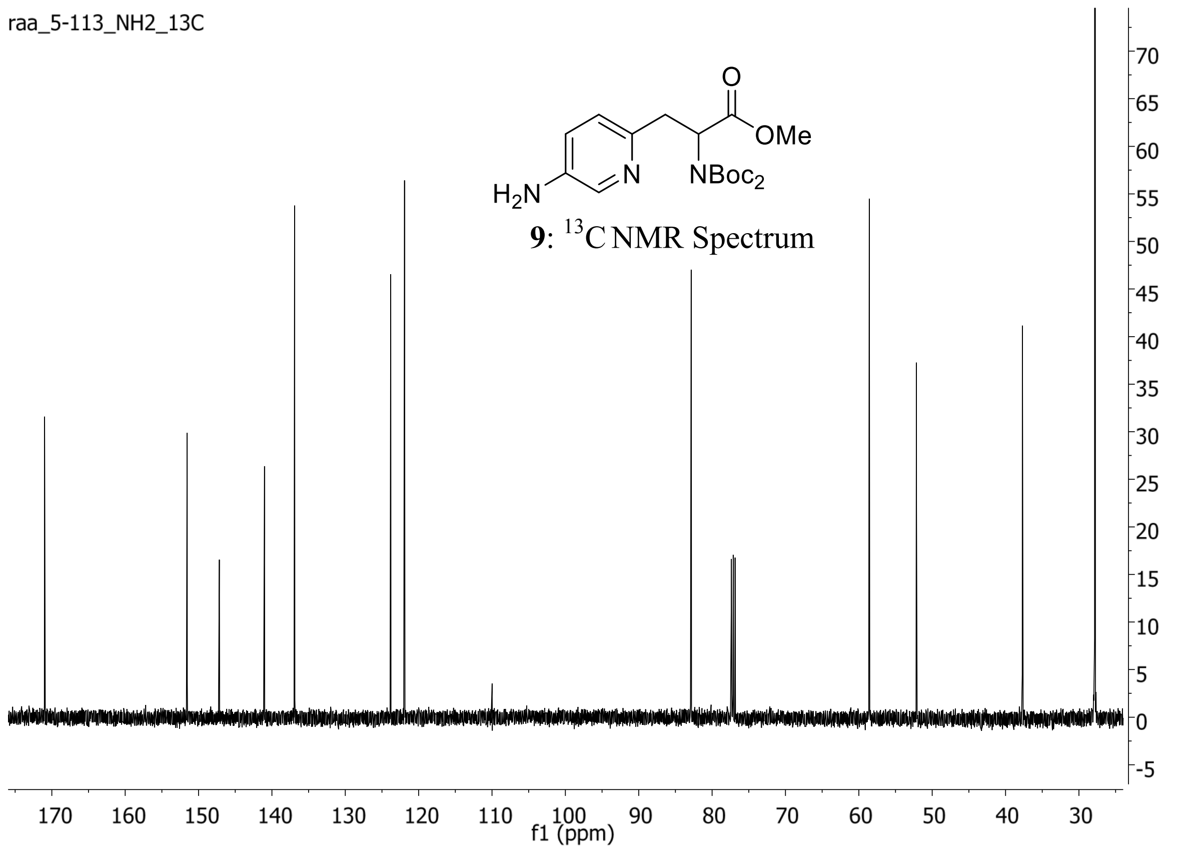
raa_5-113_NH2_13C
O
OMe
H_2N
N
$NBoc_2$
9: ^{13}C NMR Spectrum
f1 (ppm)

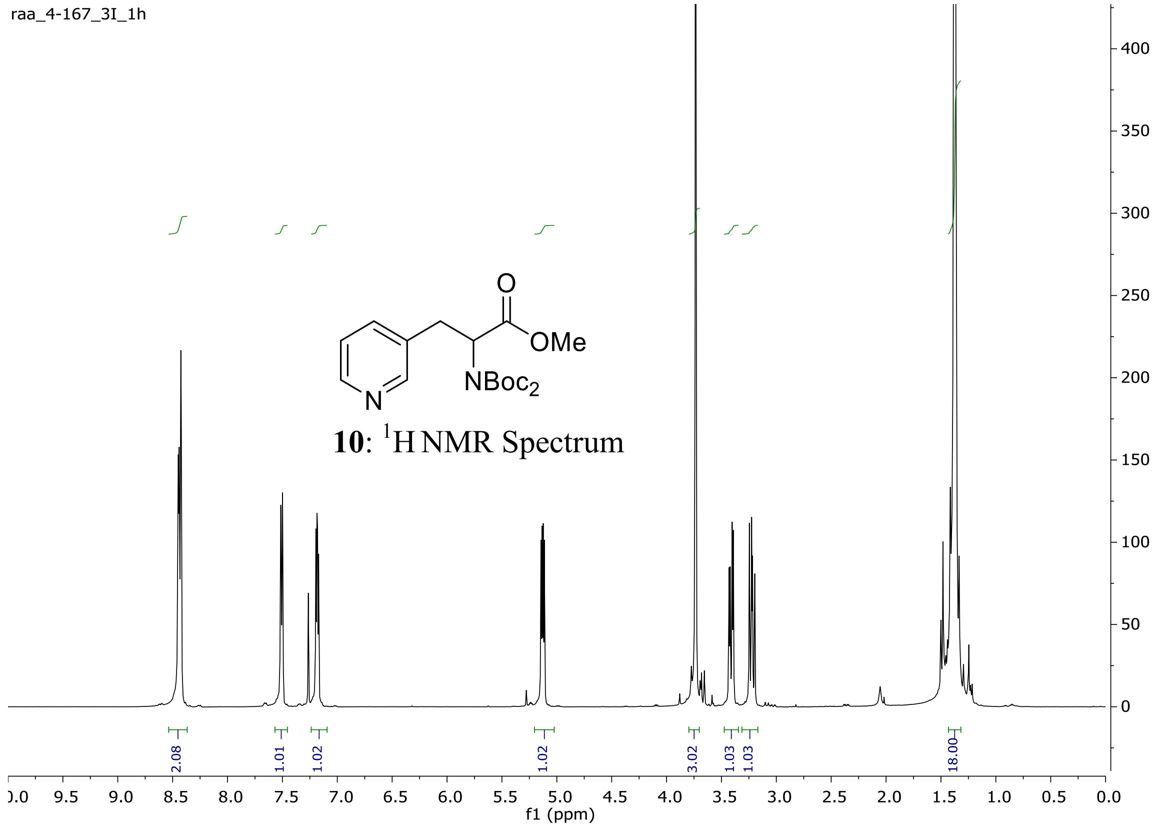
raa_4-167_3I_1h
OMe
O
N
NBoc2
10: 1H NMR Spectrum
2.08
1.01
1.02
1.02
3.02
1.03
1.03
18.00
f1 (ppm)

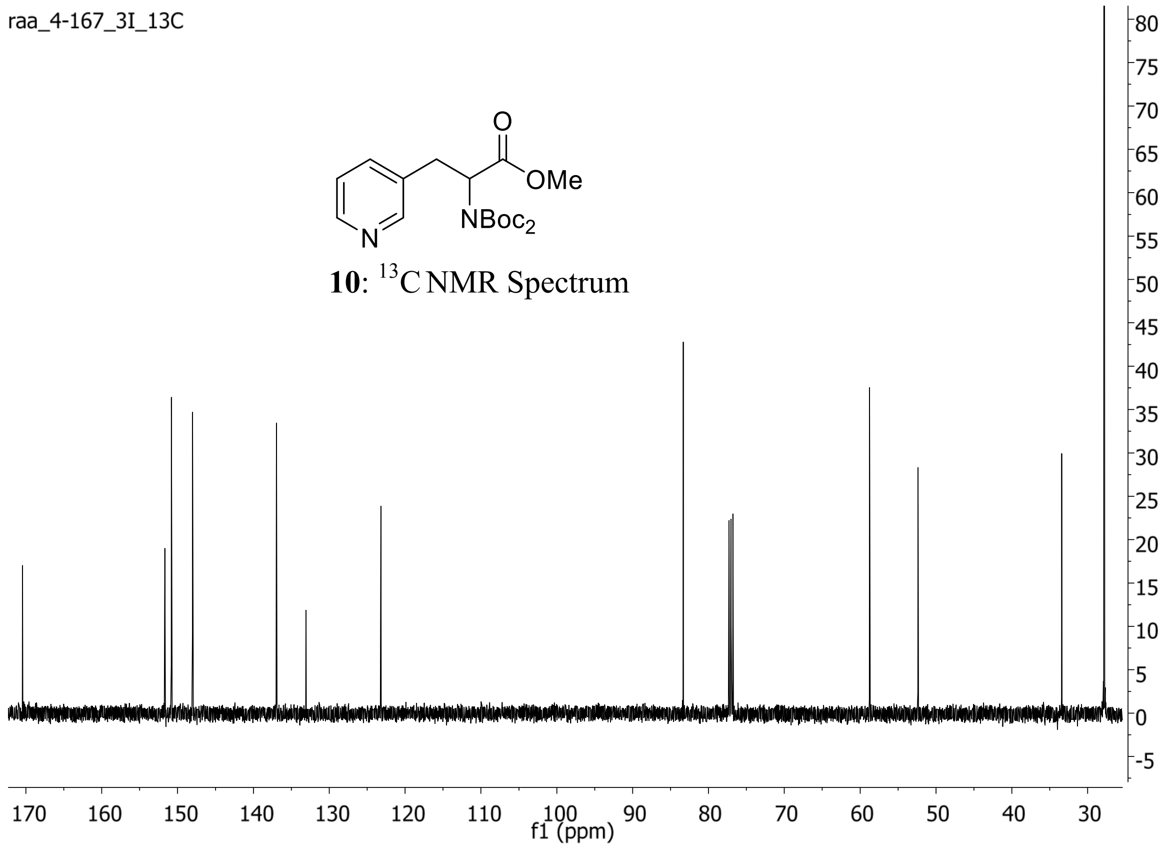
raa_4-167_3I_13C
O
OMe
N
NBoc2
10: 13C NMR Spectrum
f1 (ppm)

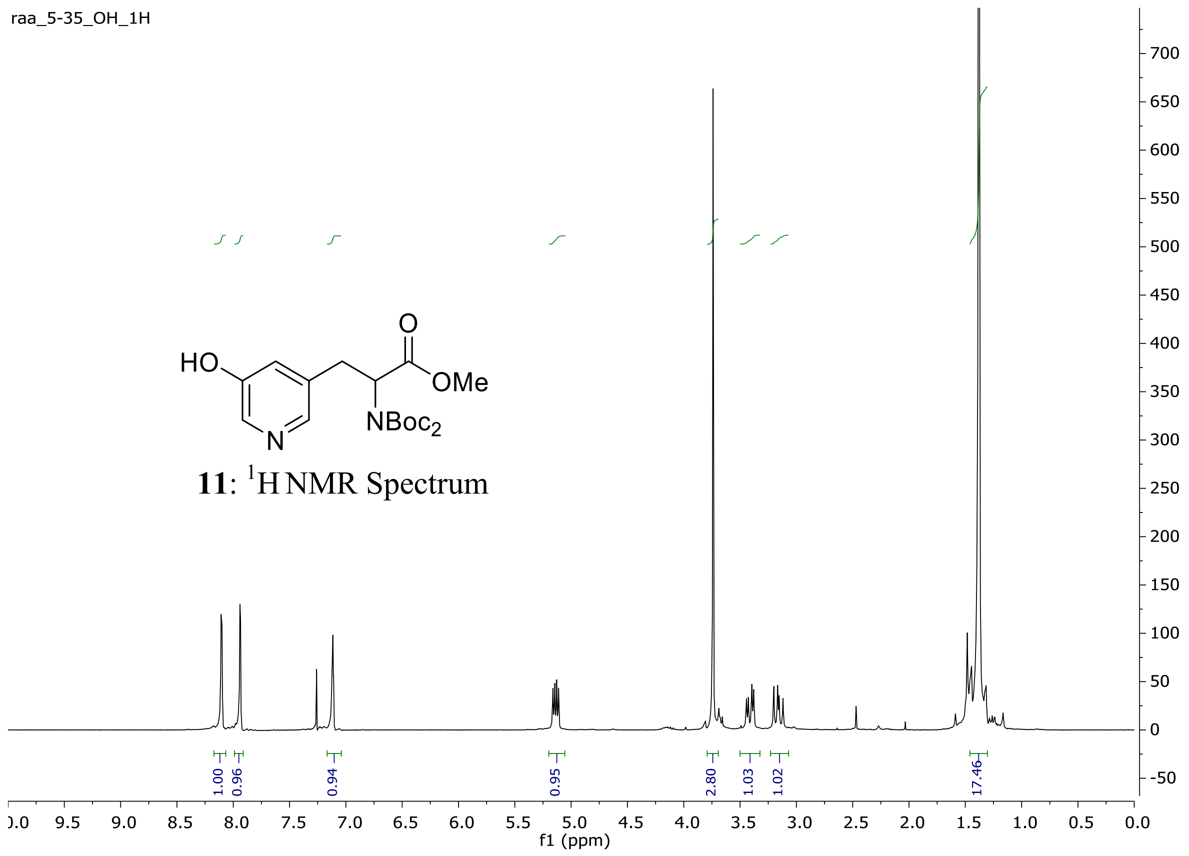
raa_5-35_OH_1H
HO
O
OMe
N
NBoc2
11: 1H NMR Spectrum
f1 (ppm)

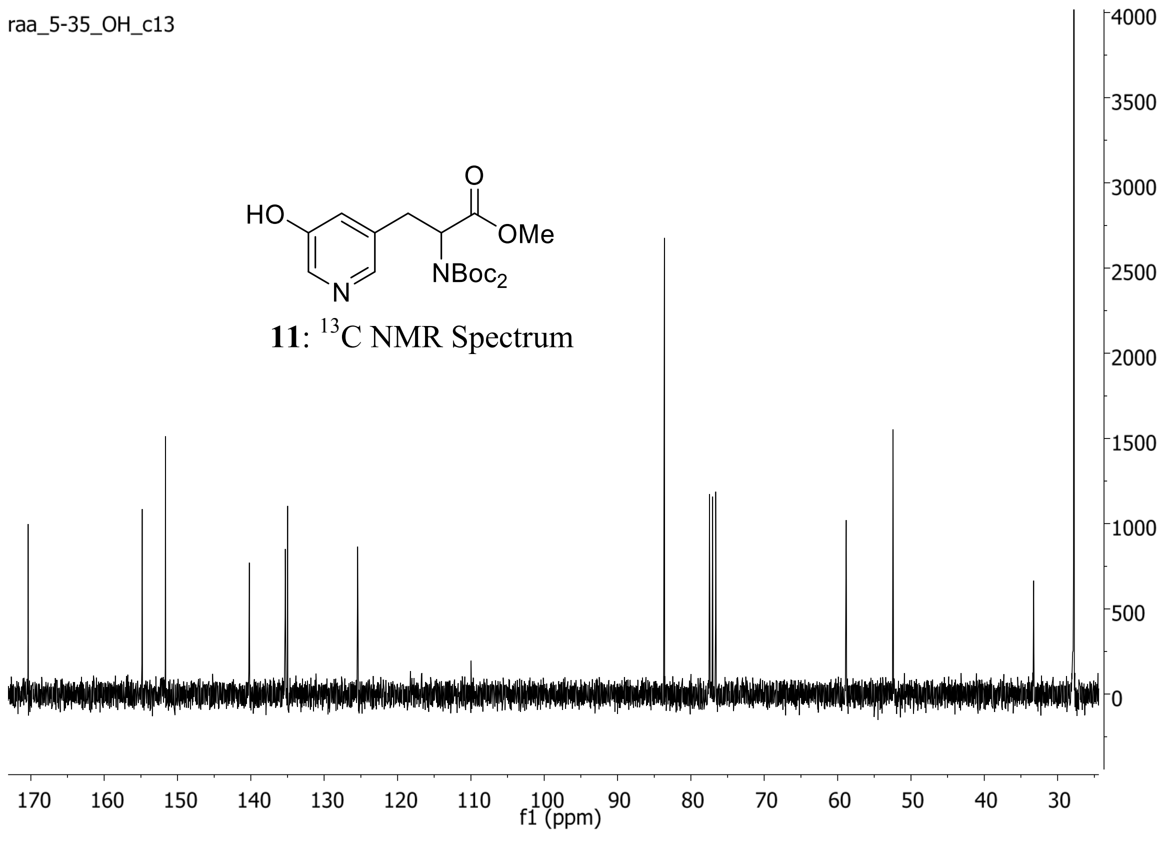
raa_5-35_OH_c13
HO
O
OMe
N
NBoc2
11: 13C NMR Spectrum
f1 (ppm)

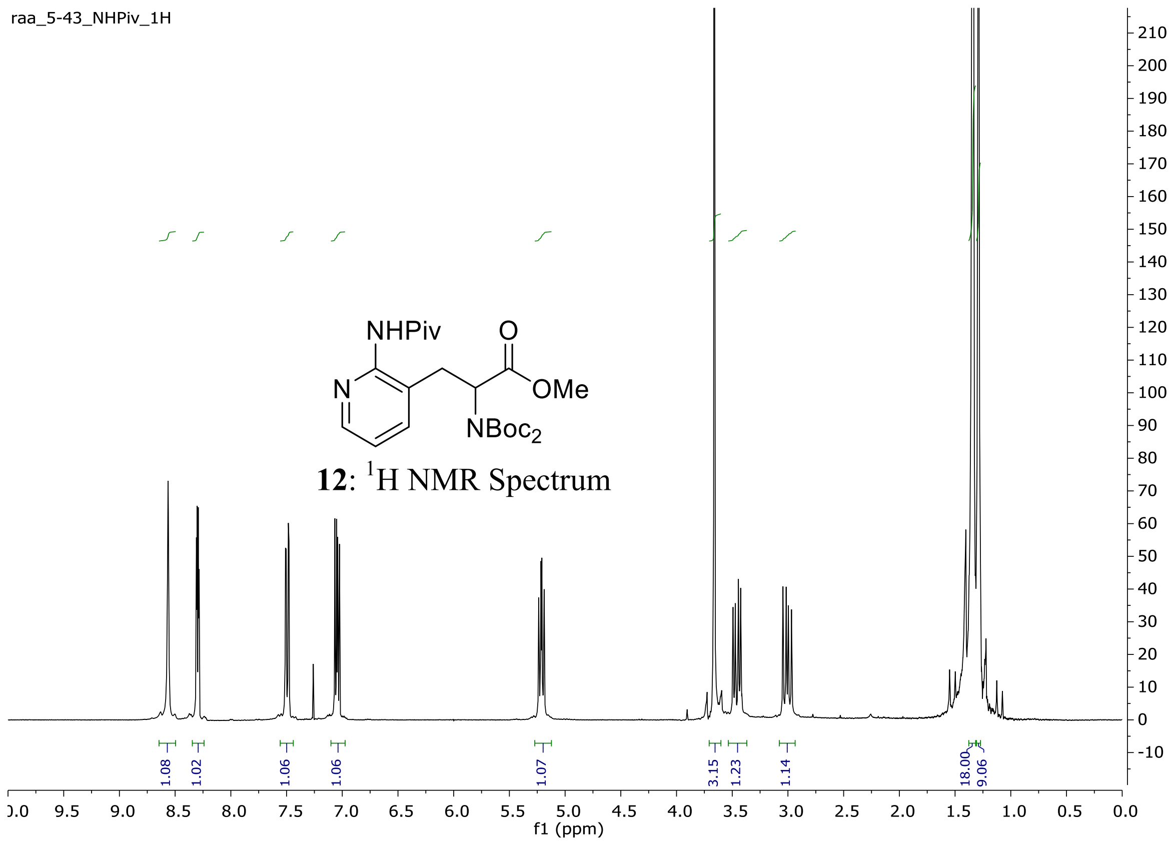

12: ^{1}H NMR Spectrum

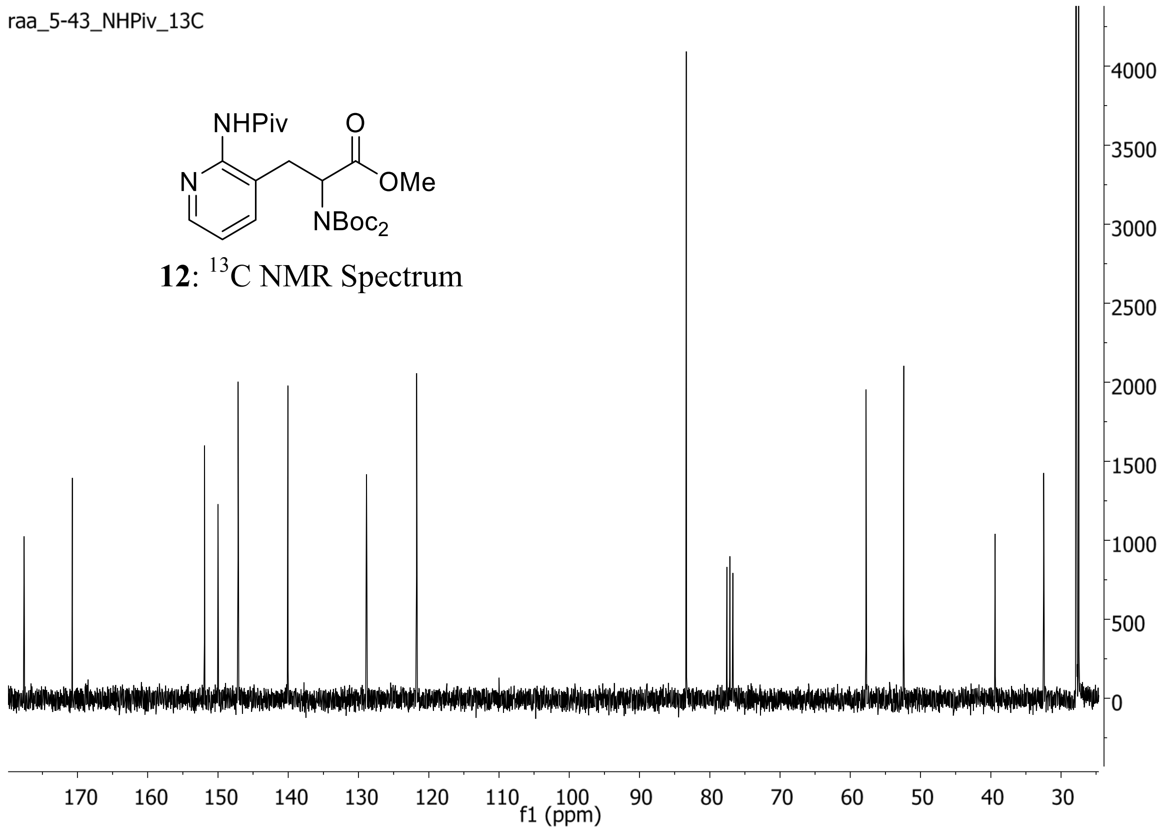

12: ^{13}C NMR Spectrum

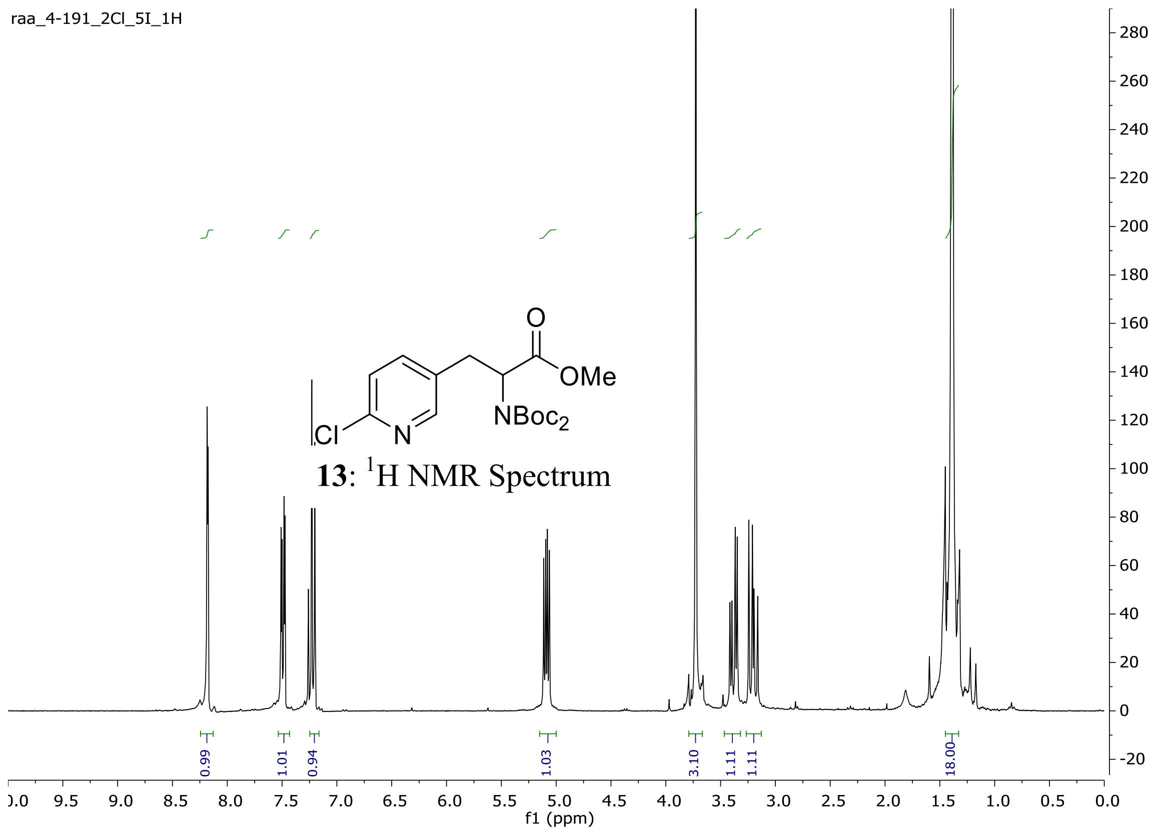
raa_4-191_2Cl_5I_1H
O
OMe
NBoc2
Cl
N
13: 1H NMR Spectrum
0.99
1.01
0.94
1.03
3.10
1.11
1.11
18.00
f1 (ppm)

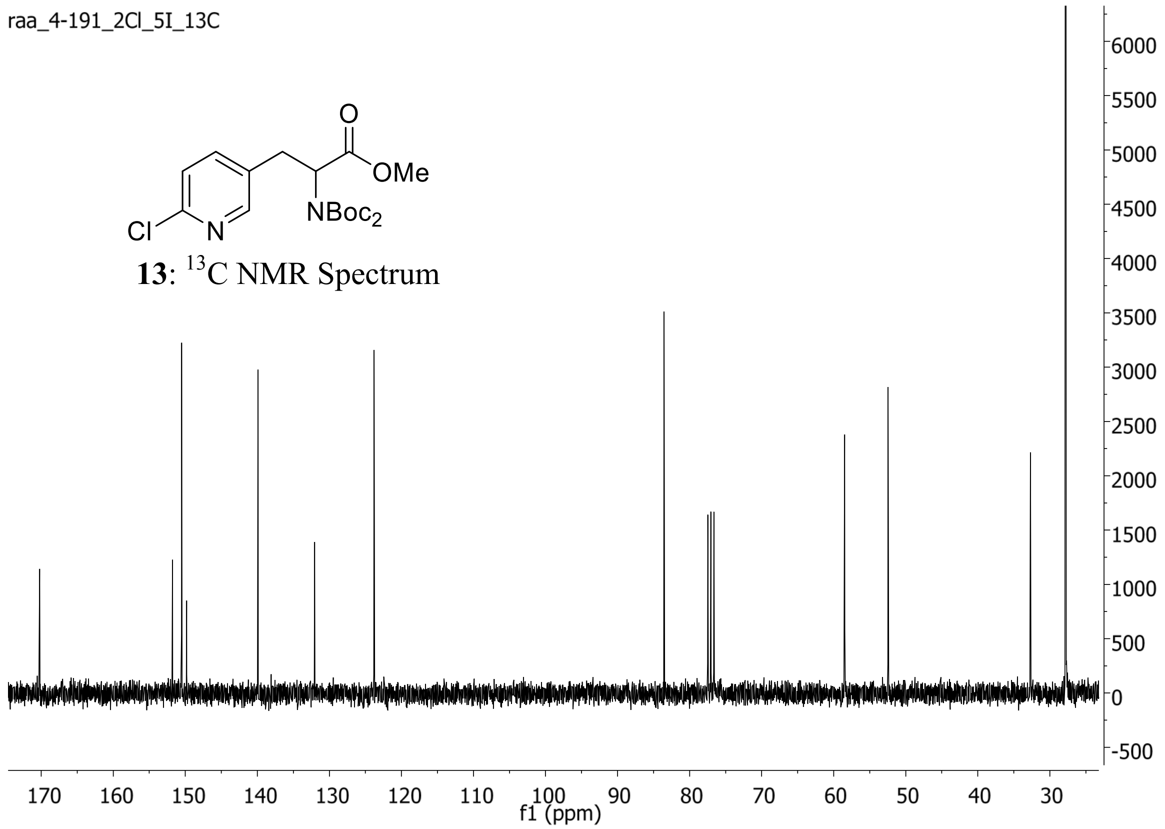
raa_4-191_2Cl_5I_13C
O
OMe
NBoc2
Cl
N
13: 13C NMR Spectrum
f1 (ppm)

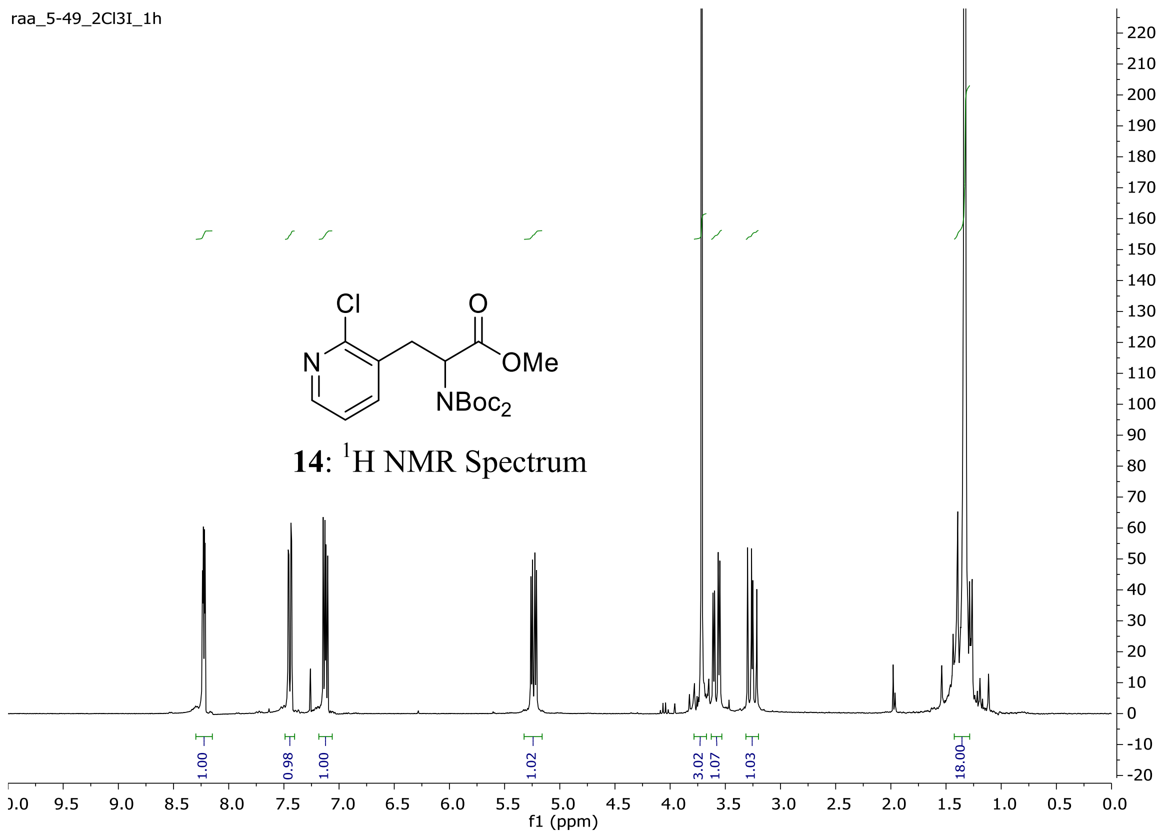
raa_5-49_2Cl3I_1h
Cl
O
N
OMe
NBoc2
14: 1H NMR Spectrum
1.00
0.98
1.00
1.02
3.02
1.07
1.03
18.00
f1 (ppm)

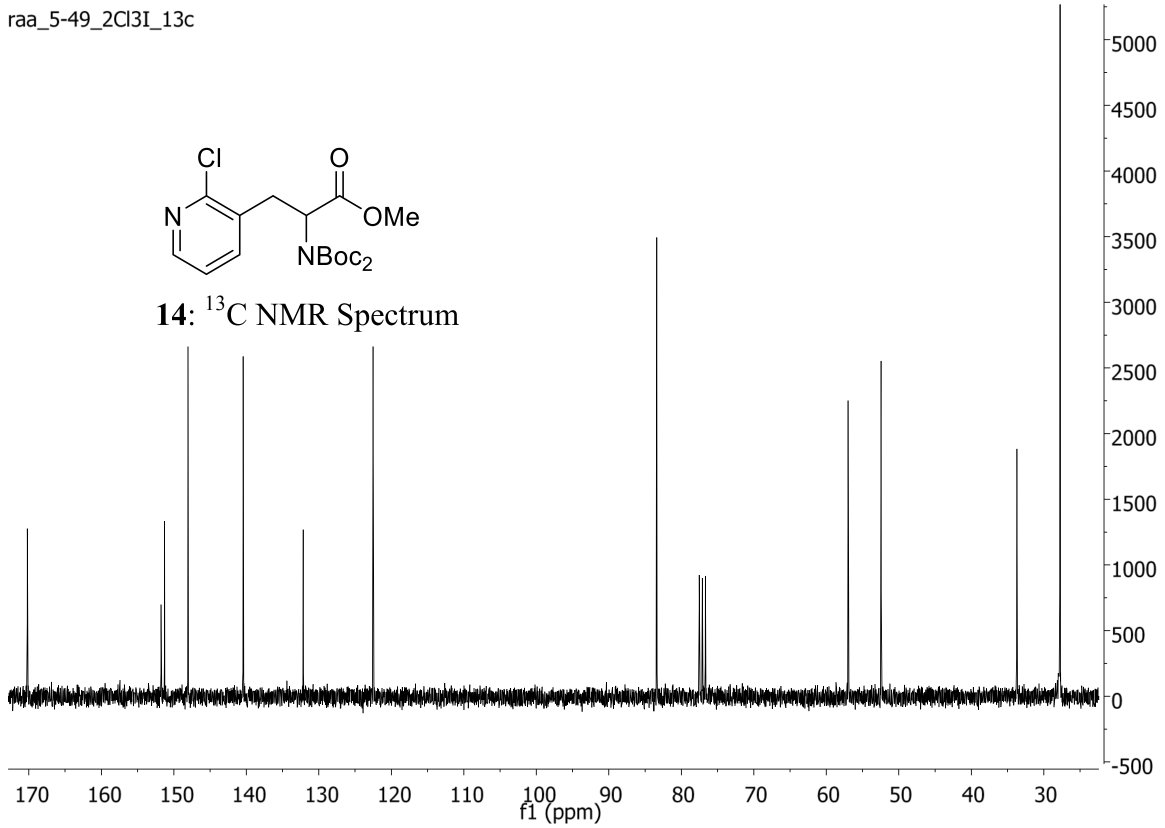
raa_5-49_2Cl3I_13c
Cl
O
N
OMe
NBoc2
14: 13C NMR Spectrum
f1 (ppm)

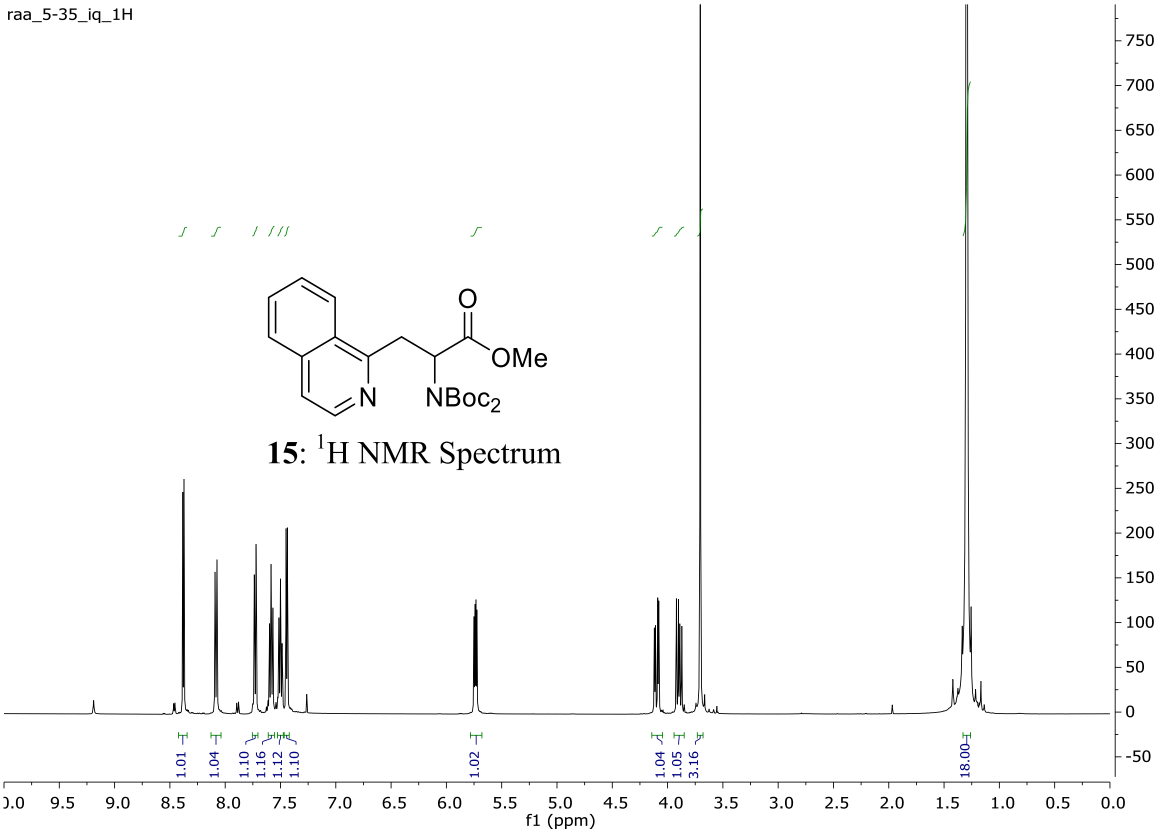

15: ^{1}H NMR Spectrum

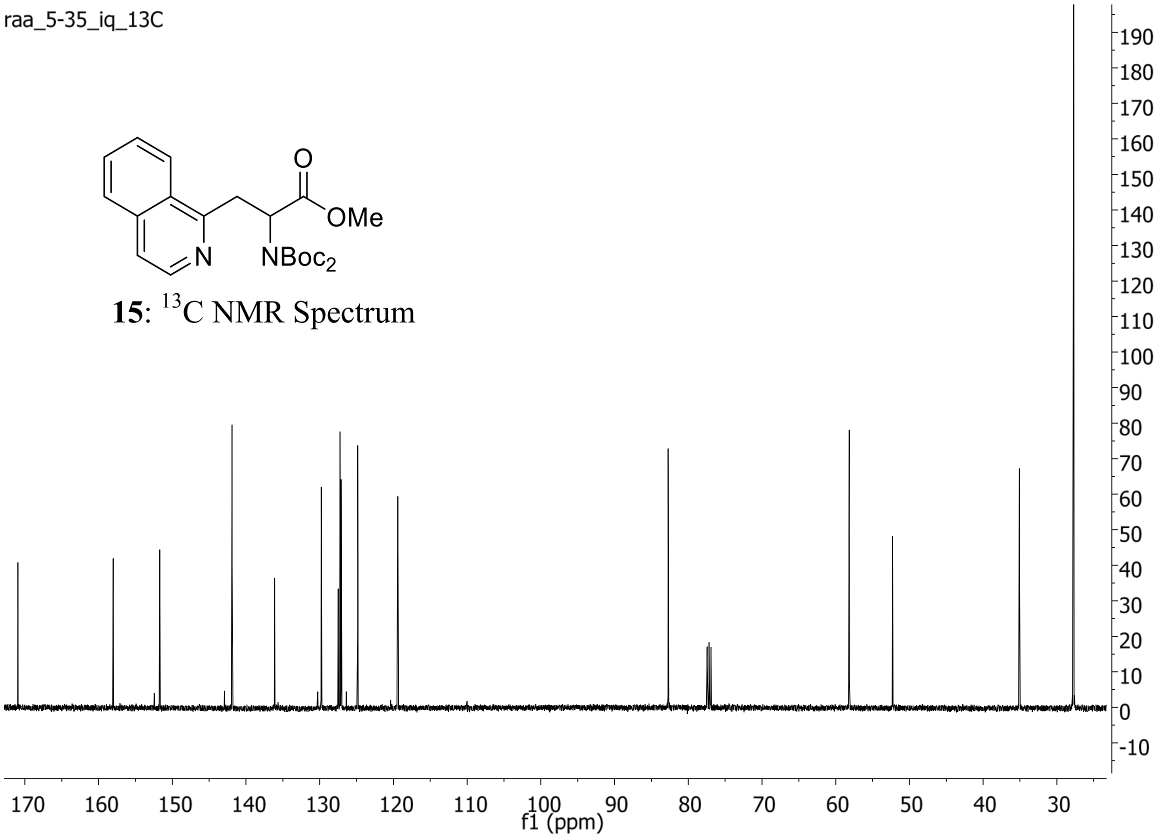

15: ^{13}C NMR Spectrum

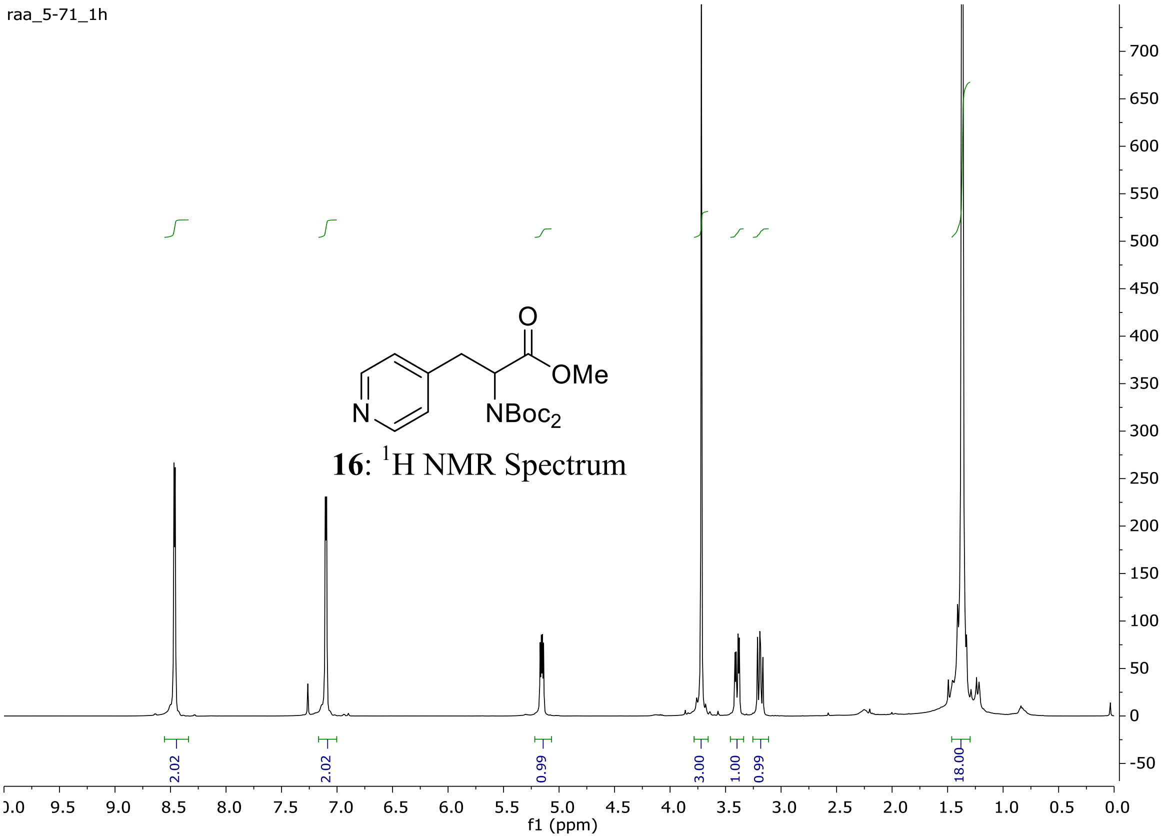
raa_5-71_1h
O
OMe
N
NBoc2
16: 1H NMR Spectrum
2.02
2.02
0.99
3.00
1.00
0.99
18.00
f1 (ppm)

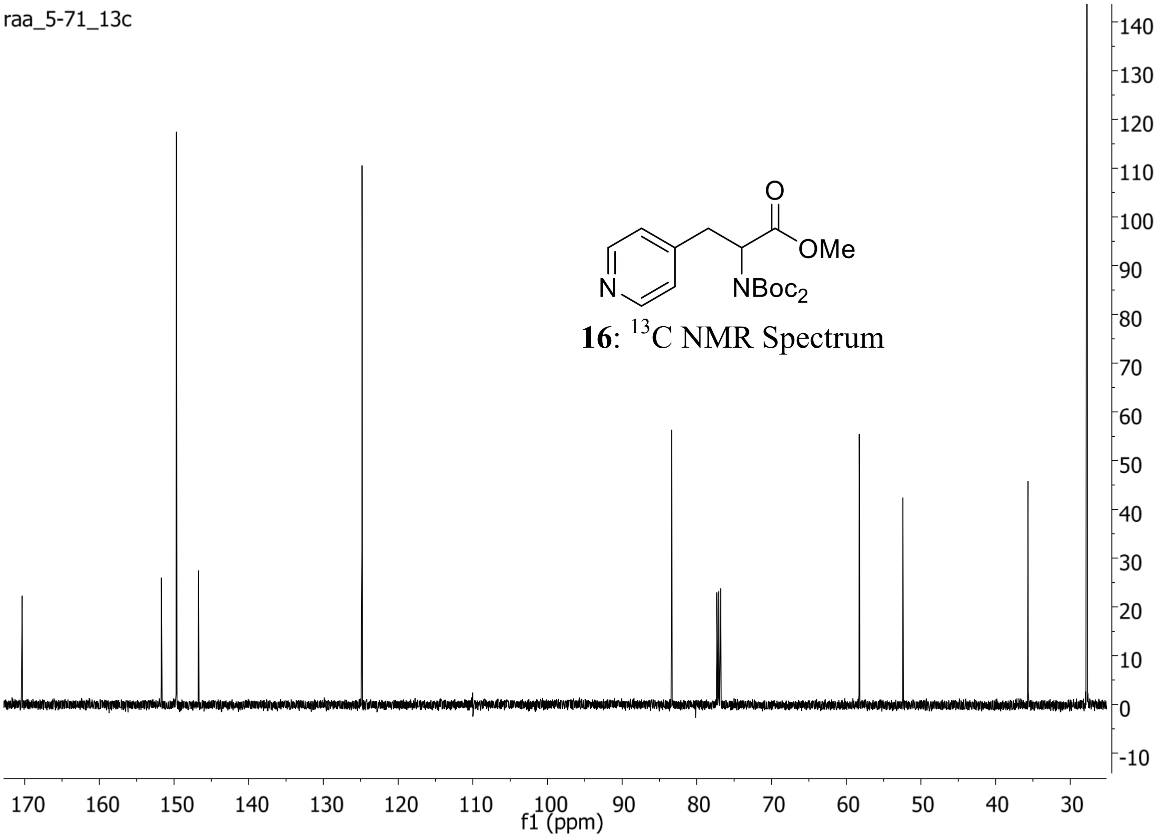
raa_5-71_13c
O
OMe
N
NBoc2
16: 13C NMR Spectrum
f1 (ppm)

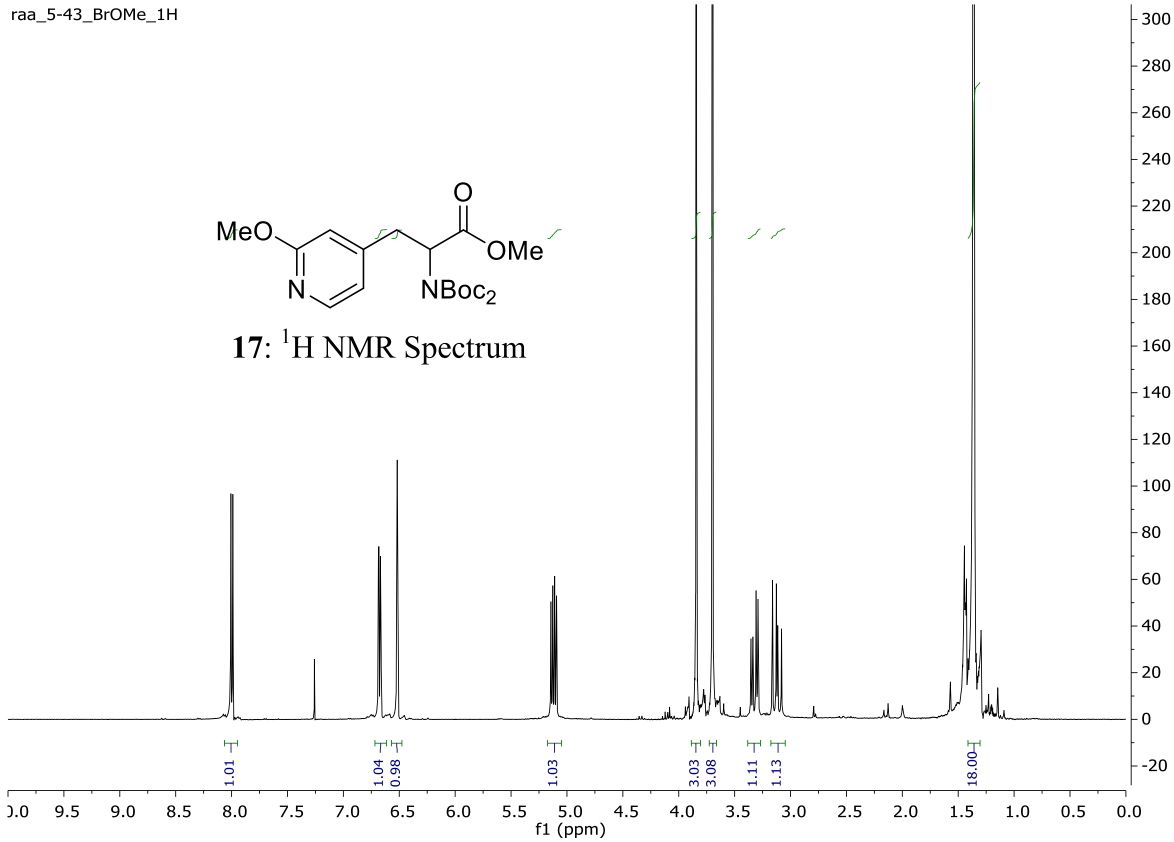

17: ^{1}H NMR Spectrum

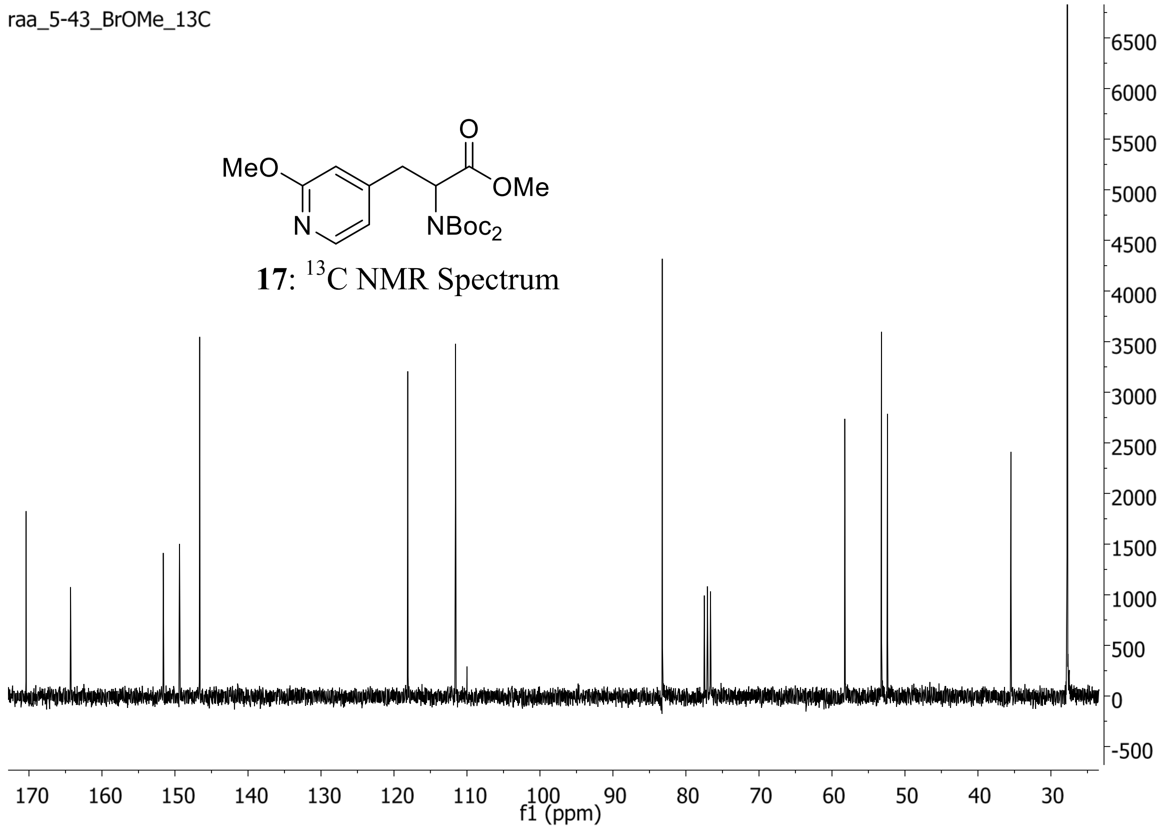

17: ^{13}C NMR Spectrum

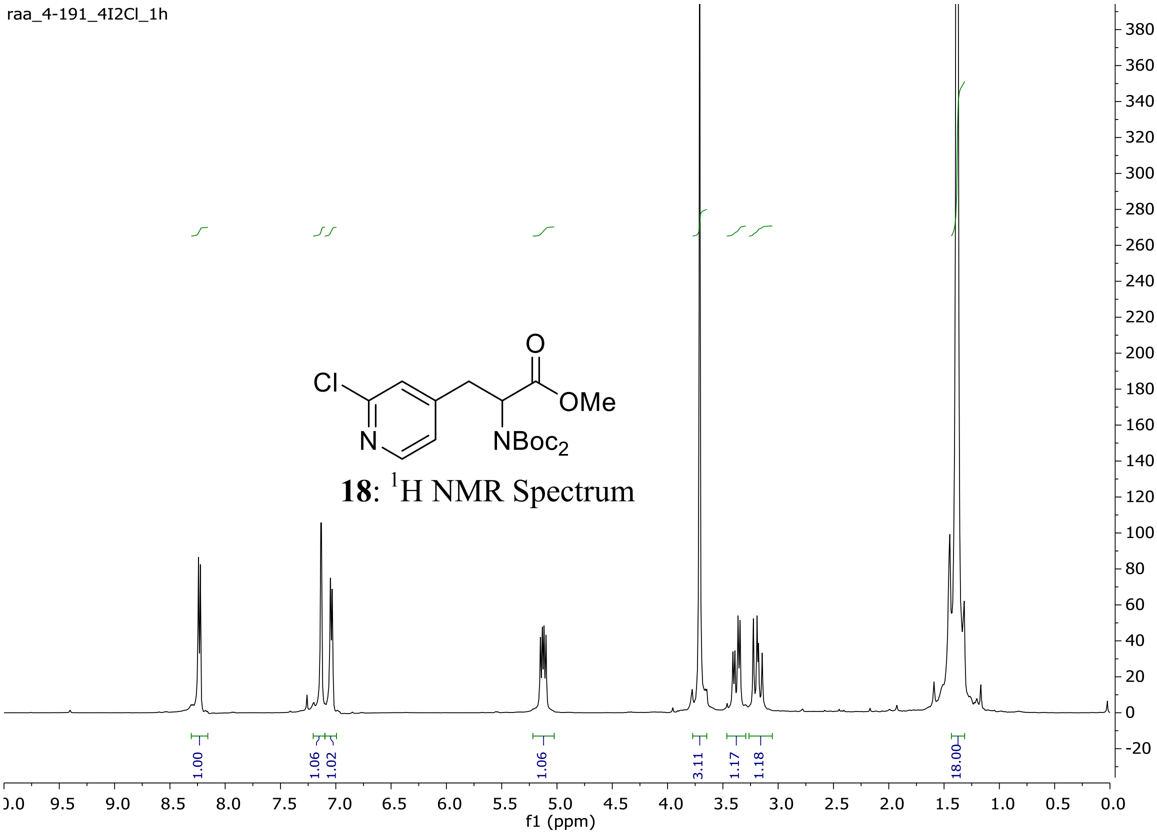
raa_4-191_4I2Cl_1h
Cl
N
O
OMe
NBoc2
18: 1H NMR Spectrum
1.00
1.06
1.02
1.06
3.11
1.17
1.18
18.00
f1 (ppm)

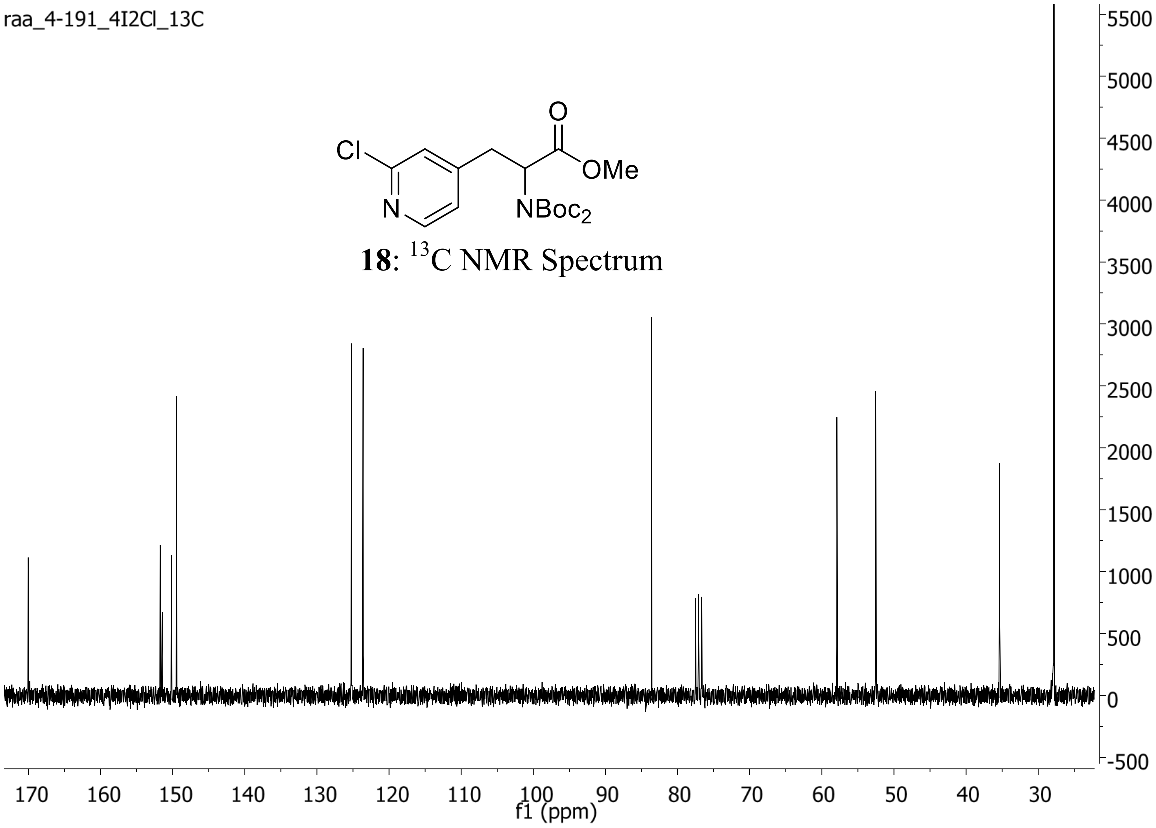
raa_4-191_4I2Cl_13C
Cl
N
O
OMe
NBoc2
18: 13C NMR Spectrum
f1 (ppm)

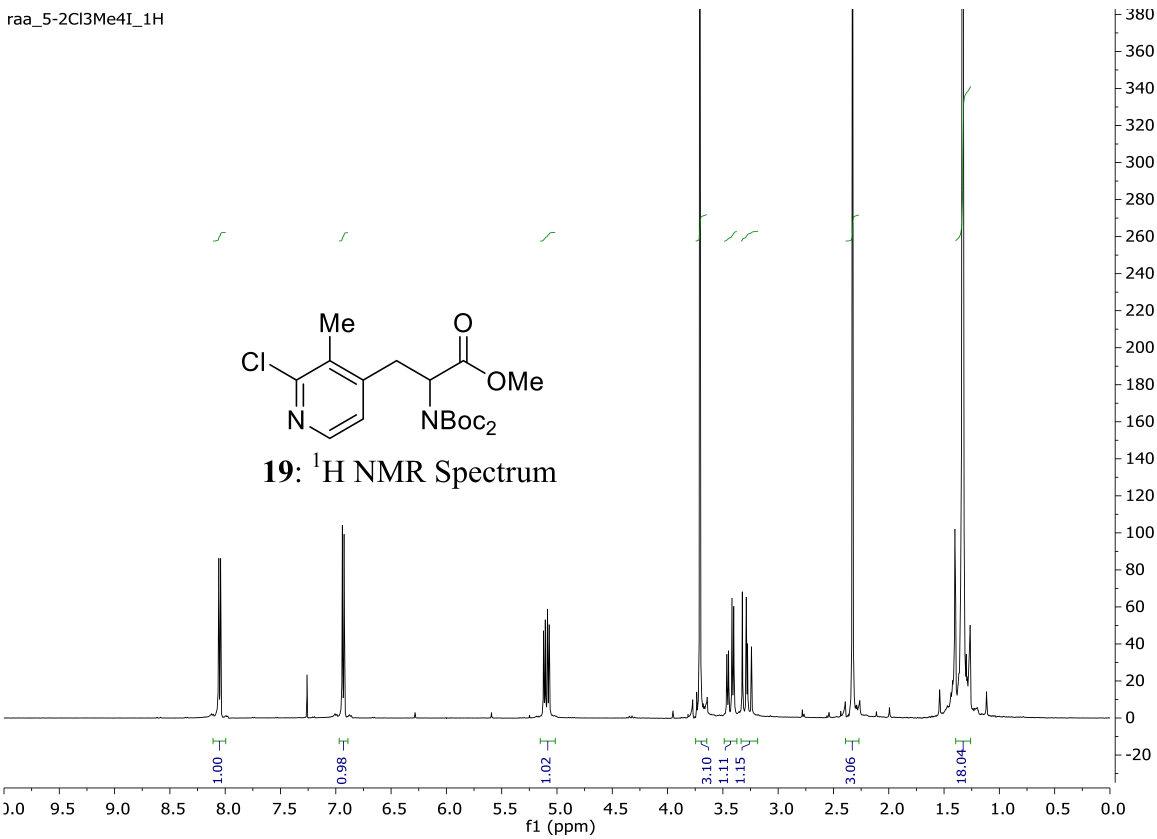

19: ^{1}H NMR Spectrum

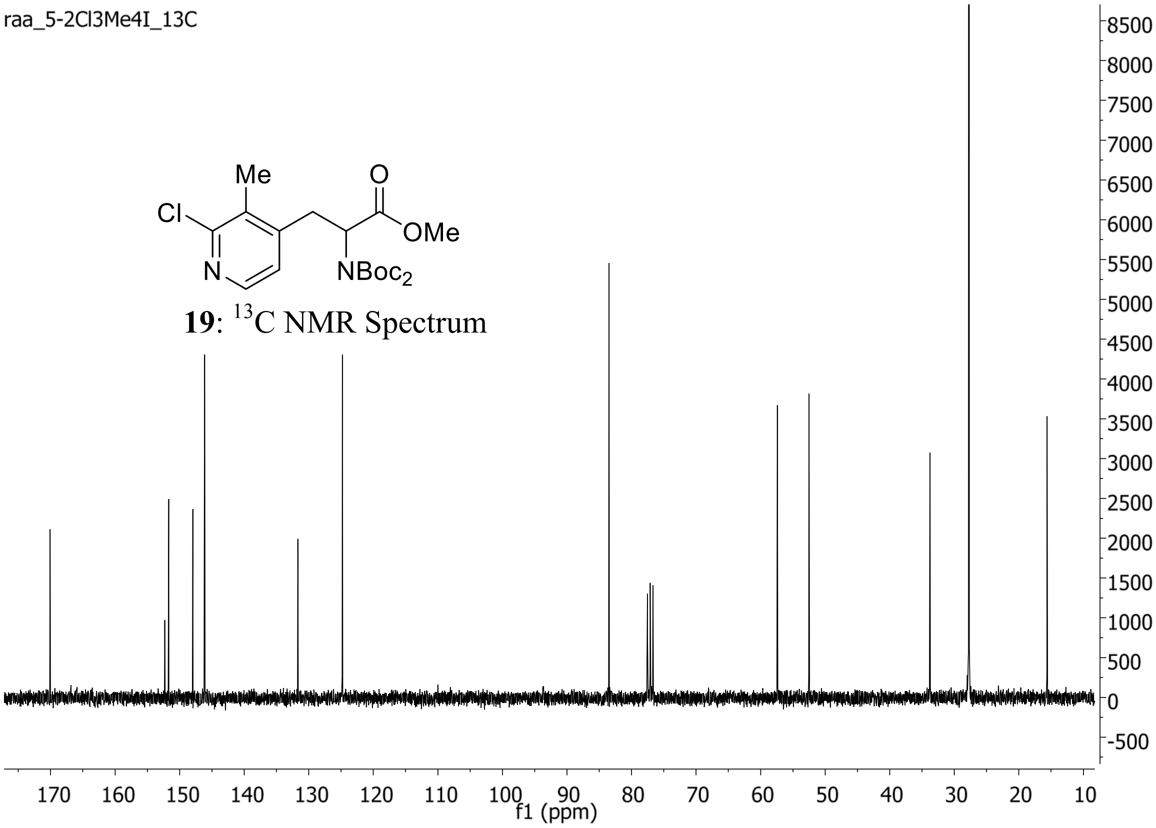

19: ^{13}C NMR Spectrum

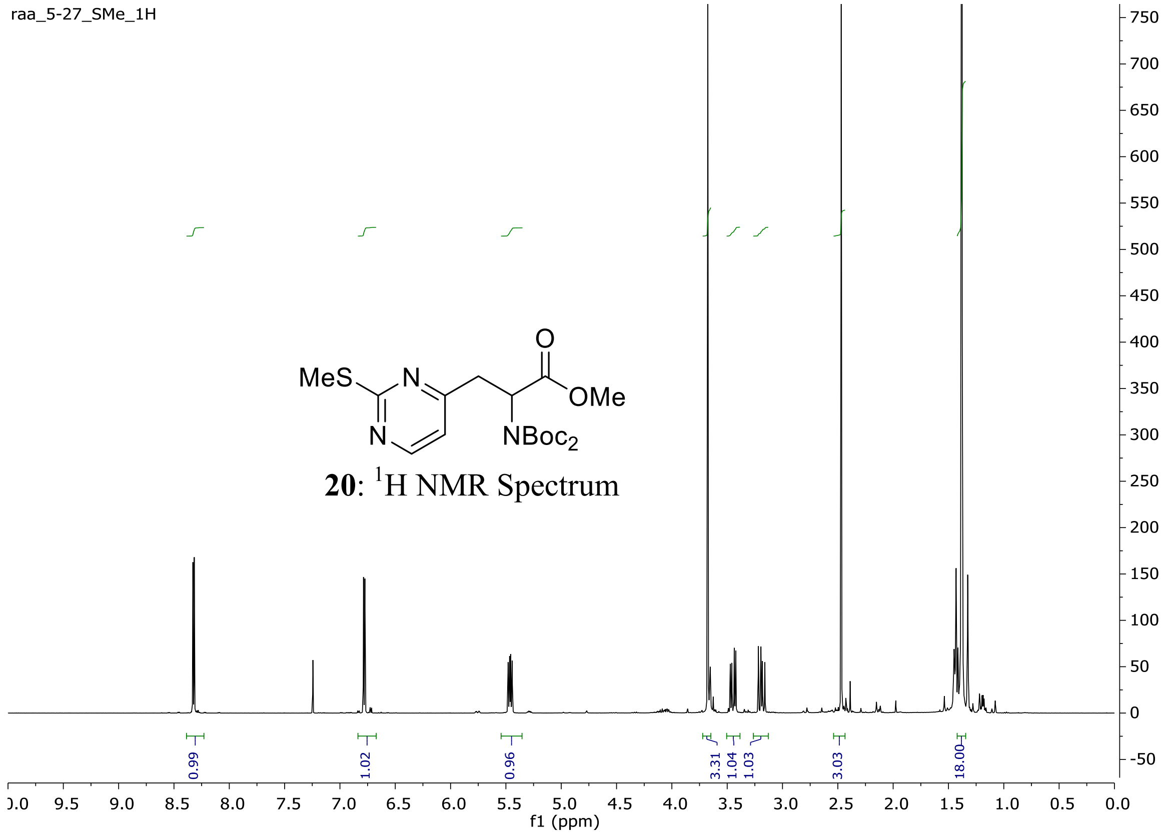
raa_5-27_SMe_1H
MeS
N
N
O
OMe
NBoc2
20: 1H NMR Spectrum
0.99
1.02
0.96
3.31
1.04
1.03
3.03
18.00
f1 (ppm)

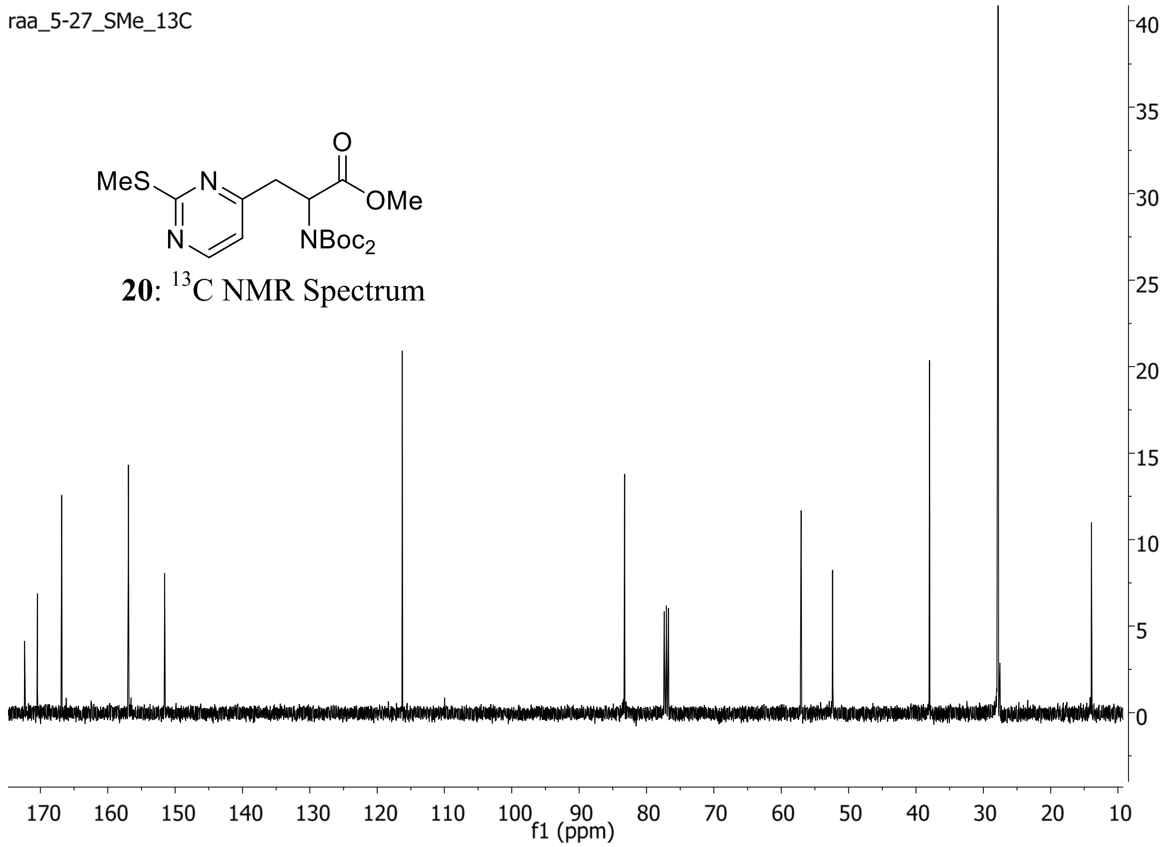
raa_5-27_SMe_13C
MeS
N
N
O
OMe
NBoc2
20: 13C NMR Spectrum
f1 (ppm)

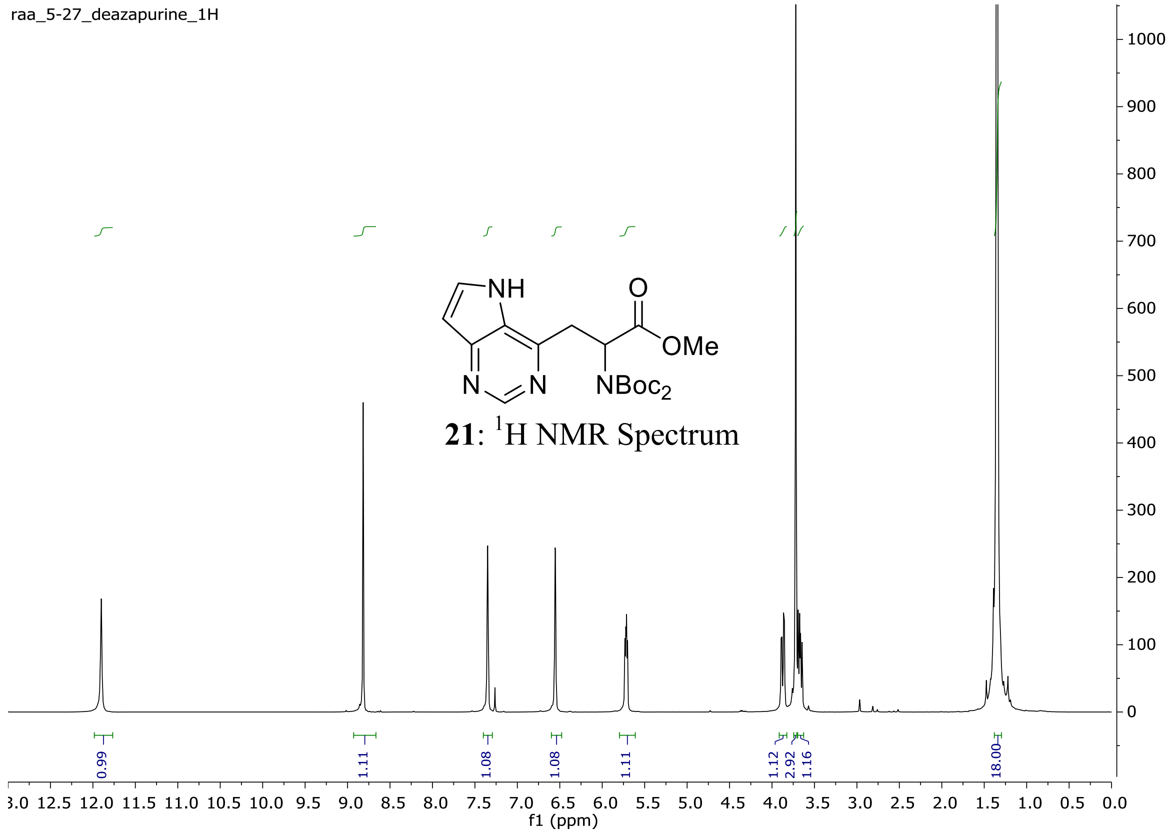

21: ^{1}H NMR Spectrum

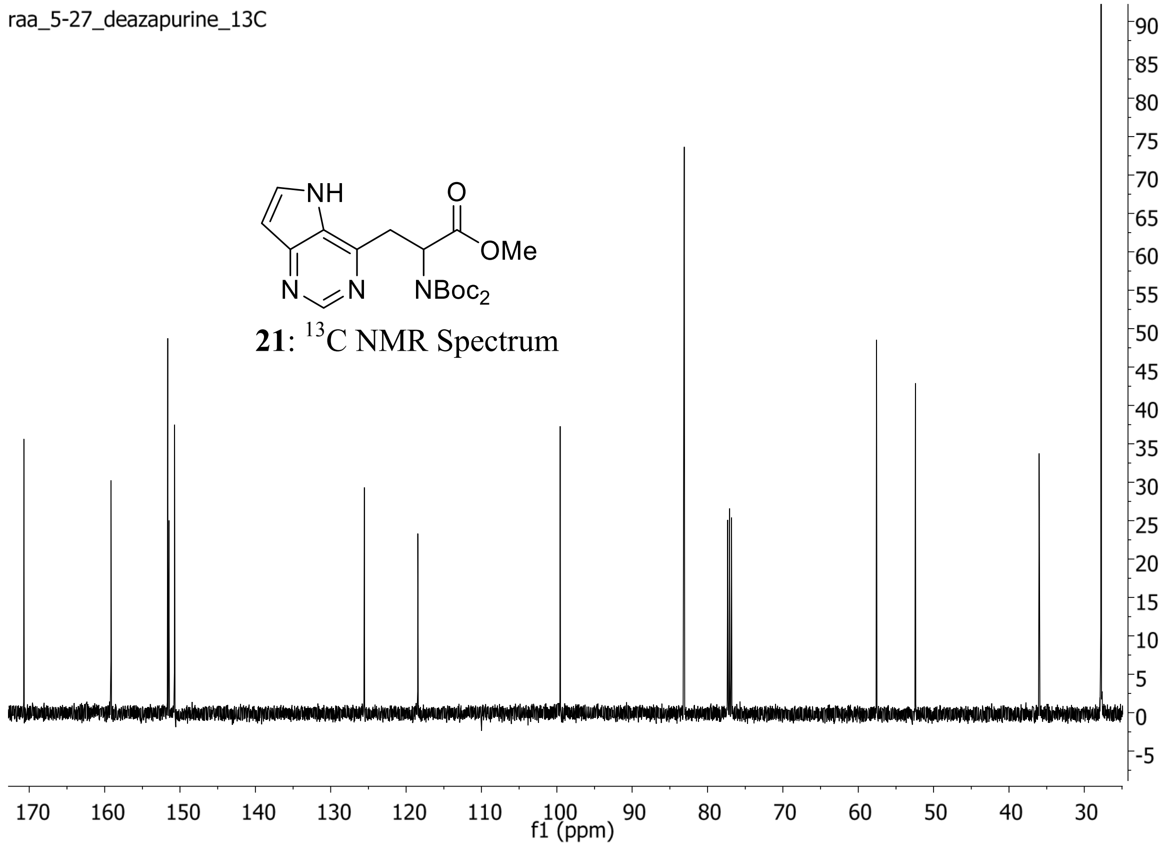

21: ^{13}C NMR Spectrum

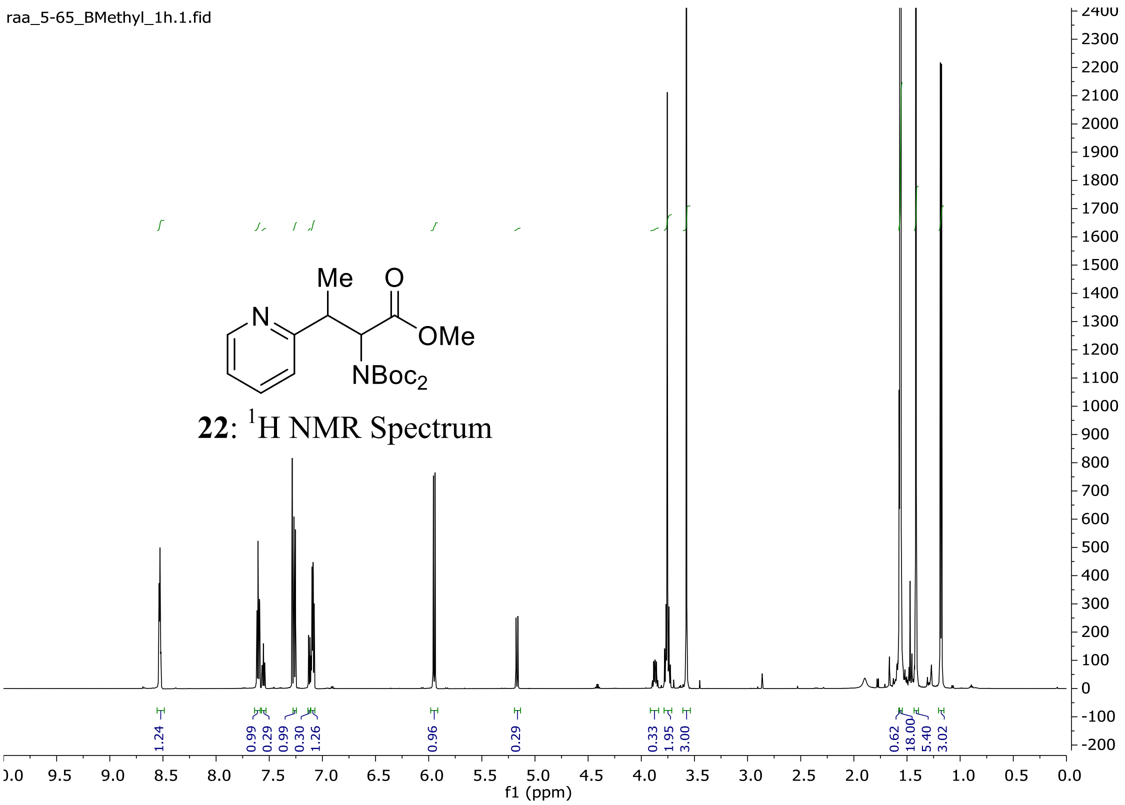
raa_5-65_BMethyl_1h.1.fid
Me
O
N
OMe
NBoc2
22: 1H NMR Spectrum
1.24
0.99
0.29
0.99
0.30
1.26
0.96
0.29
0.33
1.95
3.00
0.62
18.00
5.40
3.02
f1 (ppm)

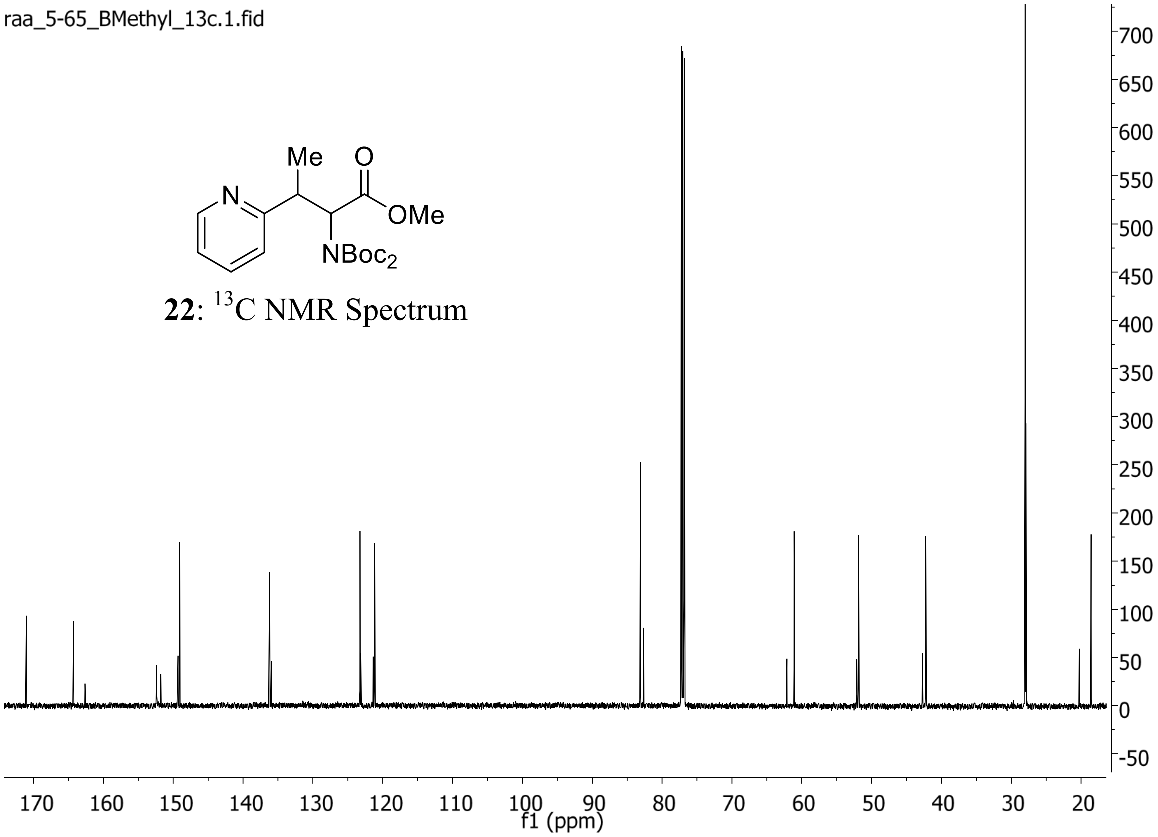
raa_5-65_BMethyl_13c.1.fid
Me
O
N
OMe
NBoc2
22: 13C NMR Spectrum
f1 (ppm)

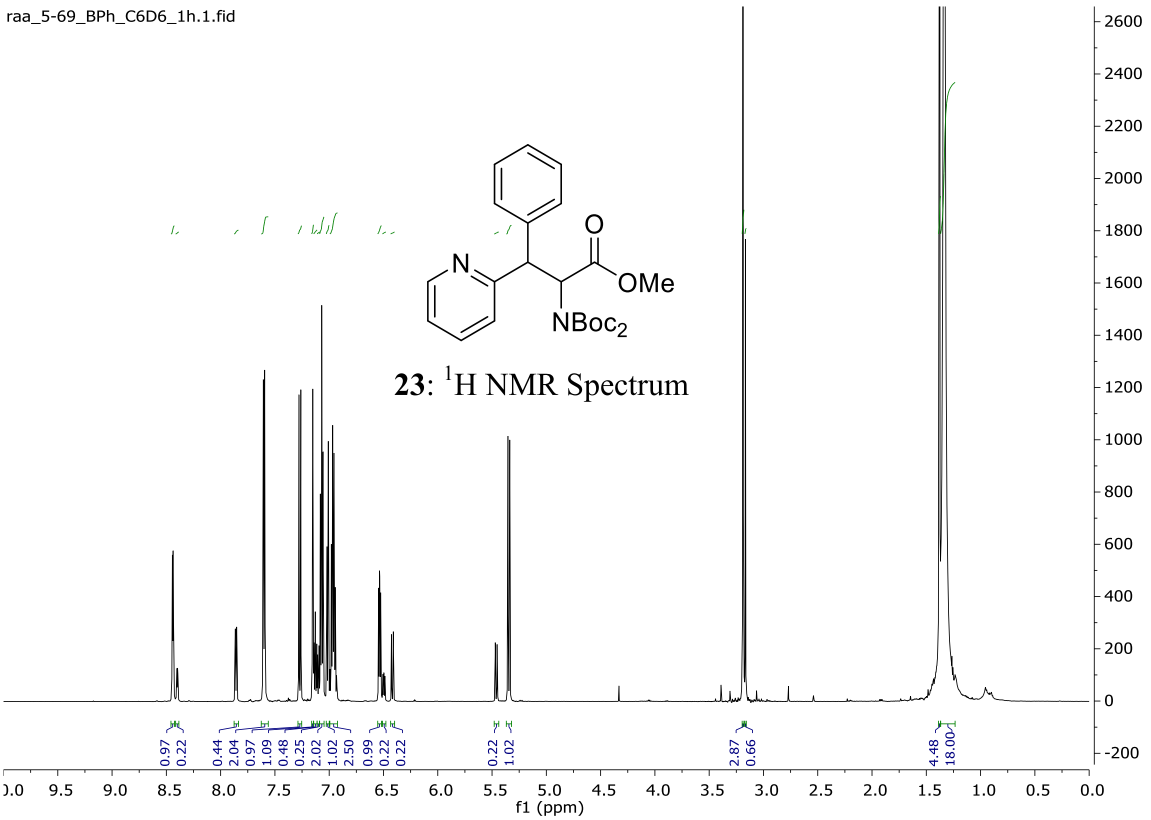
raa_5-69_BPh_C6D6_1h.1.fid
OMe
NBoc2
23: 1H NMR Spectrum
f1 (ppm)

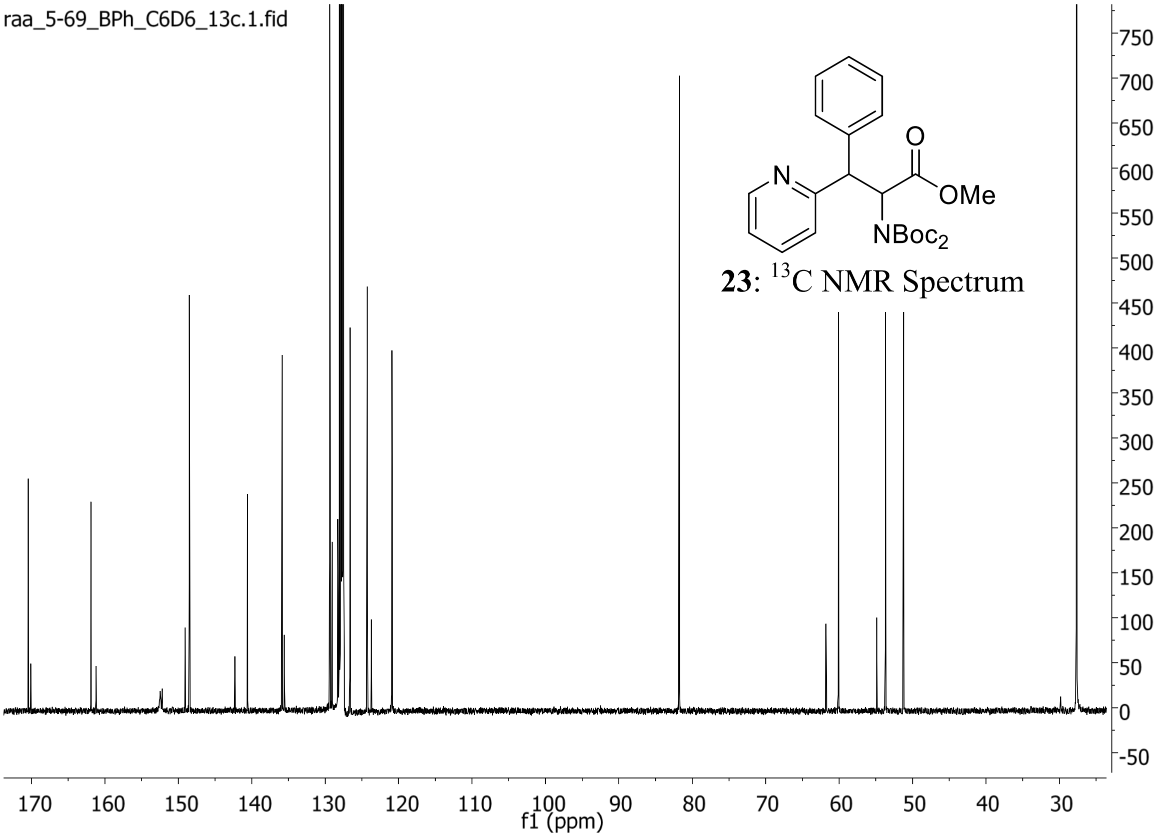
raa_5-69_BPh_C6D6_13c.1.fid
OMe
NBoc2
23: 13C NMR Spectrum
f1 (ppm)

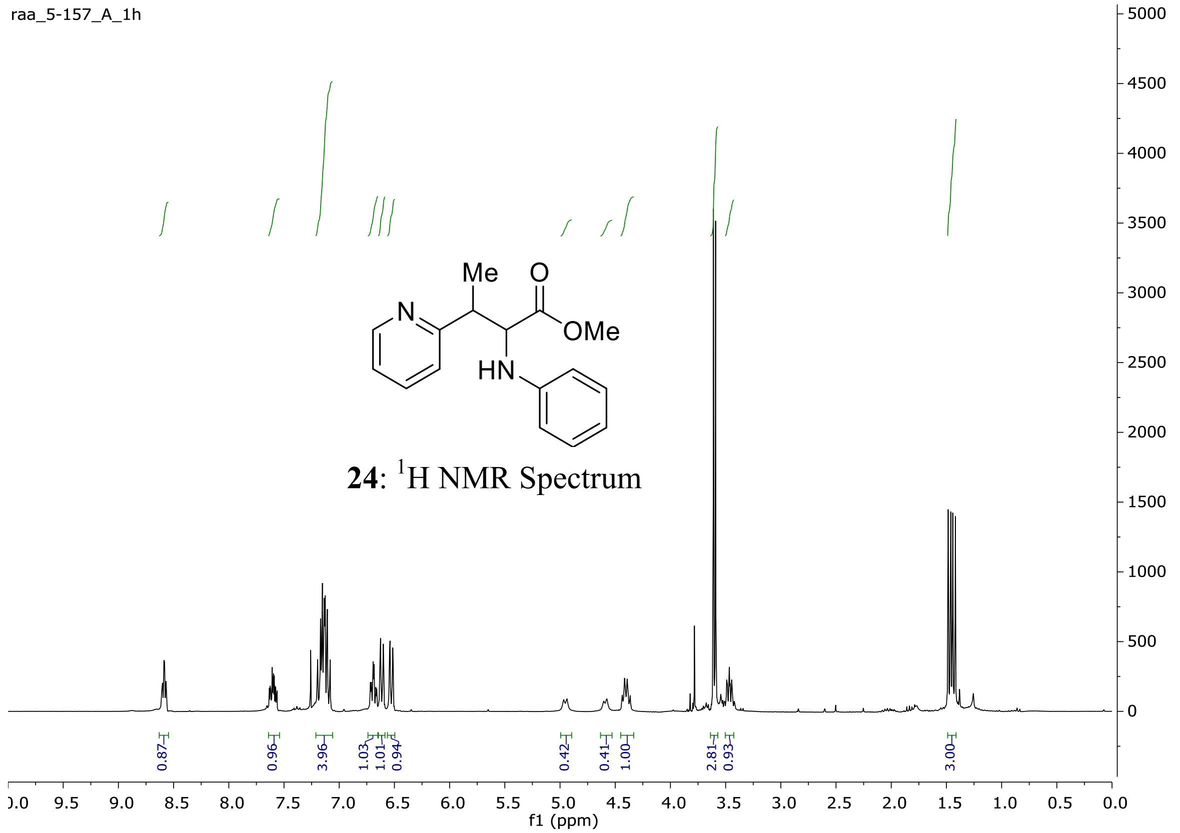
raa_5-157_A_1h
Me
O
N
OMe
HN
24: ¹H NMR Spectrum
f1 (ppm)

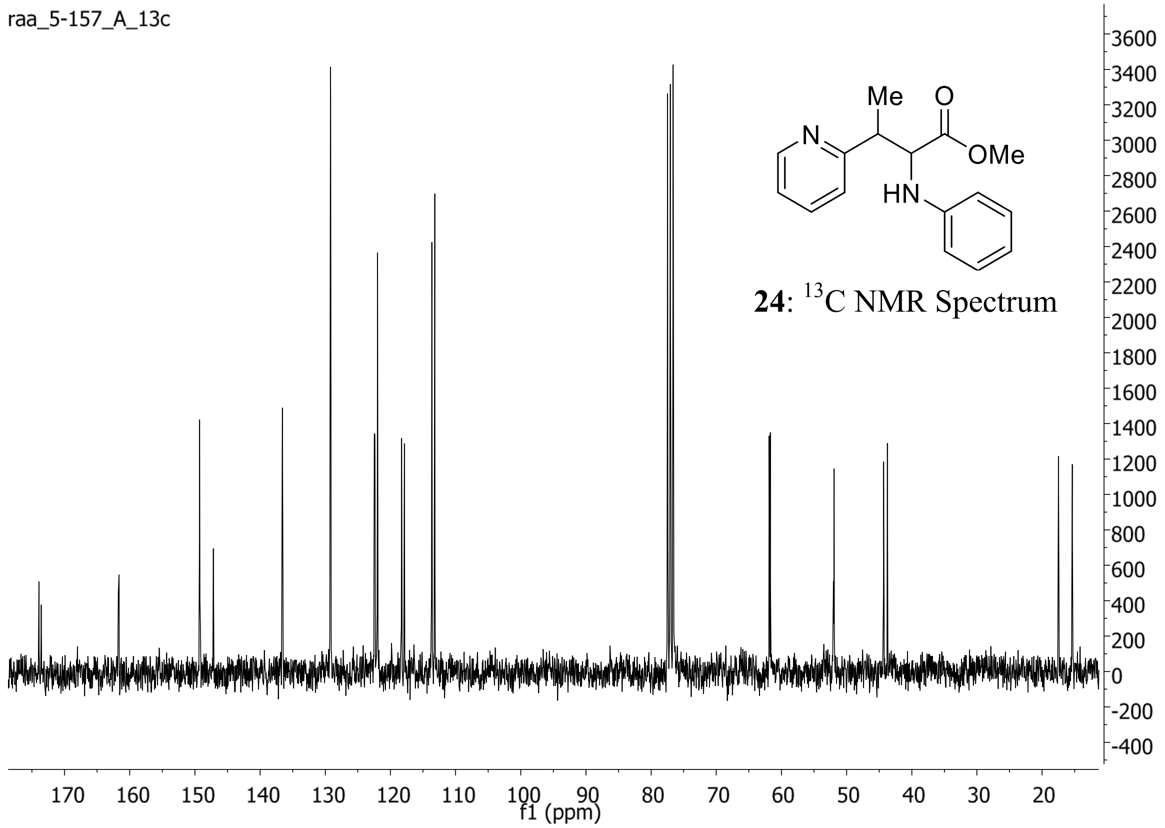
raa_5-157_A_13c
Me
O
N
OMe
HN
24: ¹³C NMR Spectrum
f1 (ppm)

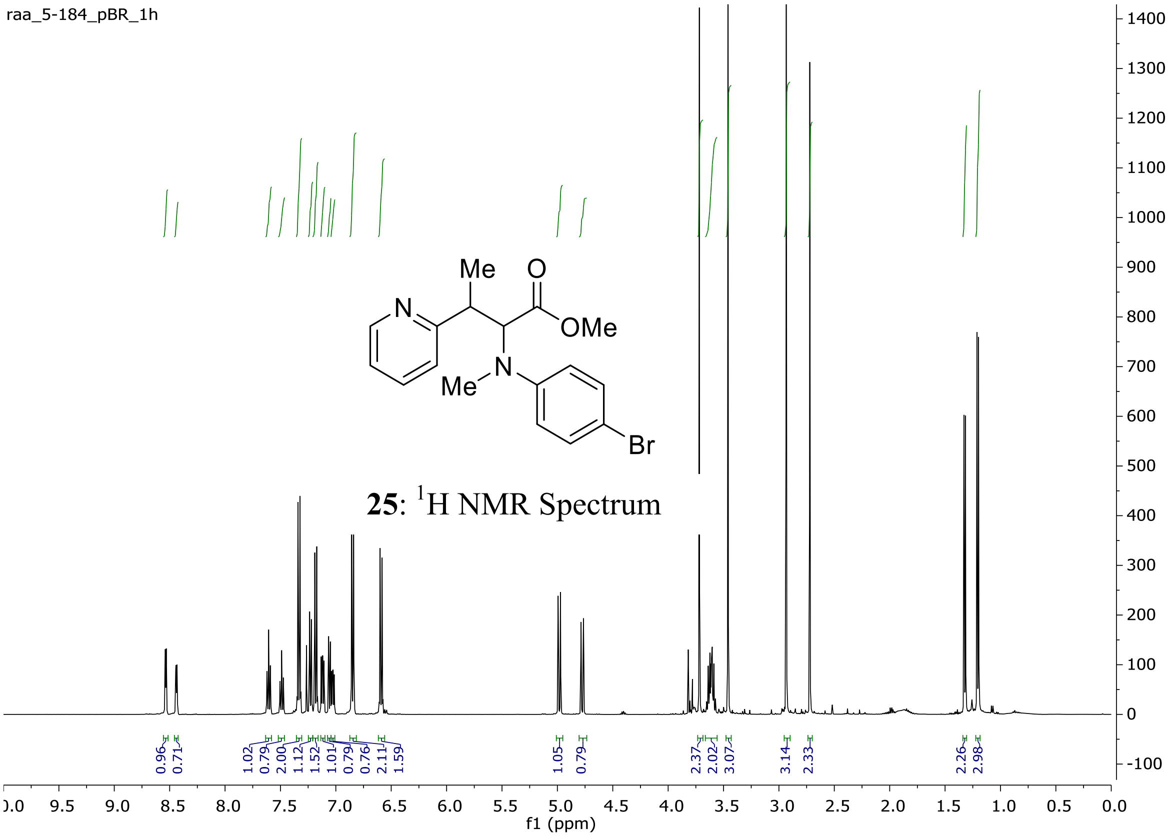
raa_5-184_pBR_1h
Me
O
N
OMe
Me
N
Br
25: 1H NMR Spectrum
f1 (ppm)

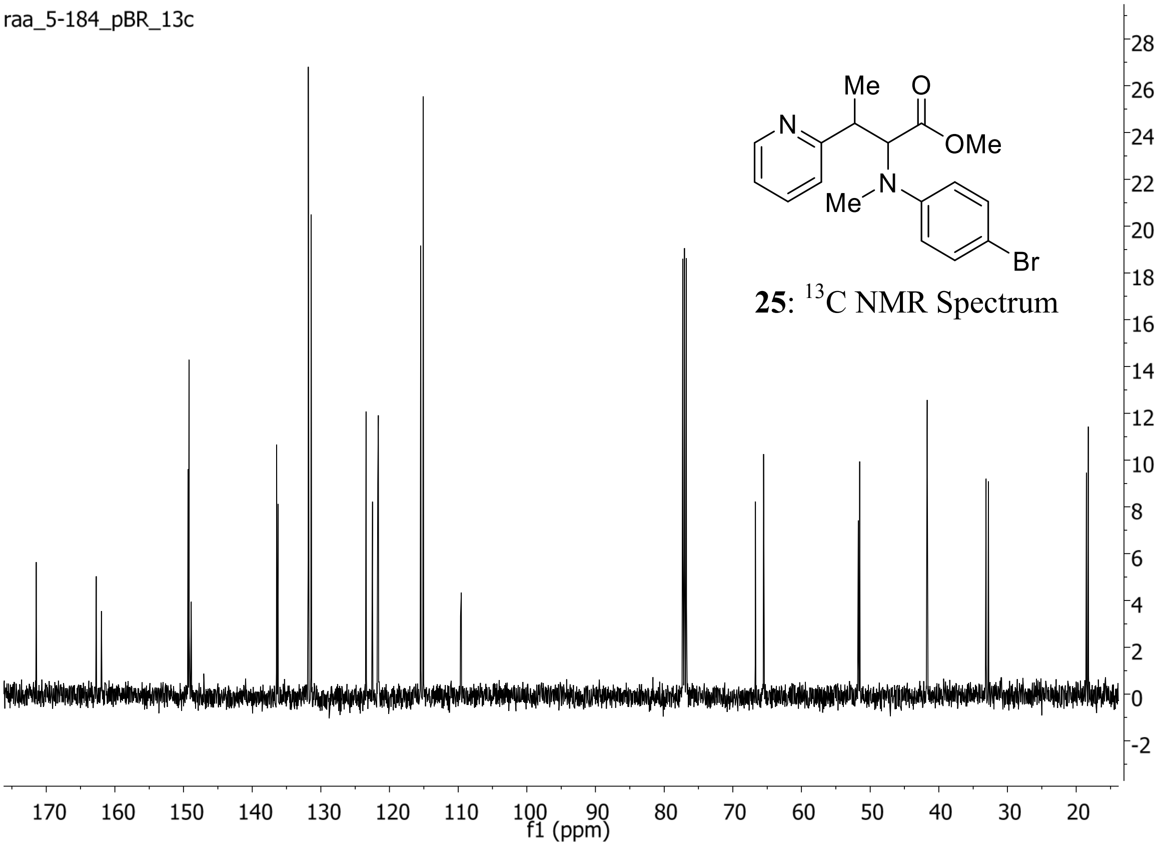
raa_5-184_pBR_13c
Me
O
N
OMe
Me
N
Br
25: 13C NMR Spectrum
f1 (ppm)

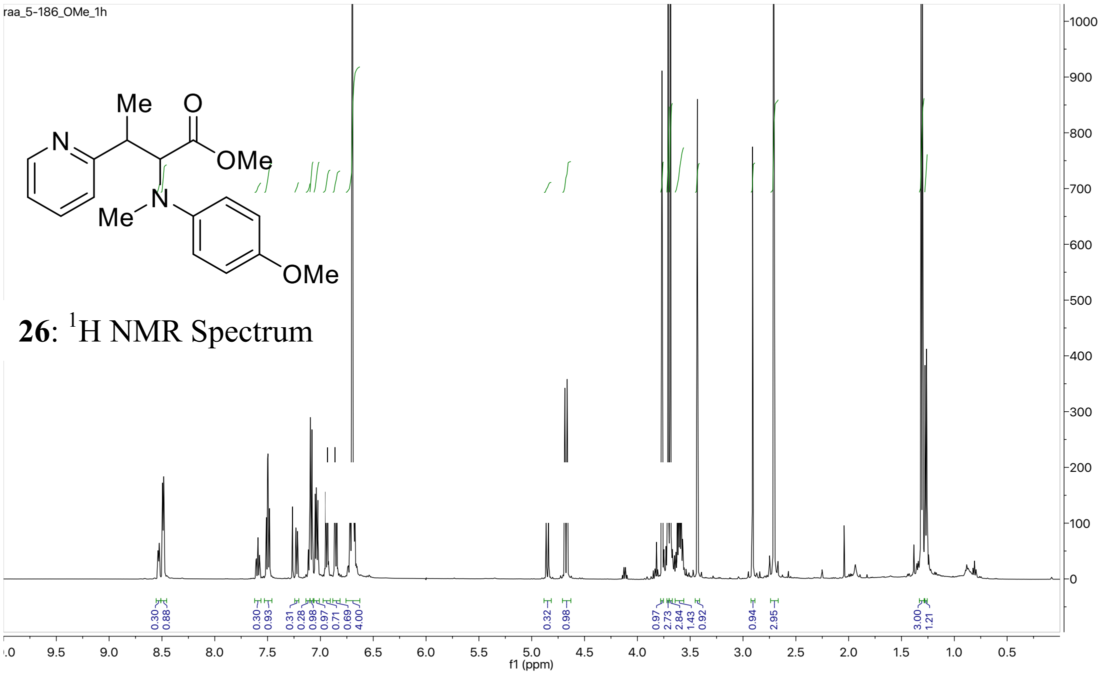
raa_5-186_OMe_1h
Me
O
N
OMe
Me
N
OMe
26: ¹H NMR Spectrum
f1 (ppm)

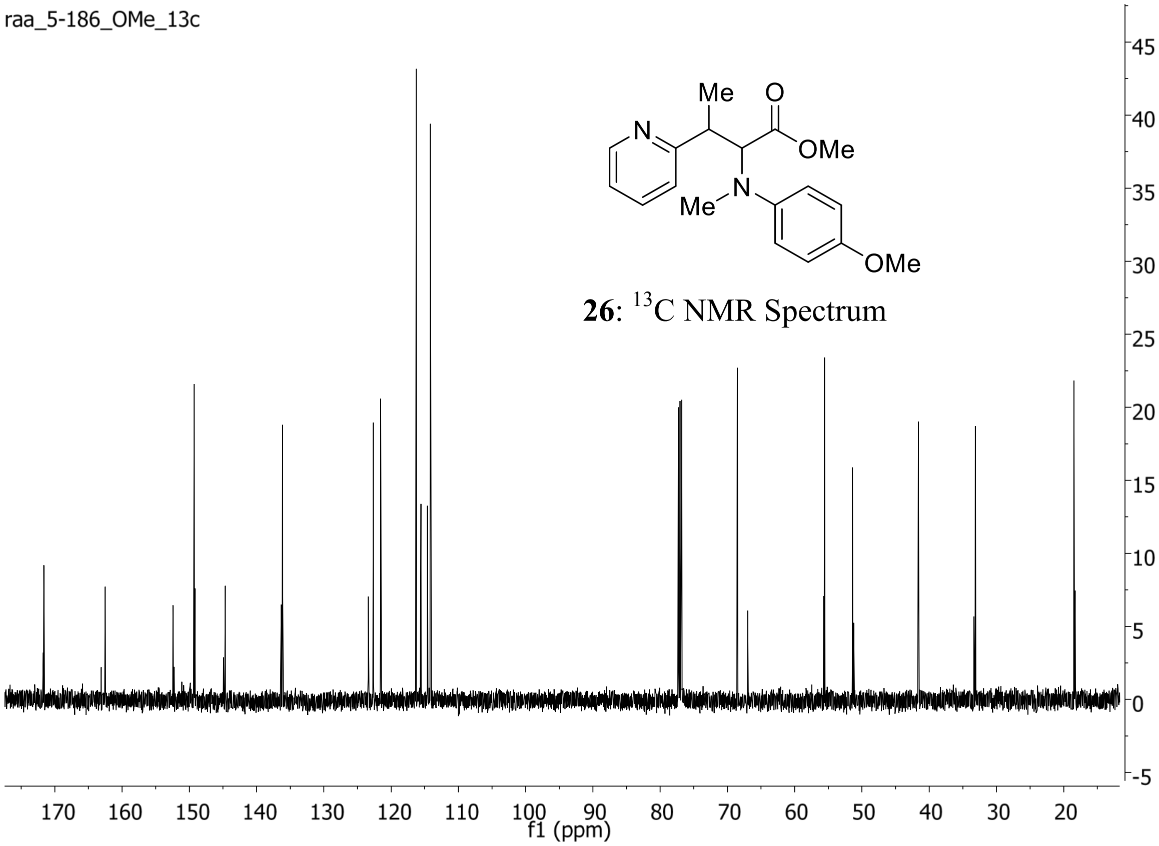
raa_5-186_OMe_13c
Me
O
N
OMe
Me
N
OMe
26: ¹³C NMR Spectrum
f1 (ppm)

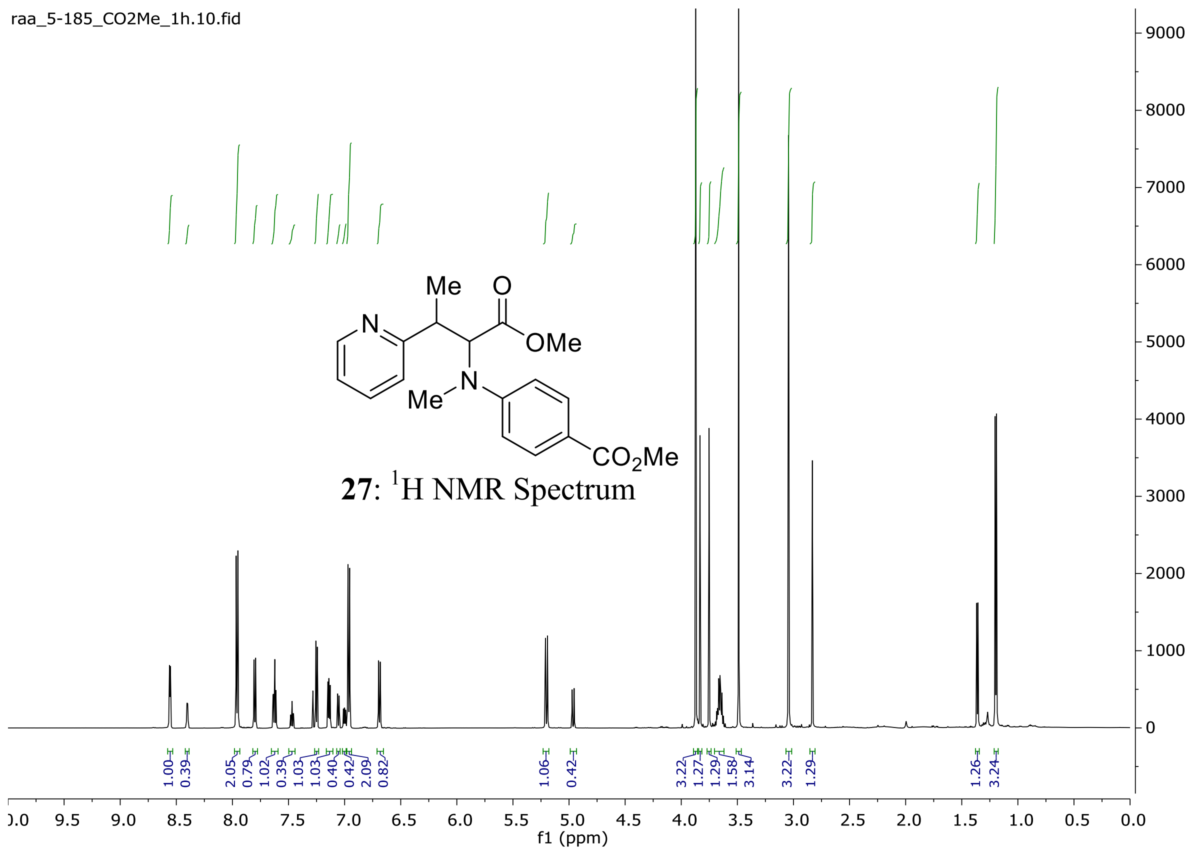
raa_5-185_CO2Me_1h.10.fid
27: 1H NMR Spectrum
f1 (ppm)

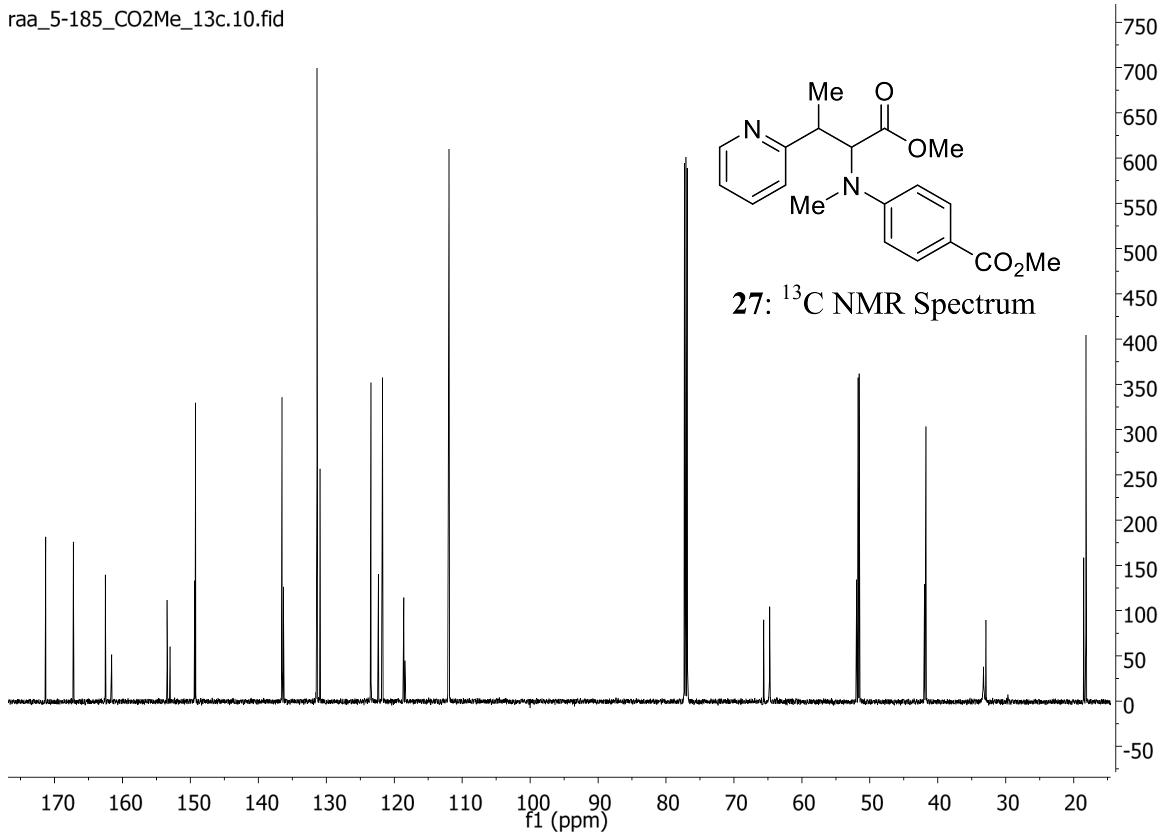
raa_5-185_CO2Me_13c.10.fid
27: 13C NMR Spectrum
f1 (ppm)

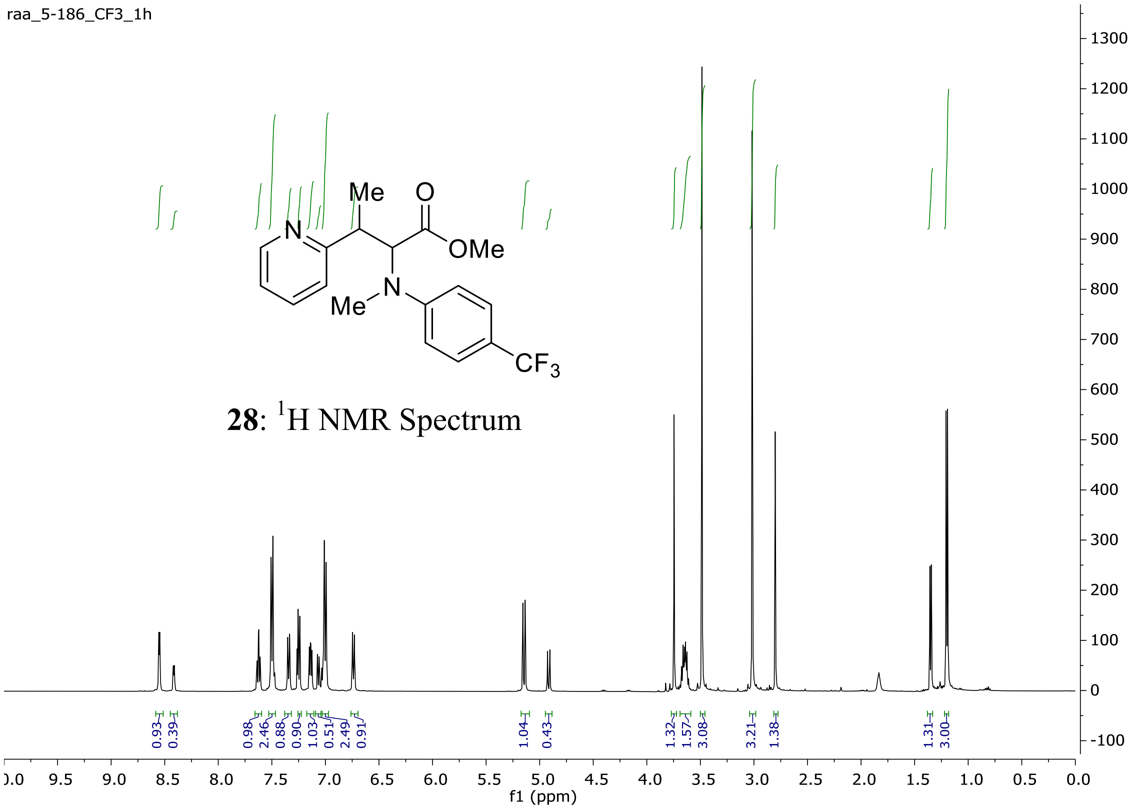
raa_5-186_CF3_1h
Me
O
N
OMe
Me
N
CF3
28: 1H NMR Spectrum
0.93
0.39
0.98
2.46
0.88
0.90
1.03
0.51
2.49
0.91
1.04
0.43
1.32
1.57
3.08
3.21
1.38
1.31
3.00
f1 (ppm)

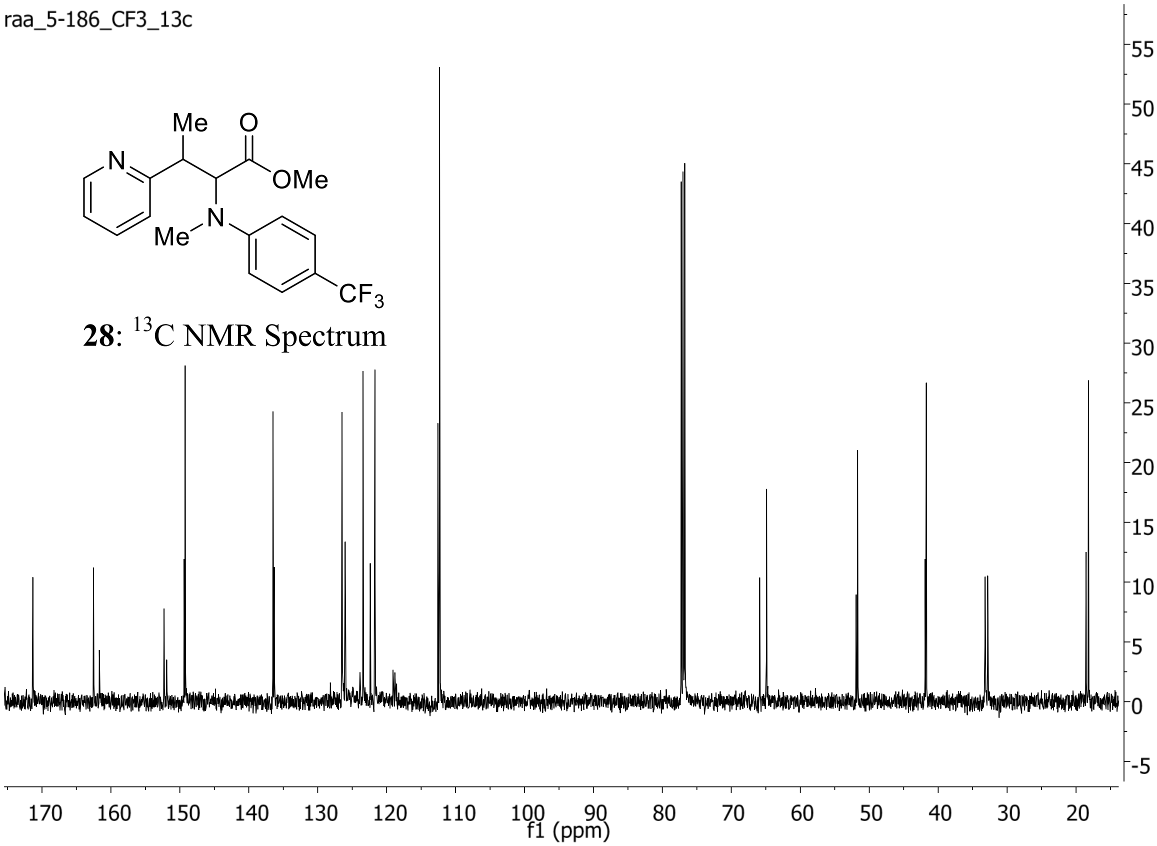
raa_5-186_CF3_13c
Me
O
N
OMe
Me
N
CF3
28: 13C NMR Spectrum
f1 (ppm)

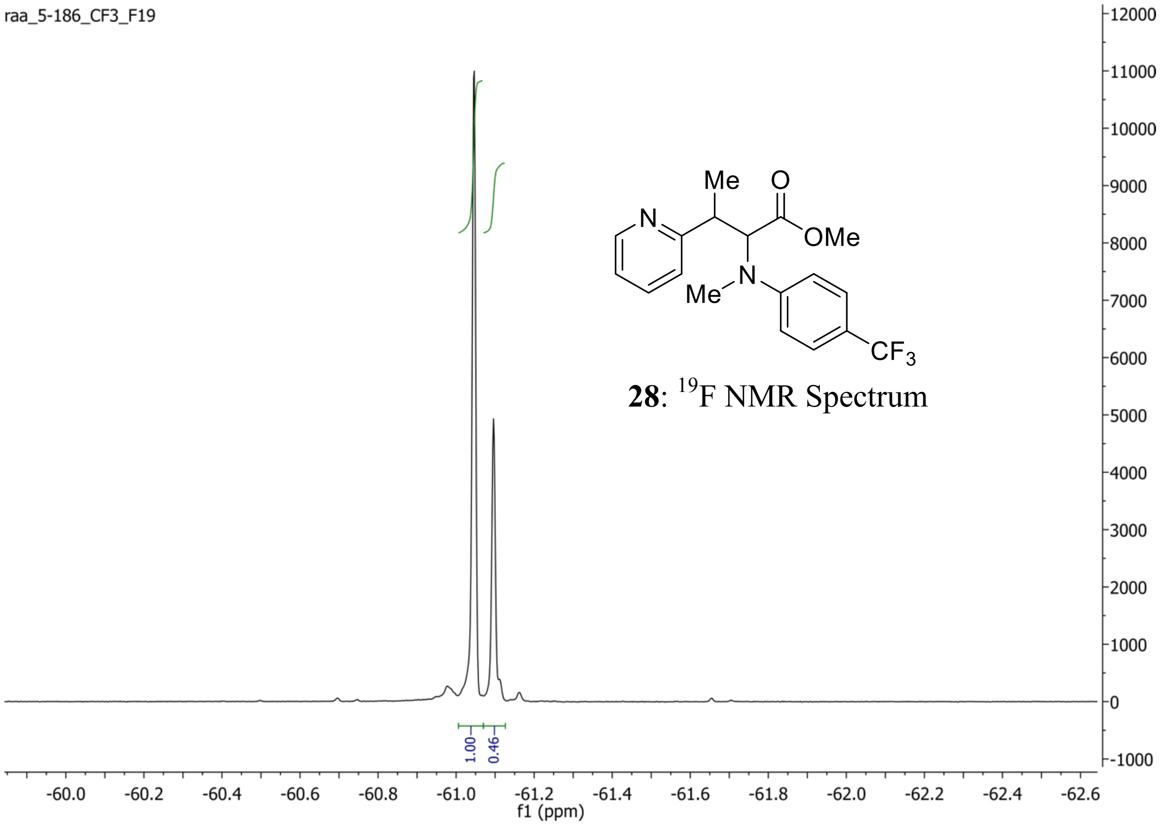

28: ^{19}F NMR Spectrum

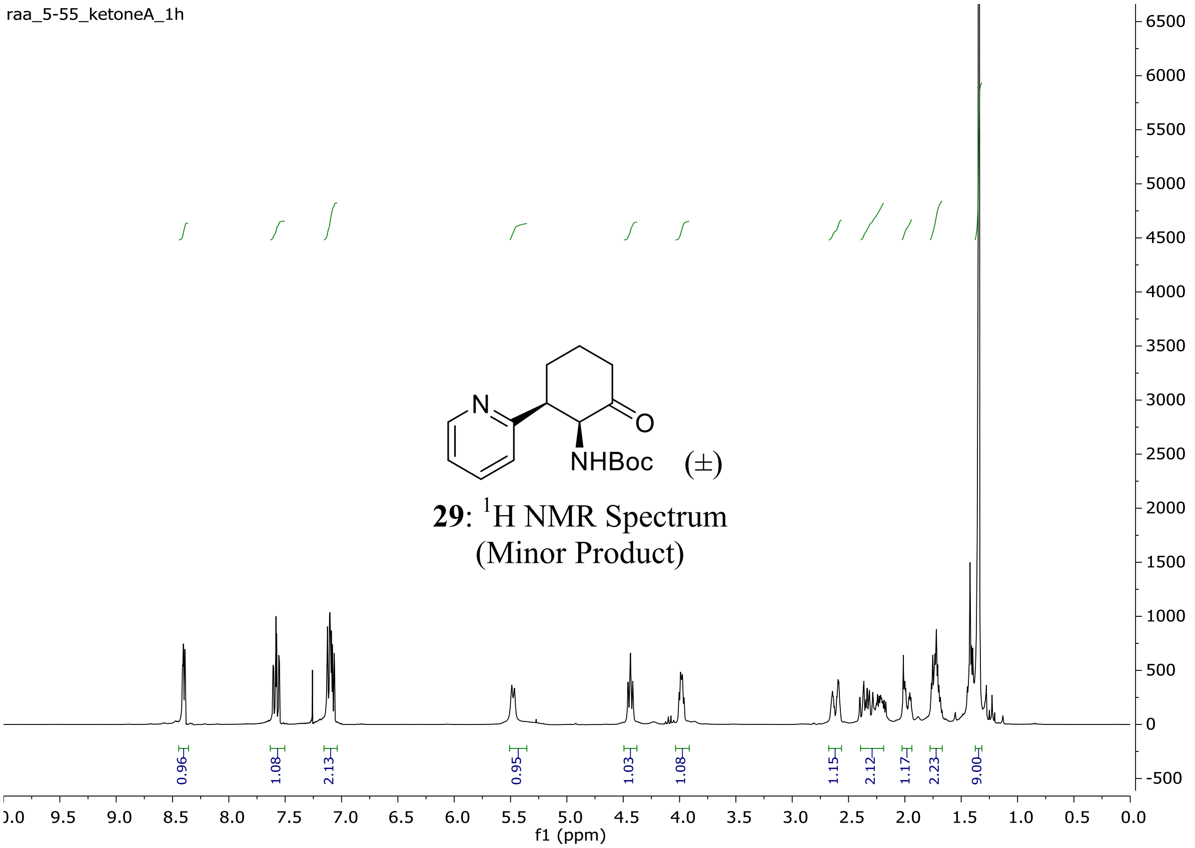

29: ^{1}H NMR Spectrum
(Minor Product)

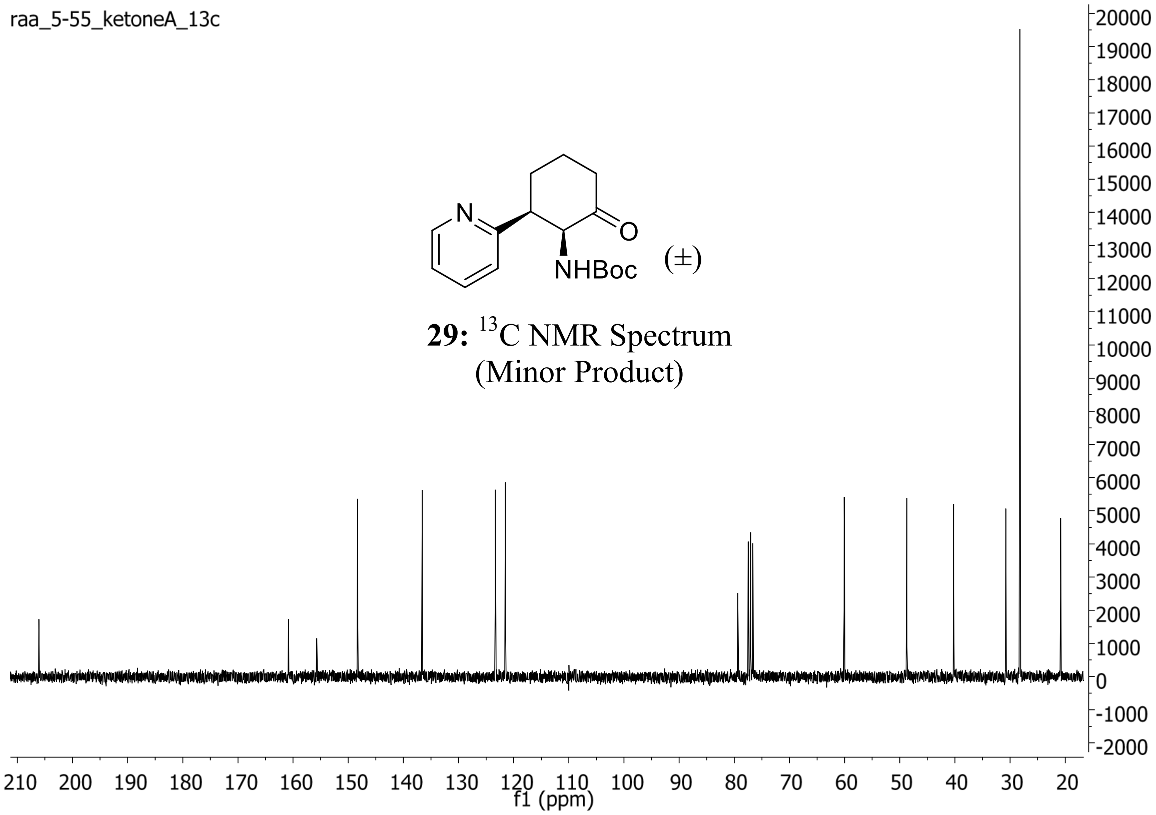

29: ^{13}C NMR Spectrum
(Minor Product)

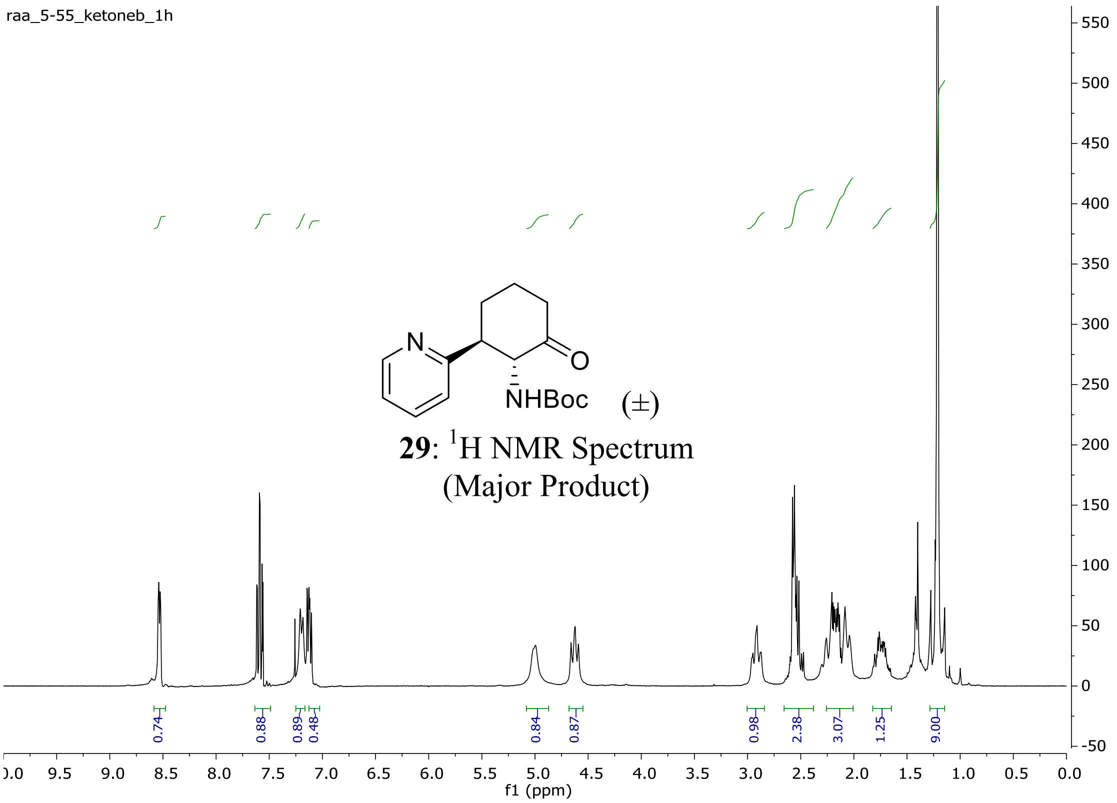
raa_5-55_ketoneb_1h
N
O
NHBoc
(±)
29: 1H NMR Spectrum
(Major Product)
0.74
0.88
0.89
0.48
0.84
0.87
0.98
2.38
3.07
1.25
9.00
f1 (ppm)

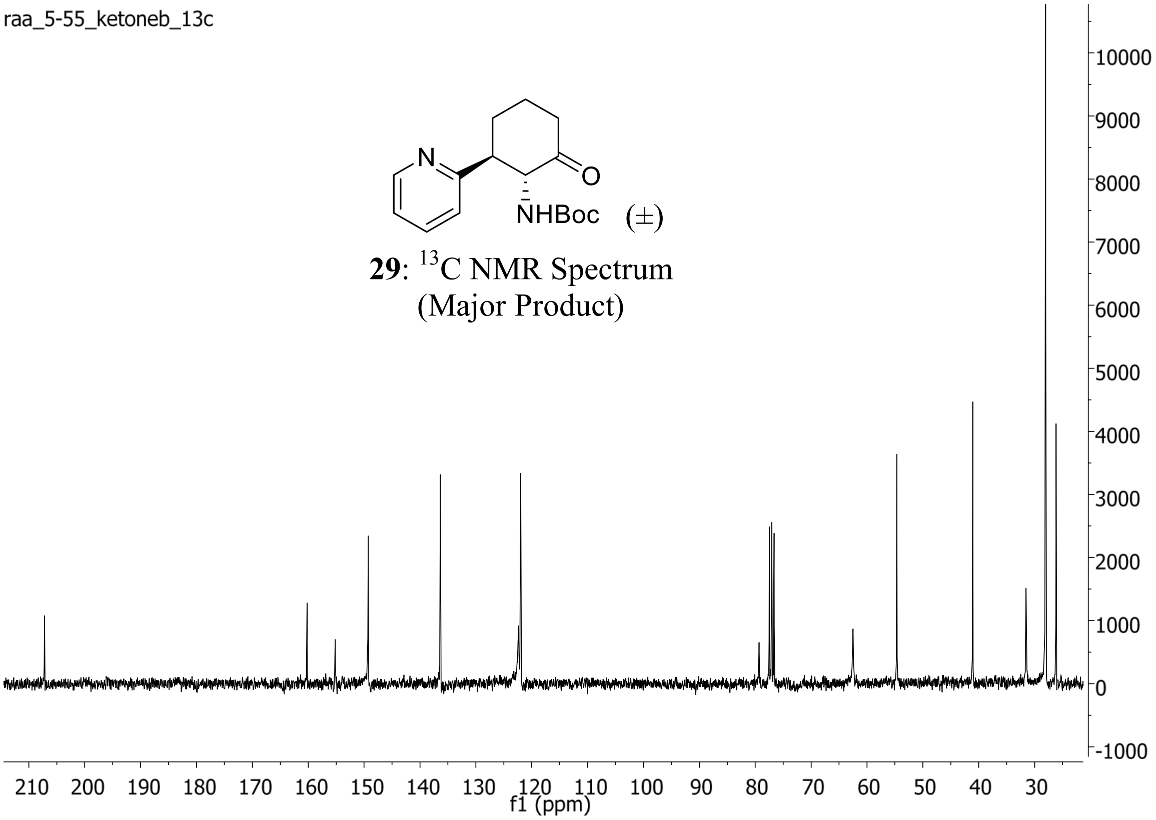
raa_5-55_ketoneb_13c
N
O
NHBoc
(±)
29: 13C NMR Spectrum
(Major Product)
f1 (ppm)

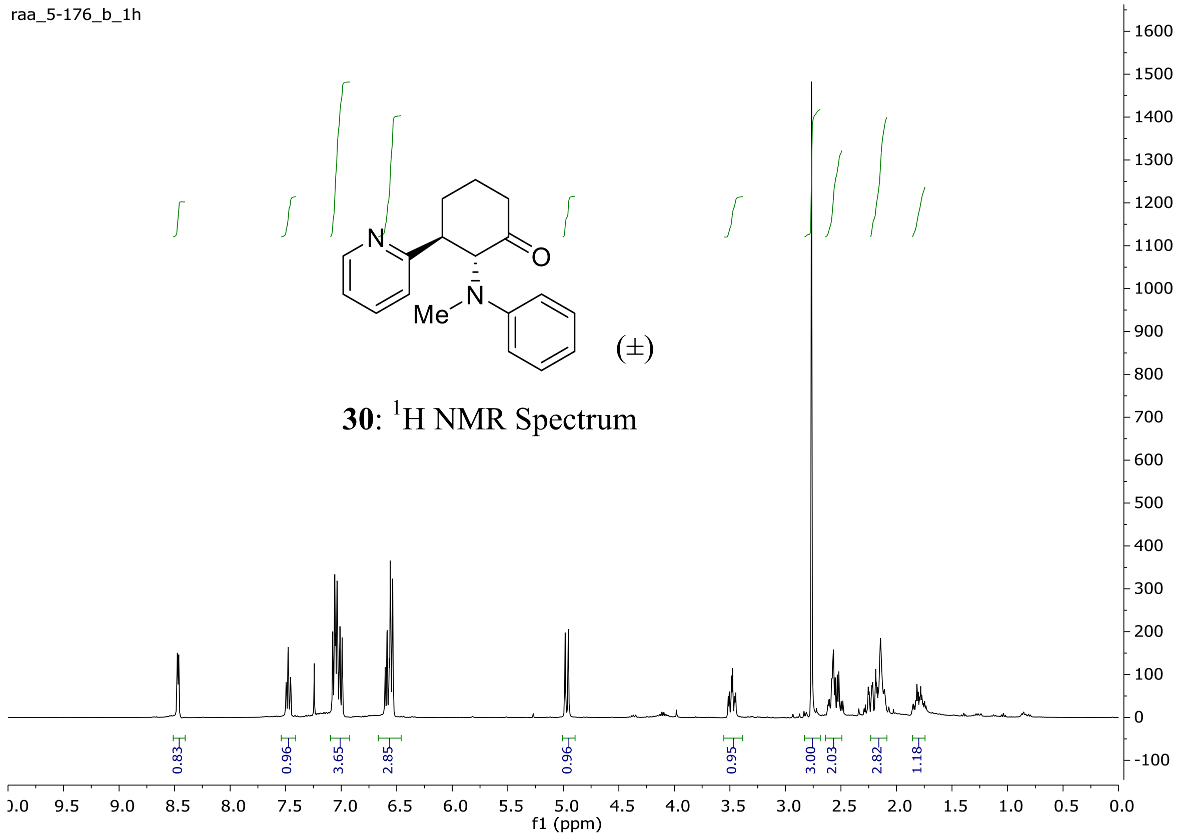
raa_5-176_b_1h
N
O
N
Me
(±)
30: 1H NMR Spectrum
0.83
0.96
3.65
2.85
0.96
0.95
3.00
2.03
2.82
1.18
f1 (ppm)

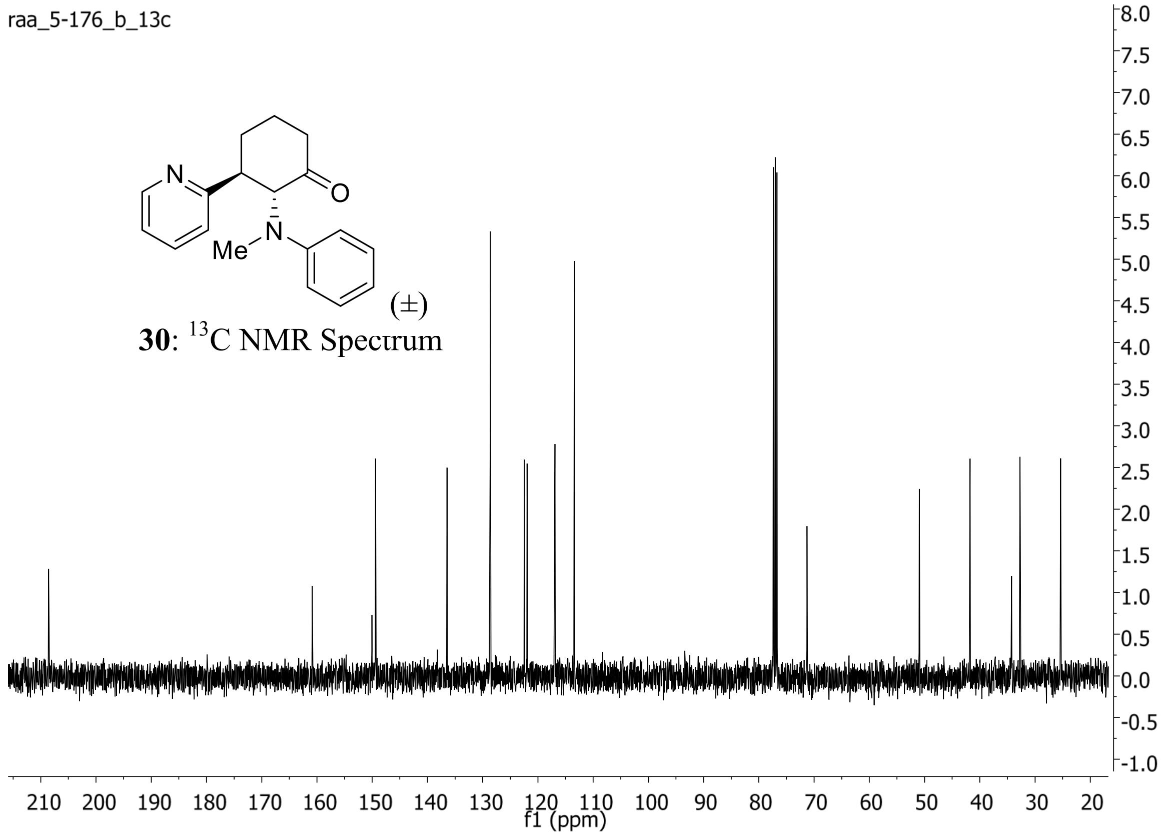
raa_5-176_b_13c
N
O
N
Me
(±)
30: 13C NMR Spectrum
f1 (ppm)

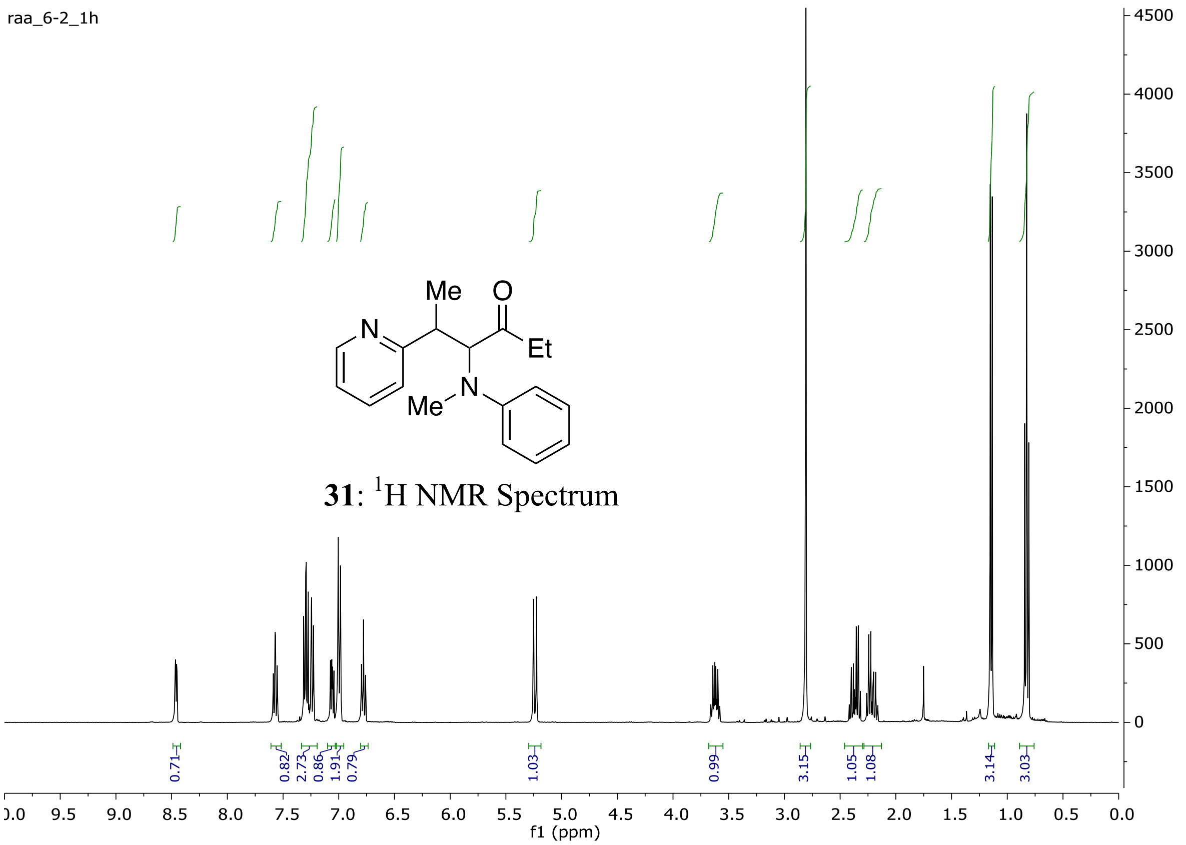
raa_6-2_1h
Me
O
N
Et
Me
N
31: ¹H NMR Spectrum
0.71
0.82
2.73
0.86
1.91
0.79
1.03
0.99
3.15
1.05
1.08
3.14
3.03
f1 (ppm)

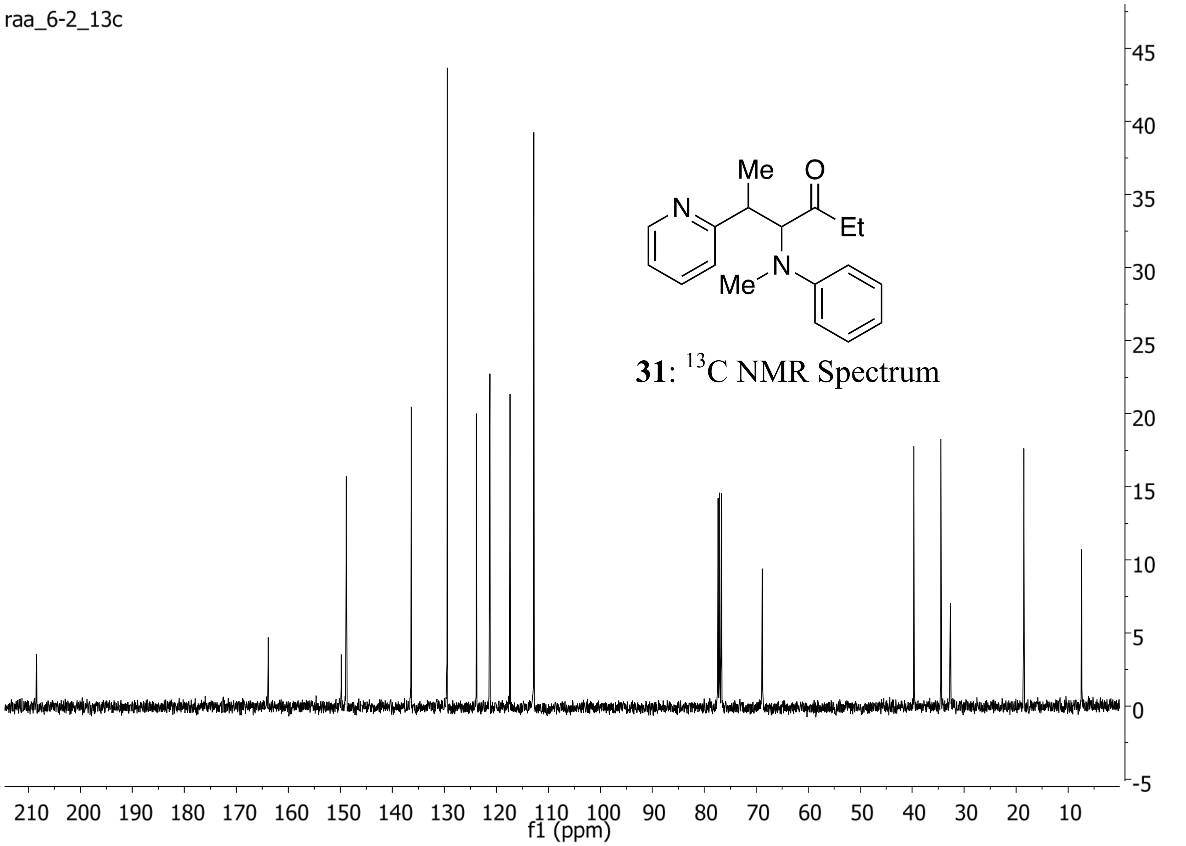
raa_6-2_13c
Me
O
N
Et
Me
N
31: ¹³C NMR Spectrum
f1 (ppm)

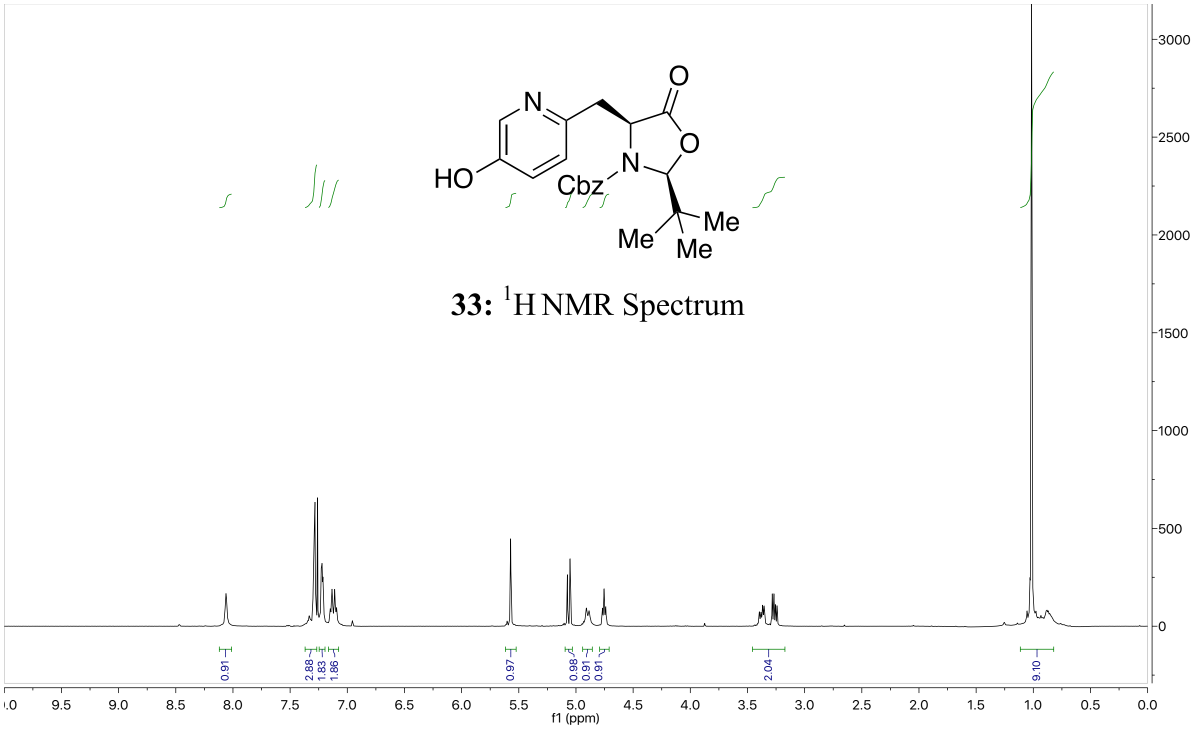

N
O
O
N
HO
Cbz
Me
Me
Me
33: ¹H NMR Spectrum
f1 (ppm)

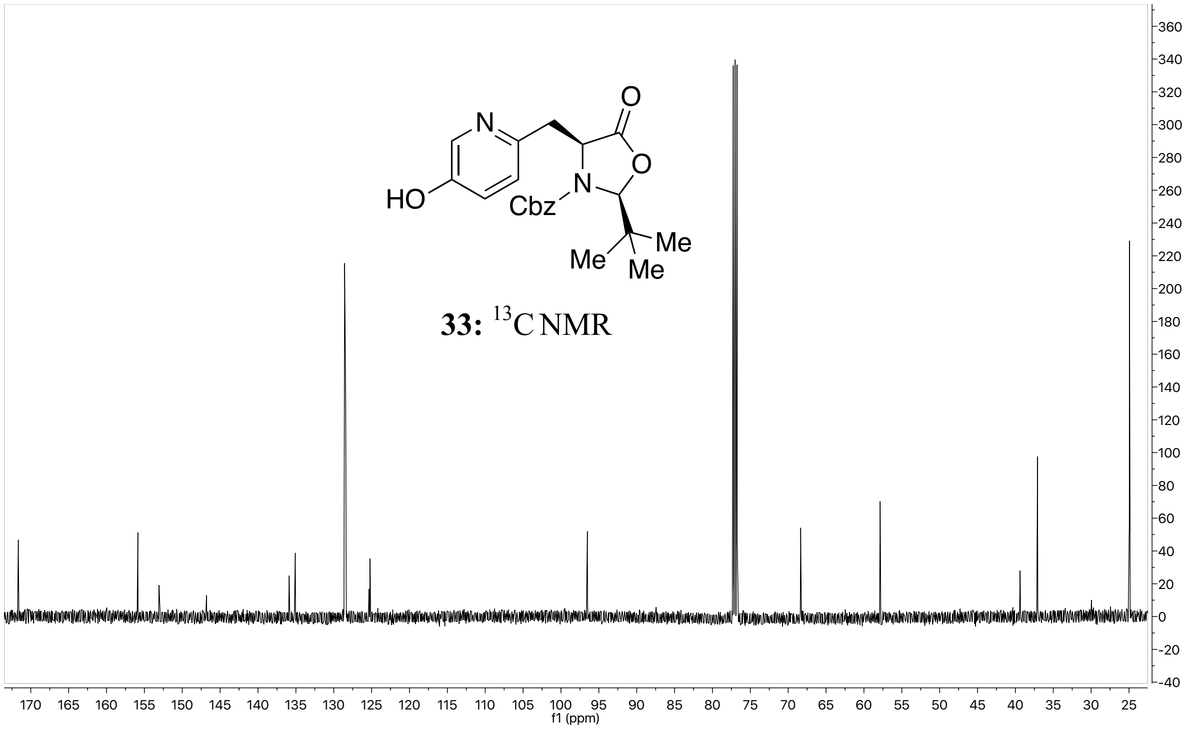

N
O
O
N
HO
Cbz
Me
Me
Me
33: ¹³C NMR
f1 (ppm)

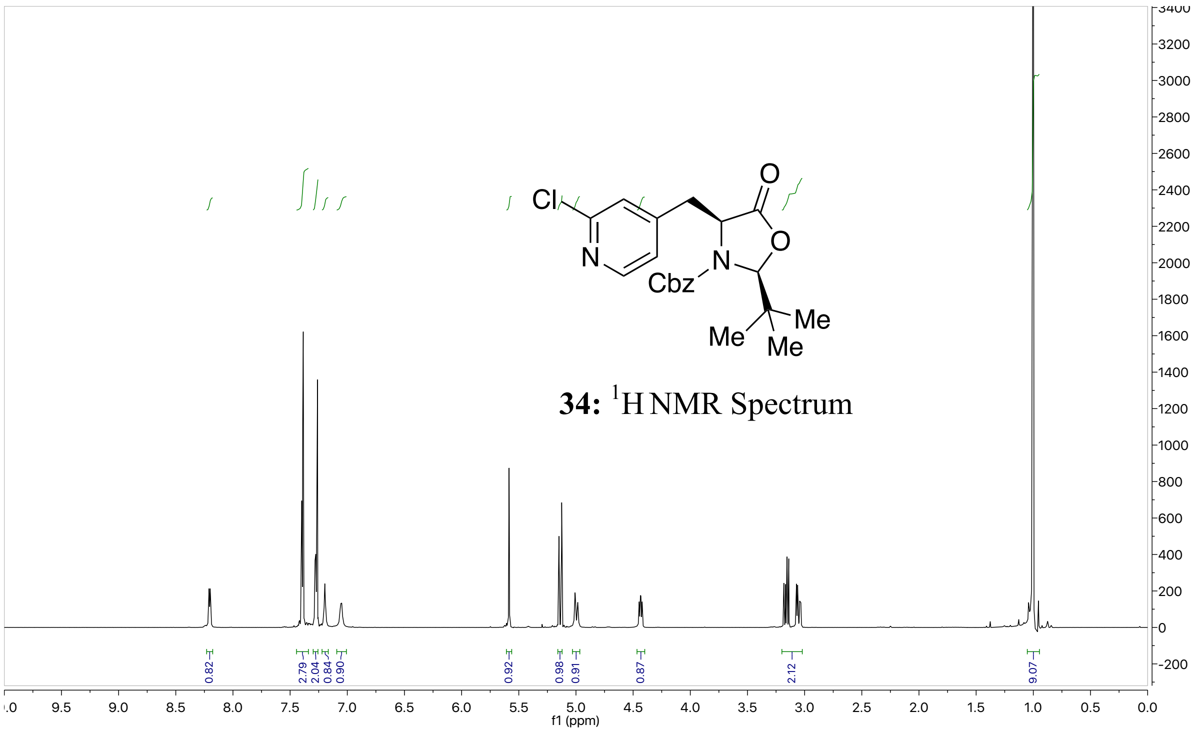
Cl
N
O
O
N
Cbz
Me
Me
Me
34: ^{1}H NMR Spectrum
0.82
2.79
2.04
0.84
0.90
0.92
0.98
0.91
0.87
2.12
9.07
f1 (ppm)

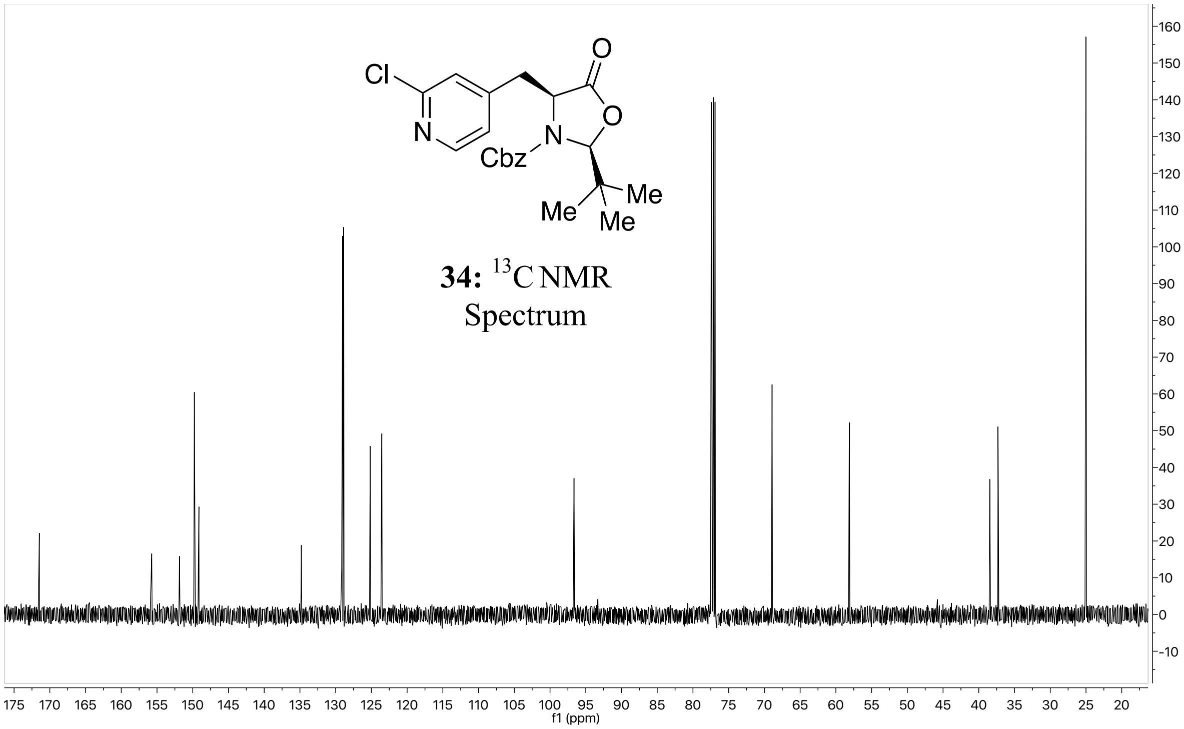
Cl
N
O
O
N
Cbz
Me
Me
Me
34: ^{13}C NMR Spectrum
f1 (ppm)

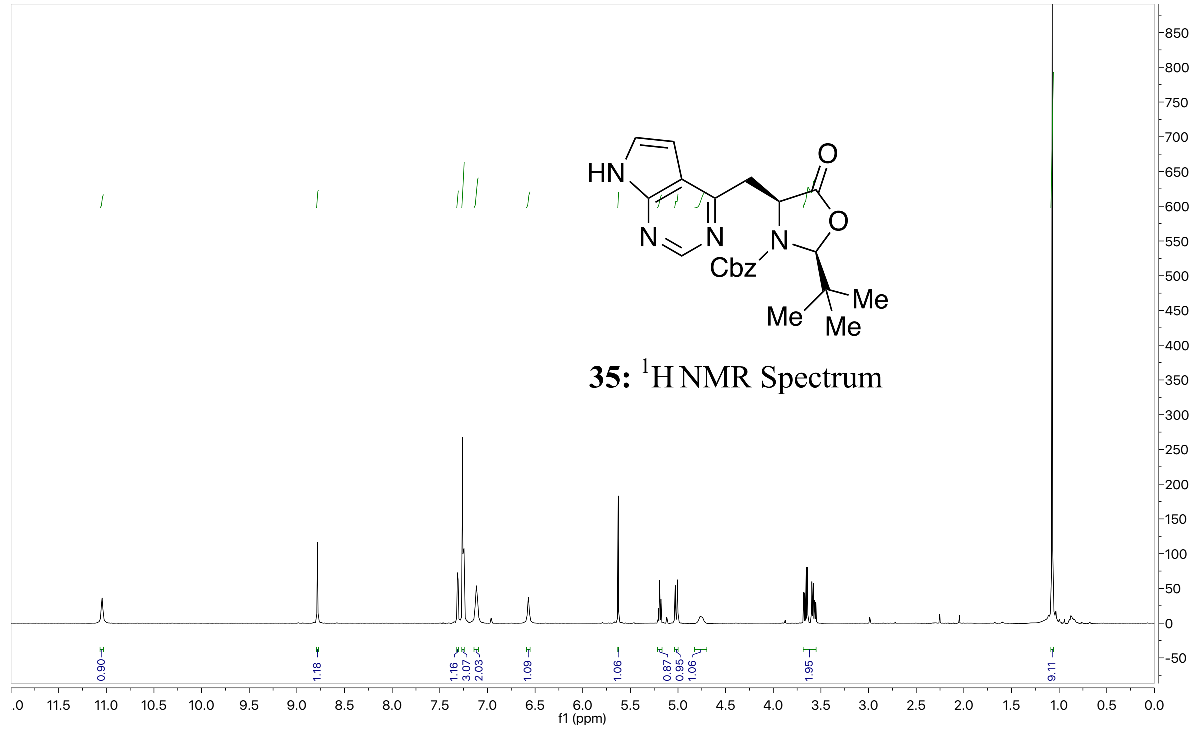
HN
N
N
O
O
N
Cbz
Me
Me
Me
35: 1H NMR Spectrum
0.90
1.18
1.16
3.07
2.03
1.09
1.06
0.87
0.95
1.06
1.95
9.11
f1 (ppm)

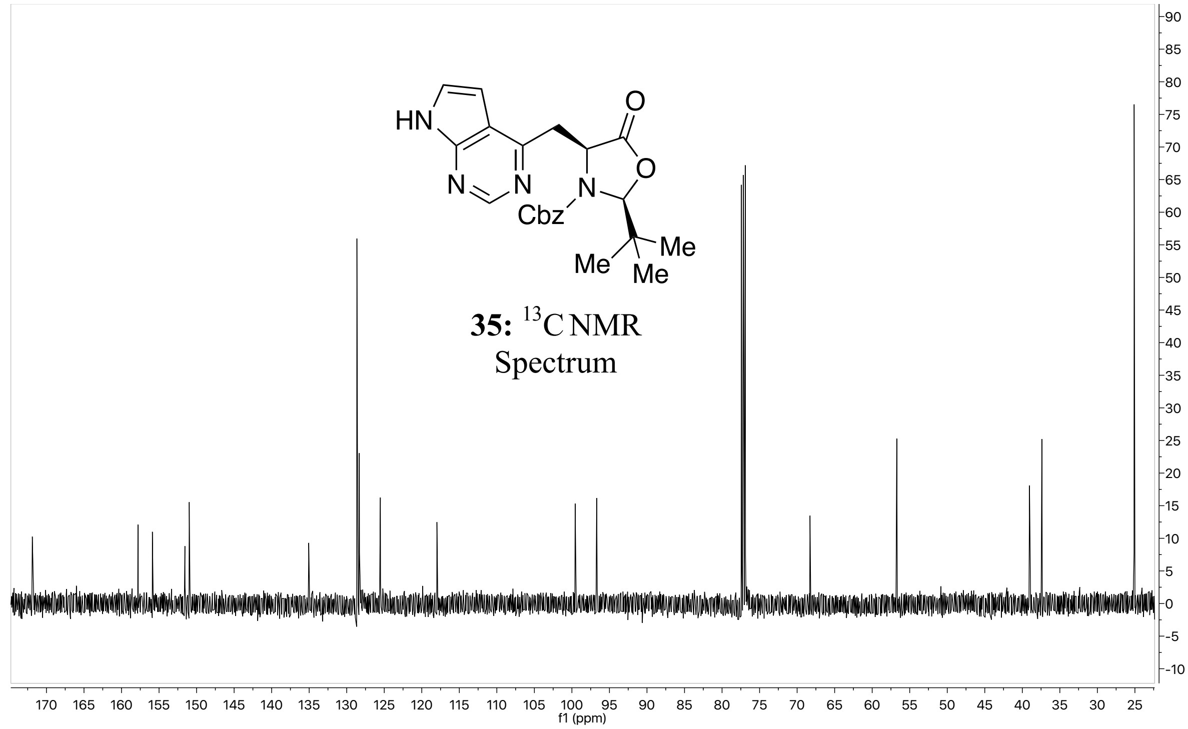
HN
N
N
O
O
N
Cbz
Me
Me
Me
35: 13C NMR Spectrum
f1 (ppm)

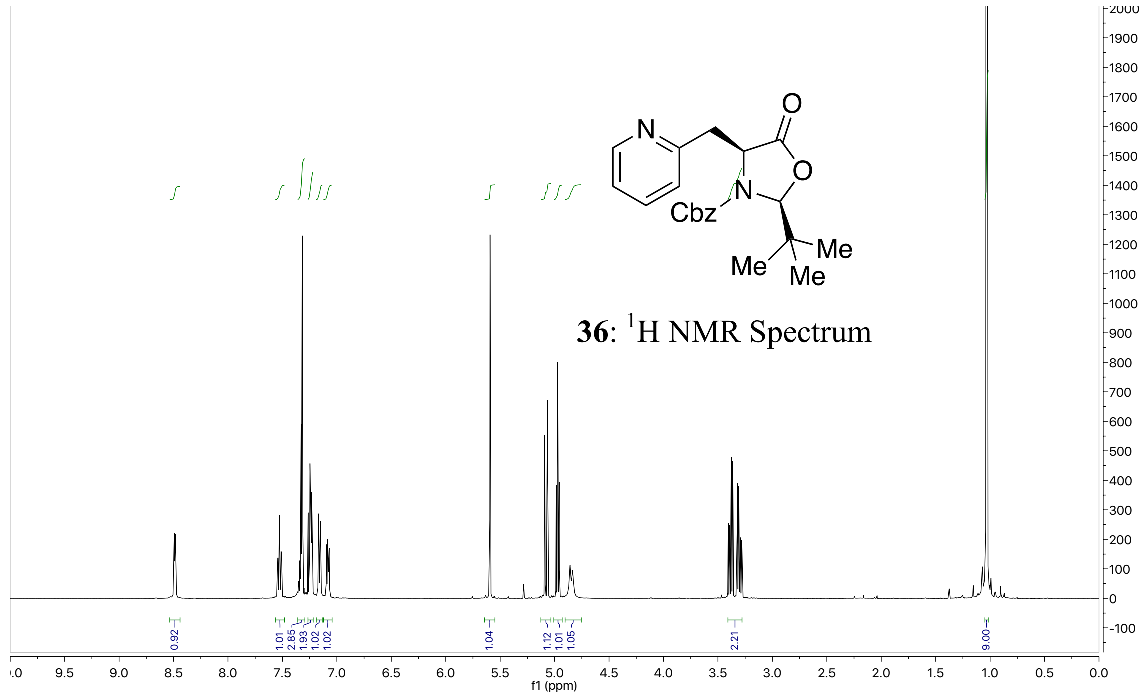

N
O
O
Cbz
N
Me
Me
Me
36: ^{1}H NMR Spectrum
0.92
1.01
2.85
1.93
1.02
1.02
1.04
1.12
1.01
1.05
2.21
9.00
f1 (ppm)

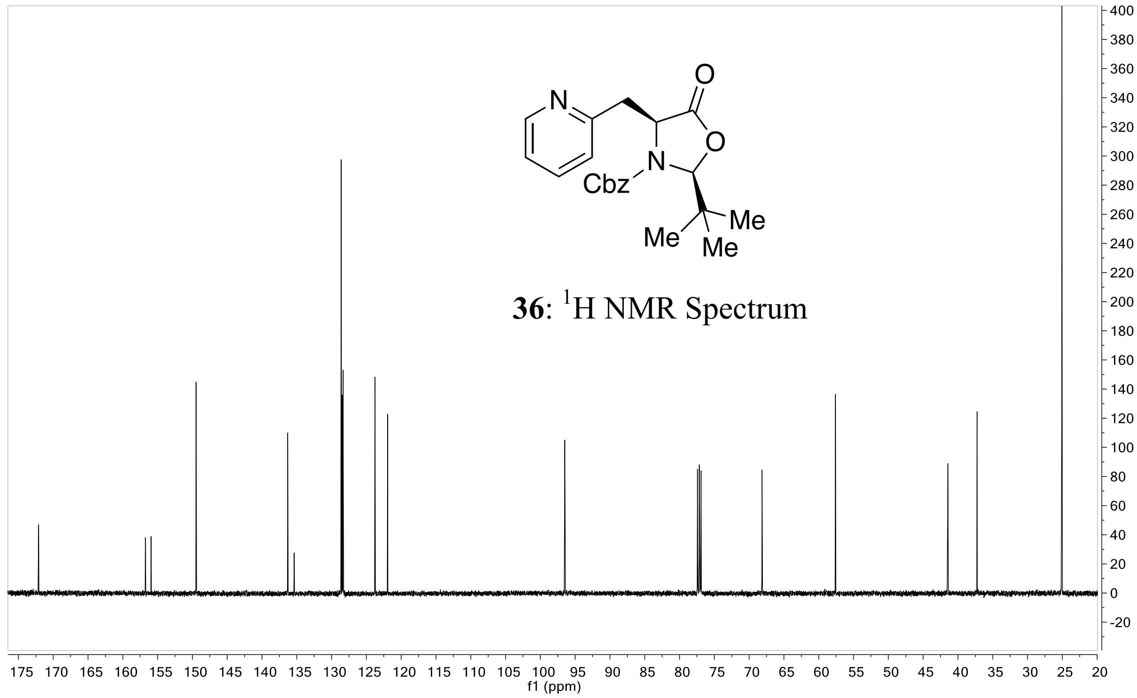

N
O
O
Cbz
N
Me
Me
Me
36: ^{1}H NMR Spectrum
f1 (ppm)

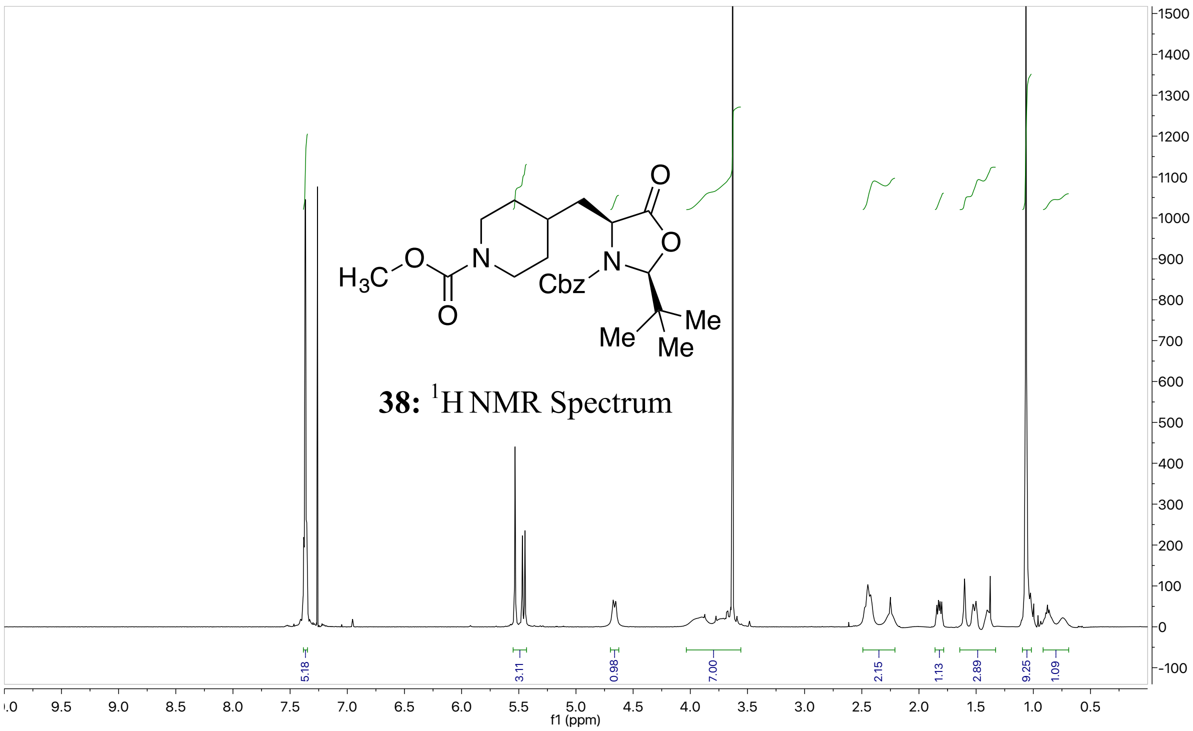
38: 1H NMR Spectrum
H3C O N O Cbz N O O Me Me Me
f1 (ppm)

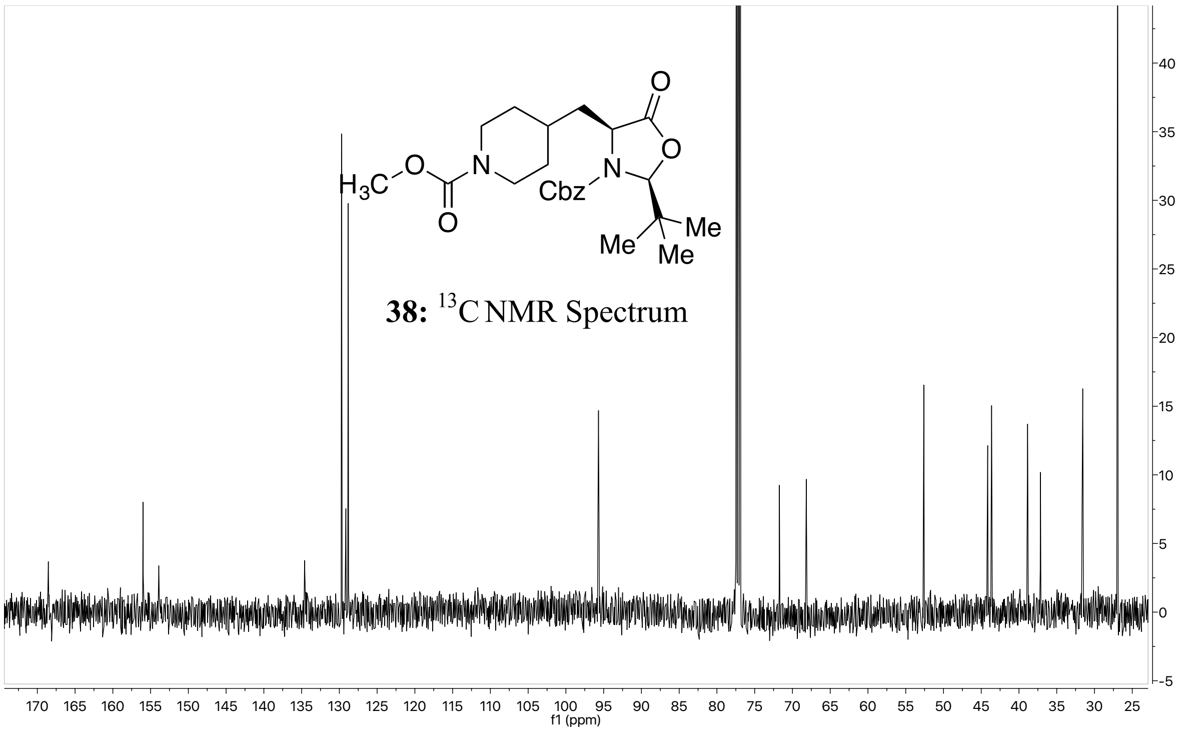
38: 13C NMR Spectrum
H3C O N O Cbz N O O Me Me Me
f1 (ppm)

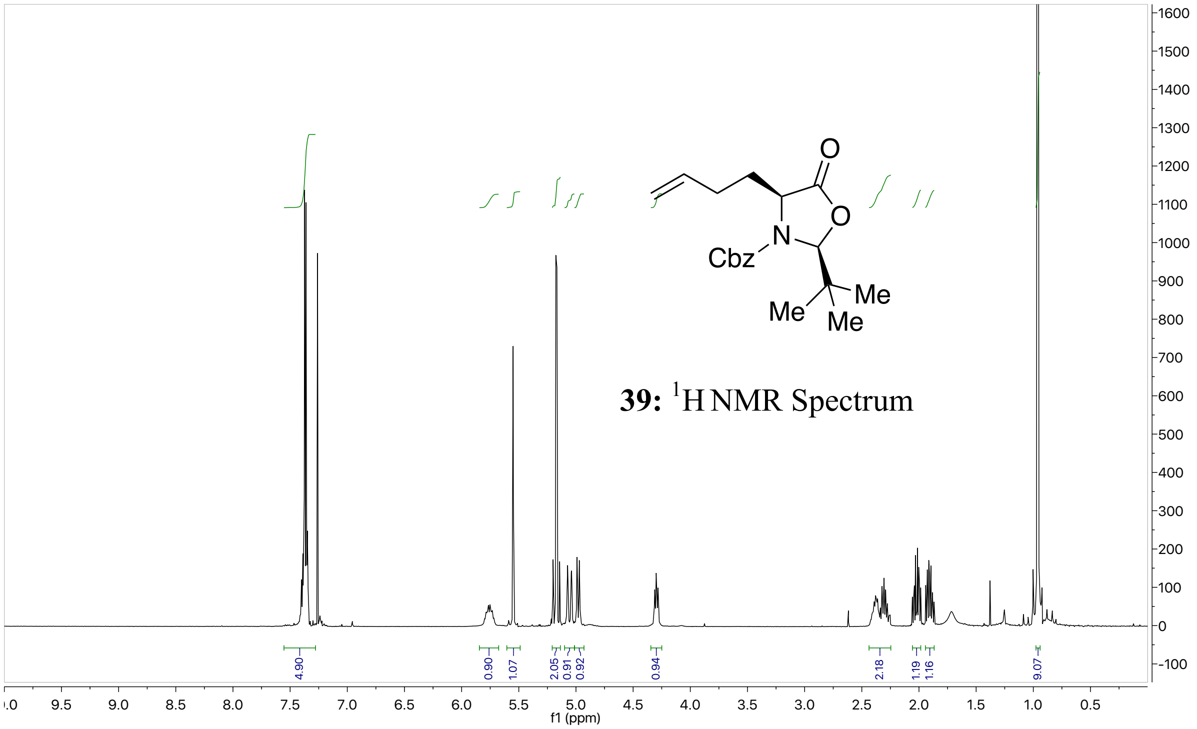
O
O
N
Cbz
Me
Me
Me
39: ¹H NMR Spectrum
4.90
0.90
1.07
2.05
0.91
0.92
0.94
2.18
1.19
1.16
9.07
f1 (ppm)

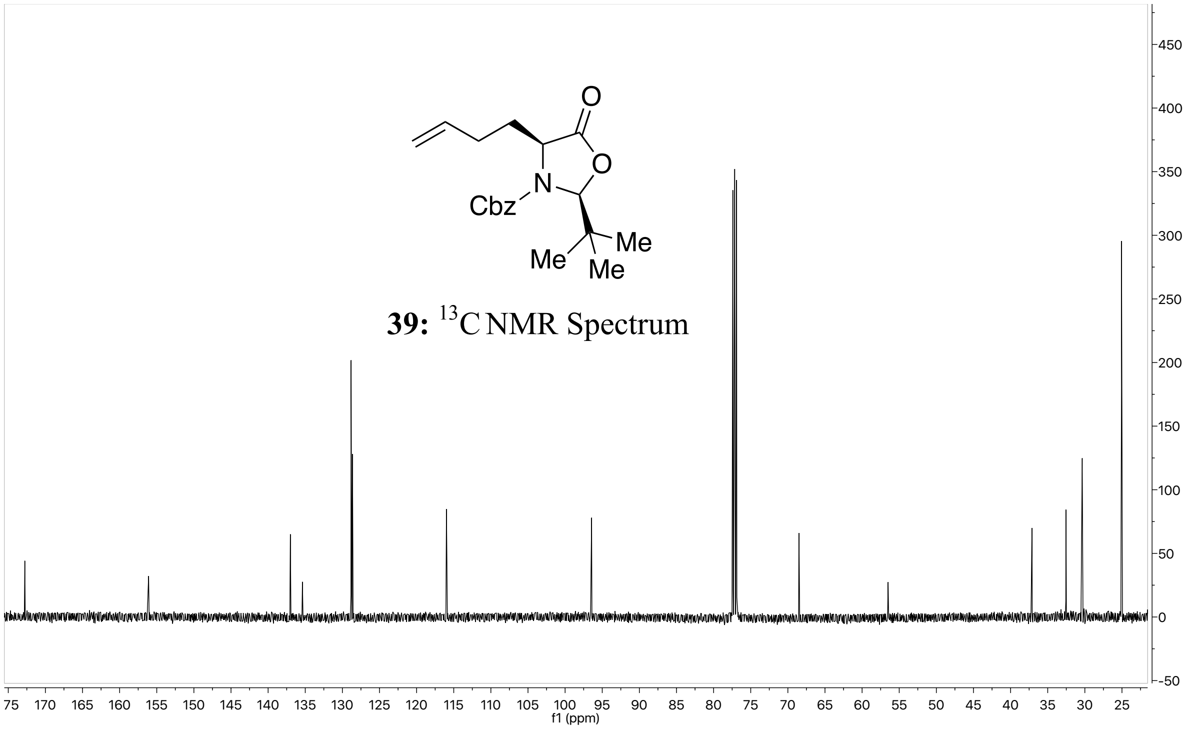
O
O
N
Cbz
Me
Me
Me
39: ¹³C NMR Spectrum
f1 (ppm)

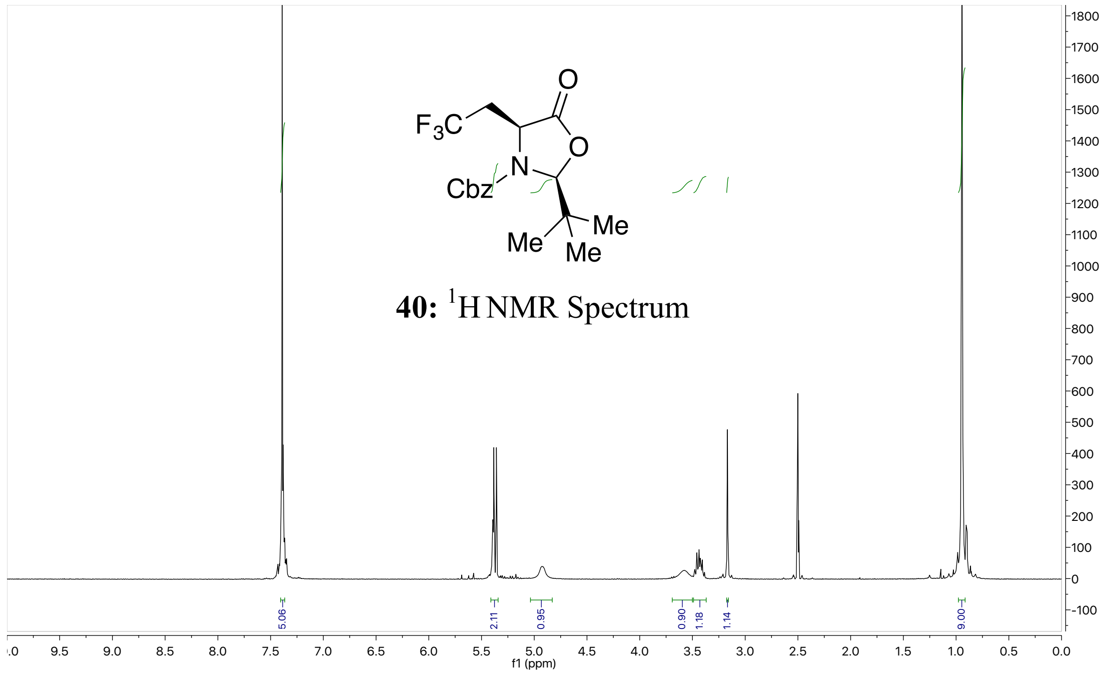

F3C
O
O
N
Cbz
Me
Me
Me
40: 1H NMR Spectrum
f1 (ppm)

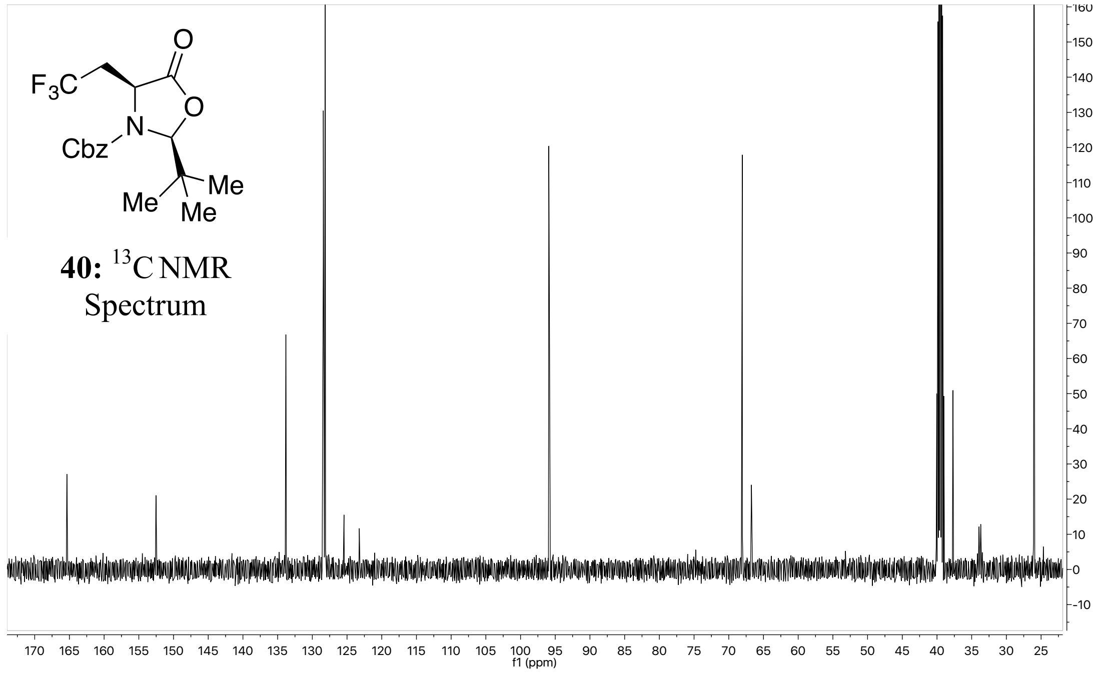

F3C
O
O
N
Cbz
Me
Me
Me
40: 13C NMR Spectrum
f1 (ppm)

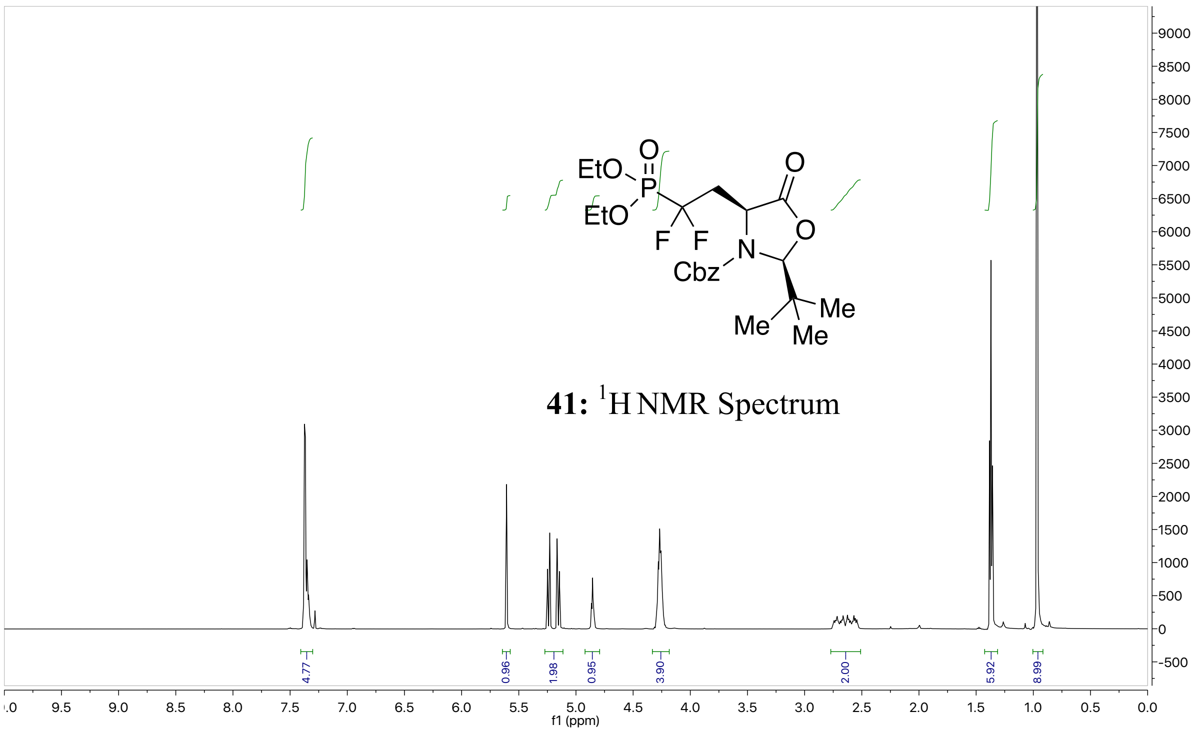
EtO
EtO
P
O
O
O
F F
N
Cbz
Me
Me
Me
41: ¹H NMR Spectrum
4.77
0.96
1.98
0.95
3.90
2.00
5.92
8.99
f1 (ppm)

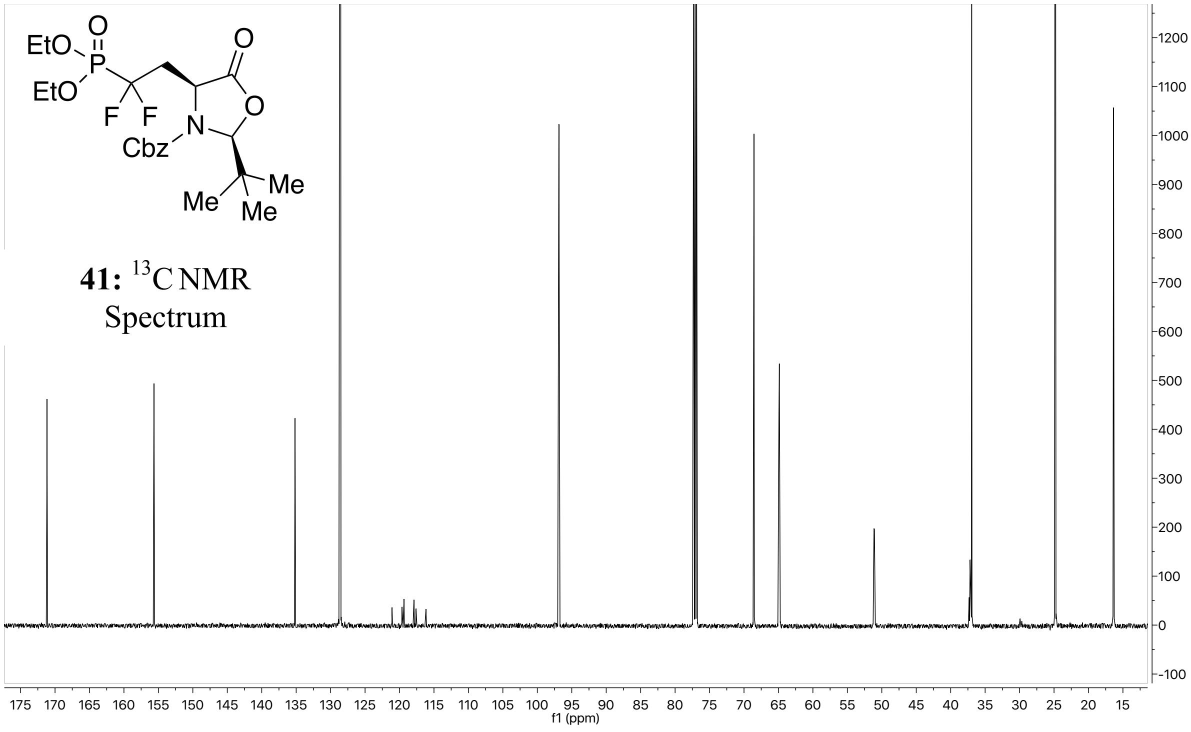
EtO
EtO
P
O
O
O
F F
N
Cbz
Me
Me
Me
41: ¹³C NMR Spectrum
f1 (ppm)

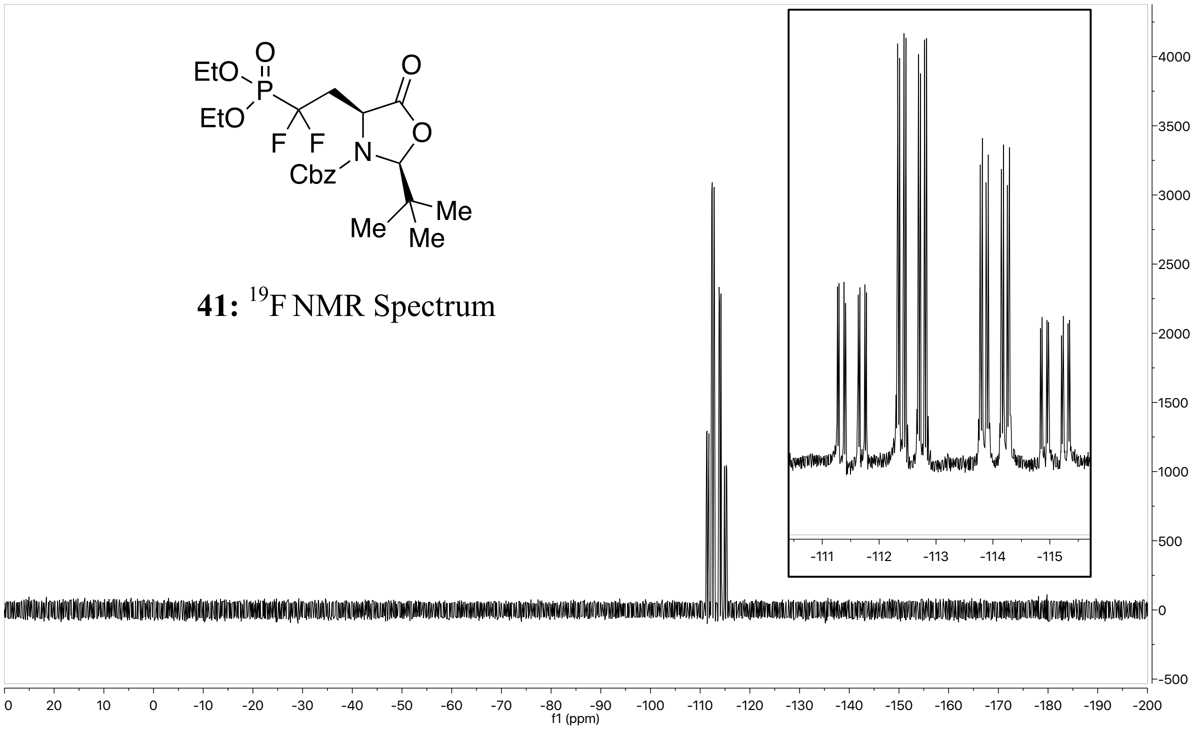
41: ^{19}F NMR Spectrum
EtO
EtO
P
O
F F
N
Cbz
O
O
Me
Me
Me
f1 (ppm)

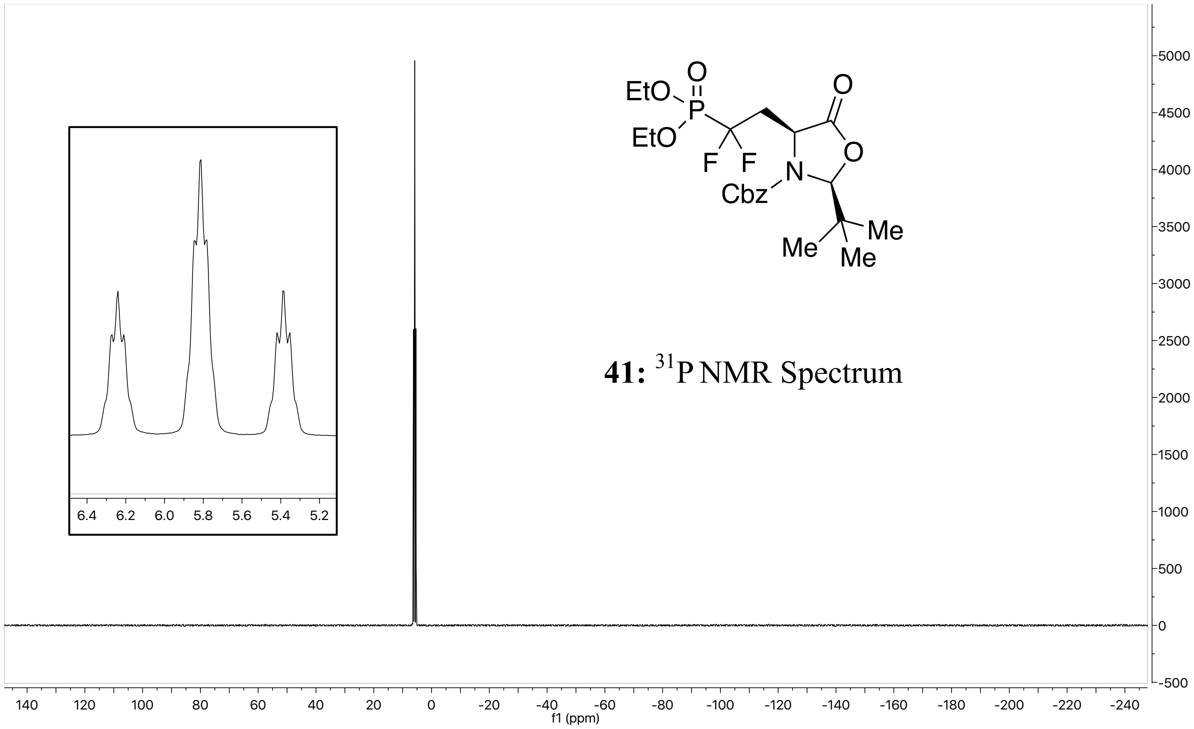
41: ^{31}P NMR Spectrum
EtO
EtO
P
O
F F
N
Cbz
O
O
Me
Me
Me
f1 (ppm)

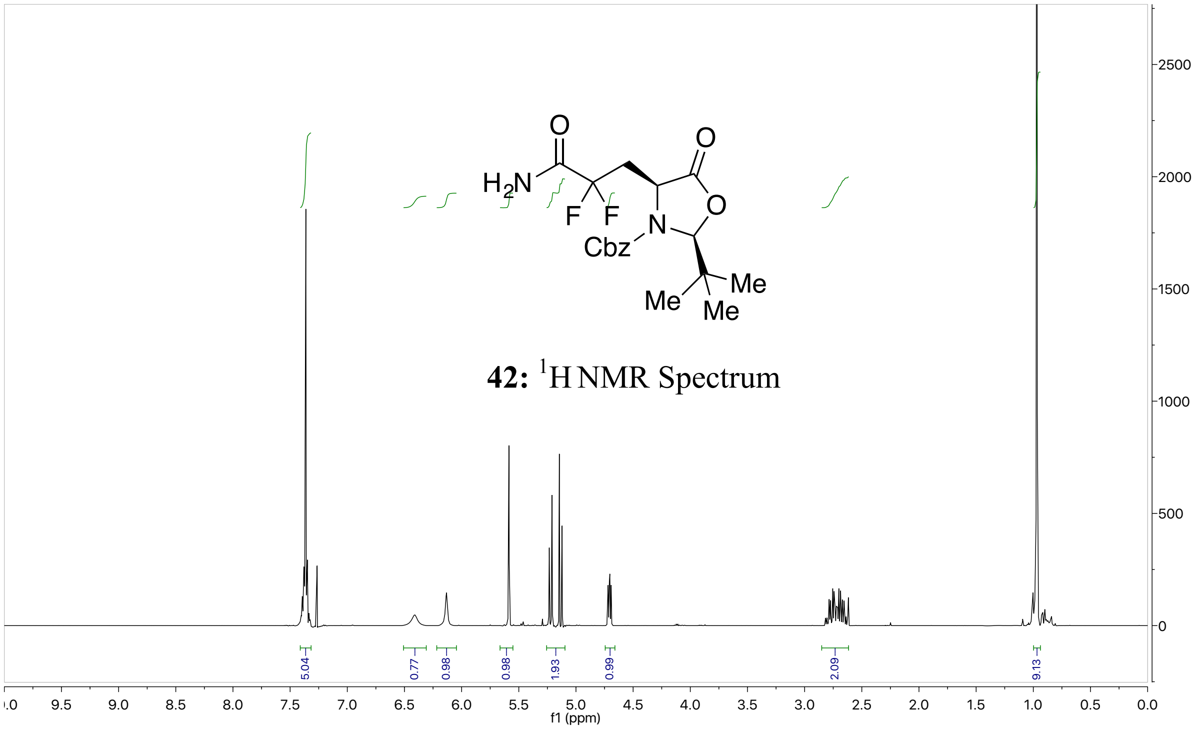

42: ¹H NMR Spectrum
H2N
O
F F
N
Cbz
O
O
Me
Me
Me
5.04
0.77
0.98
0.98
1.93
0.99
2.09
9.13
f1 (ppm)

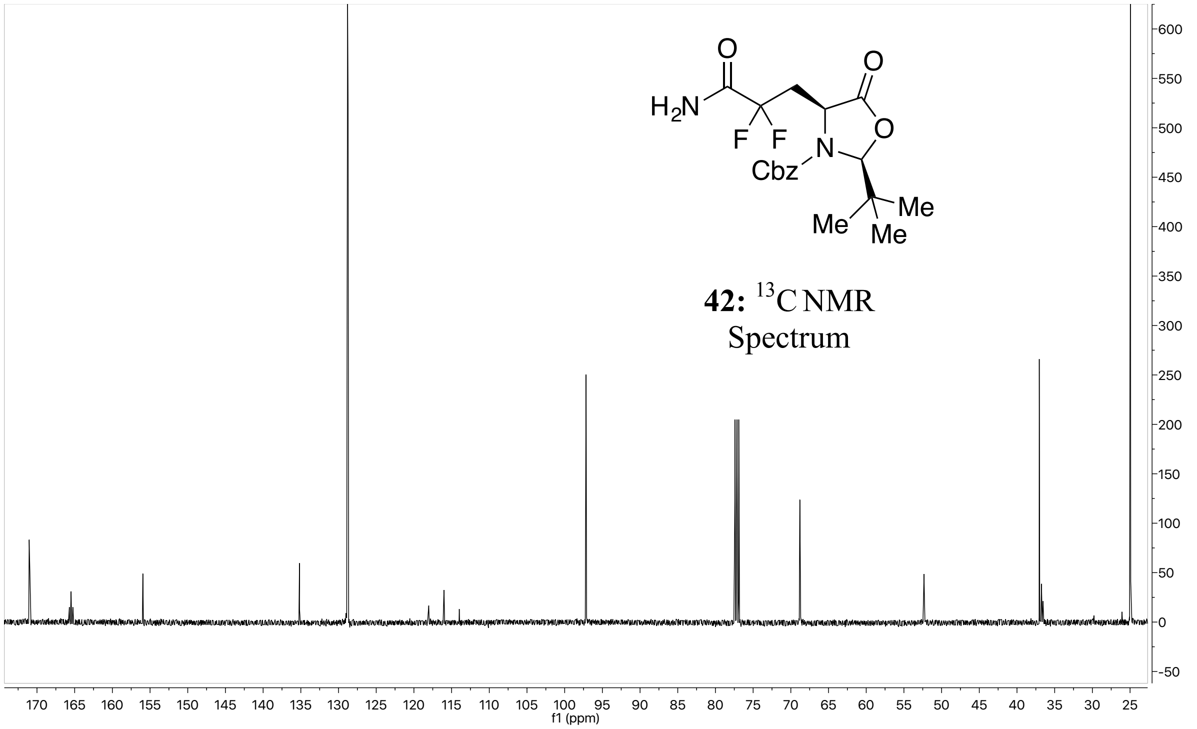

42: ¹³C NMR Spectrum
H2N
O
F F
N
Cbz
O
O
Me
Me
Me
f1 (ppm)

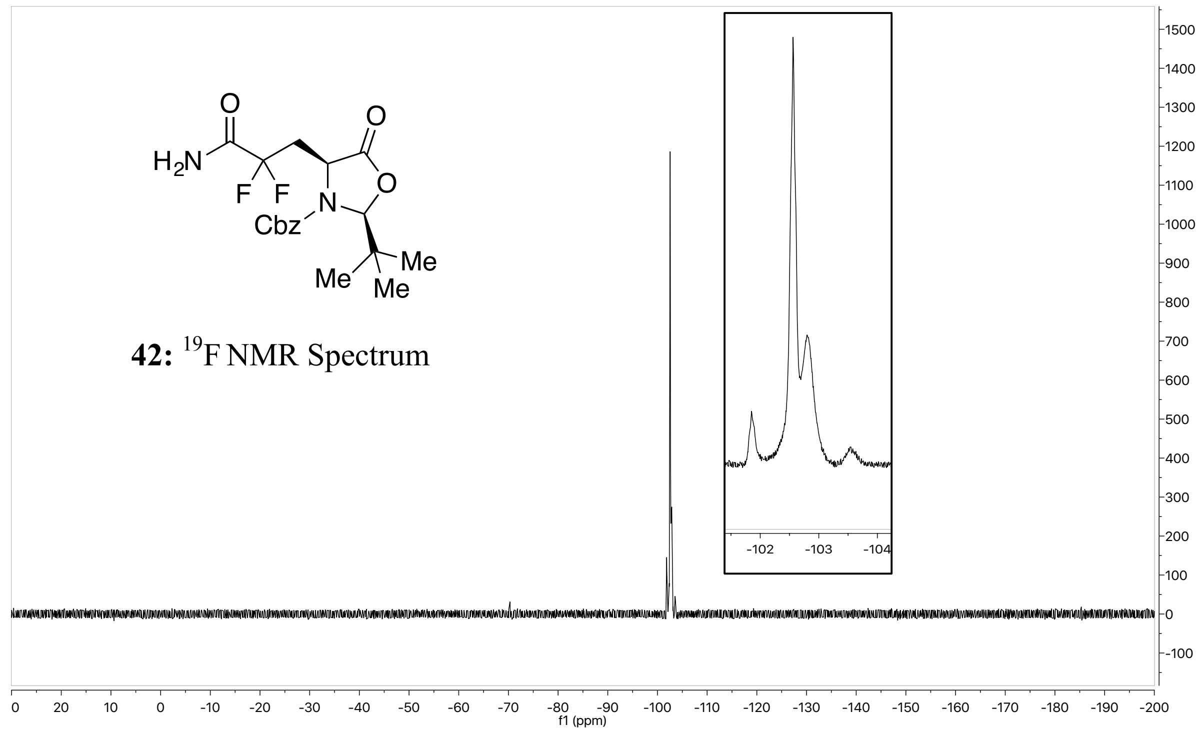
H2N
O
F F
O
N
O
Cbz
Me
Me
Me
42: 19F NMR Spectrum
f1 (ppm)

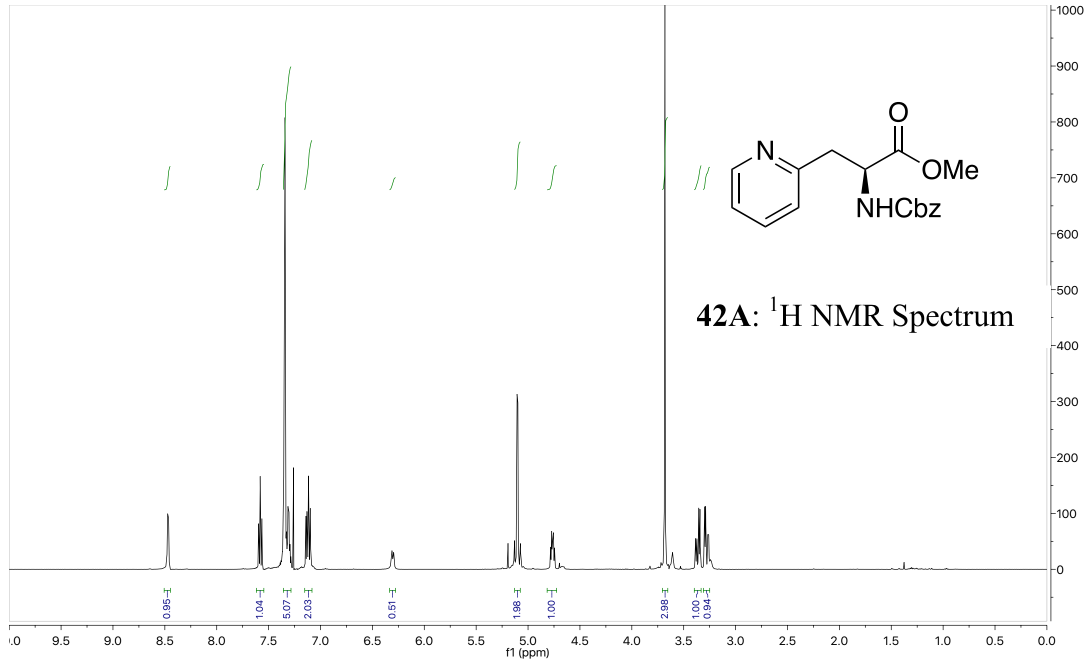
N
O
OMe
NHCbz
42A: 1H NMR Spectrum
f1 (ppm)

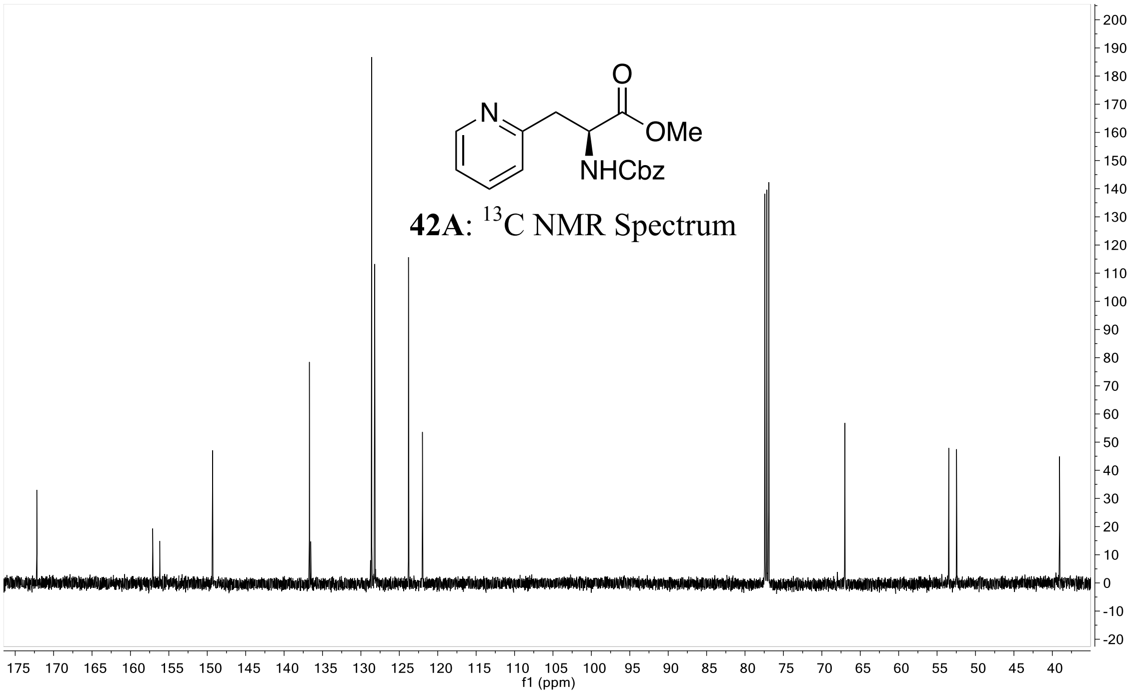

O
N
OMe
NHCbz
42A: 13C NMR Spectrum
f1 (ppm)

VIII. Chiral HPLC Data:

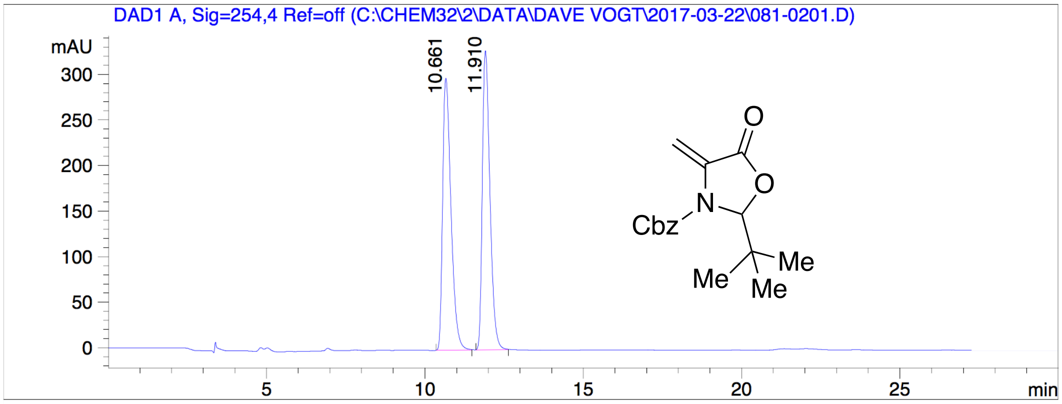

#	Compound Name	Amount	Resp.	Resp.%	Exp.RT	Meas.RT
1		0.000	5.459e3	49.955	0.000	10.661
2		0.000	5.469e3	50.045	0.000	11.910
Totals:		0.000				

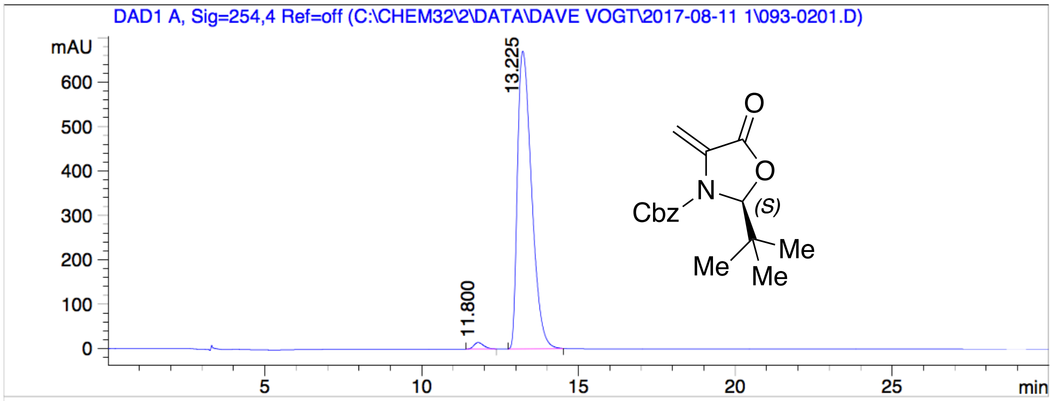

This is a result compounds table. Use the footer button in the table format dialog box to define the summations in the last table line.

#	Compound Name	Amount	Resp.	Resp.%	Exp.RT	Meas.RT
1		0.000	326.194	1.530	0.000	11.800
2		0.000	2.099e4	98.470	0.000	13.225
Totals:		0.000				

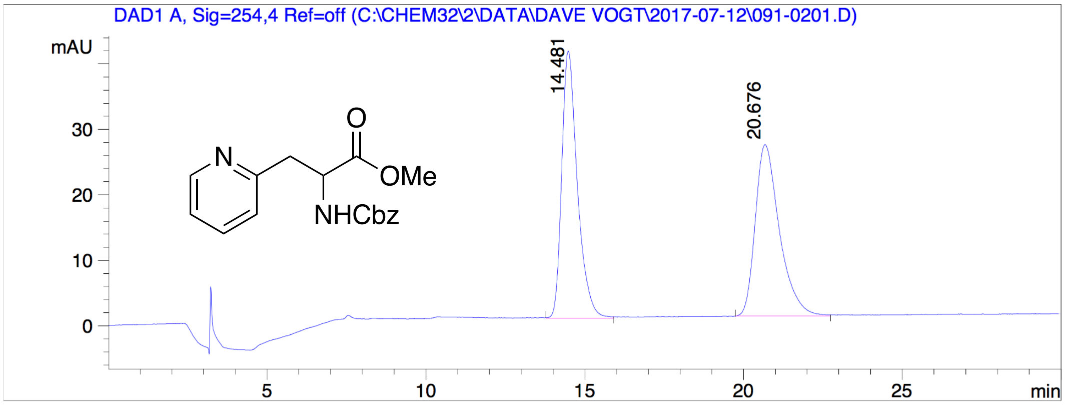

#	Compound Name	Amount	Resp.	Resp.%	Exp.RT	Meas.RT
1		0.000	1.399e3	50.170	0.000	14.481
2		0.000	1.389e3	49.830	0.000	20.676
Totals:		0.000				

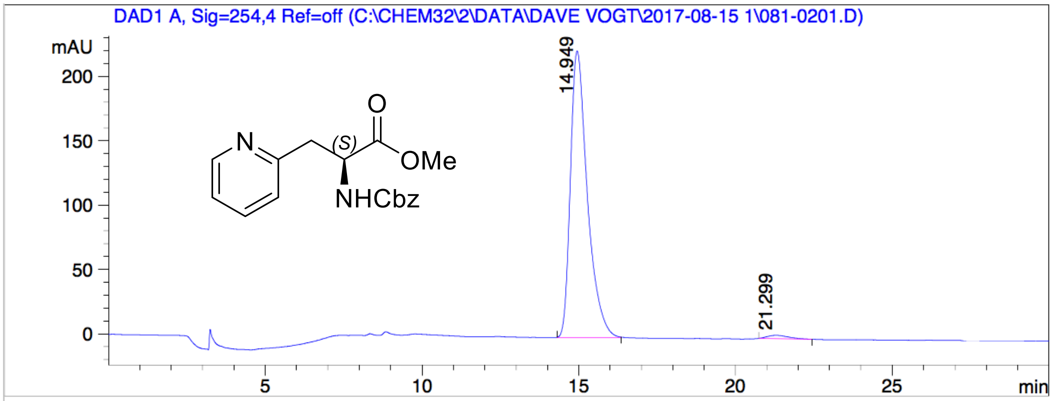

This is a result compounds table. Use the footer button in the table format dialog box to define the summations in the last table line.

#	Compound Name	Amount	Resp.	Resp.%	Exp.RT	Meas.RT
1		0.000	8.375e3	98.471	0.000	14.949
2		0.000	130.034	1.529	0.000	21.299
Totals:		0.000				

IX. Stern-Volmer Fluorescence Quenching Experiments:

All fluorescence measurements were recorded using a Horiba Scientific Dual-FL Fluorometer. Quenching studies were conducted in DMSO:H_2O (5:1) at 20 ±0.5 °C (Peltier temperature controller) with an $[Ir(ppy)_2(dtbbpy)]PF_6$ concentration of 5 μM. Raw fluorescence intensity was measured at λ = 591 nm after excitation at λ = 450 nm in a quartz cuvette with a path length of 1 cm. Measurements using Hantzsch ester, dehydroalanine, or 2-bromopyridine as quenchers were taken in triplicate at concentrations of 0, 50, 100, 250, and 500 μM. At quencher concentration of 0 μM, additional duplicate measurements were collected prior to successive quenchers to maintain accuracy. Stern-Volmer plots were generated using Igor Pro 7; data points were fit with a linear trend line.

Stern-Volmer Plots

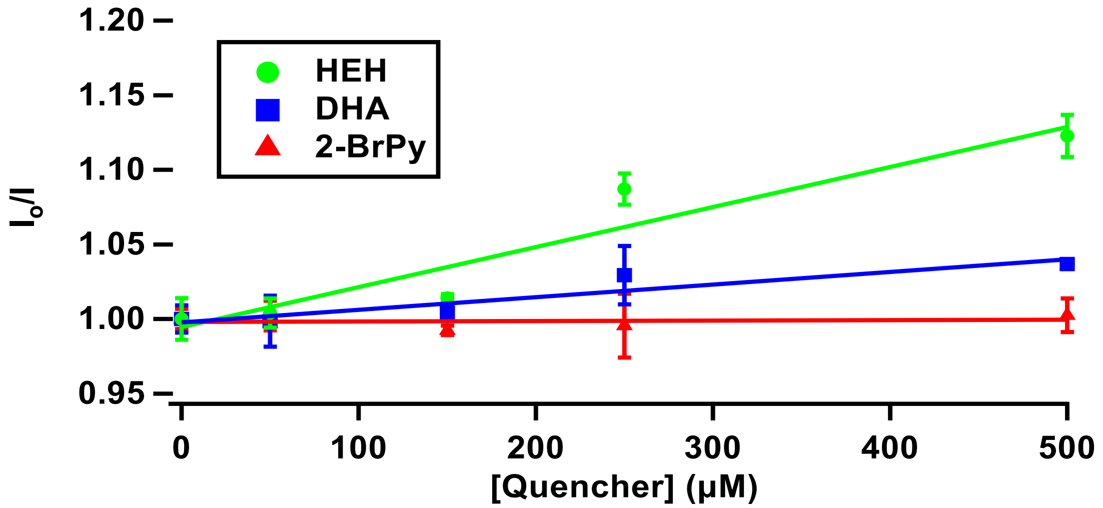

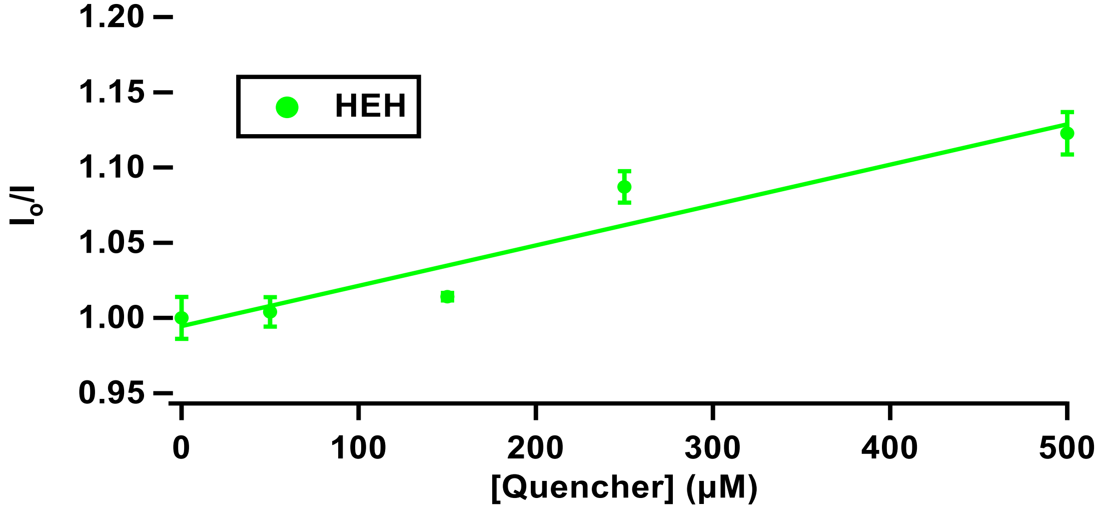

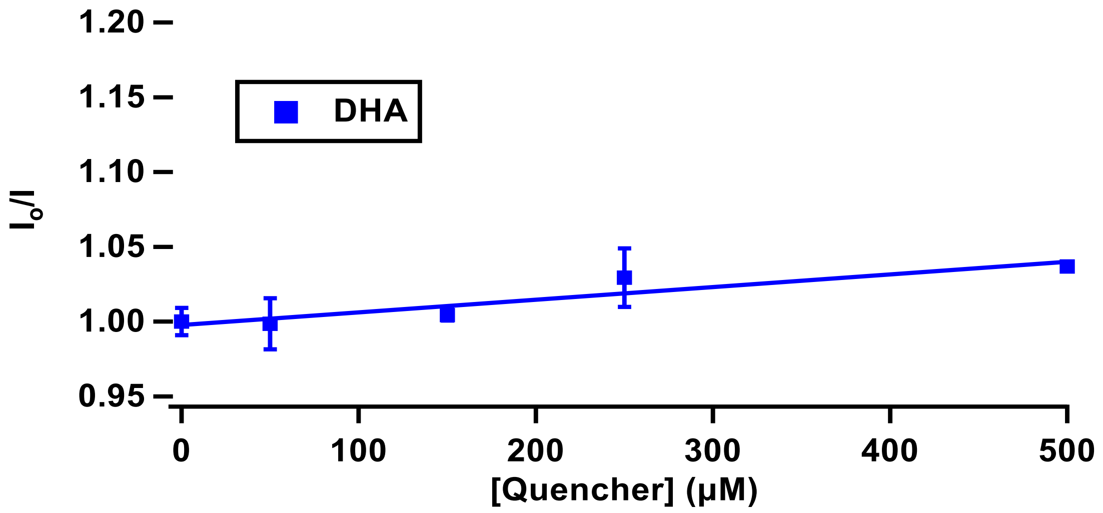

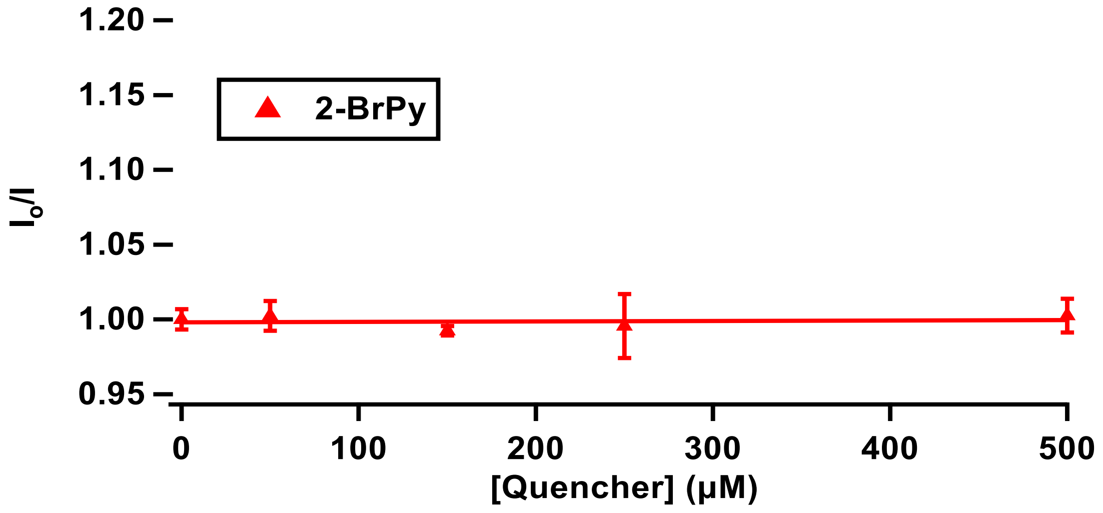

Measured Fluorescence Intensities at λ = 591 nm (counts)

[HEH] (μM)	1	2	3	avg	stdev	I_o/I	error
**0**	5966.1219	5849.4855	-	5907.8037	82.4743	1.0000	0.0140
**50**	5819.8148	5929.6318	5902.7293	5884.0586	57.2398	1.0040	0.0098
**100**	5841.5493	5819.6902	5813.9702	5825.0699	14.5553	1.0142	0.0025
**250**	5374.5285	5468.2221	5461.4238	5434.7248	52.2422	1.0870	0.0104
**500**	5200.5652	5253.6675	5331.9516	5262.0614	66.0942	1.1227	0.0141

[DHA] (μM)	1	2	3	avg	stdev	I_o/I	error
**0**	5839.3979	5914.3043	5903.3400	5885.6807	40.4553	1.0519	0.0072
**50**	6095.6538	6197.9486	6307.0152	6200.2059	105.6987	0.9985	0.0170
**100**	6173.1678	6181.4130	6138.4126	6164.3311	22.8216	1.0043	0.0037
**250**	6057.7748	6100.8092	5884.9329	6014.5056	114.2576	1.0294	0.0196
**500**	5968.5599	5976.2475	5967.5891	5970.7988	4.7436	1.0369	0.0008

[2-BrPy] (μM)	1	2	3	avg	stdv	I_o/I	error
**0**	6151.0503	6231.0219	-	6191.0361	56.5485	0.9507	0.0087
**50**	5805.9303	5890.0415	5918.6204	5871.5308	58.5812	1.0024	0.0100
**100**	5951.2881	5923.7698	5915.9019	5930.3199	18.5802	0.9925	0.0031
**250**	5829.0025	5848.4549	6057.4723	5911.6432	126.6656	0.9956	0.0213
**500**	5840.9751	5825.5125	5946.4111	5870.9662	65.7930	1.0025	0.0112

X. α-Deuteration With D_2O as Solvent:

Following the general procedure, the reaction of 2-bromopyridine (32 mg, 0.20 mmol, 1 equiv), methyl 2-(di(*tert*-butoxycarbonyl)amino)acrylate (120 mg, 0.40 mmol, 2 equiv), $[Ir(ppy)_2(dtbbpy)]PF_6$ (2.1 mg, 0.0021 mmol, 0.01 equiv) and Hantzsch ester (76 mg, 0.30 mmol, 1.3 equiv) provided the product (72 mg, 94% yield) as white solid after purification by flash column chromatography (5% – 40% ethyl acetate/hexanes). Integration of the alpha proton ^{1}H NMR signal was used to determine the percent of deuterium incorporation (94% D).

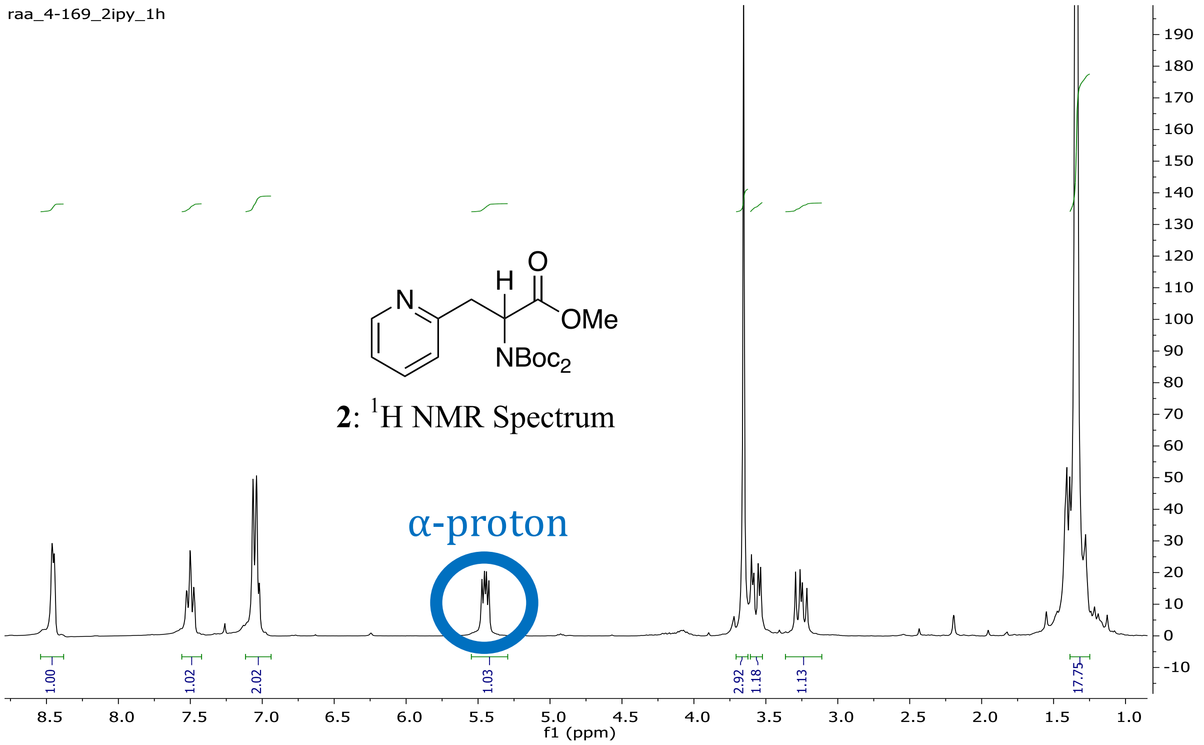

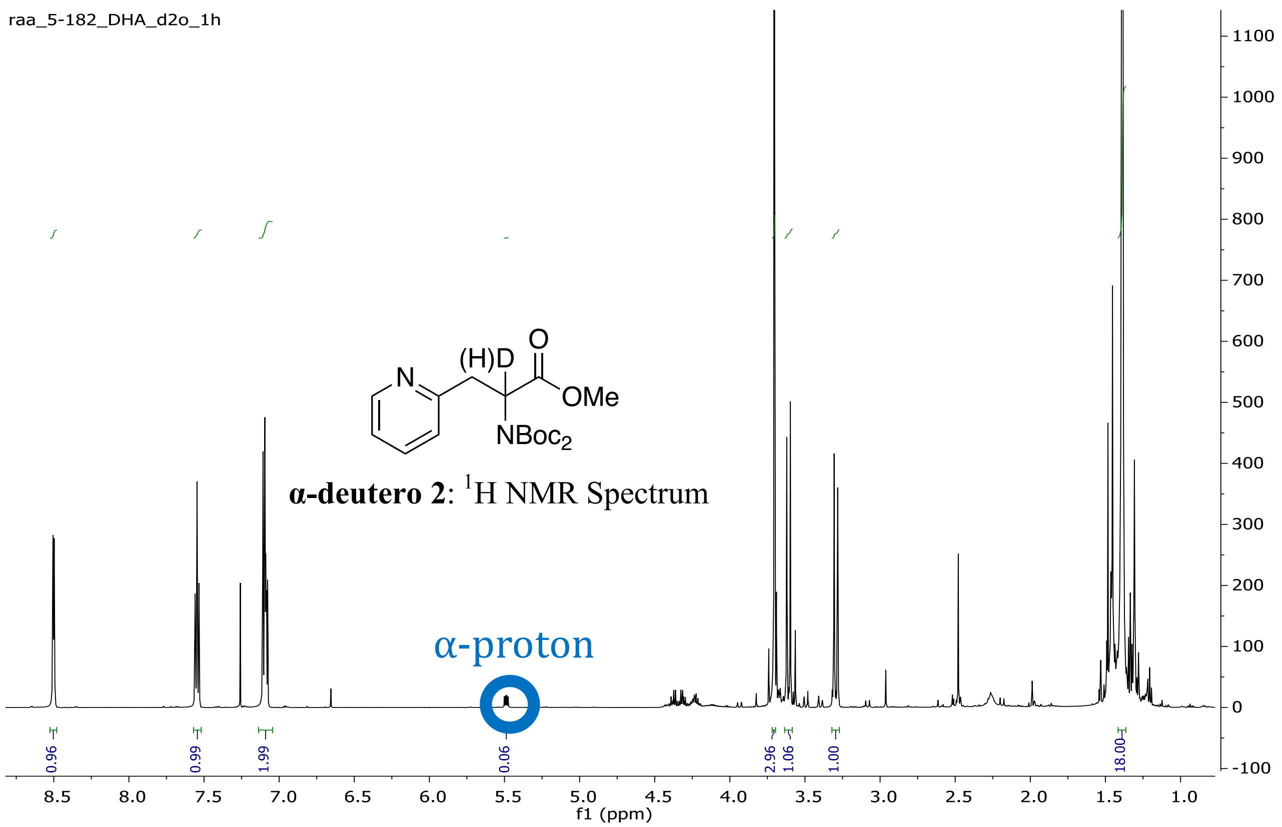
raa_5-182_DHA_d2o_1h
(H)D
N
O
OMe
NBoc$_2$
α-deutero 2: ^{1}H NMR Spectrum
α-proton
0.96
0.99
1.99
0.06
2.96
1.06
1.00
18.00
f1 (ppm)
